# Supplementary material for: Water-stable porous Al24 Archimedean solids for removal of trace iodine
Source: Nat Commun. 2022 Nov 4;13:6632. doi: 10.1038/s41467-022-34296-4 (PMC9636137; doi:10.1038/s41467-022-34296-4)
Supplement: Supplementary file 1 — Supplementary Information [file 41467_2022_34296_MOESM1_ESM.pdf]

Supplementary Information

Water-stable Porous Al_{24} Archimedean Solids for Removal of Trace Iodine

Liu et al.

Content

1. Supplementary Notes S3

1.1 Chemicals and Materials S3
1.2 Energy dispersive spectroscopies (EDS) S3
1.3 Fourier Transform Infrared (FT-IR) Spectroscopies S3
1.4 UV-vis spectroscopies S3
1.5 Powder X-ray diffractions (PXRD) S3
1.6 Thermogravimetric analyses S3
1.7 High Resolution Electrospray Ionization Mass Spectra (ESI-MS) S3
1.8 Raman spectra S3
1.9 X-ray photoelectron spectroscopies (*XPS*) S3
1.10 Gas Sorption Measurements S4
1.11 Contact Angle Measurements S4
1.12 Crystal photos and iodine adsorption videos S4
1.13 Drawing softwares S4
1.14 General methods for X-ray Crystallography S4

2. Supplementary Methods S5

3. Supplementary Figures S8

3.1 Synthesis S8
3.2 Detailed Structure Information for AlMCs S8
3.3 PXRD analysis for AlMC-1 to AlMC-8 .... S31
3.4 The EDS spectra for AlMC-1 to AlMC-8 .... S34
3.5 FT-IR spectra for AlMC-1 to AlMC-8 .... S37
3.6 UV-vis spectra of AlMC-1 to AlMC-8 .... S40
3.7 TGA curves S43
3.8 Stability, ESI-MS, contact angles and pore distribution S45
3.9 Iodine adsorption tests S53

4. Supplementary Tables S65

4.1 The sizes of Al_8 macrocycle S65
4.2 The solubility of AlMC-1 in different solvents S65
4.3 The ESI-MS data S66
4.4 Summary of sorbents for iodine ion capture in water S81
4.5 Crystallographic data S82
4.6 BVS analysis S88
4.7 Hydrogen bond parameters S91

5. Supplementary References S99

1. Supplementary Notes

1.1 Chemicals and Materials

All the reagents and solvents employed are purchased commercially and used as received without further purification. Aluminum isopropoxide ($Al(O^iPr)_3$) was acquired from Aladdin Chemical Reagent Shanghai. Ethyl alcohol (HOEt), n-propyl alcohol (HO^nPr), benzyl alcohol, benzoic acid (BA), nitric acid (HNO_3) and potassium iodide (KI) was bought from Sinopharm Chemical Reagent Beijing. Tetraethylammonium chloride, tetramethylammonium bromide, tetrabutylammonium iodide, tetrabutylammonium tetrafluoroborate, pyrazole and iodine (I_2) were purchased from Adamas-beta.

1.2 Energy dispersive spectroscopies (EDS)

The EDS analyses of single crystals were performed on a JEOL JSM6700F field-emission scanning electron microscope equipped with an Oxford INCA system.

1.3 Fourier Transform Infrared (FT-IR) Spectroscopies

FT-IR spectra (KBr pellets) were recorded on an ABB Bomem MB102 spectrometer.

1.4 UV-vis spectroscopies

The UV-vis diffuse reflection data were recorded at room temperature using powder samples with $BaSO_4$ as a standard (100% reflectance) on a PerkinElmer Lamda-950 UV spectrophotometer and scanned at 200-800 nm. The absorption data are calculated from the Kubelka-Munk function, ($F(R) = (1-R)^2/2R$), where R represents the reflectance.

1.5 Powder X-ray diffractions (PXRD)

PXRD data were collected on a Rigaku Mini Flex II diffractometer using CuKα radiation (λ = 1.54056 Å) under ambient conditions.

1.6 Thermogravimetric analyses

The thermogravimetric analyses (TGA) were performed on a Mettler Toledo TGA/SDTA 851e analyzer in a nitrogen atmosphere with a heating rate of 10 ^oC/min.

1.7 High Resolution Electrospray Ionization Mass Spectra (ESI-MS)

The dissolved samples used for ESI-MS measurements are firstly filtered through a membrane filter and diluted, then pumped into Impact II Q-TOF mass spectrometer. The experimental peaks were simulated by DataAnalysis software.

1.8 Raman spectra

Analysis of the Raman spectra was performed on Labram HR Evolution microscopic confocal Raman spectrometer.

1.9 X-ray photoelectron spectroscopies (*XPS*)

XPS was performed on ground powders using a Thermo Scientific ESCALAB 250 Xi spectrometer equipped with a monochromatic Al Kα X-ray source (1486.6 eV) operating at 300 W. Samples were analyzed under vacuum (P

<10^{-8} mbar) with a pass energy of 150 eV (survey scans) or 25 eV (high-resolution scans). A low-energy electron flood gun was employed for charge neutralization. Prior to the *XPS* measurements, the crystalline powders were pressed on copper foil, mounted on stubs, and successively put into the entry-load chamber to pump. All peaks were referenced to the signature C 1*s* peak binding energy at 284.8 eV for adventitious carbon. The experimental peaks were fitted with Avantage software.

1.10 Gas Sorption Measurements

Before gas sorption, the crystals should be soaked in solvent for three days. During this time, the solvent should be exchanged several times a day. Then, the samples were degassed under a dynamic vacuum at 80 or 100 ^{o}C for 6h to obtain activated samples. The gas adsorption isotherms of active samples were obtained on a Micromeritics ASAP 2020 volumetric adsorption instrument.

1.11 Contact Angle Measurements

Contact angles were tested on powder samples by using a contact angle meter with a rotatable substrate holder. 10 mg of powder samples were deposited on a glass substrate bed and flattened by glass slide. A ~20 μL water droplet was released slowly to the flat surface of the powder samples. Later, the droplet image was captured using a high-performance charge-coupled device (CCD) sensor. Five-point simulation analysis was used to analyze the contact angles of all the powder samples.

1.12 Crystal photos and iodine adsorption videos

Crystal photos and iodine adsorption videos were obtained on a LW500LJT infinite high-definition transmission-reflection metallographic microscope with a 20-megapixel color imaging system.

1.13 Drawing softwares

The structural diagrams are plotted using Diamond 4.6.5. The channel simulations were realized by using Mercury 2021.3.0. The Al_{24} molecular model was constructed by Cinema 4D R19. The cavity volume of the Al_{24} ***tcu*** cage was calculated via the 3V Volume Assessor program.

1.14 General methods for X-ray Crystallography

Crystallographic data were collected on a Rigaku Synergy Custom (Liquid MetalJet D2+) diffractometer with Ga K*α* radiation (λ = 1.3405 Å). The structures were solved with direct methods using OLEX^2 and refined by full-matrix least-squares on F^2 using *SHELXTL*.[2] All hydrogen atoms were theoretical hydrogenation, riding on the relevant atoms and refined with fixed thermal factors. Non-hydrogen atoms were refined anisotropically. Some alcohol ligands or guests are severely disordered, thus, the related hydrogen atoms could not be added (**AlOC-60**, **AlMC-3**, **AlMC-7**). A comparison of the Fourier difference maps for the bare and I-loaded samples revealed the existence of new electron density peaks of iodine species. The obtained crystallographic data for **AlOC-60**, **AlMC-1** to **AlMC-8** as well as I-loaded crystals are summarized in Supplementary Table 10 to 15. The A- or B- level alertes in CheckCif files may be owing to the severe disorder of guest molecules and poor crystal quality after iodine adsorption. Besides, the diffraction peaks at high angles for this series compounds are very weak, which is common in high-nuclear clusters. Thus, the data integrity for some compounds cannot reach 100% and some guest molecues in pore cannot be defined although we have made several attempts to obtain better quality data. Combining with other characterization, we are confident that the structure modes for this series compounds are valid.

2. Supplementary Methods

Amphoteric Al^{3+} ions, behaving as acids in bases and bases in acids, are readily hydrated in an aqueous solution to form octahedral $Al(H_2O)_6^{3+}$ at pH < 3.0 but form the tetrahedral $Al(OH)_4^-$ at pH > 7.[1-6] Polynuclear species are isolated upon additional titration with a hard base NaOH, including a range of Keggin-type cations such as $[Al_{13}O_4(OH)_{24}(H_2O)_{12}]^{7+}$,[7] $[Al_{30}O_8(OH)_{56}(H_2O)_{24}]^{18+}$,[8-9] $[Al_{32}O_8(OH)_{60}(H_2O)_{28}(SO_4)_2]^{16+}$ (S-Al_{32}),[10] and $[Ge_4O_{16}Al_{48}(OH)_{108}(H_2O)_{24}]^{20+}$ (Al_{48}) (Supplementary Figure 1).[11] Another structural arrangement is found in the ligand-stabilized Brucite-like molecular cations $Al_8(\mu_3\text{-}OH)_2(\mu_2\text{-}OH)_{12}(H_2O)_{12}^{10+}$,[12] $Al_{13}(\mu_3\text{-}OH)_6(\mu_2\text{-}OH)_{18}(H_2O)_6^{15+}$ cores,[13] and $[Al_{15}(\mu_3\text{-}O)_4(\mu_3\text{-}OH)_6(\mu\text{-}OH)_{14}(hpdta)_4]^{3-}$ (Al_{15}).[14] Recently, our group discovered a novel structural type of neutral aluminum molecular rings via solvothermal synthesis, involving the controlled hydrolysis of aluminum alkoxides $Al(OR)_3$, in the presence of organic acids.[15-16] Relevant to the current work, inorganic acids have been found to provide excellent anionic templates, and can also provide counter ions for other templates in the synthesis of porous structures.[17] Thus, we wondered how the aggregation of aluminum oxo compounds would be affected by the presence of inorganic acids, since this strategy has been successfully applied in the field of transition metal/rare earth oxo cages,[18-21] and mesoporous silica.[22] Our synthetic approach in the current work involves the hydrolysis of $Al(O^iPr)_3$ in the presence of benzoic acid (BA) and $HNO_{3(aq)}$ in alcohol solvents with an anionic template. Regulators, such as H_2O, benzyl alcohol, quaternary ammonium salts and pyrazole, are also added which affect crystal growth and supramolecular assembly in the solid state. A series of closely related host-guest cage compounds based on the novel porous cationic host $[Al_{24}(BA)_{12}(\mu_2\text{-}OR)_{24}(\mu_2\text{-}OH)_{24}(\mu_3\text{-}OH)_8]^{4+}$ (R = Et, ^{i}Pr) (Al_{24}) were obtained using these reaction conditions (see below); $Al_{24}\cdot(NO_3)_4\cdot(HOEt)_2\cdot(H_2O)_2$ (**AlMC-1**), $Al_{24}\cdot(NO_3)_4\cdot(HO^nPr)_4$ (**AlMC-2**), $Al_{24}\cdot(NO_3)_2\cdot(OEt)_2\cdot(HOEt)_4$ (**AlMC-3**), $Al_{24}\cdot NO_3\cdot Cl_3$ (**AlMC-4**), $Al_{24}\cdot NO_3\cdot Br_3$ (**AlMC-5**). $Al_{24}\cdot I_4$ (**AlMC-6**), $Al_{24}\cdot(HNO_3)\cdot(OEt)_6\cdot(Al_6(BA)_6(OEt)_6(NO_3)_2)_{0.5}$ (**AlMC-7**), and $Al_{24}\cdot NO_3\cdot Br_2\cdot OEt$ (**AlMC-8**). The single-crystal structures of the complexes are discussed in the next section. This is the first time formation of polynuclear Al^{3+} species has been studied in the presence of inorganic acids. The cationic Al_{24} cage has a porous core(inorganic)-shell(organic) structure and is probably derived from the condensation of metallocycles $Al_8(\mu_2\text{-}OH)_8(BA)_8(OEt)_8$ (**AlOC-60**) (Supplementary Figures 2 and 3).

Synthesis of $Al_8(BA)_8(\mu_2\text{-}OEt)_8(\mu_2\text{-}OH)_8$ (AlOC-60). $Al(O^iPr)_3$ (1.220 g, 6 mmol), BA (0.365 g, 3 mmol), tetrabutylammonium tetrafluoroborate (0.33 g, 1 mmol) were dissolved in HOEt (8 mL) at room temperature. The resultant solution was heated at 80 ^oC for three days. After cooling to room temperature, colorless block crystals of **AlOC-60** were obtained (yield: ~245 mg, ~19% based on $Al(O^iPr)_3$).

Synthesis of $Al_{24}(BA)_{12}(\mu_2\text{-}OEt)_{24}(\mu_2\text{-}OH)_{24}(\mu_3\text{-}OH)_8\cdot(NO_3)_4\cdot(HOEt)_2\cdot(H_2O)_2$ (**AlMC-1**). **AlMC-1** was obtained by introducing HNO_3 into the reaction system of **AlOC-60**. The detailed synthesis conditions are as follows. $Al(O^iPr)_3$ (1.220 g, 6 mmol), BA (0.365 g, 3 mmol) were dissolved in HOEt (8 mL), then 100 μL HNO_3 was added to the bottle and mixed at room temperature. The resultant solution was heated at 80 ^oC for three days. After cooling to room temperature, colorless rectangular crystals of **AlMC-1** were obtained (yield: ~113 mg, ~11% based on $Al(O^iPr)_3$). It's worth noting that the introduction of a small amount of water (60 μL) can accelerate the hydrolysis and also improve the quality and yield of compound **AlMC-1** (yield: ~441 mg, ~43% based on $Al(O^iPr)_3$). Elemental analysis calcd. (%) for $C_{136}H_{228}Al_{24}N_4O_{96}$ (MW 4102.73): C 39.81, H 5.60, N 1.37; found: C 38.56, H 6.02, N 1.03.

Scale-up synthesis of AlMC-1. A mixture of $Al(O^iPr)_3$ (6.0 g), BA (1.8 g), HNO_3 (0.5 mL), H_2O (300 μL), and HOEt (40 mL) was sealed in an 80 mL vial and heated at 80 °C for 7 days. When cooled to room temperature, the white precipitate and colorless crystals were washed with ethanol over and over. After drying, the precipitate and crystals are sieved by 200-mesh sieve, and pure-phase rectangular crystals (~50μm) can be obtained. (Yield: ~817 mg, ~16% based on $Al(O^iPr)_3$).

Synthesis of $Al_{24}(BA)_{12}(\mu_2\text{-}O^nPr)_{24}(\mu_2\text{-}OH)_{24}(\mu_3\text{-}OH)_8\cdot(NO_3)_4\cdot(HO^nPr)_4$ (**AlMC-2**). $Al(O^iPr)_3$ (1.225 g, 6 mmol), BA (0.362 g, 3 mmol) were dissolved in HO^nPr (8 mL), then 5 drops HNO_3 were added to the bottle and mixed at room temperature. The resultant solution was heated at 80 ^oC for three days. After cooling to room temperature, colorless block crystals of **AlMC-2** were obtained (yield: ~113 mg, ~10% based on $Al(O^iPr)_3$). Elemental analysis calcd. (%) for $C_{168}H_{292}Al_{24}N_4O_{96}$ (MW 4551.56): C 44.33, H 6.46, N 1.23; found: C 45.61, H 6.78, N 0.94.

Synthesis of $Al_{24}(BA)_{12}(\mu_2\text{-}OEt)_{24}(\mu_2\text{-}OH)_{24}(\mu_3\text{-}OH)_8\cdot(NO_3)_2\cdot(OEt)_2\cdot(HOEt)_4$ (**AlMC-3**). $Al(O^iPr)_3$ (1.223 g, 6 mmol), BA (0.367 g, 3 mmol), benzyl alcohol (1 mL) were dissolved in HOEt (7 mL), then 5 drops HNO_3 were added to the bottle and mixed at room temperature. The resultant solution was heated at 80 ^oC for three days. After cooling to room temperature, colorless globular crystals of **AlMC-3** were obtained (yield: ~361 mg, ~35% based on $Al(O^iPr)_3$). Elemental analysis calcd. (%) for $C_{144}H_{246}Al_{24}N_2O_{92}$ (MW 4124.93): C 41.93, H 6.01, N 0.68; found: C 42.24, H 5.72, N 0.36.

Synthesis of $Al_{24}(BA)_{12}(\mu_2\text{-}OEt)_{24}(\mu_2\text{-}OH)_{24}(\mu_3\text{-}OH)_8\cdot NO_3\cdot Cl_3$ (**AlMC-4**). $Al(O^iPr)_3$ (1.222 g, 6 mmol), BA (0.359 g, 3 mmol), tetraethylammonium chloride (0.236 g, 1.43 mmol) were dissolved in HOEt (8 mL), then 5 drops HNO_3 were added to the bottle and mixed at room temperature. The resultant solution was heated at 80 ^oC for three days. After cooling to room temperature, colorless rhombus crystals of **AlMC-4** were obtained (yield: ~302 mg, ~31% based on $Al(O^iPr)_3$). Elemental analysis calcd. (%) for $C_{132}H_{212}Al_{24}Cl_3NO_{83}$ (MW 3894.88): C 40.70, H 5.48, N 0.36; found: C 41.68, H 5.03, N 0.52.

Synthesis of $Al_{24}(BA)_{12}(\mu_2\text{-}OEt)_{24}(\mu_2\text{-}OH)_{24}(\mu_3\text{-}OH)_8\cdot NO_3\cdot Br_3$ (**AlMC-5**). $Al(O^iPr)_3$ (1.228 g, 6 mmol), BA (0.362 g, 3 mmol), tetramethylammonium bromide (0.370 g, 2.40 mmol) were dissolved in HOEt (8 mL), then 5 drops HNO_3 were added to the bottle and mixed at room temperature. The resultant solution was heated at 80 ^oC for three days. After cooling to room temperature, colorless cube crystals of **AlMC-5** were obtained (yield: ~685 mg, ~68% based on $Al(O^iPr)_3$). Elemental analysis calcd. (%) for $C_{132}H_{212}Al_{24}Br_3NO_{83}$ (MW 4028.18): C 39.36, H 5.30, N 0.35; found: C 38.51, H 5.09, N 0.48.

Synthesis of $Al_{24}(BA)_{12}(\mu_2\text{-}OEt)_{24}(\mu_2\text{-}OH)_{24}(\mu_3\text{-}OH)_8\cdot I_4$ (**AlMC-6**). $Al(O^iPr)_3$ (1.227 g, 6 mmol), BA (0.362 g, 3 mmol), tetrabutylammonium iodide (0.369 g, 1 mmol) were dissolved in HOEt (8 mL), then 5 drops HNO_3 were added to the bottle and mixed at room temperature. The resultant solution was heated at 80 ^oC for three days. After cooling to room temperature, yellow dodecahedral crystals of **AlMC-6** were obtained (yield: ~794 mg, ~75% based on $Al(O^iPr)_3$). Elemental analysis calcd. (%) for $C_{132}H_{212}Al_{24}I_4O_{80}$ (MW 4234.12): C 37.44, H 5.05; found: C 38.98, H 4.79.

Synthesis of $Al_{24}(BA)_{12}(\mu_2\text{-}OEt)_{24}(\mu_2\text{-}OH)_{24}(\mu_3\text{-}OH)_8\cdot(HNO_3)\cdot(OEt)_6\cdot(Al_6(BA)_6(OEt)_6(NO_3)_2)_{0.5}$ (**AlMC-7**).

$Al(O^iPr)_3$ (1.225 g, 6 mmol), BA (0.360 g, 3 mmol), and pyrazole (0.201 g, 3 mmol) were dissolved in HOEt (8 mL), then 5 drops HNO_3 were added to the bottle and mixed at room temperature. The resultant solution was heated at 80 ^oC for three days. After cooling to room temperature, colorless fusiform crystals of **AlMC-7** were obtained (yield: ~411 mg, ~35% based on $Al(O^iPr)_3$). Elemental analysis calcd. (%) for $C_{171}H_{273}Al_{27}N_2O_{101}$ (MW 4701.36): C 43.68, H 5.85, N 0.60; found: C 42.25, H 5.34, N 0.38.

Synthesis of $Al_{24}(BA)_{12}(\mu_2\text{-OEt})_{24}(\mu_2\text{-OH})_{24}(\mu_3\text{-OH})_8\cdot NO_3\cdot Br_2\cdot OEt$ (**AlMC-8**). 3 mg **AlMC-5** dissolved in MeCN (3 mL) at room temperature. The lid of the bottle should be unscrewed to allow the evaporation of the solvent. After one-week, colorless parallelogram crystals of **AlMC-8** were obtained (yield: ~1 mg, ~34% based on **AlMC-5**).

3. Supplementary Figures

3.1 Synthesis

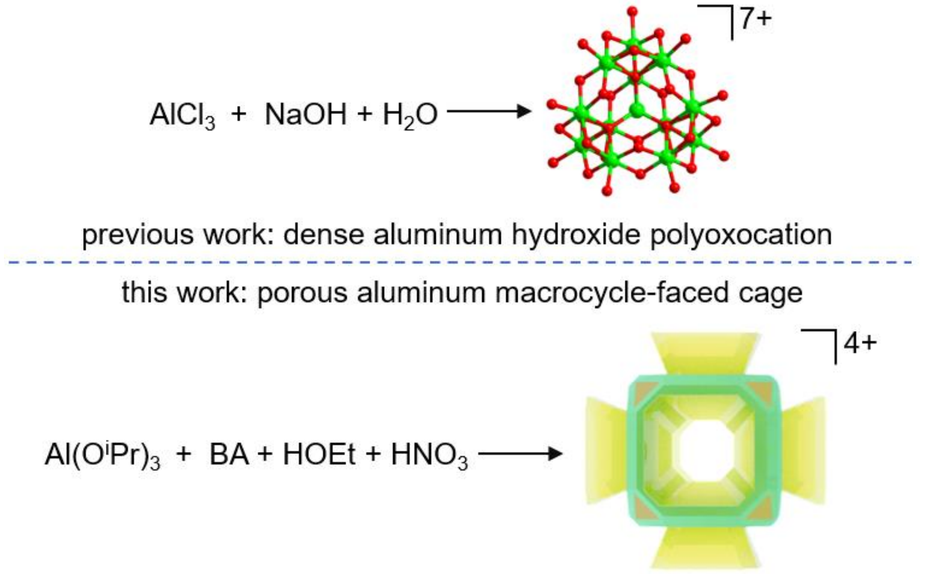

Supplementary Figure 1. Comparison of the synthetic route. Top: the synthetic method used in the 'traditional' aluminum hydroxide polyoxocations (taking $[\varepsilon\text{-}Al_{13}O_4(OH)_{24}(H_2O)_{12}]^{7+}$ [23] as an example); Bottom: the synthetic method for the porous **AlMCs** described in the current work.

3.2 Detailed Structure Information for AlMCs

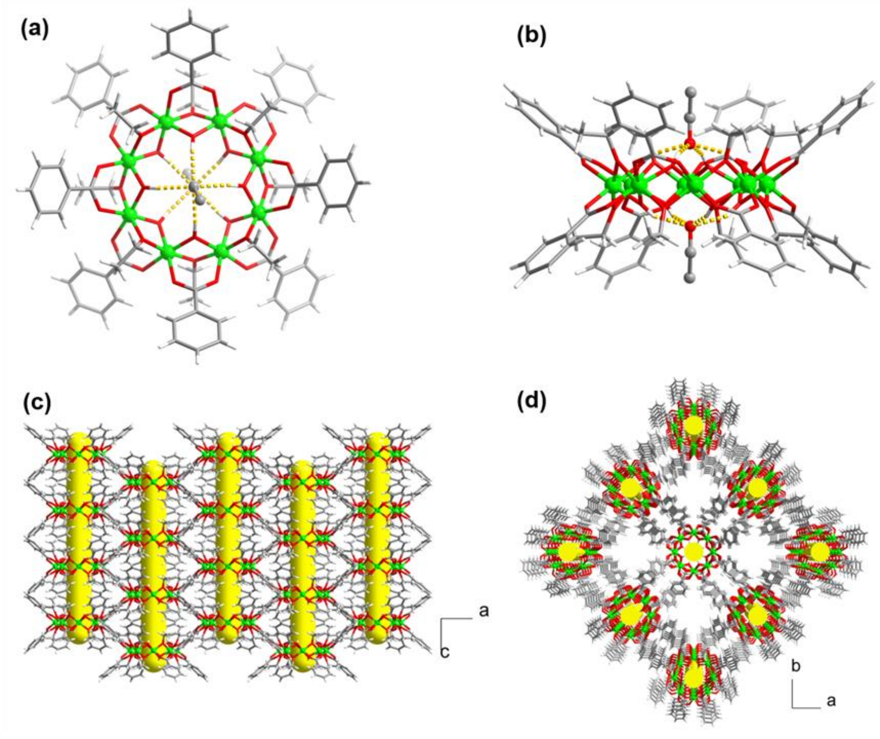

Supplementary Figure 2. Molecular structure of **AlOC-60**. (a) top view; (b) side view; (c and d) packing view.

AlOC-60 crystallizes in the tetragonal *P*4/nnc space group, and consists of one 8-membered aluminum molecular ring and one ethanol guest (Supplementary Figure 2a). The planar aluminum molecular ring composes of eight Al^{3+}, eight alcoholate groups, eight benzoate ligands and eight μ_2-OH groups. Of these, the eight μ_2-OH groups point to the center of the ring, whereas alcoholate and benzoate ligands coordinated to the upper and lower sides of the ring. Notably, the alcohol guest is disordered on two sites and the occupancy of each site is 0.5 (Supplementary Figure 2b). Interestingly, these rings form parallel supermolecule nanotubes (Supplementary Figure 2c), and are further organized into a rhombic array (Supplementary Figure 2d).

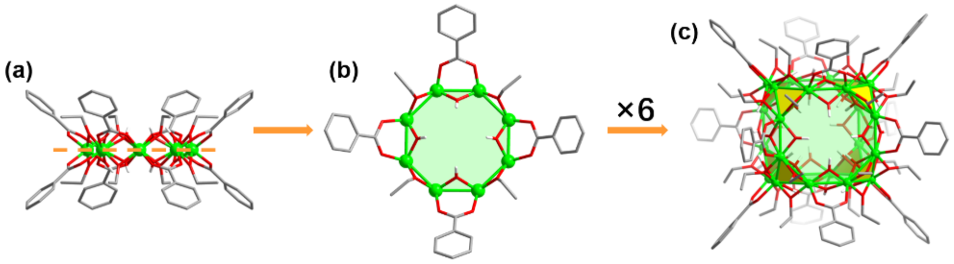

Supplementary Figure 3. Deduction on possible structural evolution from **AlOC-60** to **AlMC**. (a) The aluminum molecular ring in **AlOC-60**; (b) the half of the aluminum molecular ring; (c) the Al_{24} cage.

The coplanar Al centers in **AlOC-60** are bridged by 8 μ_2-OH groups, 8 benzoates and 8 alkoxides. Coincidentally, the ligand quantities in this ring compound are double those in one octagonal face of the Al_{24} cage, and half of the aluminum molecular ring in **AlOC-60** (Supplementary Figures 3a and 3b) is also great similar to the octagon face in **AlMC**. Therefore, we infer that the cage for **AlMC** is probably condensed from **AlOC-60** (Supplementary Figure 3c).

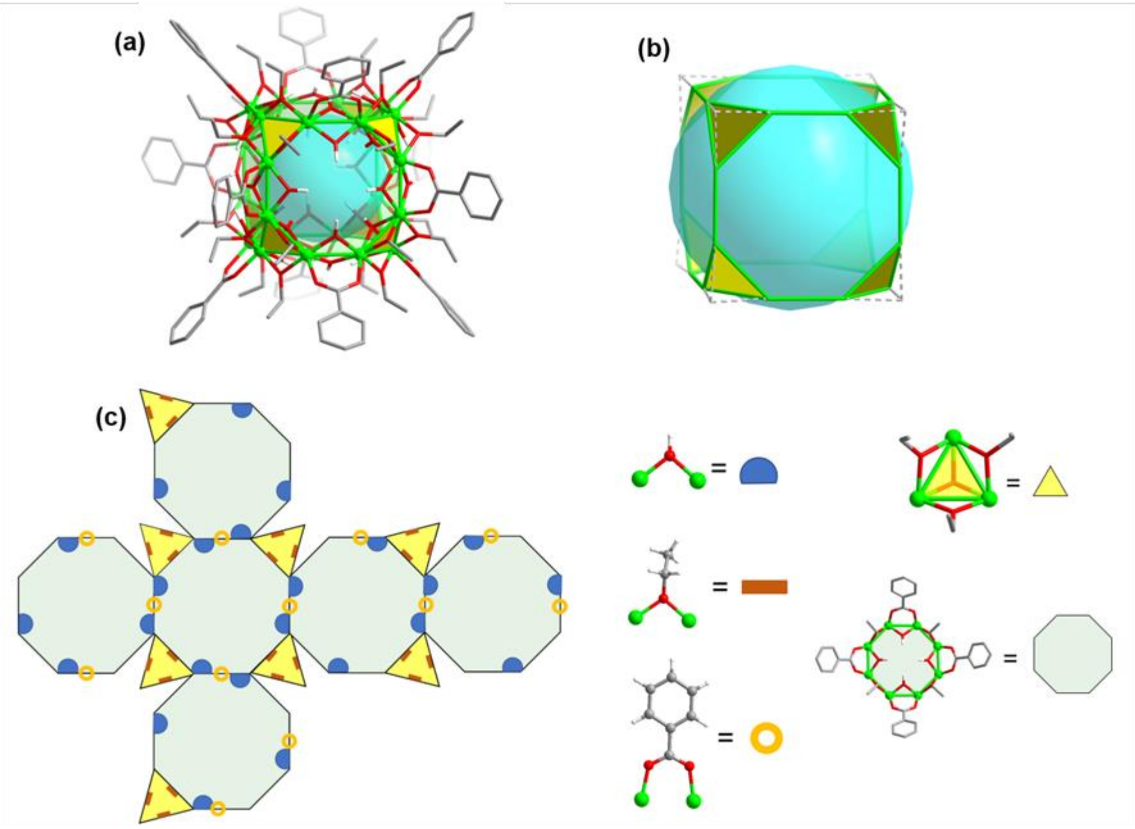

Supplementary Figure 4. The structure analysis of Al_{24} cage. (a) The ***tcu*** cage in $[Al_{24}(BA)_{12}(\mu_2\text{-OR})_{24}(\mu_2\text{-OH})_{24}(\mu_3\text{-OH})_8]^{4+}$ (Al: bright green; O: red; C: grey; H: white). (b) Eight truncated vertices are highlighted in gray dotted lines. (c) Schematic representation of an unfolded ***tcu*** cage.

Single-crystal X-ray diffraction (SCXRD) revealed that this series of compounds contains an organic-inorganic hybrid cage with the formula of $[Al_{24}(BA)_{12}(\mu_2\text{-OR})_{24}(\mu_2\text{-OH})_{24}(\mu_3\text{-OH})_8]^{4+}$ (R=Et, ^{n}Pr) (Supplementary Figure 4a). Such a cage contains one kind of vertices, two kinds of edges, and two kinds of face, thus is recognized as dice-shaped Archimedean truncatedhexahedron (***tcu***) cage (Supplementary Figures 4b and 4c), which can be described by a $[3^8.8^6]$ tiling with O_h symmetry (Supplementary Figure 4c). 24

Al vertices are in octahedral coordination environments. Of the 36 edges, 12 of them are common edges of two adjacent octagons, which are occupied by benzoate and two μ$_2$-OH. While the other 24 edges are common edges of adjacent triangular and octagonal faces, that are taken up by alkoxide ligands. All of the benzoate, alkoxide and OH ligands adopt a μ$_2$-coordination mode to bridge adjacent Al vertices. The triangular face in the ***tcu*** cage consists of three Al vertices, three μ$_2$-OR and one μ$_3$-OH group. Although such a trinuclear fragment has been widely found in annular,[24] spherical,[25] lamellar compounds[26] as well as polyoxometalates,[27] the direct assembly of this fragment into a ***tcu*** cage has not been reported. The octagonal face is composed of two types of edges that are arranged alternately, in which all μ$_2$-OH groups point to the center of the octagons (Supplementary Figure 5). In conclusion, the ***tcu*** cage is composed of 92 components, namely, 24 Al ions located at vertexes, 12 benzoic acids and 24 μ$_2$-OH groups occupying the common edges of adjacent octagonal surfaces, 24 alkoxide situated at the common edges of triangles and octagons, and 8 μ$_3$-OH groups pointed to the body center of the cage. Bond valence sum (BVS) calculations show that the valence of Al ion is + 3, and that of all OH groups is close to –1 (Supplementary Tables 16–23). Therefore, the hole organic-inorganic hybrid ***tcu*** cage carries four positive charges.

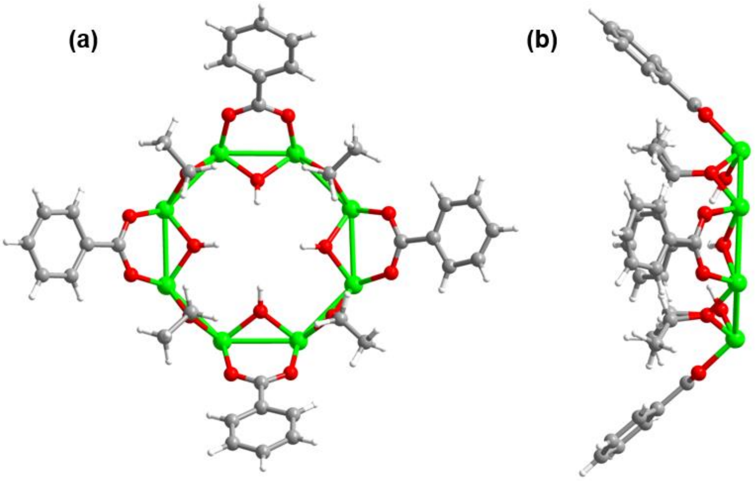

Supplementary Figure 5. The octagonal faces in the ***tcu*** cage contain eight Al^{3+} ions, four benzoates, four alkoxides and four μ$_2$-OH groups. (a) Top view; (b) side view. All μ$_2$-OH groups point to the face center of the octagon.

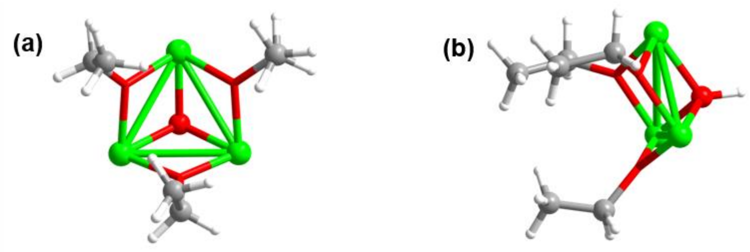

Supplementary Figure 6. The triangular face in the ***tcu*** cage consists of three Al ions bonded by one μ$_3$-OH and three μ$_2$-OR groups. (a) top view; (b) side view.

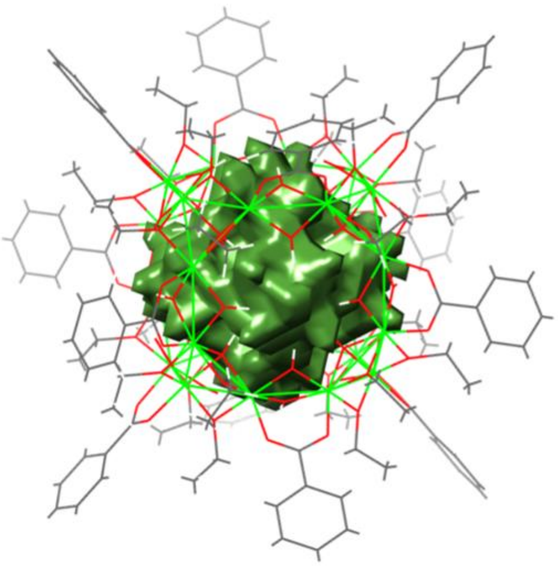

Supplementary Figure 7. The surface and cavity of Al_{24} ***tcu*** cage were calculated via the 3V Volume Assessor program. The calculated surface area is 947 $Å^2$, and the calculated volume is 320 $Å^3$.

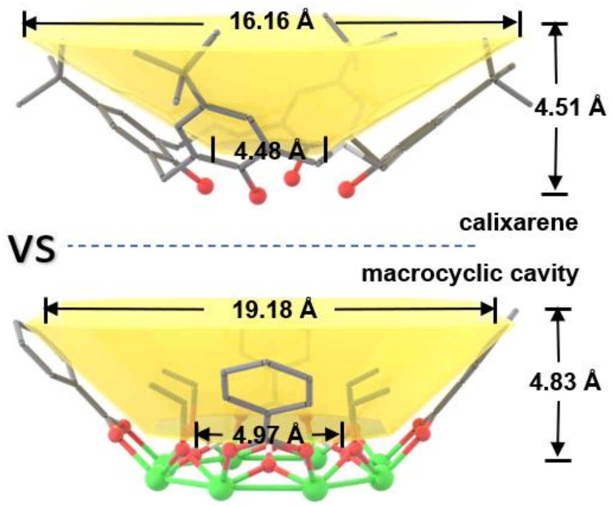

Supplementary Figure 8. The comparison between a calixarene and the macrocyclic cavity in the Al_{24} ***tcu*** cage.

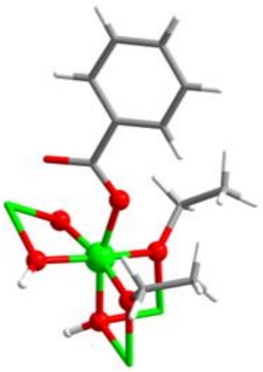

Supplementary Figure 9. The octahedral coordination environment of the Al centers. 24 octahedral Al^{3+} cations are located in the same coordination environment with one -O_{COO} from benzoate, one μ_3-OH, two μ_2-OH and two alkoxide ligands.

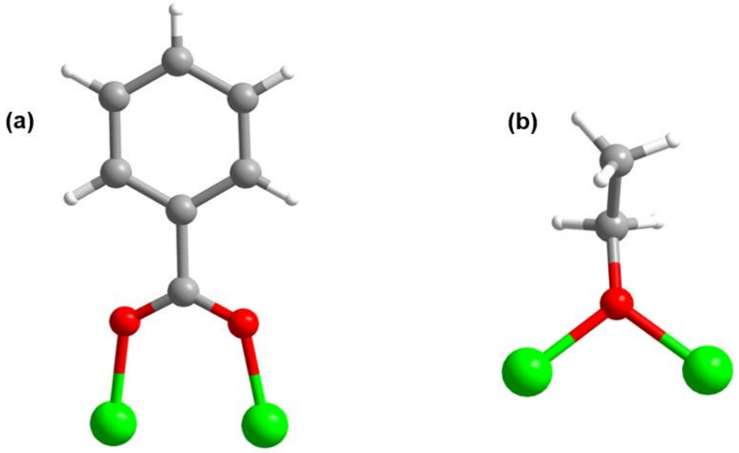

Supplementary Figure 10. The coordination modes of benzoate and alkoxide. (a) benzoate; (b) alkoxide. All benzoate and alkoxide adopt μ_2-coordination modes to bridge adjacent Al vertices.

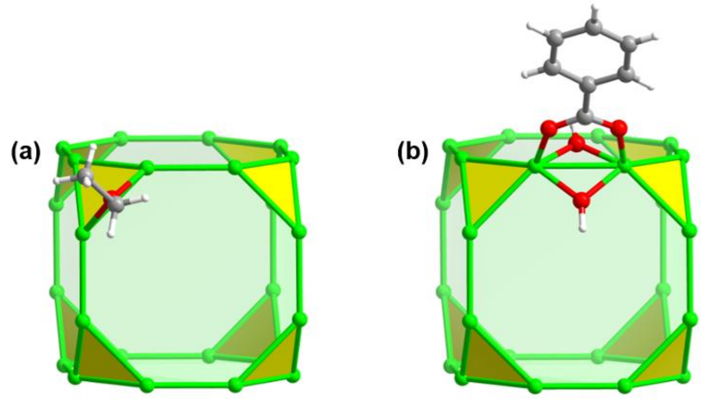

Supplementary Figure 11. The two kinds of edges in the ***tcu*** cage. (a) 24 edges formed by alkoxides; (b) 12 edges formed by benzoates.

The edges in this ***tcu*** cage can be divided into two types, 24 of them are made up of alkoxides and the average distance between Al centers is 2.970 Å (Supplementary Figure 11a). The remaining 12 edges are formed by benzoate and μ_2-OH groups linking adjacent triangular faces, and the average Al–Al distance is 2.854 Å (Supplementary Figure 11b).

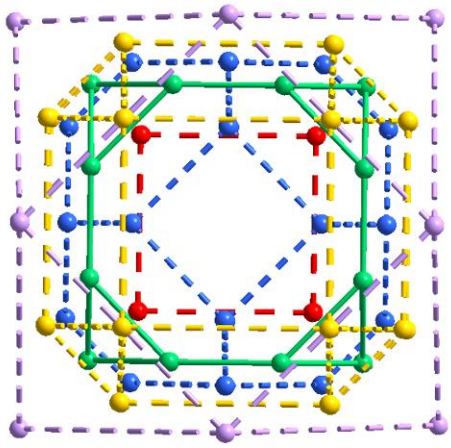

Supplementary Figure 12. The quintuple core-shell organic-inorganic hybrid Russian-doll-like concentric cage in Al_{24}: O_8 ***cube*** @ O_{24} ***tro*** @ Al_{24} ***tcu*** @ $(OR)_{24}$ ***rco*** @ $(BA)_{12}$ ***cuo***.

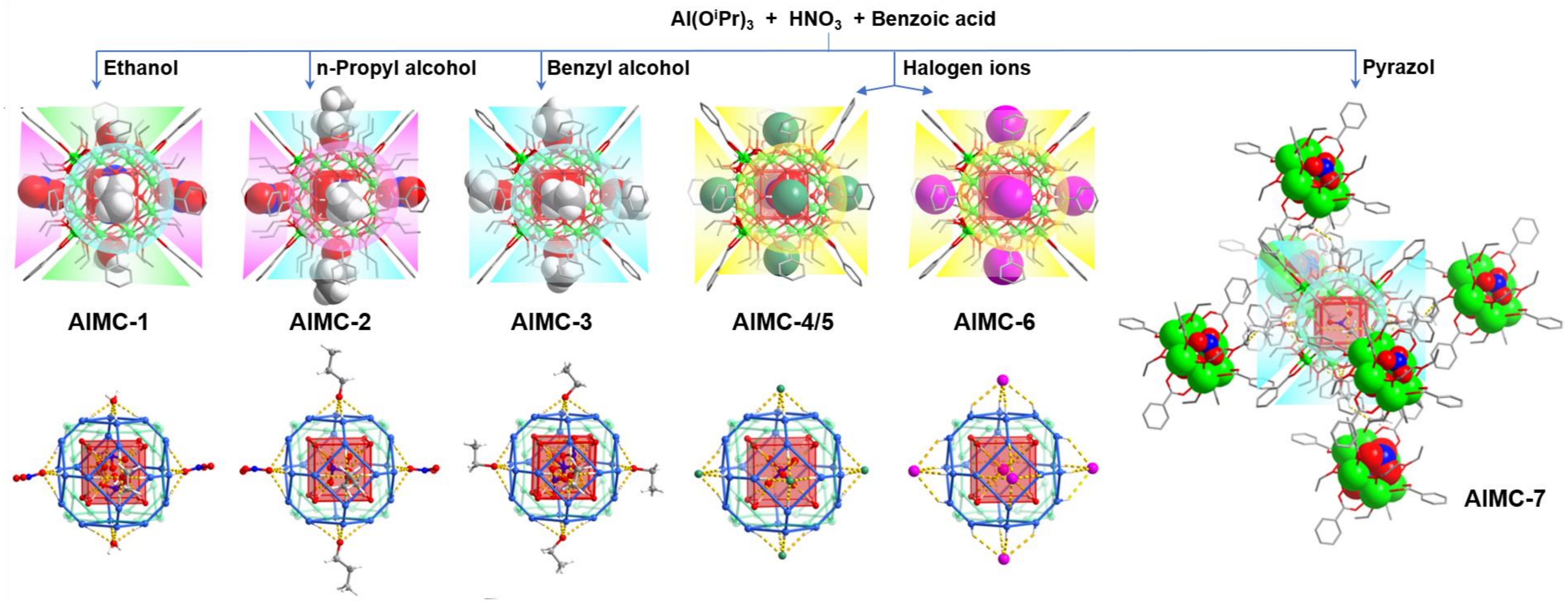

Supplementary Figure 13. Guest inclusion in the Al_{24} ***tcu*** cages and the detailed hydrogen bond interactions. (Al: bright green; O: red; C: grey; H: white; N: blue; Cl: sea-green; I: pink; NO_3^- cavity: pink; water cavity: green; alcohol/alkoxide cavity: blue; halogen ion cavity: yellow)

The +4-charge and the abundant OH groups on the cages provide a golden opportunity to capture a wide range of guests with an assortment of sizes, shapes and charges, this can be applied to generate supramolecular compounds. In this series of compounds, the guests are distributed in two environments. One is in the cavity of the O_8 ***cube***, in which the number of anionic guests is greatly related to the ionic strength of the reaction system. Namely, when quaternary ammonium salts were introduced, the number of anions in the O_8 cube reduces. The others are located on the outside of the octagonal faces of the ***tcu*** cage. Then ions interact with four μ_2-OH groups on O_{24} ***tro*** cage through strong hydrogen bonds, making the original ***tro*** becomes a fake octahedron. Herein, the guests are situated at the six vertices of the octahedron.

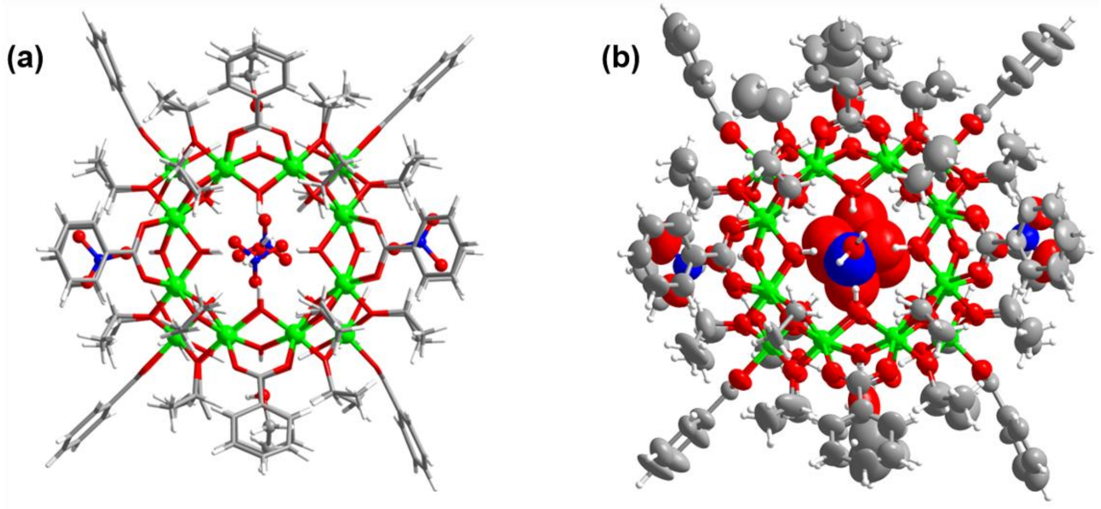

Supplementary Figure 14. The molecular structure of **AlMC-1**. (a) The ball-and-stick view; (b) the ORTEP-style view.

AlMC-1 crystallizes in the monoclinic space group *C*2/c. The cavity of the Al_{24} ***tcu*** cage is filled with two NO_3^- anions, while the six face centers were occupied by three pairs of guests, namely, ethanol molecules, water molecules and nitrate ions (Supplementary Figure 14). In the inner O_8-***cube***, two NO_3^- anions (one resides at the body center and the other is disordered in two symmetrical positions) (Supplementary Figure 39) is trapped within the hydrogen-bonding site by μ_3-OH groups. Each of the oxygen atoms of the NO_3^- anions are associated with 2 to 4 adjacent μ_3-OH groups, thus, there are a total of 18 hydrogen bonds, average distance of O-H···O is 2.92 Å (Supplementary Figure 41). For the other three pairs of guests, all of them are perpendicular to the octagonal faces, generating strong hydrogen bond interactions with the octagonal face through μ_2-OH groups and OEt groups (Supplementary Figure 20). The hydrogen bond lengths range between guests and μ_2-OH groups are respectively, 2.952–3.354 Å for ethanol molecules, 2.864–2.933 Å for water molecules, and 2.896–2.909 Å for nitrate ions. While the hydrogen bond interactions between guest and OEt groups are weaker, ranging from 3.763 to 3.950 Å for ethanol molecules, from 3.453 to 3.768 Å for water molecules, and from 3.245 to 3.979 Å for nitrate ions.

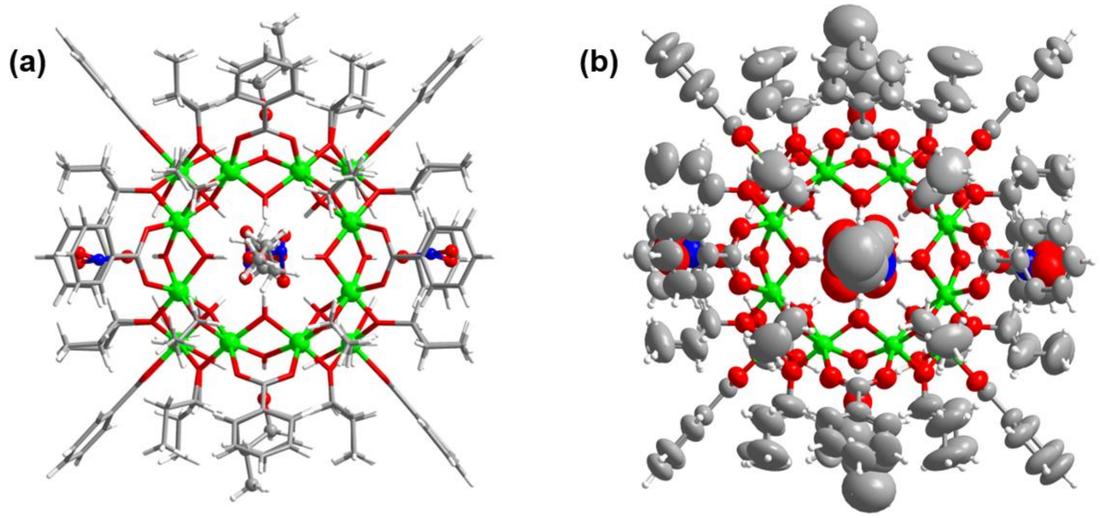

Supplementary Figure 15. The molecular structure of **AlMC-2**. (a) The ball-and-stick view; (b) the ORTEP-style view.

AlMC-2 is isolated by changing the solvent from ethanol to n-propyl alcohol. Single crystal X-ray diffraction (SCXRD) analysis indicates that **AlMC-2** crystallizes in the *P*-1 space group, contains one Al_{24} host, two inner NO_3^- guests, two external NO_3^- ions as well as four n-propyl alcohol molecules (Supplementary Figure 15). Compared with **AlMC-1**, the OEt ligands on the Al_{24} host have been completely replaced by O^nPr groups, indicating that the synthesis of the Al_{24} ***tcu*** cage is universal in an alcohol solvent. The two H_2O guests in **AlMC-2** are also replaced by n-propyl alcohol molecules, leading to the transformation of the crystal system from monoclinic to triclinic. The trapped NO_3^- ions and n-propyl alcohol molecules also interact with the Al_8 macrocyclic units through hydrogen bond interactions (Supplementary Figure 21). The average O-H···O, C-H···O and C-H···C lengths are respectively 2.994 Å, 3.795 Å and 3.639 Å.

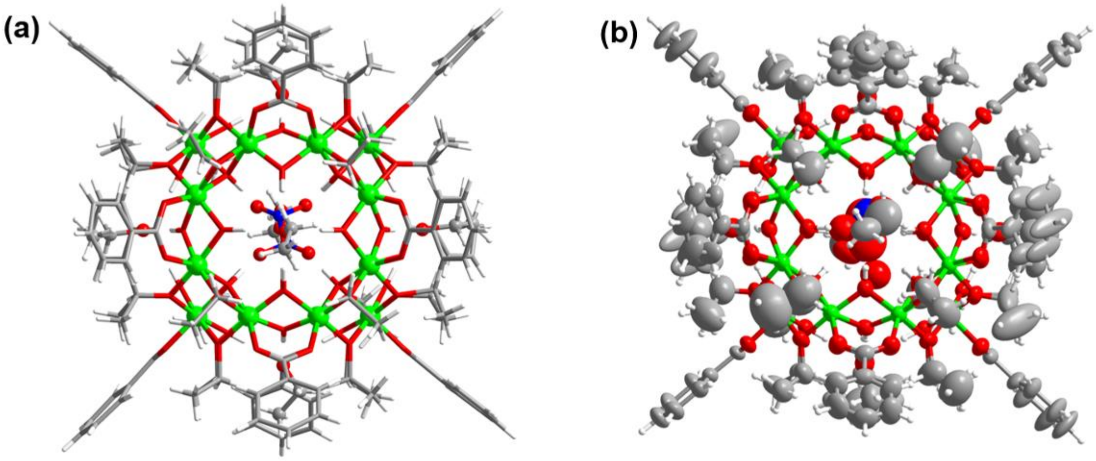

Supplementary Figure 16. The molecular structure of **AlMC-3**. (a) The ball-and-stick view; (b) the ORTEP-style view.

When a small amount of benzyl alcohol was introduced into the synthesis system, **AlMC-3** was isolated. It crystallizes in the space group $P2_1/n$ and consists of one Al_{24} host, two inner NO_3^- ions and three pairs of ethanol guests (Supplementary Figure 16). The inner NO_3^- ions in the Al_{24} cavity are disordered and similar to those in **AlMC-1** and **AlMC-2**. The three pairs of ethanol guests point to the face center of the octagonal faces and are bonded to the Al_8 macrocyclic units through multiple hydrogen bond interactions (Supplementary Figure 22). The lengths of O-H···O, C-H···O and C-H···C bonds are in the range of 2.823–2.938 Å, 3.681–3.898 Å and 3.132–3.982 Å, respectively.

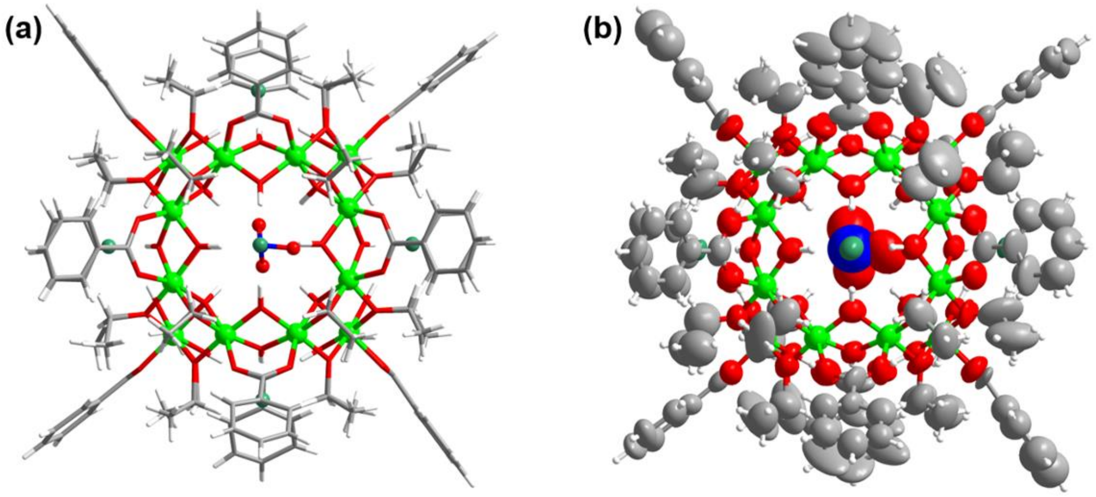

Supplementary Figure 17. The molecular structure of **AlMC-4**. (a) The ball-and-stick view; (b) the ORTEP-style view.

In addition, we speculated that the choice of counter anion could facilitate the generation of isomorphous salts. We selected halogen ions with small hindrance as probe anions and synthesized halogen-compensated compounds $Al_{24}(BA)_{12}(\mu_2\text{-OEt})_{24}(\mu_2\text{-OH})_{24}(\mu_3\text{-OH})_8\cdot NO_3\cdot X_3$ (X=Cl, **AlMC-4**; X=Br, **AlMC-5**) and $Al_{24}(BA)_{12}(\mu_2\text{-OEt})_{24}(\mu_2\text{-OH})_{24}(\mu_3\text{-OH})_8\cdot I_4$ (**AlMC-6**) when introducing quaternary ammonium salts. The results showed that Cl^-, Br^- and I^- guests are located at the six octagonal face centers of the cage with 0.5 occupancies for each position (Supplementary Figures 17–19). **AlMC-4** to **AlMC-6** are respectively crystallized in triclinic space group *P*-1, cubic space group *Ia*-3 and cubic space group *I*m-3m. Notably, the introduction of quaternary ammonium salts greatly improves the ionic strength, thus, there is only one NO_3^- encapsulated in the cage. In **AlMC-4** and **AlMC-5**, the N center on the NO_3^- anion is located at the body center, while the O atoms are respectively disordered in six and twelve positions (Supplementary Figure 40). Each O atom on the inner NO_3^- combines with the neighboring two μ_3-OH groups through strong hydrogen bond interactions (O-H···O: 2.863–3.424 Å) (Supplementary Figure 42). Intriguingly, in **AlMC-6**, the inner cavity of the Al_{24} cage is occupied by an I^- ion rather than NO_3^- anion (Supplementary Figure 40c). This I^- ion interacts with μ_3-OH groups via weak O-H···I interaction, and the distance is 3.935 Å (Supplementary Figure 42c). For face-centered halide ions, there are also obvious O-H···X (X = Cl, Br, I) interactions between the halogen ions and μ_2-OH groups with bond lengths ranging from 3.131 to 3.728 Å (Supplementary Figures 23–25). Halide ions can also interact with OEt groups through $-CH_2$ groups, the average C-H···X (X = Cl, Br, I) interactions are respectively 3.838 Å for **AlMC-4**, 3.750 Å for **AlMC-5** and 3.833 Å for **AlMC-6**.

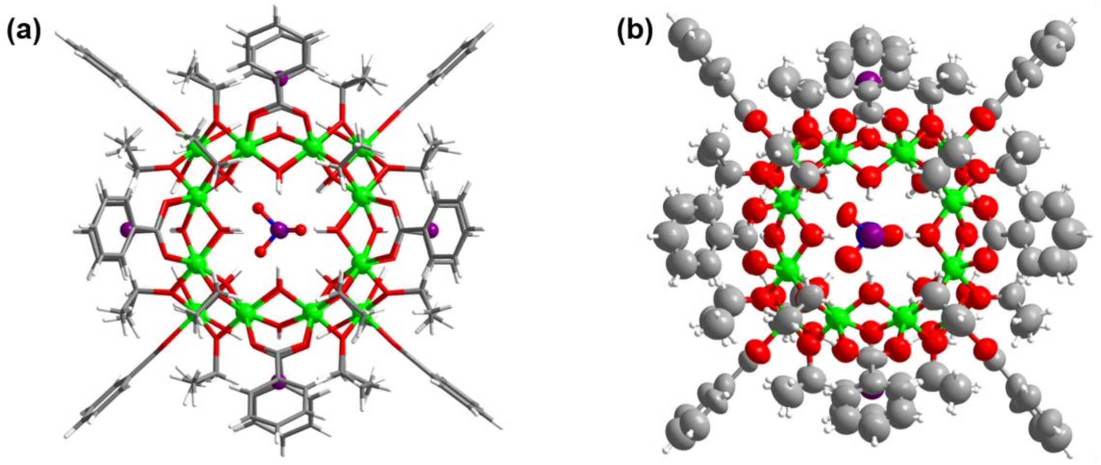

Supplementary Figure 18. The molecular structure of **AlMC-5**. (a) The ball-and-stick view; (b) the ORTEP-style view.

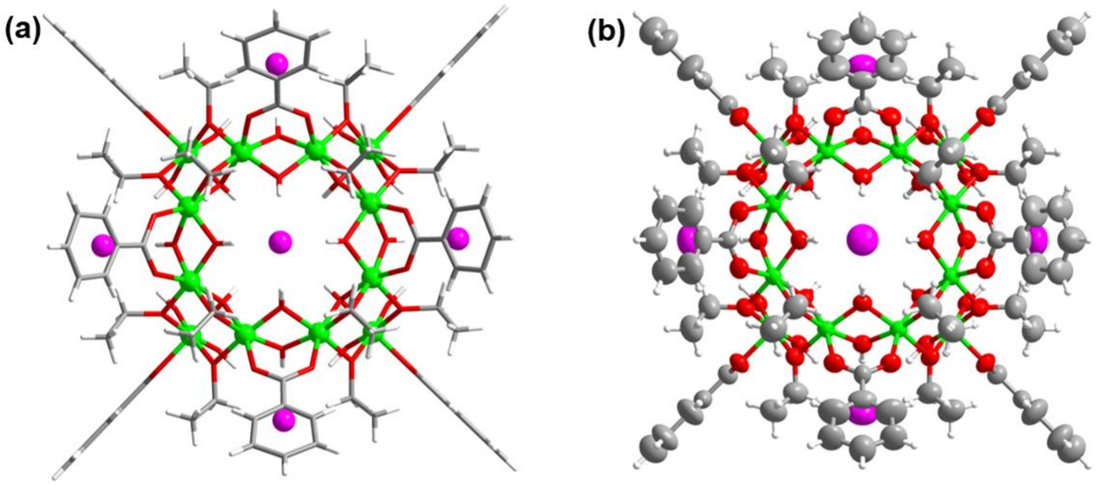

Supplementary Figure 19. The molecular structure of **AlMC-6**. (a) The ball-and-stick view; (b) the ORTEP-style view.

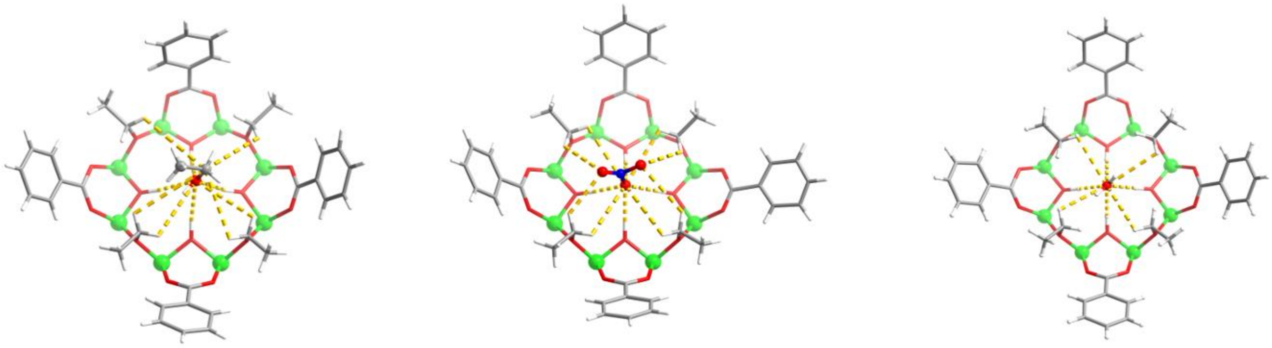

Supplementary Figure 20. The hydrogen bond interactions between guest and Al_8 macrocycle in **AlMC-1**.

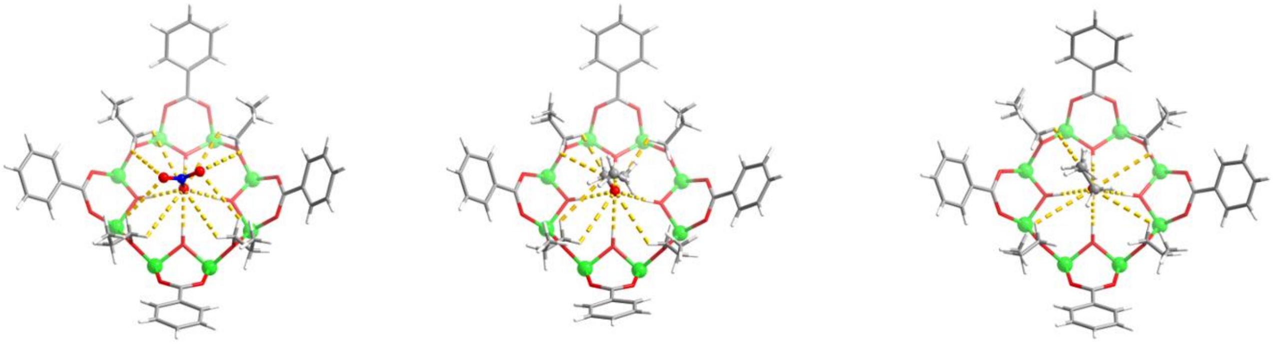

Supplementary Figure 21. The hydrogen bond interactions between guest and Al_8 macrocycle in **AlMC-2**.

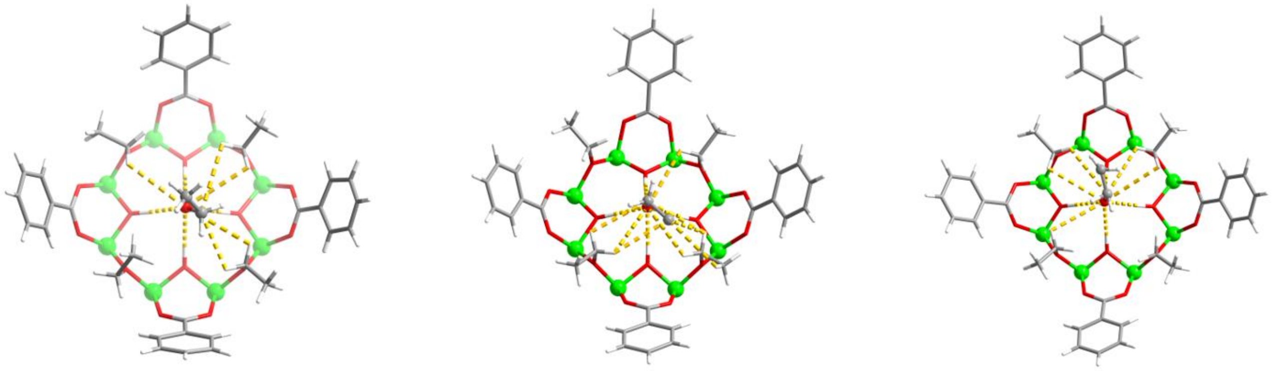

Supplementary Figure 22. The hydrogen bond interactions between guest and Al_8 macrocycle in **AlMC-3**.

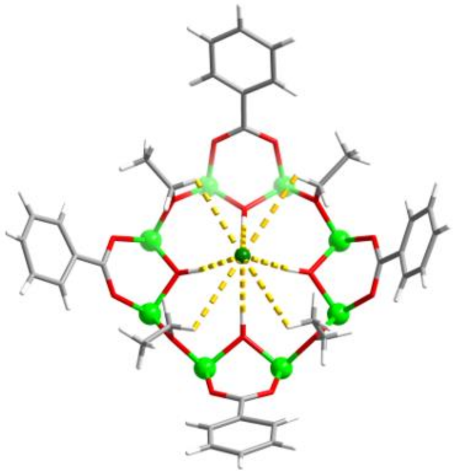

Supplementary Figure 23. The hydrogen bond interactions between guest and Al_8 macrocycle in **AlMC-4**.

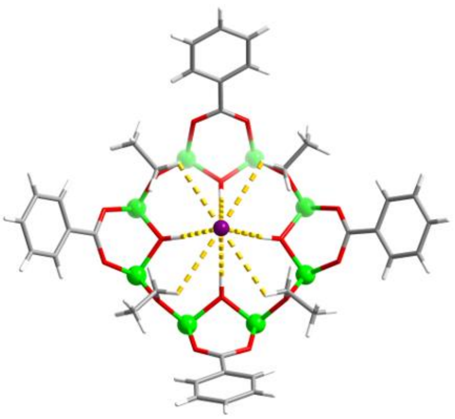

Supplementary Figure 24. The hydrogen bond interactions between guest and Al_8 macrocycle in **AlMC-5**.

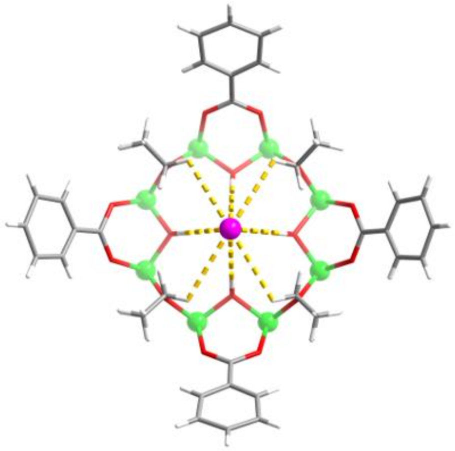

Supplementary Figure 25. The hydrogen bond interactions between guest and Al_8 macrocycle in **AlMC-6**.

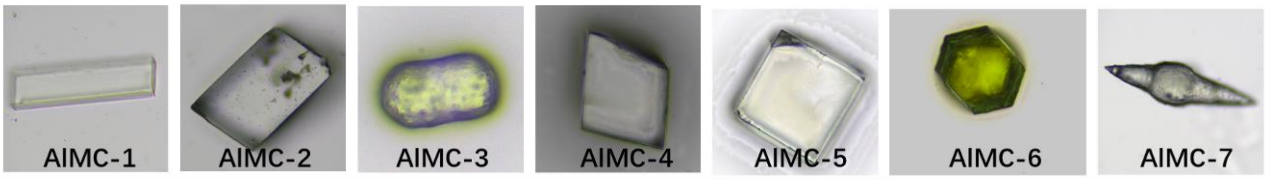

Supplementary Figure 26. Crystal photos of **AlMC-1** to **AlMC-7**.

Except for **AlMC-1**, other crystals are extremely susceptible to mechanical degradation when exposed to air. Once removed from the mother fluid, a large number of cracks occur on the crystal surfaces. Thus, the crystals were immediately soaked in oil before taking pictures to preserve their transparency. Obviously, highly symmetrical space groups lead to the generation of crystals with highly symmetrical shapes, especially for **AlMC-5** and **AlMC-6**.

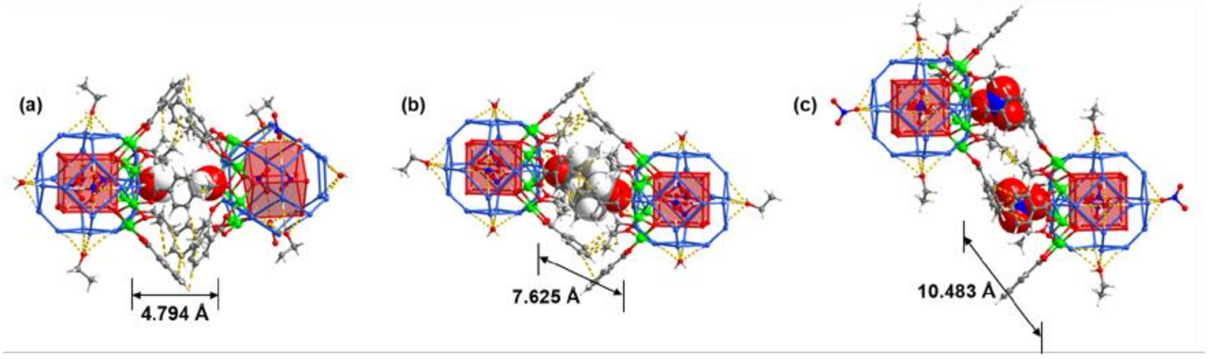

Supplementary Figure 27. The hydrogen bonding cavity in **AlMC-1**. (a) H_2O cavity; (b) ethanol cavity; (c) NO_3^- cavity. The size of the cavity is calculated based on the distance of two Al_8 macrocycles' bottom centers.

Two adjacent Al_{24} cages can form a cavity by using their opposite Al_8 macrocycles, and further encapsulate a pair of guests. In **AlMC-1**, there are three types of guest cavities, whose sizes are respectively 4.794 Å, 7.625 Å and 10.483 Å. Due to the smaller steric hindrance, water molecules trapped in the Al_8 macrocycles can be assembled face-to-face (Supplementary Figure 27a), so the cavity size is the smallest. In this cavity, the ethanol ligands in one Al_8 macrocycle form strong hydrogen bonds with the benzoate ligands on the other macrocycle, and the average C-H···C bond length is 3.871 Å. For the ethanol and NO_3^- cavities (Supplementary Figures 27b and 27c), the two Al_8 macrocycles are misaligned, and the average hydrogen bond lengths between the benzene ring ligands and ethanol ligands are respectively 3.786 Å and 3.820 Å.

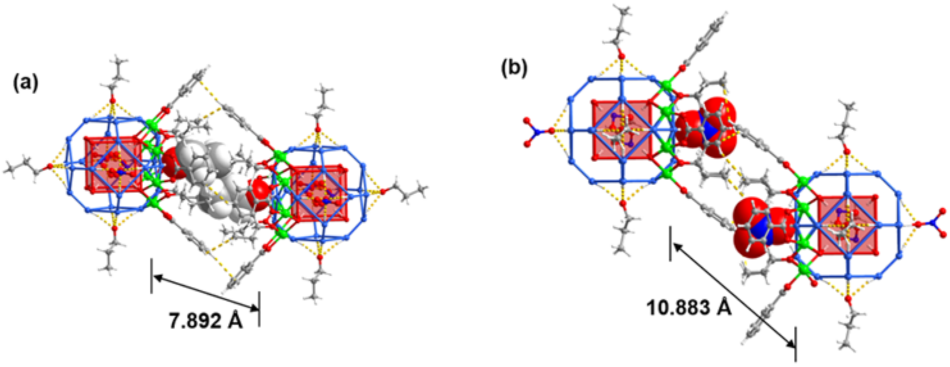

Supplementary Figure 28. The hydrogen bonding cavities in **AlMC-2**. (a) n-propanol cavity; (b) NO_3^- cavity. The size of the cavity is calculated based on the distance of two Al_8 macrocycles' bottom centers.

In **AlMC-2**, there are two kinds of cavities, one is filled by n-propanol, and the other by NO_3^- ions. In the n-propanol cavity, the hydrogen bond interactions between two the Al_8 macrocycles are generated by coordinated n-propanol and benzoate ligands. The average C-H···C length is 3.758 Å. For the NO_3^- cavity, there are three pairs of hydrogen bond interactions, two of them are between coordinated n-propanol and benzoate, and the bond lengths are 3.959 Å and 3.953 Å. The other is between two coordinated benzoate ligands with a bond length of 3.968 Å.

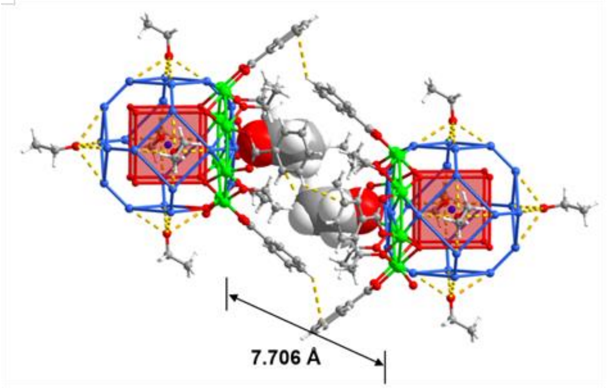

Supplementary Figure 29. The hydrogen bond cavity in **AlMC-3**. The size of the cavity is calculated based on the distance of two Al_8 macrocycles' bottom centers.

In **AlMC-3**, there is only one kind of ethanol cavity, and the distance between two centers of octagonal faces is 7.706 Å. In this cavity, there are two pairs of hydrogen bonds between neighboring benzoate ligands, and the distances are respectively 3.551 Å and 3.915 Å.

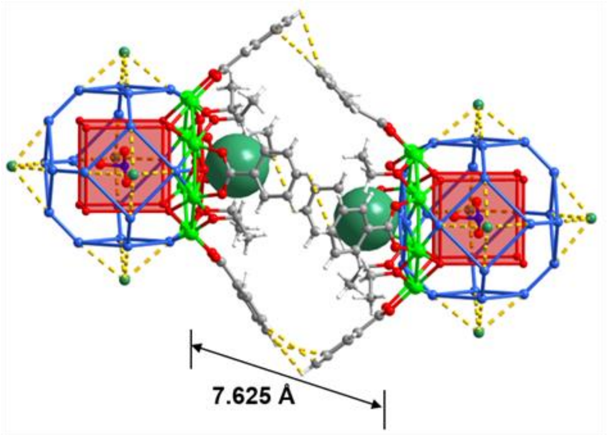

Supplementary Figure 30. The hydrogen bond cavity in **AlMC-4**. The size of the cavity is calculated based on the distance of two Al_8 macrocycles' bottom centers.

The size of Cl^- ion cavity in **AlMC-4** is close to that of the ethanol cavity. It is also involved in numerous hydrogen bonding interactions with the length ranging 3.645–4.035 Å.

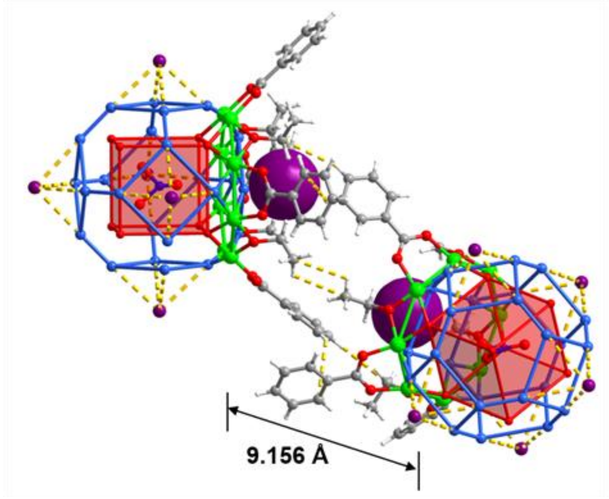

Supplementary Figure 31. The hydrogen bond cavity in **AlMC-5**. The size of the cavity is calculated based on the distance of two Al_8 macrocycles' bottom centers.

Due to the two opposite octagonal surfaces are not parallel, the Br^- ions cavity in **AlMC-5** is semi-open. Thus, the hydrogen bonds only focus on one side of the cavity. The three pairs of hydrogen bonds are respectively located between benzoate and benzoate (3.986 Å), ethylate and ethylate (3.360 Å), as well as benzoate and ethylate (3.980 Å).

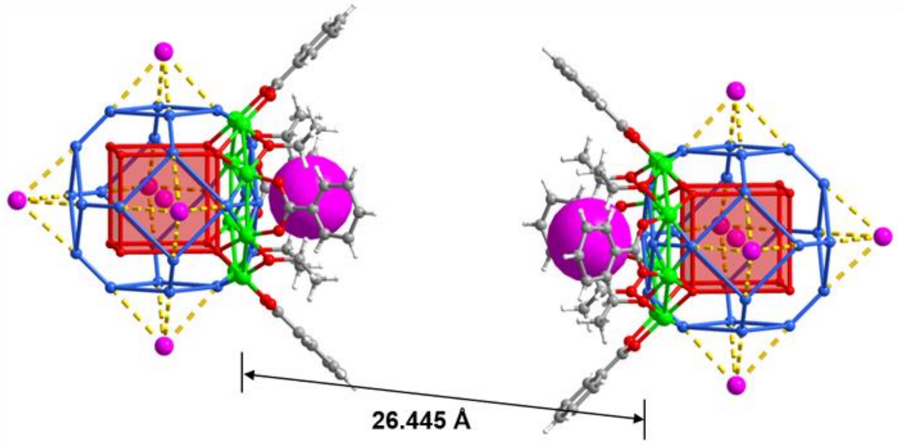

Supplementary Figure 32. No hydrogen bond cavity in **AlMC-6**. The size is calculated based on the distance of two Al_8 macrocycles' bottom centers.

Due to the porous accumulation mode, the distance between adjacent Al_{24} cage in **AlMC-6** is large, thus, there no hydrogen bonding cavity can be found in **AlMC-6**. The cavity and pore surface structures of **AlMC-1** to **AlMC-8** have been simulated on Mercury (yellow / dark brown curved surface) (Supplementary Figures 33–38, 49 and 53). Yellow represents the outer surface of the cavity or pore, while dark brown is the internal surface of the cavity or pore. The probe radius and approx. grid spacings are 1.2 and 0.7 Å, respectively. In order to present a clear cavity structure, some guests are omitted. Al_{24} cages stack up to create a 1D pore in **AlMC-1**, 3D pores in **AlMC-5** and **AlMC-6**, which have been highlighted by blue square frames. The are no pores in the other compounds. The isolated H_2O cavity, NO_3^- cavity, alcohol cavity, n-propanol cavities, Cl^- cavity and Br^- cavity can also be observed in **AlMC-1**, **AlMC-2**, **AlMC-3**, **AlMC-4** and **AlMC-5**, that are highlighted by red circles. Interestingly, some of them present various shapes based on the packing modes and guest types. For example, the H_2O cavity in **AlMC-1** is a spindle shape (Supplementary Figure 33a), the NO_3^- cavity in **AlMC-1** a peanut shape (Supplementary Figure 33b), the alcohol cavity in **AlMC-1** is a Z-type (Supplementary Figure 33c), while the Cl^- ion cavity in **AlMC-4** is a saucer shape (Supplementary Figure 36b).

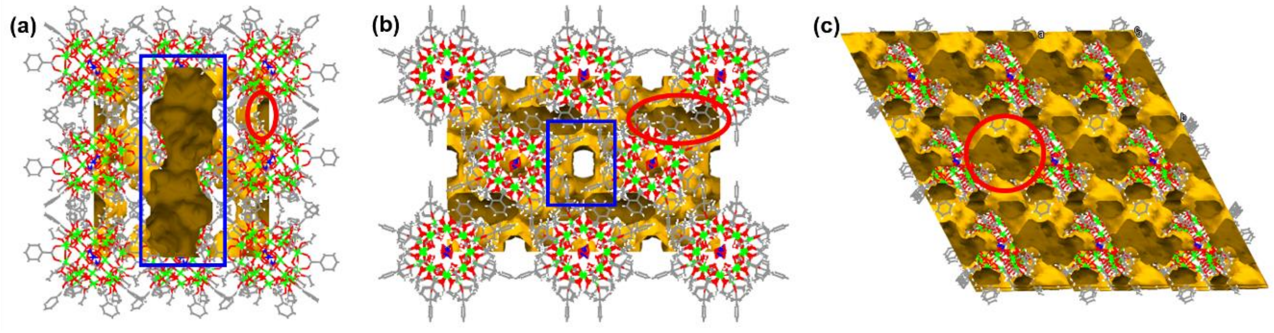

Supplementary Figure 33. Packing in **AlMC-1** along the crystallographic axis. (a) a-axis; (b) c-axis; (c) b-axis. The 1D pore has been highlighted by the blue square frame. The spindle-shaped H_2O cavity (a), peanut-shape NO_3^- cavity (b) and Z-shape alcohol cavity (c) in **AlMC-1** have been highlighted by red circles.

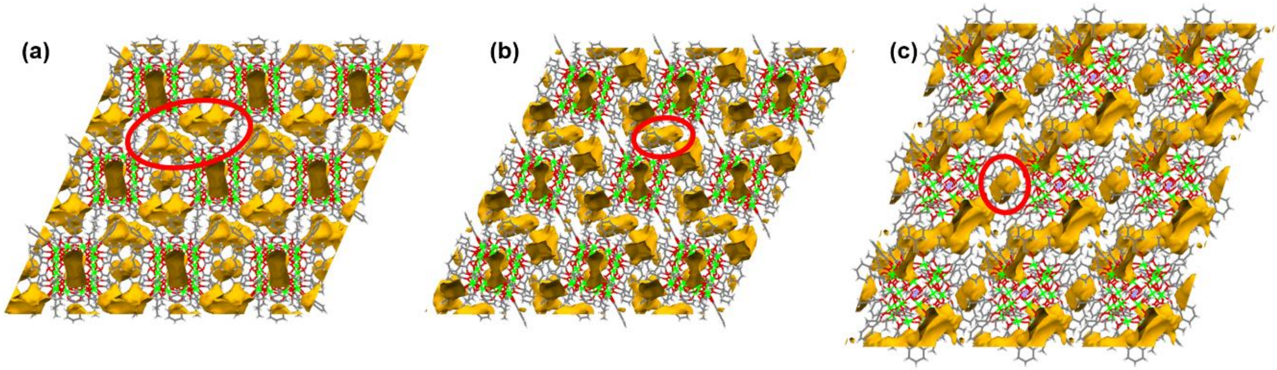

Supplementary Figure 34. Packing of **AlMC-2** along the crystallographic axis. (a) a-axis; (b) b-axis; (c) c-axis. The simulated NO_3^- cavity (a), n-propanol cavities (b and c) in **AlMC-2** have been highlighted by red circles.

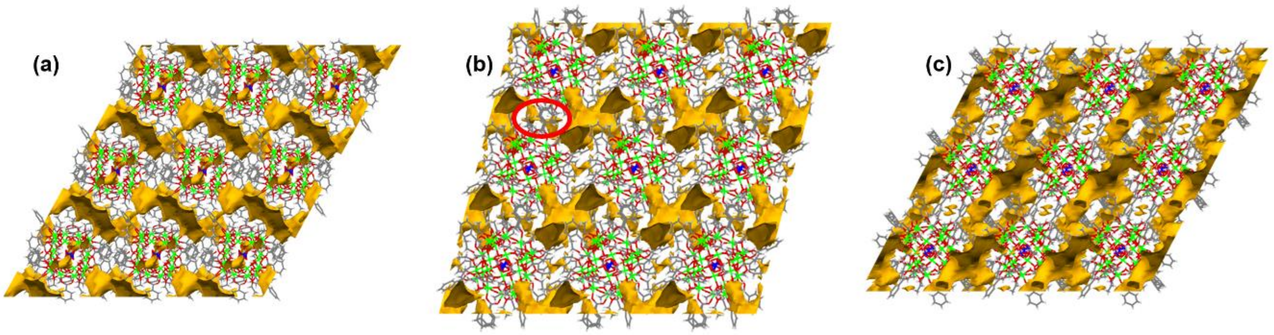

Supplementary Figure 35. Packing of **AlMC-3** along the crystallographic axis. (a) a-axis; (b) b-axis; (c) c-axis. The simulated alcohol cavity (b) in **AlMC-3** has been highlighted by a red circle.

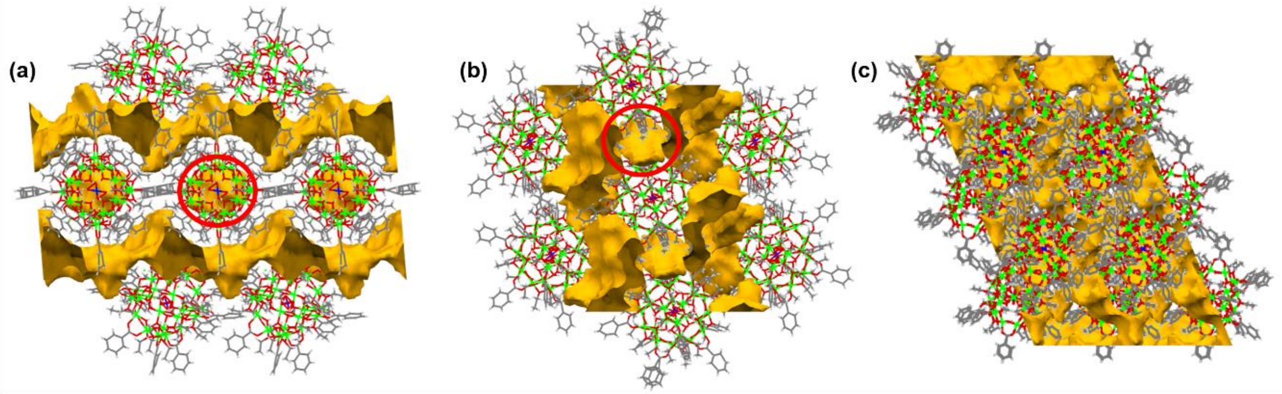

Supplementary Figure 36. Packing of **AlMC-4** along the crystallographic axis. (a) a-axis; (b) b-axis; (c) c-axis. The simulated flying saucer-shaped Cl^- cavity (a and b) in **AlMC-4** has been highlighted by red circles.

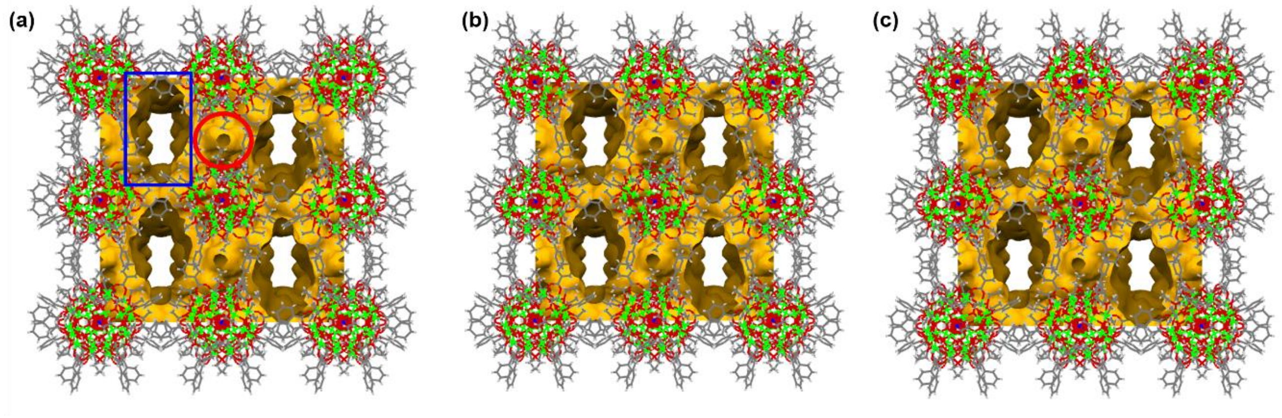

Supplementary Figure 37. Packing of **AlMC-5** along the crystallographic axis. (a) a-axis; (b) b-axis; (c) c-axis. The simulated 3D pore has been highlighted by a blue square frame. The simulated Br^- cavity in **AlMC-5** has been highlighted by a red circle.

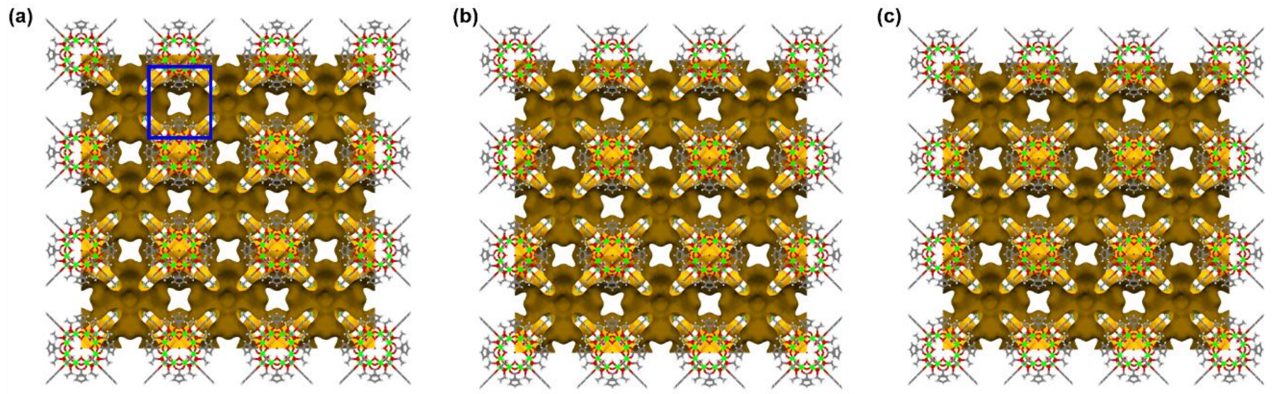

Supplementary Figure 38. Packing of **AlMC-6** along the crystallographic axis. (a) a-axis; (b) b-axis; (c) c-axis. The simulated 3D pore has been highlighted by a blue square frame.

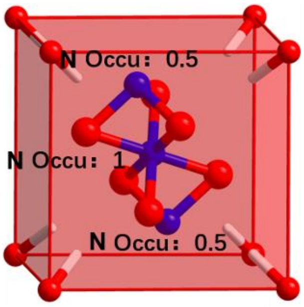

Supplementary Figure 39. The disordered NO_3^- with N atoms in different occupancies (Occu) in **AlMC-1**–**AlMC-3**.

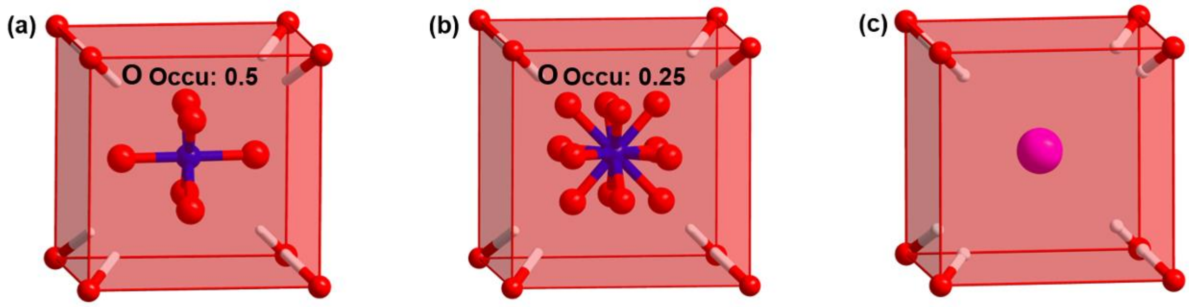

Supplementary Figure 40. The guest in O_8 ***cube*** for **AlMC-4**–**AlMC-6**. The disordered NO_3^- ion in the O_8 ***cube*** of **AlMC-4** (O atoms occupancies: 0.5) (a); the disordered NO_3^- ion in the O_8-cube of **AlMC-5** (O atoms occupancies: 0.25); the I ion in the O_8-cube of **AlMC-6** (c).

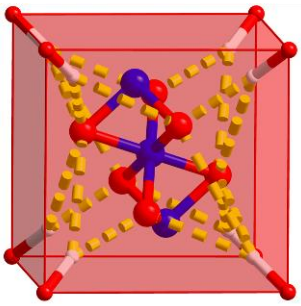

Supplementary Figure 41. The hydrogen-bond interactions between disordered NO_3^- and μ$_3$-OH groups in **AlMC-1**–**AlMC-3**.

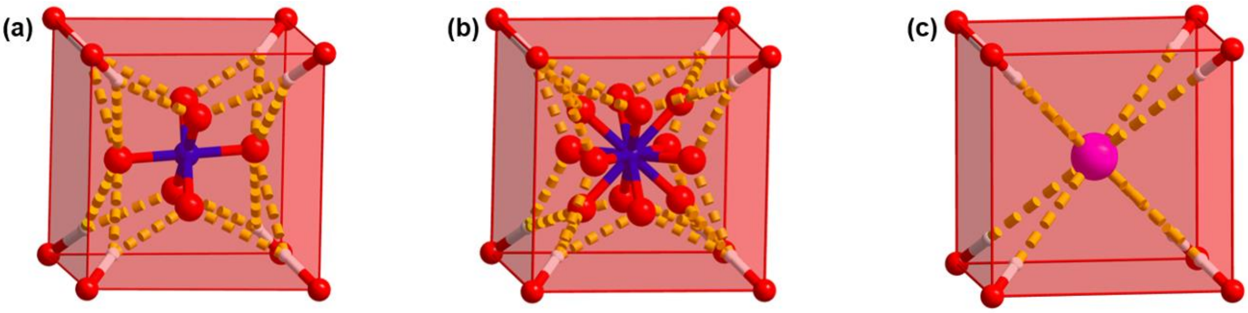

Supplementary Figure 42. The hydrogen-bond interactions between anions and μ$_3$-OH groups in **AlMC-4**–**AlMC-6**. (a) The hydrogen-bond interactions between NO_3^- and μ$_3$-OH groups in **AlMC-4**; (b) the hydrogen-bond interactions between NO_3^- and μ$_3$-OH groups in **AlMC-5**; (c) the hydrogen-bond interactions between I^- and μ$_3$-OH groups in **AlMC-6**.

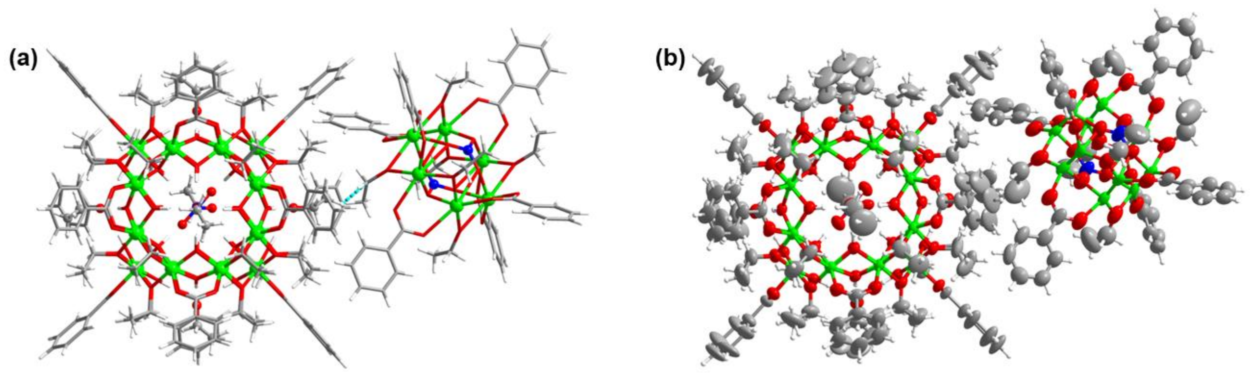

Supplementary Figure 43. The molecular structure of **AlMC-7**. (a) The ball-and-stick view; (b) the ORTEP-style view.

The introduction of an organic base has a great effect on the generation of the Archimedian solid, and the Al_{24} cage can also encapsulate an unprecedented aluminum molecular ring during crystallization. Compound $[Al_{24}(BA)_{12}(\mu_2\text{-}OEt)_{24}(\mu_2\text{-}OH)_{24}(\mu_3\text{-}OH)_8]\cdot(HNO_3)\cdot(OEt)_6\cdot[(Al_6(BA)_6(OEt)_6(NO_3)_2)_{0.5}]$ (**AlMC-7**) (Supplementary Figure 43a) crystallizes in the trigonal *R*-3 space group, comprising of two positively charged nanoclusters (the $\{Al_{24}\}^{4+}$ cage and the $[(Al_6(BA)_6(OEt)_6(NO_3)_2)_{0.5}]^{2+}$ ($\{Al_6\}_{0.5}$) ring) (Supplementary Figure 44), six ethylate guests and one inner HNO_3. This is the first time that such an aluminum ring unit has been co-crystallized in this way. Six ethylate guests are embedded in six Al_8 macrocycles through multiple O-H···O (range: 2.907–2.971 Å), O-H···C (range: 3.675–3.992 Å), and C-H···C (range: 3.776–3.836 Å) interactions and point to the centers of octagonal surfaces. On the outside of the Al_{24} cage, each of the Al_8 macrocycles further captures $\{Al_6\}_{0.5}$ rings through C-H···C interactions (range: 3.383–3.845 Å) and C-H···π interactions (range: 3.797–3.882 Å) (Supplementary Figure 45). Thus, each Al_{24} cage in **AlMC-7** is surrounded by six $\{Al_6\}_{0.5}$ rings (Supplementary Figure 46), and each $\{Al_6\}_{0.5}$ ring is also enwrapped by six Al_{24} cages (Supplementary Figures 47 and 48). The weak interactions between $\{Al_6\}_{0.5}$ ring and the octahedral hydrogen bond cage have been highlighted in Supplementary Figure 47, while the hydrogen bond interactions between neighboring the Al_{24} cages have been also provided in Supplementary Figure 48.

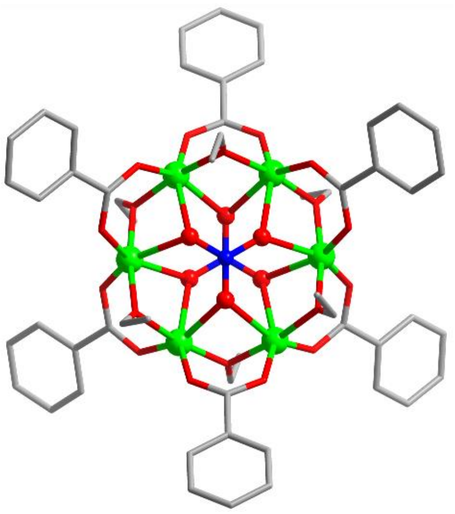

Supplementary Figure 44. The molecular structure of $[(Al_6(BA)_6(OEt)_6(NO_3)_2)_{0.5}]^{2+}$.

The cationic $\{Al_6\}$ ring in **AlMC-7** consists of six Al^{3+} ions, six coordinated benzoates, six coordinated alkoxides as well as two NO_3^- anions (Supplementary Figure 44). Benzoates and alkoxides on $\{Al_6\}$ ring are distributed on the outside of the ring. While two NO_3^- anions are located above and below the center of the ring, and further coordinated with Al atoms. Although a large number of aluminum molecular rings have been reported,[28-29, 15] the inner surfaces of these are all coordinated by hydroxyl groups or alcohols. The presence of NO_3^- anions in such a ring unit is the first report.

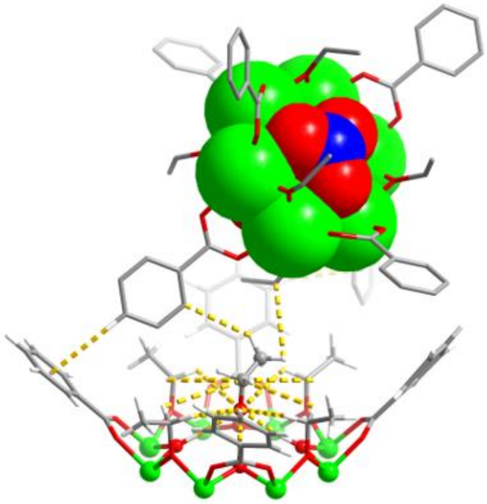

Supplementary Figure 45. The hydrogen bond interactions and C-H···π interactions between Al_8 macrocycles, ethylate guest and Al_6 rings in **AlMC-7**.

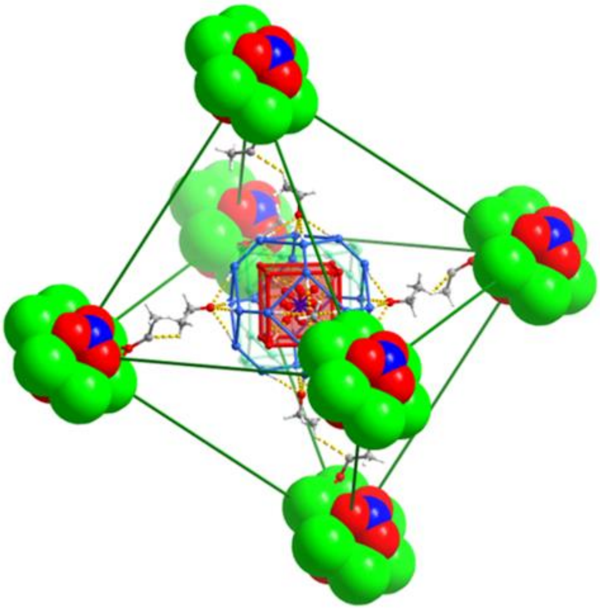

Supplementary Figure 46. The dual-Platonic octahedra in **AlMC-7**, one is formed by the face-centered OEt^- anions and the other is constructed by Al_6 rings.

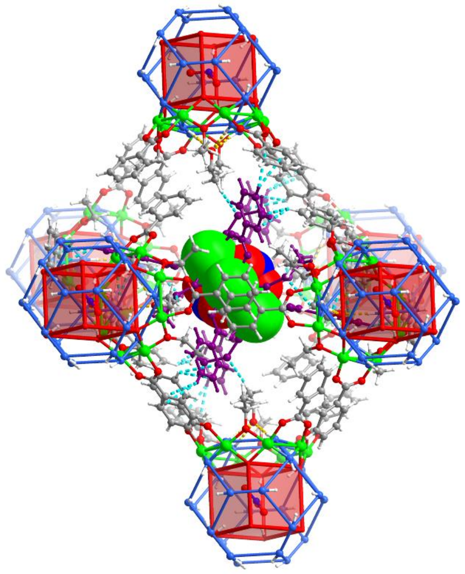

Supplementary Figure 47. The hydrogen bond interactions between Al_{24} cages and Al_6 rings in the octahedral hydrogen bonding cage of **AlMC-7**. The benzoates and alkoxides on the Al_6 ring are highlighted in dark purple.

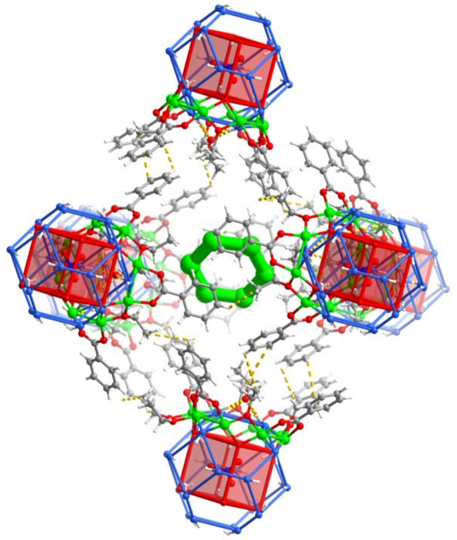

Supplementary Figure 48. The hydrogen bond interactions between neighboring Al_{24} cages in the octahedral hydrogen bonding cage of **AlMC-7**. The benzoates and alkoxides on Al_6 ring have been omitted for clarity.

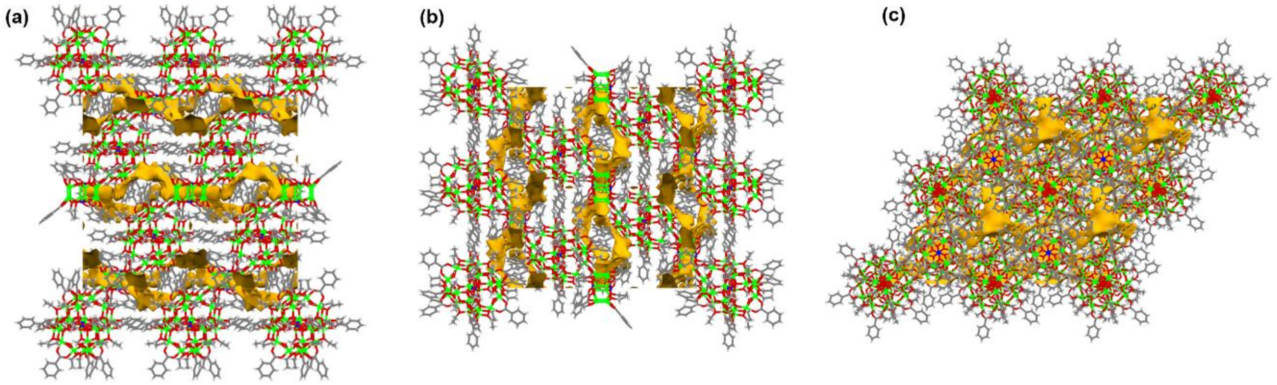

Supplementary Figure 49. Packing of **AlMC-7** along the crystallographic axis. (a) a-axis; (b) b-axis; (c) c-axis. Due to the dense packing, there are no cavities or pores.

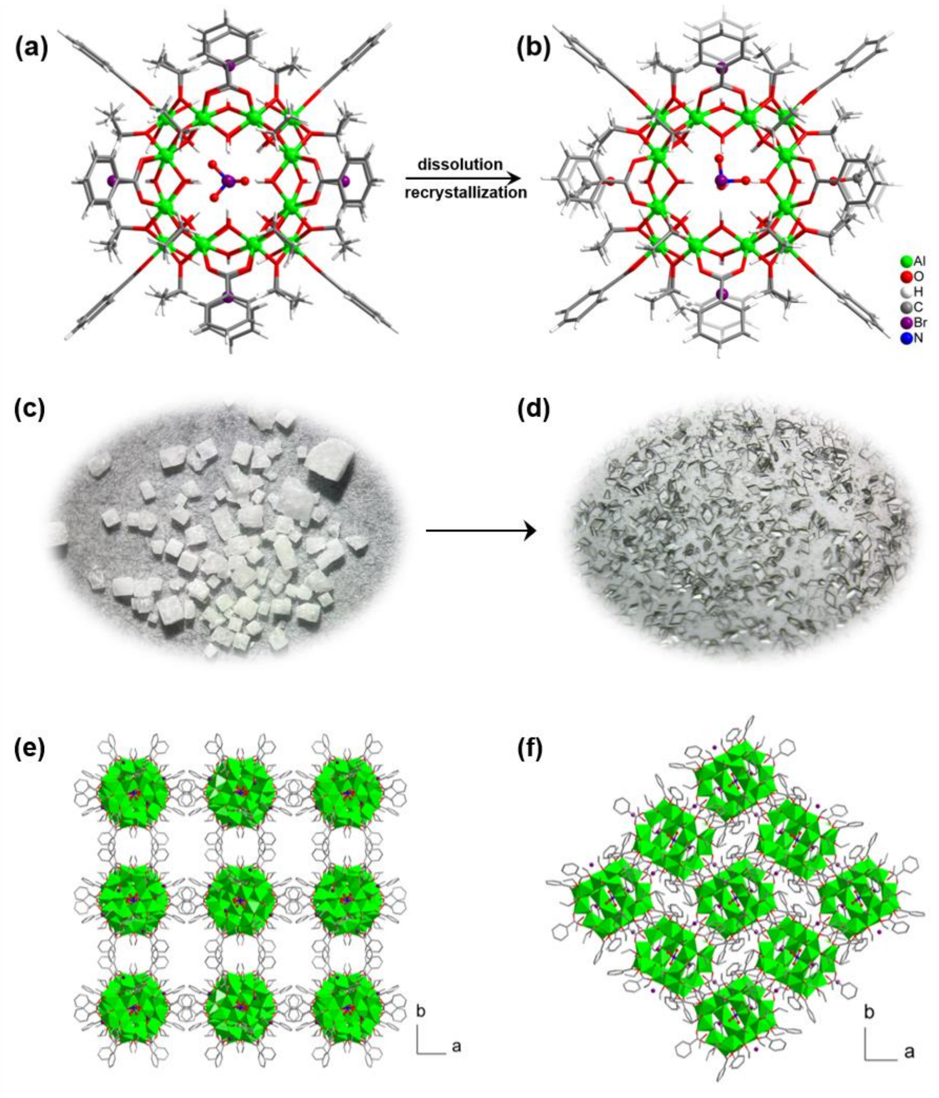

Supplementary Figure 50. The structural conversion from **AlMC-5** to **AlMC-8**. (a) The molecular structure of **AlMC-5**; (b) the molecular structure of **AlMC-8** recrystallized from **AlMC-5**; (c) photograph of **AlMC-5**; (d) photograph of **AlMC-8**; (e) the packing of **AlMC-5**; (f) the packing of **AlMC-8**.

AlMC-8 was obtained by recrystallization of **AlMC-5** from MeCN (Supplementary Figure 50a). It crystallizes in the monoclinic $P2_1/n$ space group and consists of one Al_{24} cage, one inner NO_3^- guest, two Br^- ion guests and one OEt^- guest (Supplementary Figure 50b). Notably, the Br^- ion and OEt^- ion guests are disorder within the six Al_8 macrocyclic units, and the occupancy for each position is 0.5. These guests are fixed by multiple hydrogen bond interactions from coordinated hydroxyl groups, ethylates and benzoates, including O-H···O (range: 2.908–3.507 Å), O-H···Br (range: 3.183–3.329 Å), C-H···Br (range: 3.621–3.894 Å), C-H···C (range: 3.699–3.840 Å) and C-H···O (range: 3.674–3.823 Å) bonds (Supplementary Figure 51). Compared with **AlMC-5**, the Al_{24} skeleton and the body-centered NO_3^- guest is still maintained after recrystallization. But a pair of face-centered disorder Br^- ions are substituted by OEt^- groups. These OEt^- guests may be derived from the partial redistribution of coordination ethylates on dissolved the Al_{24} cage, which can be demonstrated by ESI-MS. Despite the high yield of **AlMC-5**, the crystals are not very pure and easily degenerate in the air (Supplementary Figure 50c). However, we can obtain pure-phase crystals of **AlMC-8** after recrystallization (Supplementary Figure 50d), which have a parallelogram shape and remain bright after exposure to the air for one month. Moreover, the packing of **AlMC-5** and **AlMC-8** is also different, one is porous (Supplementary Figure 50e), while the other is compact (Supplementary Figure 50f).

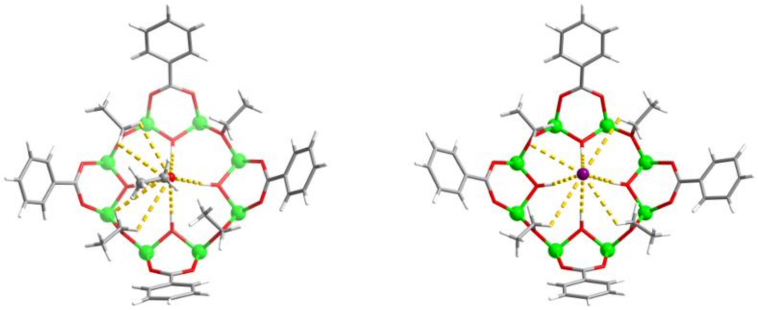

Supplementary Figure 51. The hydrogen bond interactions between guests and the Al_8 macrocyclic units in **AlMC-8**.

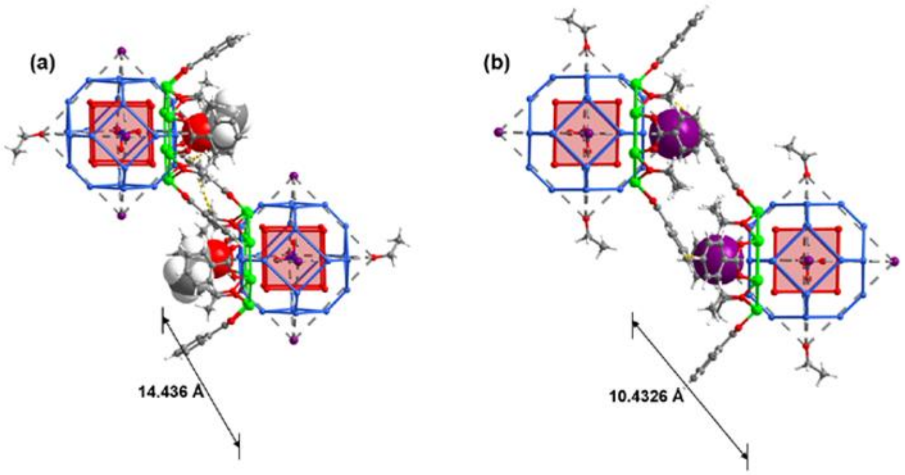

Supplementary Figure 52. No hydrogen bond cavity in **AlMC-8**. (a) The hydrogen bonded interactions between two OEt^- cavities; (b) The hydrogen bonded interactions between two Br^- cavities.

The distance between the two Al_8 macrocyclic units in neighboring molecules in **AlMC-8** is too great to form a closed hydrogen bond cavity despite the presence of week hydrogen bonding interactions between two macrocycles (C–H···C range: 3.661–3.993 Å) (Supplementary Figure 52).

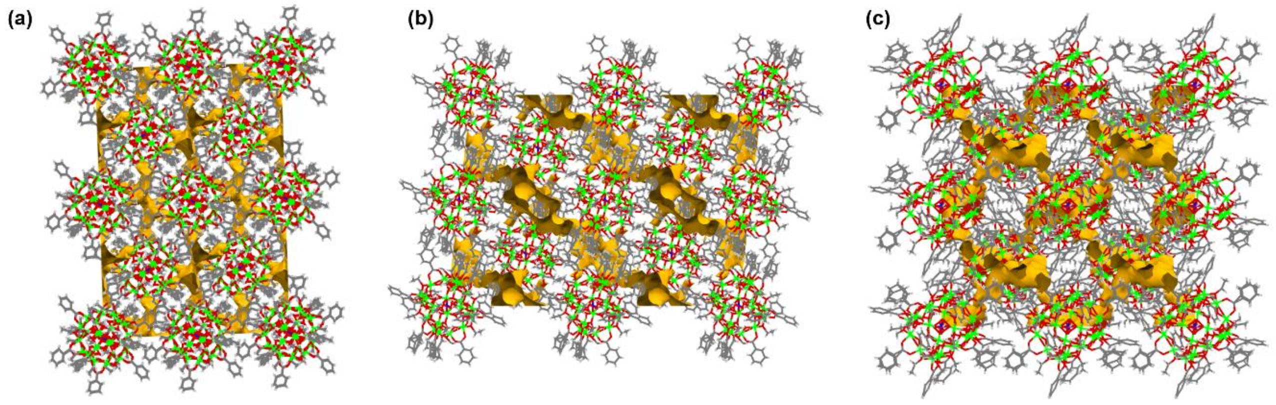

Supplementary Figure 53. Packing of **AlMC-8** along the crystallographic axis. (a) a-axis; (b) b-axis; (c) c-axis. Due to the dense packing, there are no cavities or pores.

3.3 PXRD analysis for AlMC-1 to AlMC-8

The experimental PXRD patterns for **AlMC-1** to **AlMC-8** are consistent with the simulated ones from SCXRD, which indicates that the samples are pure (Supplementary Figures 54–61). The differences in intensity between the experimental and simulated patterns might be due to the variation in crystal orientation for power samples.

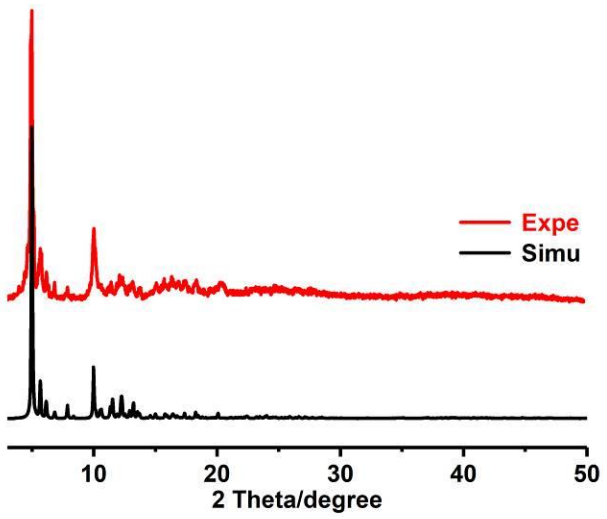

Supplementary Figure 54. The PXRD of the simulated and experimental patterns of **AlMC-1**.

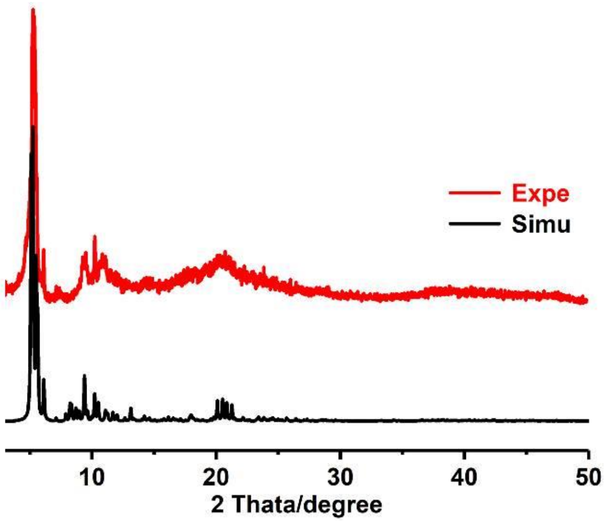

Supplementary Figure 55. The PXRD of the simulated and experimental patterns of **AlMC-2**.

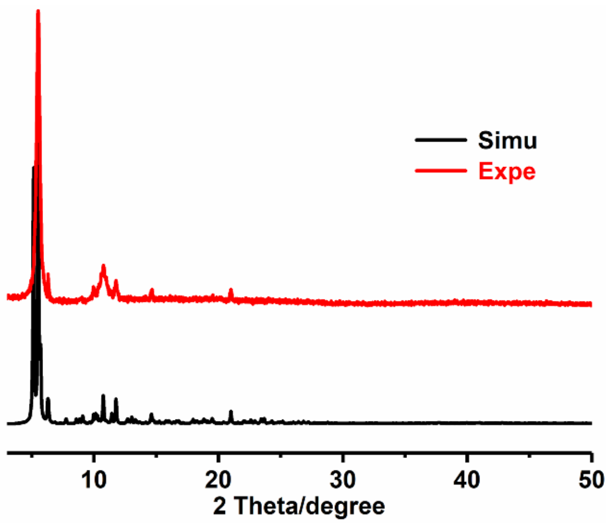

Supplementary Figure 56. The PXRD of the simulated and experimental patterns of **AlMC-3**.

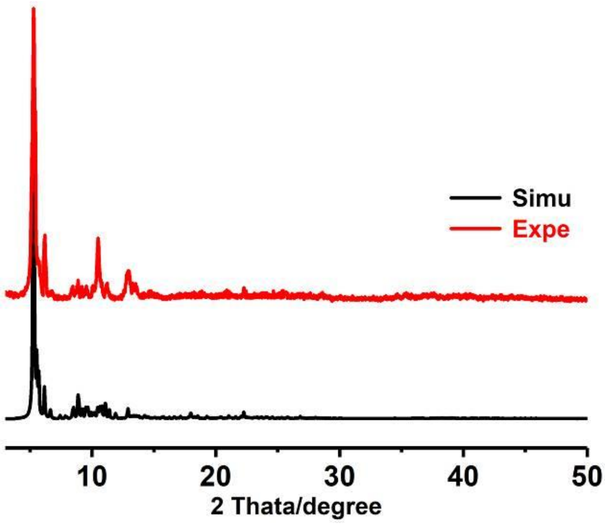

Supplementary Figure 57. The PXRD of the simulated and experimental patterns of **AlMC-4**.

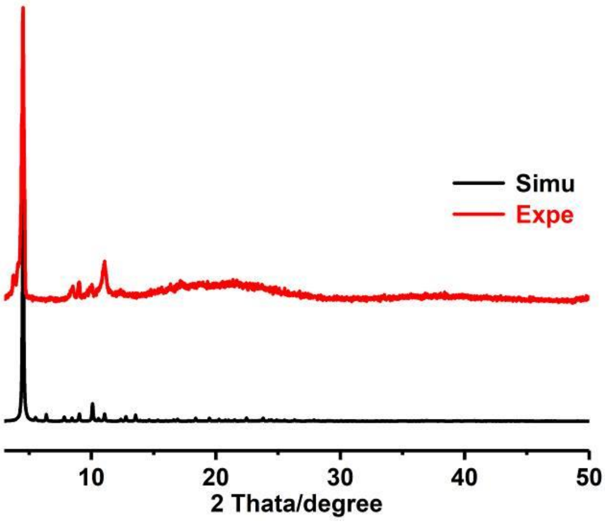

Supplementary Figure 58. The PXRD of the simulated and experimental patterns of **AlMC-5**.

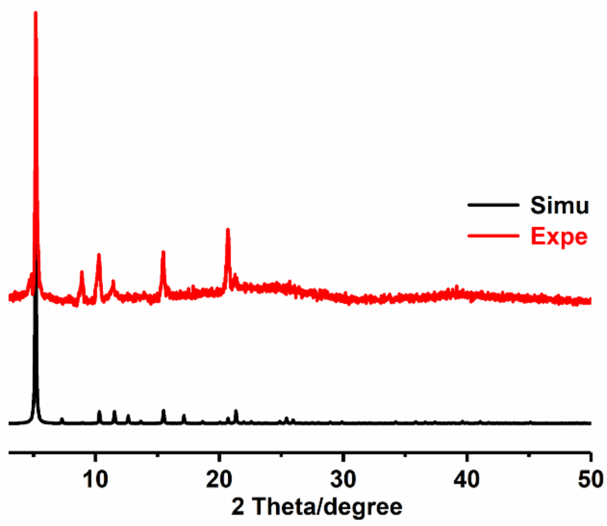

Supplementary Figure 59. The PXRD of the simulated and experimental patterns of **AlMC-6**.

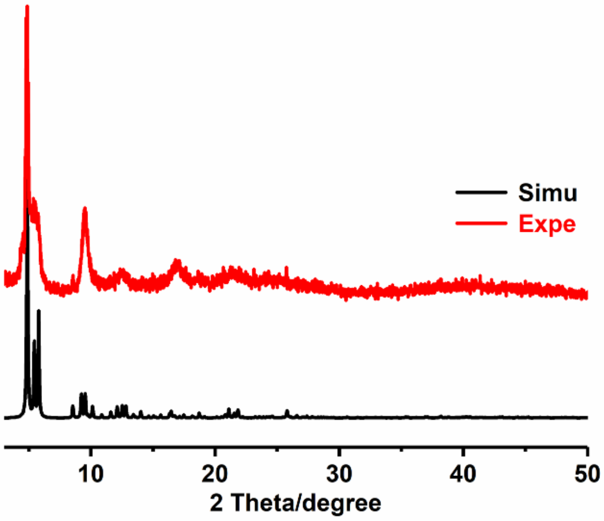

Supplementary Figure 60. The PXRD of the simulated and experimental patterns of **AlMC-7**.

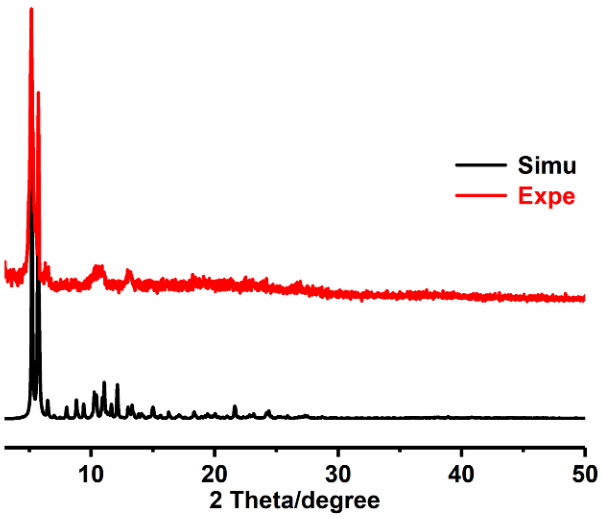

Supplementary Figure 61. The PXRD of the simulated and experimental patterns of **AlMC-8**.

3.4 The EDS spectra for AlMC-1 to AlMC-8

The presence of elements (Al, C, O, Cl, Br, and I) in **AlMC-1** to **AlMC-8** have been validated by EDS spectra (Supplementary Figures 62–69). Due to the lower mass percentage, N atoms on NO_3^- guests are hard to detect. However, the peaks of NO_3^- guests can be seen in the FT-IR spectra (Supplementary Figures 70–77).

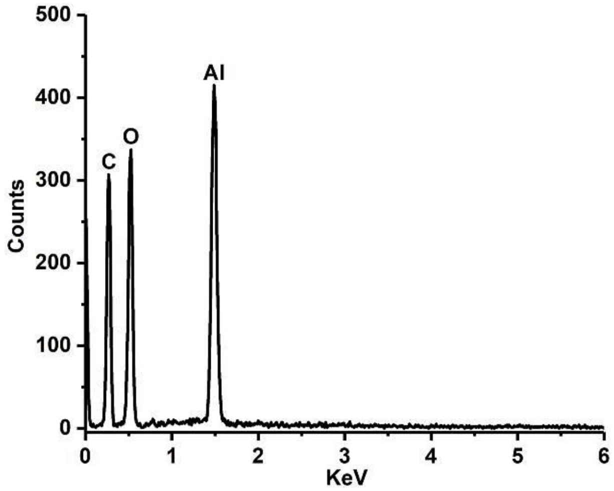

Supplementary Figure 62. The EDS spectrum of **AlMC-1** ($Al_{24}(BA)_{12}(OEt)_{24}(\mu_2\text{-}OH)_{24}(\mu_3\text{-}OH)_8\cdot(NO_3)_4\cdot(HOEt)_2\cdot(H_2O)_2$).

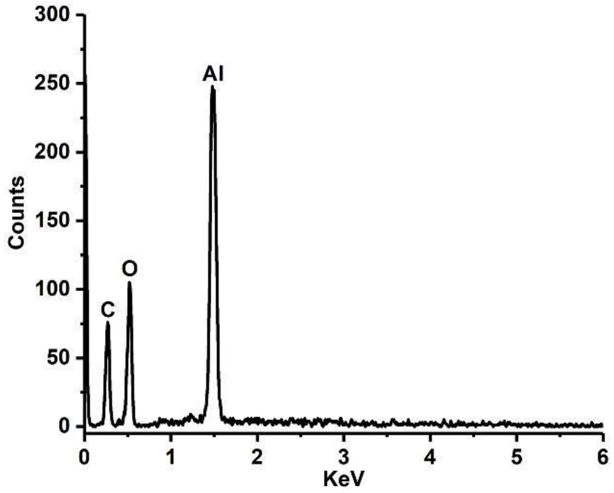

Supplementary Figure 63. The EDS spectrum of **AlMC-2** ($Al_{24}(BA)_{12}(O^nPr)_{24}(\mu_2\text{-}OH)_{24}(\mu_3\text{-}OH)_8\cdot(NO_3)_4\cdot(HO^nPr)_4$).

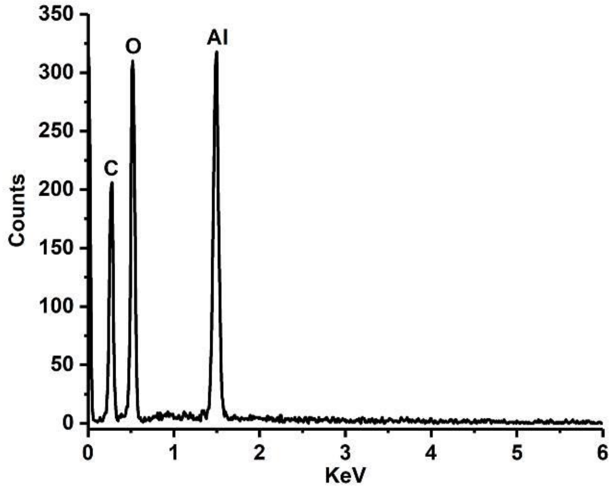

Supplementary Figure 64. The EDS spectrum of **AlMC-3** ($Al_{24}(BA)_{12}(OEt)_{24}(\mu_2\text{-}OH)_{24}(\mu_3\text{-}OH)_8\cdot(NO_3)_2\cdot(HOEt)_4\cdot(OEt)_2$).

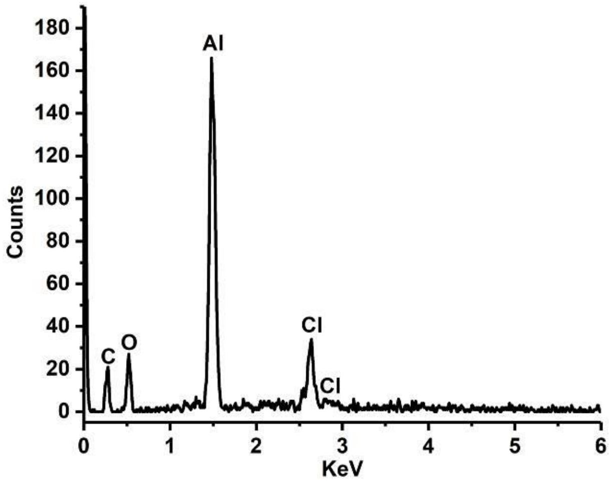

Supplementary Figure 65. The EDS spectrum of **AlMC-4** ($Al_{24}(BA)_{12}(OEt)_{24}(\mu_2\text{-}OH)_{24}(\mu_3\text{-}OH)_8\cdot NO_3\cdot Cl_3$).

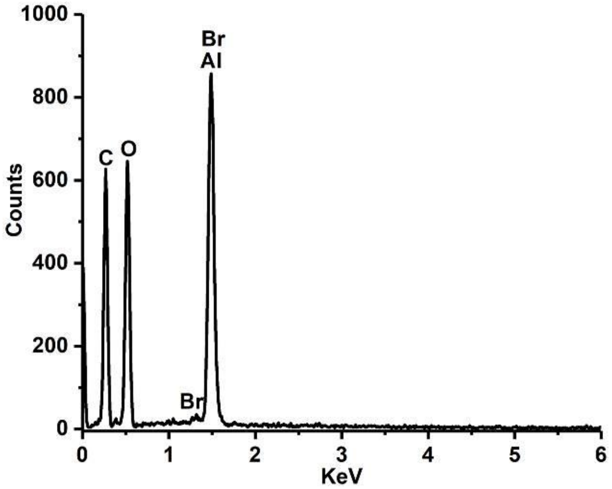

Supplementary Figure 66. The EDS spectrum of **AlMC-5** ($Al_{24}(BA)_{12}(OEt)_{24}(\mu_2\text{-}OH)_{24}(\mu_3\text{-}OH)_8\cdot NO_3\cdot Br_3$).

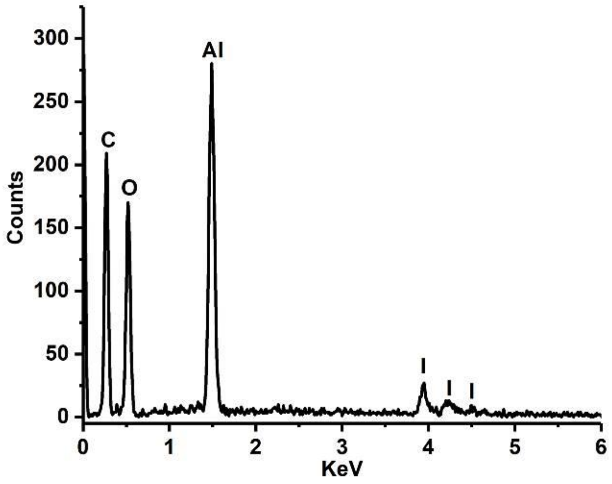

Supplementary Figure 67. The EDS spectrum of **AlMC-6** ($Al_{24}(BA)_{12}(OEt)_{24}(\mu_2\text{-}OH)_{24}(\mu_3\text{-}OH)_8\cdot I_4$).

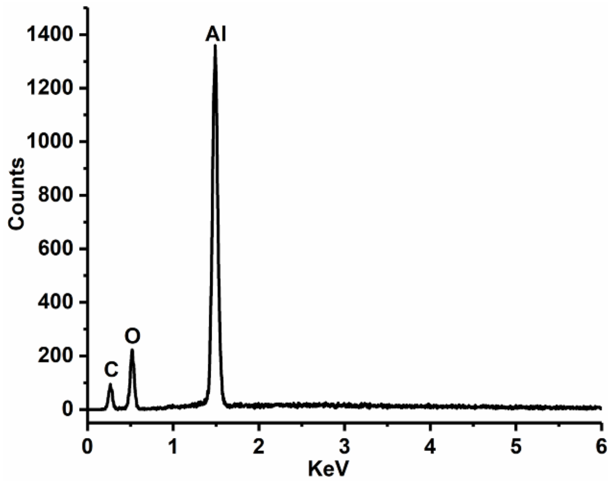

Supplementary Figure 68. The EDS spectrum of **AlMC-7** ($Al_{24}(BA)_{12}(OEt)_{24}(\mu_2\text{-}OH)_{24}(\mu_3\text{-}OH)_8\cdot(HNO_3)\cdot(OEt)_6\cdot(Al_6(BA)_6(OEt)_6(NO_3)_2)_{0.5}$).

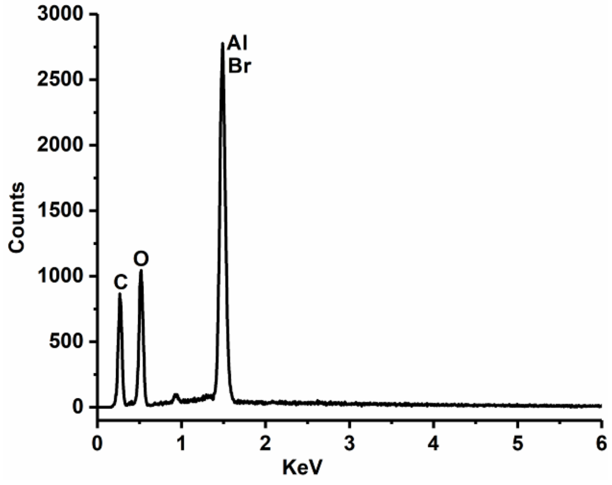

Supplementary Figure 69. The EDS spectrum of **AlMC-8** ($Al_{24}(BA)_{12}(OEt)_{24}(\mu_2\text{-}OH)_{24}(\mu_3\text{-}OH)_8\cdot NO_3\cdot Br_2\cdot OEt$).

3.5 FT-IR spectra for AlMC-1 to AlMC-8

FT-IR spectra have been recorded on solid samples palletized with KBr, which are presented in Supplementary Figure 70–77. In the high wavenumber region (v > 1000 cm^{-1}), the weak absorption bands at 3745−3147 cm^{-1}, 2981−2964 cm^{-1}, and 2933−2921 cm^{-1} can be ascribed to the stretching vibrational modes of O-H bonds in hydroxyl groups and C–H bonds in methylene or methyl groups. The characteristic stretching vibrations $v(CO_2^{2-})$ of in carboxylic groups and v(C=C) in benzene rings are overlapped from 1650 cm^{-1} to 1405 cm^{-1}. Among them, the asymmetric stretching vibration (v^{as}) and symmetric stretching vibration (v^{s}) of the carboxylate group can be attributed, namely, the band at 1569−1558 cm^{-1} is assigned to the $v^{as}(CO_2^{2-})$ whilst the signal at 1438−1429 cm^{-1} is ascribed to the $v^{s}(CO_2^{2-})$. The absorption peaks appearing at 1070−1062 cm^{-1} are assigned to the stretching vibrations of v(C−O) from alkoxide, and the stretching vibrations (v) and deformation vibration (δ) for NO_3^- guests respectively appeared at 1357−1348 cm^{-1} and 861−854 cm^{-1}. In the low wavenumber region (v < 1000 cm^{-1}), the absorptions in the region ca. 900−650 cm^{-1} for **AlMC-1** to **AlMC-8** can be attributed to the C–H in-plane or out-of-plane bending, ring breathing, and ring deformation absorptions of benzoates. These assignments are consistent with those previously reported.[30-32]

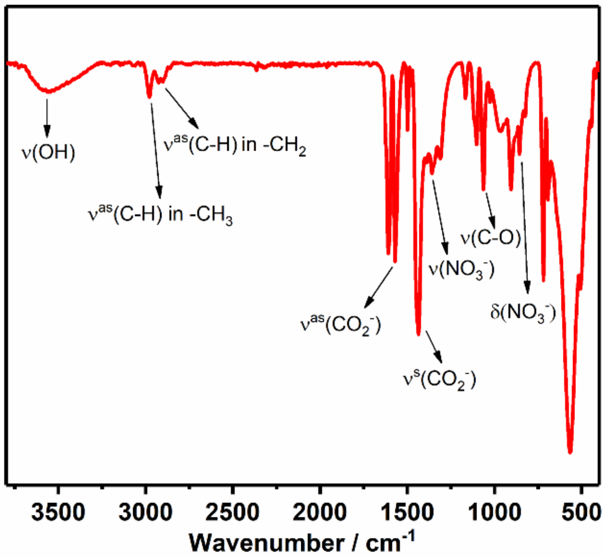

Supplementary Figure 70. The FT-IR spectrum of **AlMC-1**.

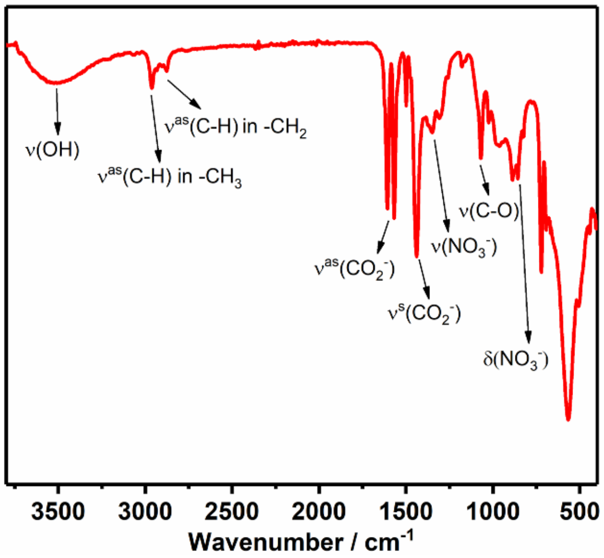

Supplementary Figure 71. The FT-IR spectrum of **AlMC-2**.

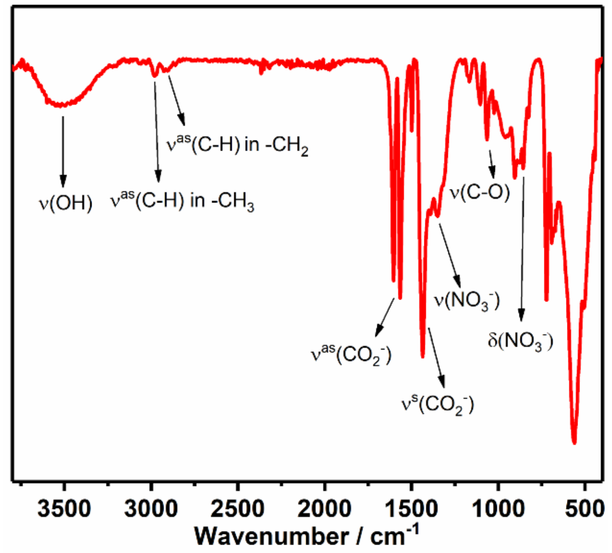

Supplementary Figure 72. The FT-IR spectrum of **AlMC-3**.

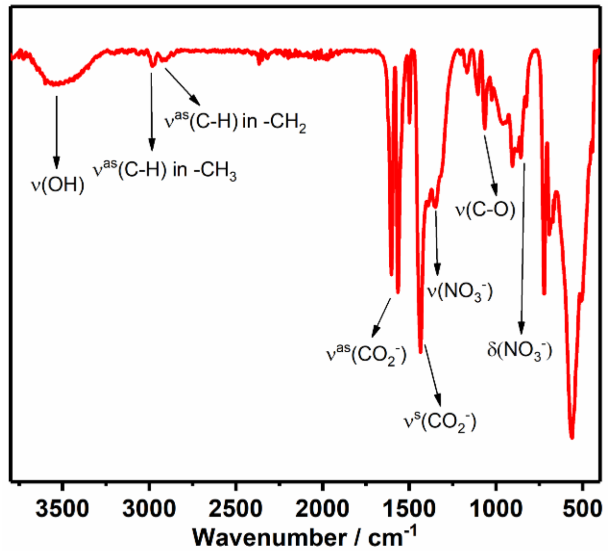

Supplementary Figure 73. The FT-IR spectrum of **AlMC-4**.

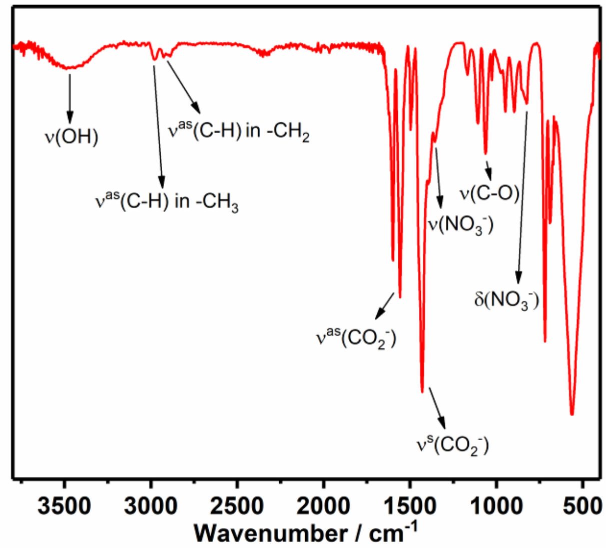

Supplementary Figure 74. The FT-IR spectrum of **AlMC-5**.

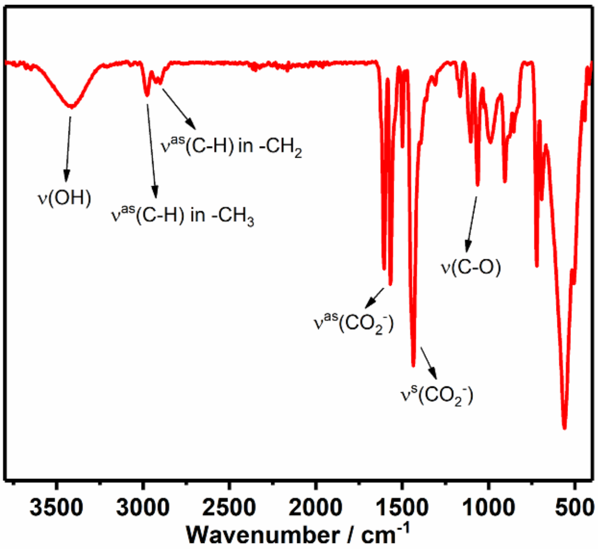

Supplementary Figure 75. The FT-IR spectrum of **AlMC-6**.

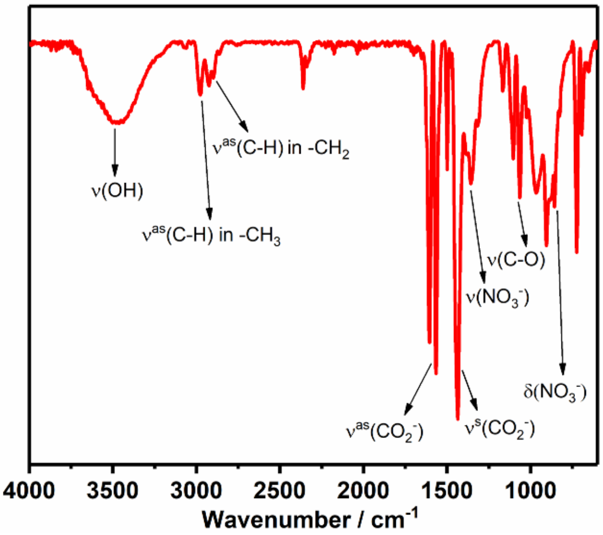

Supplementary Figure 76. The FT-IR spectrum of **AlMC-7**.

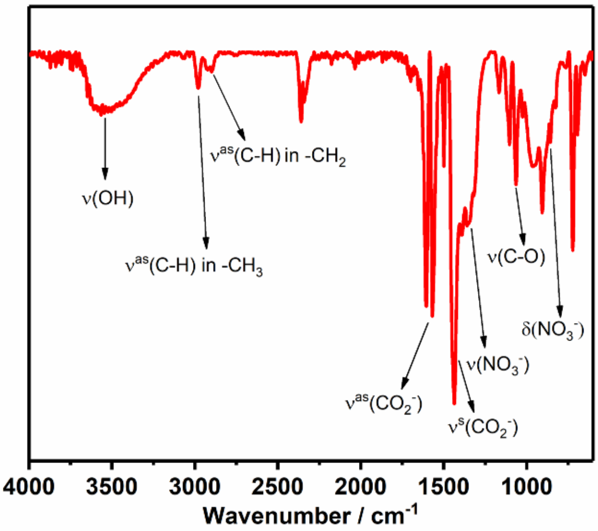

Supplementary Figure 77. The FT-IR spectrum of **AlMC-8**.

3.6 UV-vis spectra of AlMC-1 to AlMC-8

UV-vis spectra of colorless crystals **AlMC-1** to **AlMC-5**, **AlMC-7** and **AlMC-8** display narrow absorptions ranging from 200 to 350 nm, and the calculated adsorption edges are respectively 4.25 eV for **AlMC-1**, 4.25 eV for **AlMC-2**, 4.20 eV for **AlMC-3**, 4.30 eV for **AlMC-4**, 4.20 eV for **AlMC-5**, 4.22 eV for **AlMC-7**, and 4.23 eV for **AlMC-8** (Supplementary Figures 78–82, 84 and 85). While for I^- loaded **AlMC-6**, the crystal is yellow and presents enhanced adsorption from 350 to 450 nm (Supplementary Figure 83).

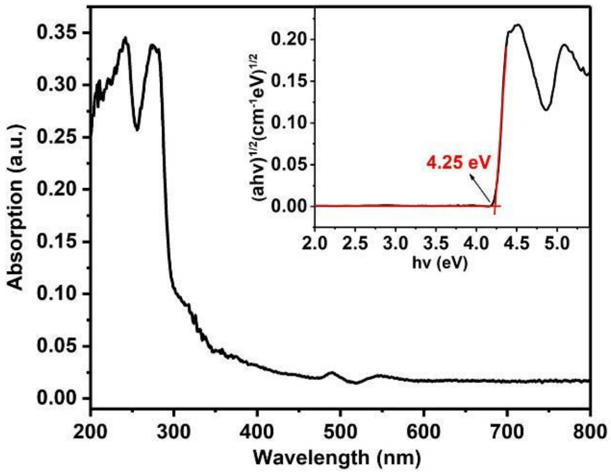

Supplementary Figure 78. The solid-state UV-vis absorption spectrum of **AlMC-1**. Inset is the optical bandgap.

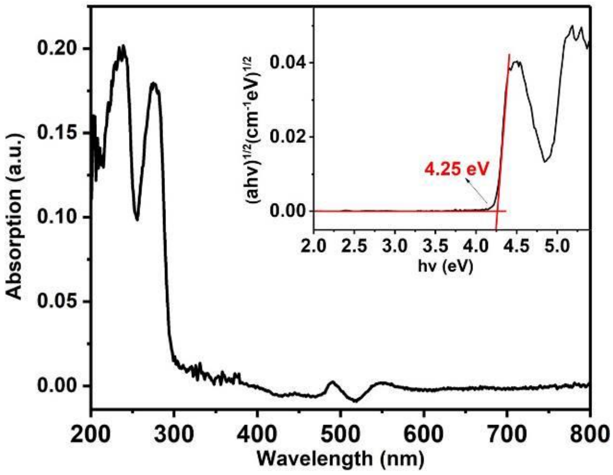

Supplementary Figure 79. The solid-state UV-vis absorption spectrum of **AlMC-2**. Inset is the optical bandgap.

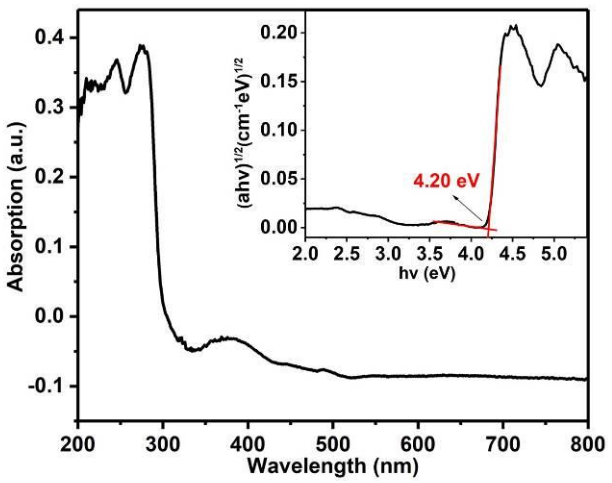

Supplementary Figure 80. The solid-state UV-vis absorption spectrum of **AlMC-3**. Inset is the optical bandgap.

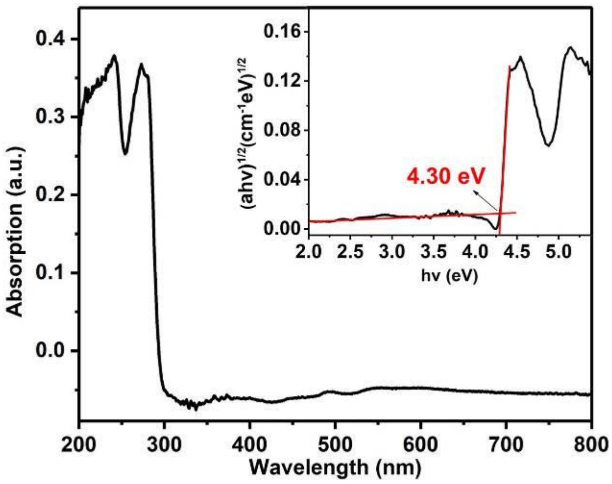

Supplementary Figure 81. The solid-state UV-vis absorption spectrum of **AlMC-4**. Inset is the optical bandgap.

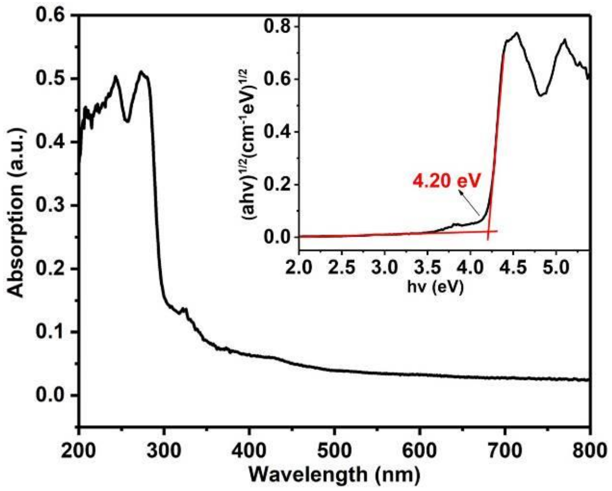

Supplementary Figure 82. The solid-state UV-vis absorption spectrum of **AlMC-5**. Inset is the optical bandgap.

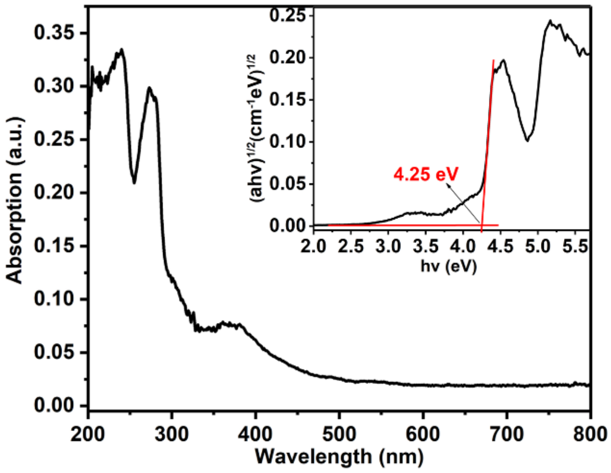

Supplementary Figure 83. The solid-state UV-vis absorption spectrum of **AlMC-6**. Inset is the optical bandgap. The adsorption peak at 375 nm is attributed to the characteristic absorption of iodine.

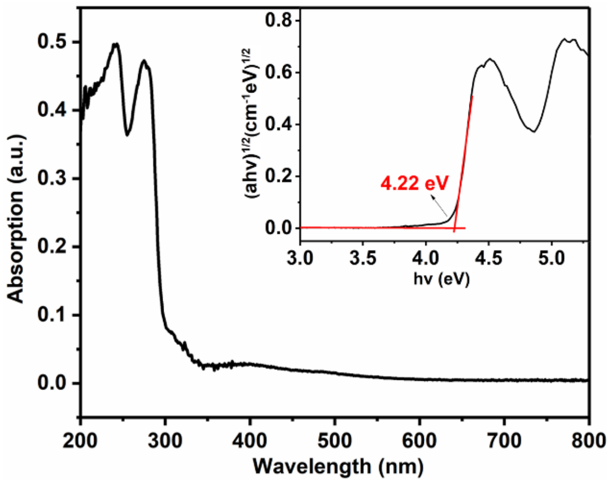

Supplementary Figure 84. The solid-state UV-vis absorption spectrum of **AlMC-7**. Inset is the optical bandgap.

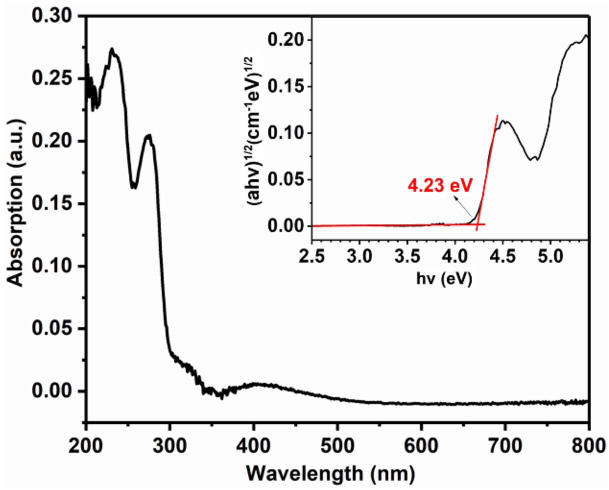

Supplementary Figure 85. The solid-state UV-vis absorption spectrum of **AlMC-8**. Inset is the optical bandgap.

3.7 TGA curves

The thermogravimetric analyses (TGA) of **AlMC-1** to **AlMC-7** show that Al_{24} cage decompose at 170 –180 ^{o}C. The first weight loss of 5.17% for **AlMC-1**, 5.93% for **AlMC-2**, 6.18% for **AlMC-3**, 6.96% for **AlMC-4**, 6.25% for **AlMC-5**, 2.77% for **AlMC-6**, and 7.64% for **AlMC-7** is corresponded to the release of guests and unresolved solvent molecules. The severe weight loss after 180 ^{o}C for all of them indicates the skeleton collapse of Al_{24} cage.

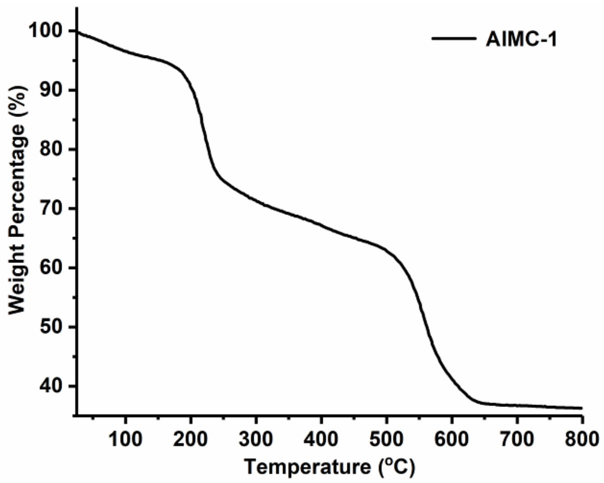

Supplementary Figure 86. TGA curve of **AlMC-1**.

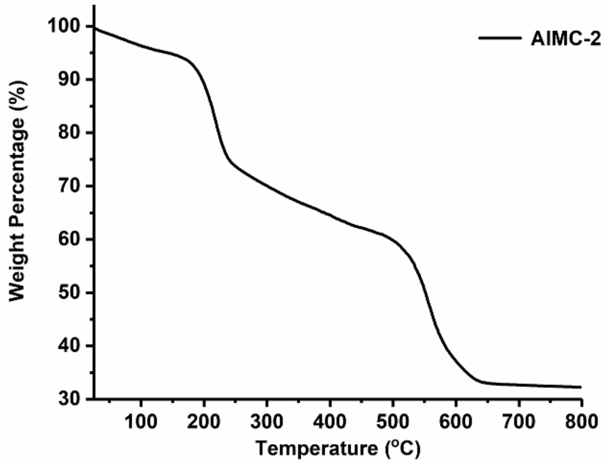

Supplementary Figure 87. TGA curve of **AlMC-2**.

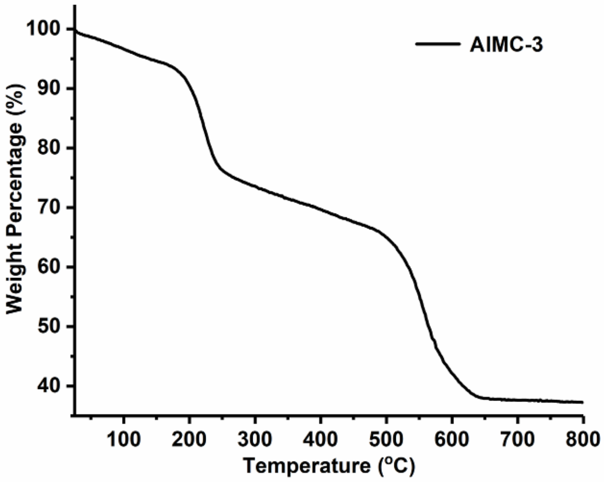

Supplementary Figure 88. TGA curve of **AlMC-3**.

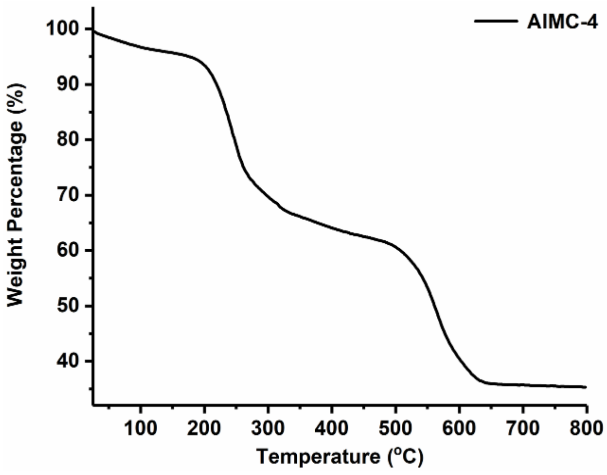

Supplementary Figure 89. TGA curve of **AlMC-4**.

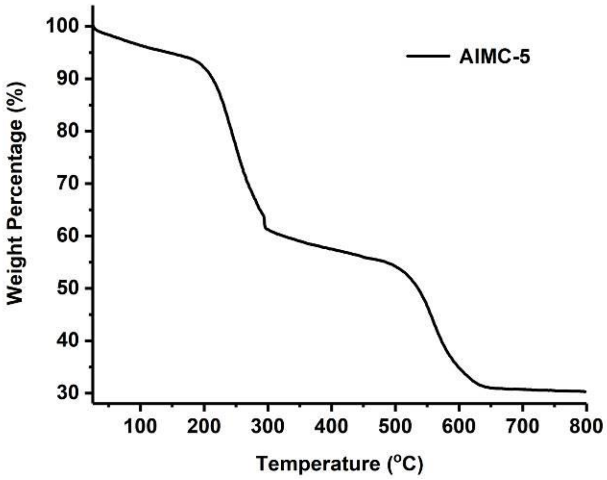

Supplementary Figure 90. TGA curve of **AlMC-5**.

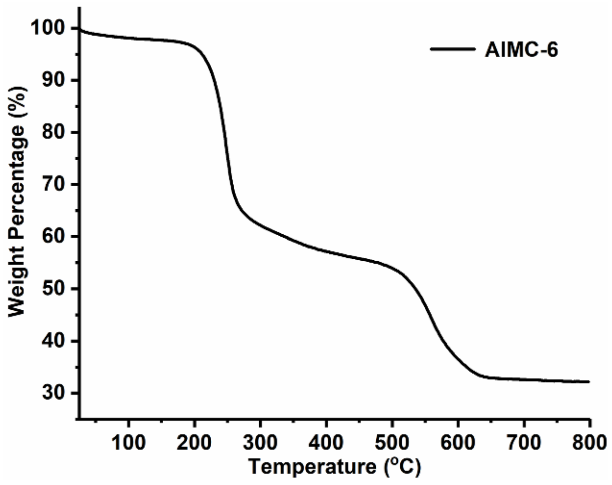

Supplementary Figure 91. TGA curve of **AlMC-6**.

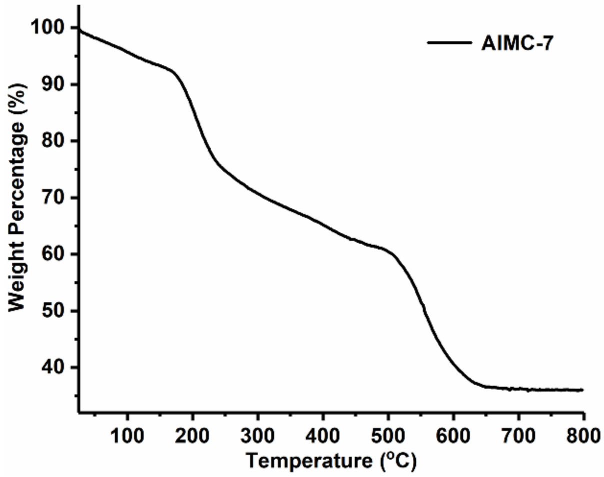

Supplementary Figure 92. TGA curve of **AlMC-7**.

3.8 Stability, ESI-MS, contact angles and pore distribution

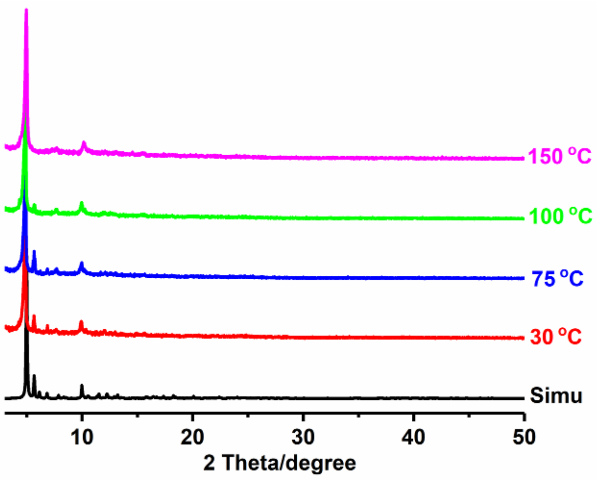

Supplementary Figure 93. In-situ temperature-dependent PXRD patterns of **AlMC-1**.

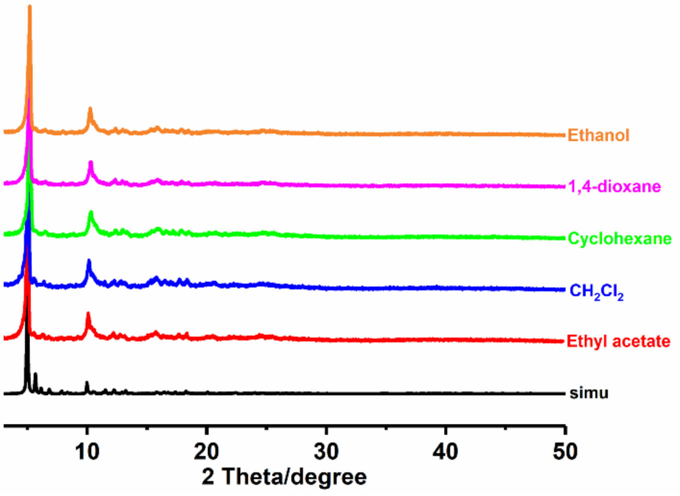

Supplementary Figure 94. The PXRD patterns of **AlMC-1** after soaking in different organic solvents at room temperature for 24h.

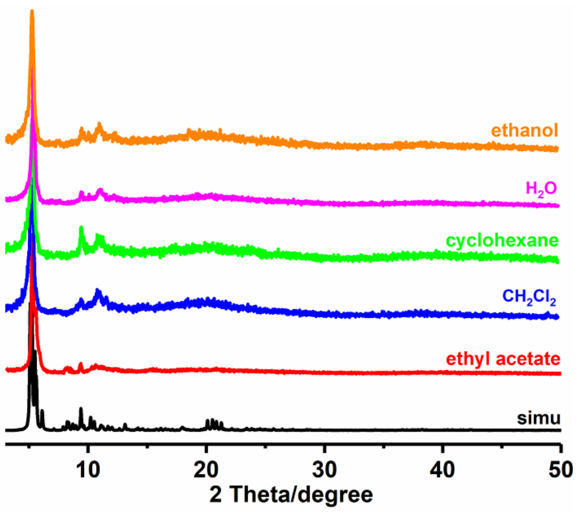

Supplementary Figure 95. The PXRD patterns of **AlMC-2** after soaking in H_2O and different organic solvents at room temperature for 24h.

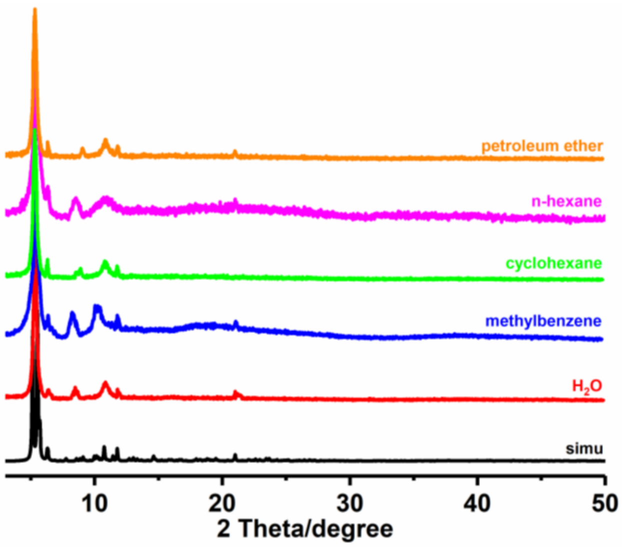

Supplementary Figure 96. The PXRD patterns of **AlMC-3** after soaking in H_2O and different organic solvents at room temperature for 24h.

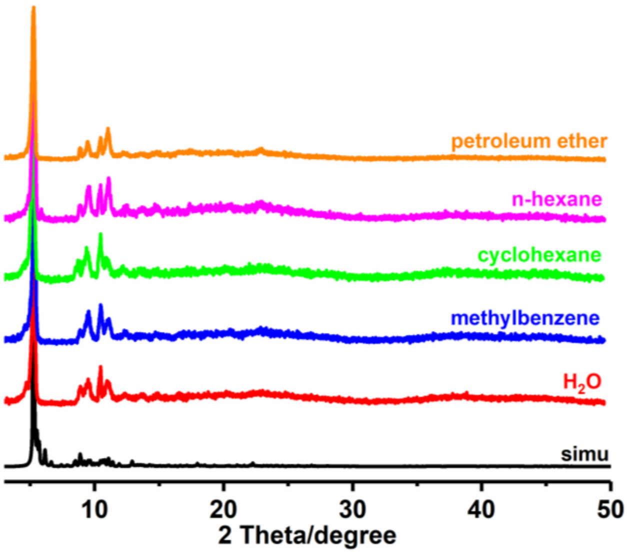

Supplementary Figure 97. The PXRD patterns of **AlMC-4** after soaking in H_2O and different solvents at room temperature for 24h.

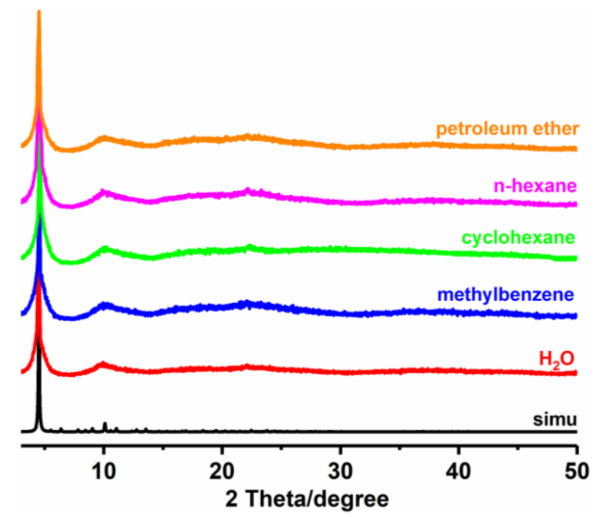

Supplementary Figure 98. The PXRD patterns of **AlMC-5** after soaking in H_2O and different solvents at room temperature for 24h.

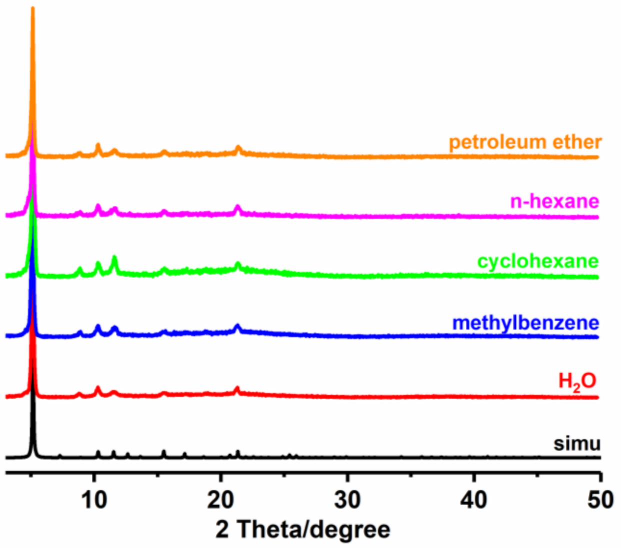

Supplementary Figure 99. The PXRD patterns of **AlMC-6** after soaking in H_2O and different solvents at room temperature for 24h.

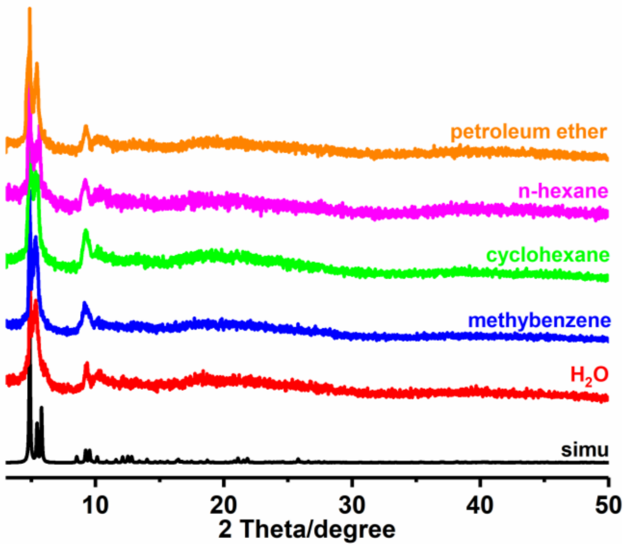

Supplementary Figure 100. The PXRD patterns of **AlMC-7** after soaking in H_2O and different solvents at room temperature for 24h.

Interestingly, although all of **AlMC-1** to **AlMC-7** can keep stable in water, they behave differently in organic solvents. **AlMC-1** and **AlMC-2** can keep stable in organic solvents with medium or low polarity, such as cyclohexane (polarity: 0.1), CH_2Cl_2 (polarity: 3.4), ethanol (polarity: 4.3), ethyl acetate (polarity: 4.3), 1,4-dioxane (polarity: 4.8). While, **AlMC-3** to **AlMC-7** will be dissolved in CH_2Cl_2, ethanol etc. solvents with medium polarity, and can only keep stable in petroleum ether, n-hexane, cyclohexane, methylbenzene.

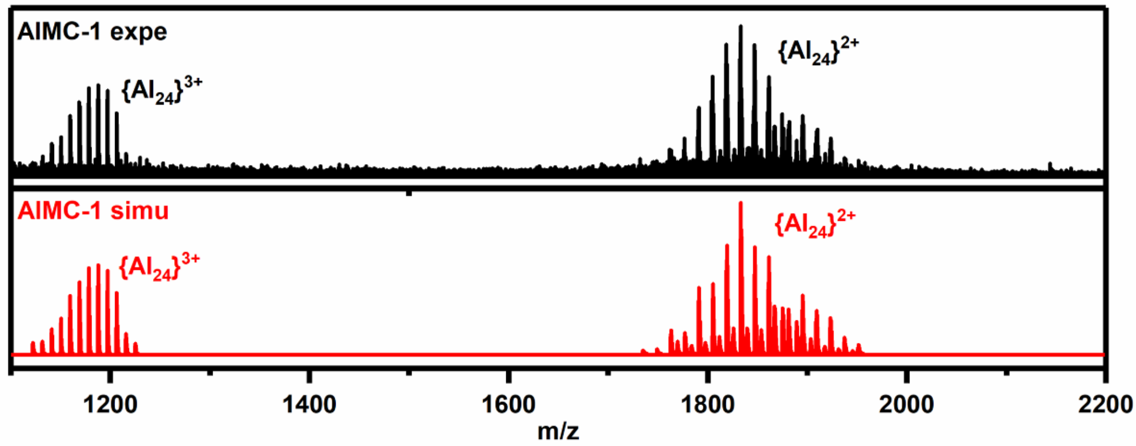

Supplementary Figure 101. Experimental and simulated mass spectra of **AlMC-1** in DMSO under positive mode. The solution was diluted with MeCN before entering the mass spectrometer to avoid clogging.

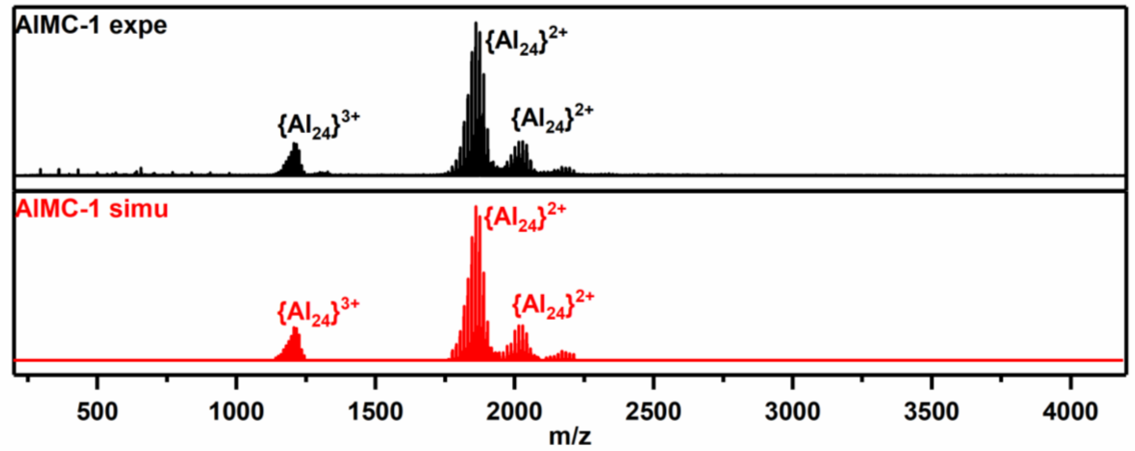

Supplementary Figure 102. Experimental and simulated mass spectra of **AlMC-1** in MeCN under positive mode.

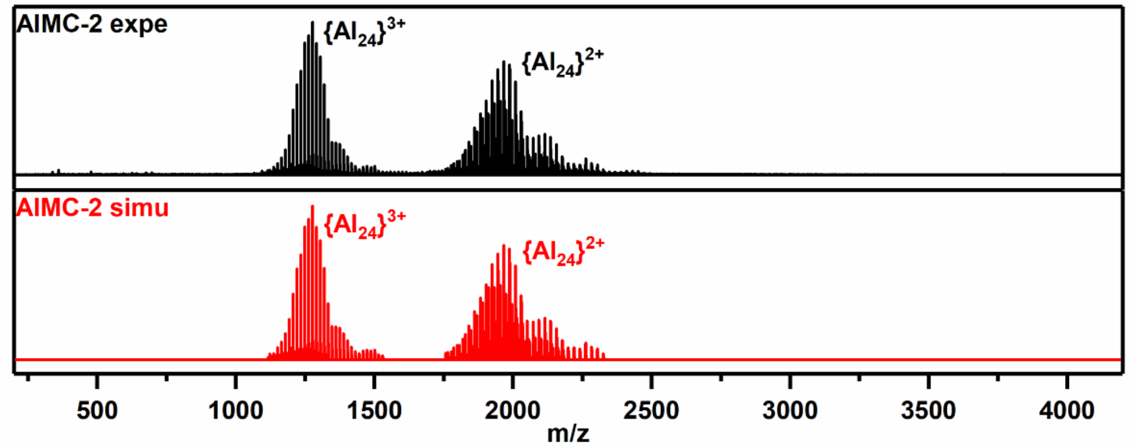

Supplementary Figure 103. Experimental and simulated mass spectra of **AlMC-2** in MeCN under positive mode.

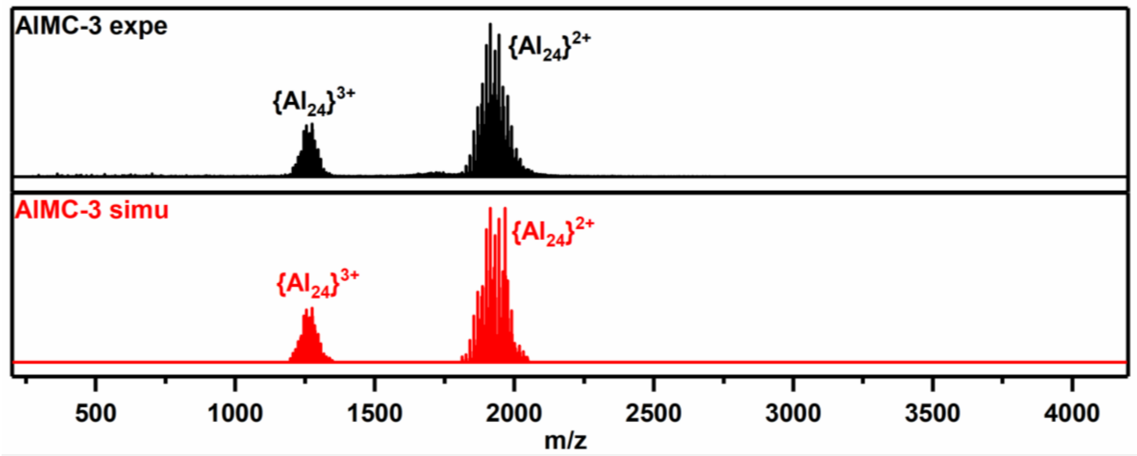

Supplementary Figure 104. Experimental and simulated mass spectra of **AlMC-3** in MeCN under positive mode.

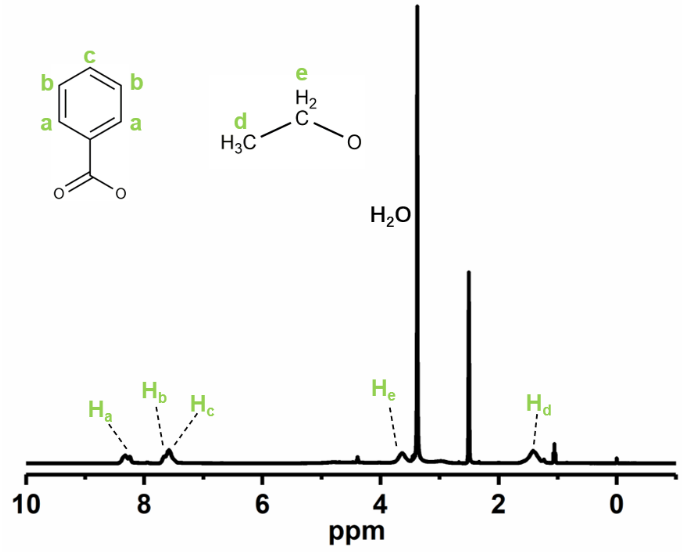

Supplementary Figure 105. ^{1}H NMR spectrum of **AlMC-1** in DMSO-d_6.(Identified the absorption peak)

The peaks around 7.35–7.75 ppm and 8.15–8.45 ppm (with the integrals ratio of 1.56 (3.12 : 2)) are attributed to the three kinds of H atoms in benzoate. The signals for H_b and H_c are overlap, which is common in assemble structures.[33-35] The signals for coordinated ethoxides can be found at 1.15–1.60 ppm (H_d) and 3.45–3.81 ppm (H_e) (integrals ratio 3.00 : 2.07), which can be assigned to the vibrations of H from methyl and methylene groups. The peaks at 0.95–1.13 ppm, 3.40–3.45 ppm and 4.30–4.40 ppm are from free ethanol guest or exfoliated ethoxides from Al_{24} cage hosts. The other peaks may originate from impurities in DMSO-d_6. Thus, the ^{1}H NMR result is consistent with mass spatra, indicating that the Al_{24} cage can be well dissolved in DMSO.

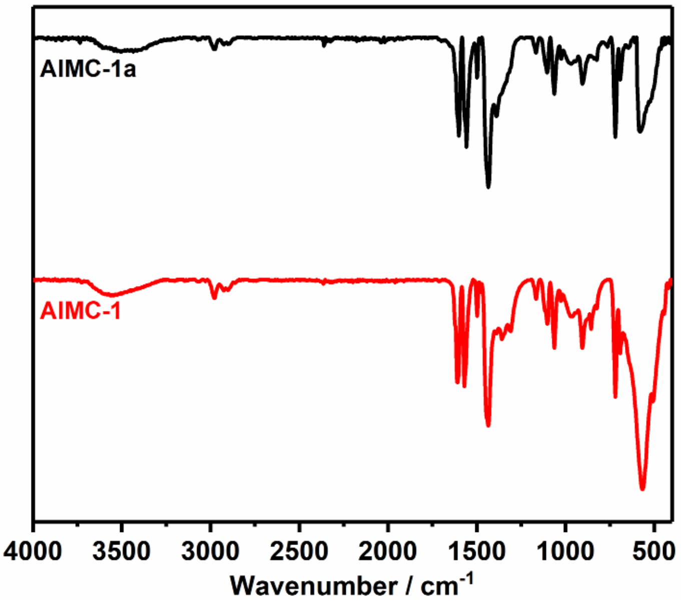

Supplementary Figure 106. The IR spectrum of **AlMC-1a** which is obtained by immersing **AlMC-1** in water for 7 days.

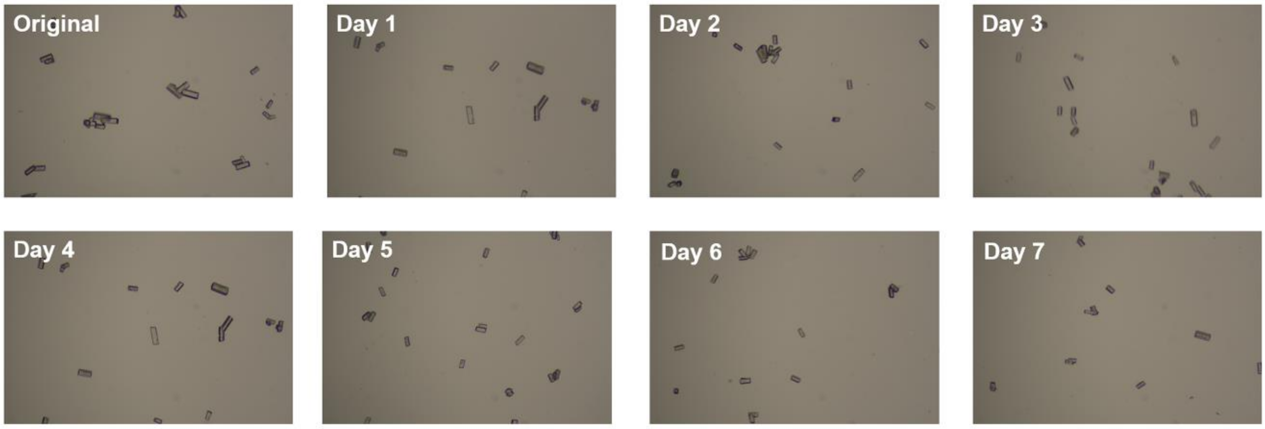

Supplementary Figure 107. The crystals morphology of **AlMC-1** immersing in water for 7 days.

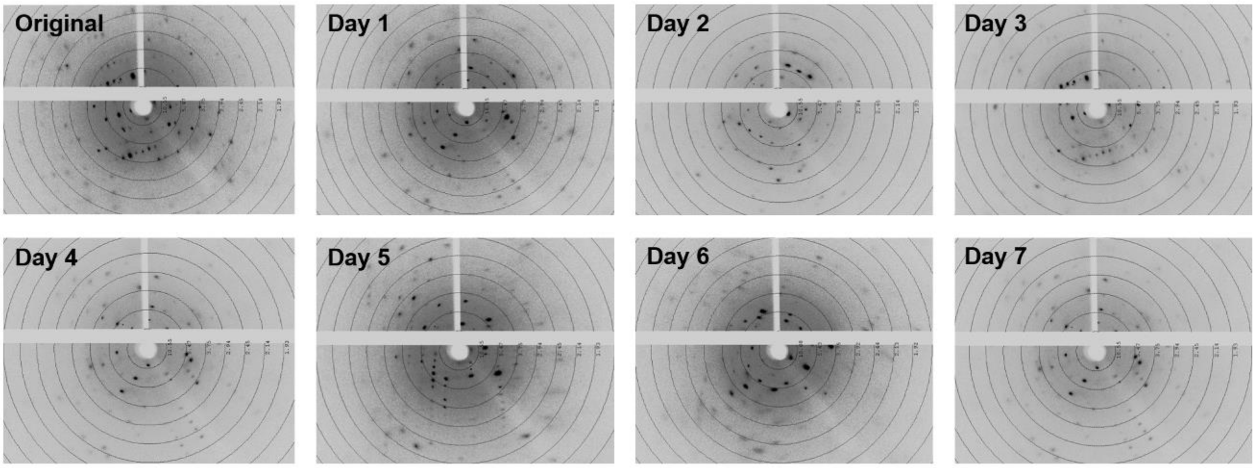

Supplementary Figure 108. The diffraction images of **AlMC-1** immersing in water for 7 days.

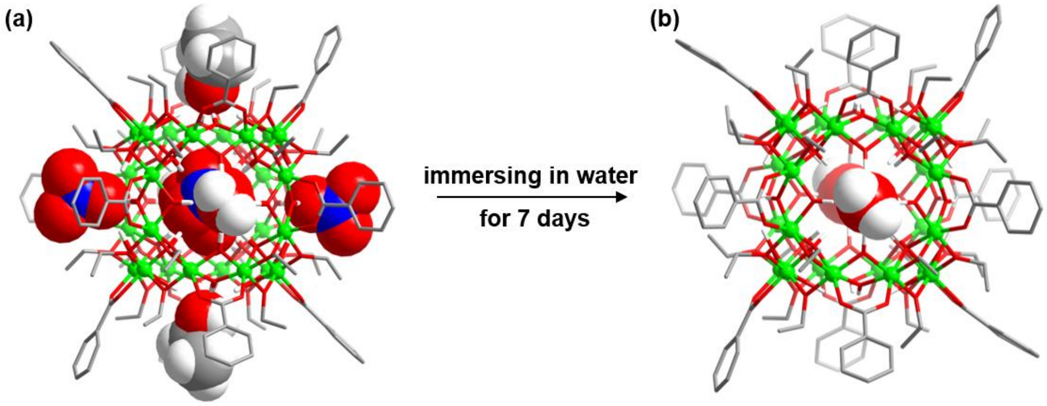

Supplementary Figure 109. The stability of Al_{24} skeleton in water. The structure of **AlMC-1a** (b) obtained by immersing **AlMC-1** (a) in water for 7 days.

The NO_3^- and HOEt guests are swapped out by H_2O, thus they cannot be found in **AlMC-1a**. However, other counter ions or new H_2O sites cannot be identified due to their highly disorder after immersing. Similar difficulty in resolving the counter ions was also reported in other work.[36]

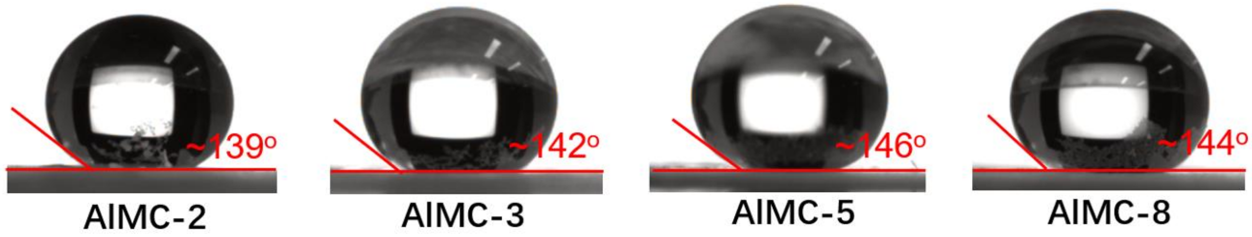

Supplementary Figure 110. Contact angle tests for **AlMC-2**, **AlMC-3**, **AlMC-5** and **AlMC-8**.

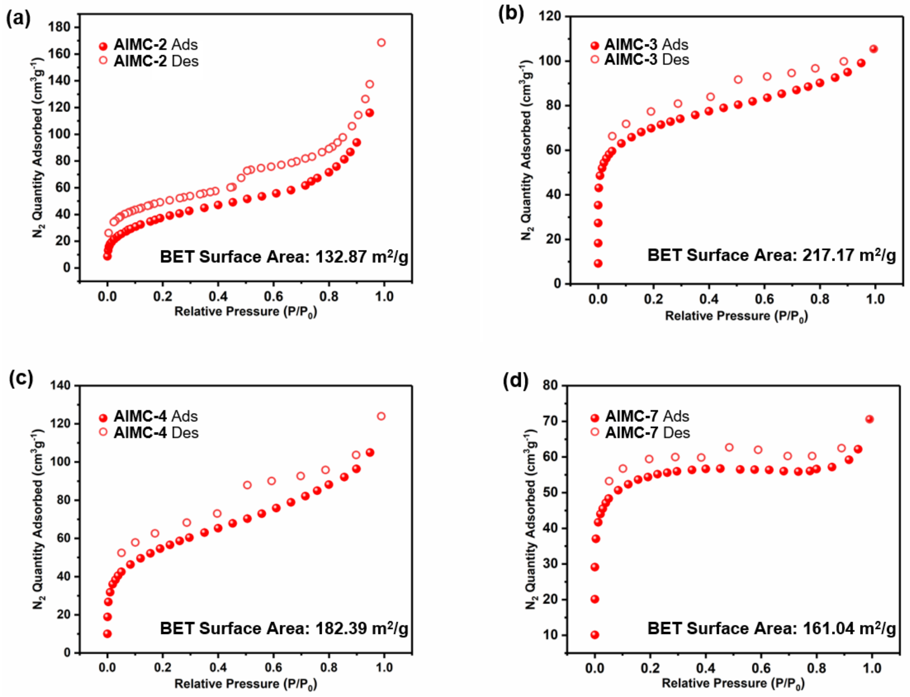

Supplementary Figure 111. The N_2 gas sorption isotherms at 77 K. (a) **AlMC-2**; (b) **AlMC-3**; (c) **AlMC-4** and (d) **AlMC-7**.

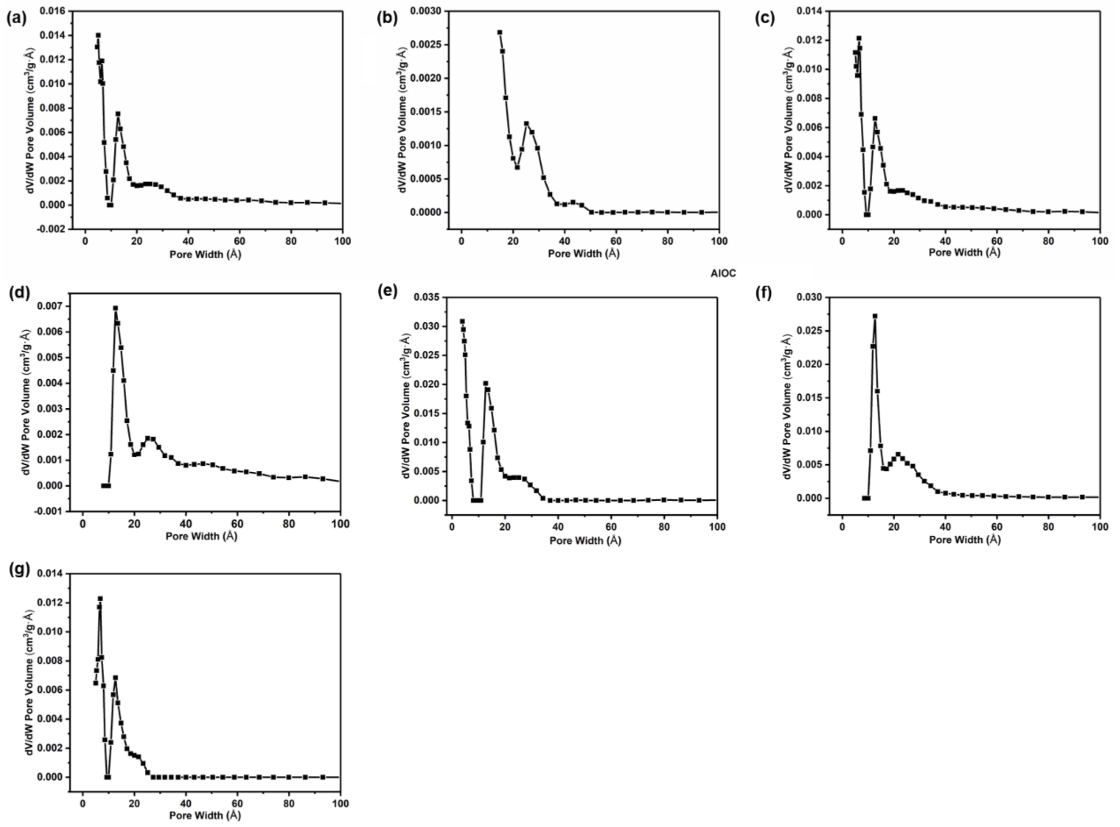

Supplementary Figure 112. The calculated pore size distribution profiles. (a) **AlMC-1**; (b) **AlMC-2**; (c) **AlMC-3**; (d) **AlMC-4**; (e) **AlMC-5**; (f) **AlMC-6** and (g) **AlMC-7**.

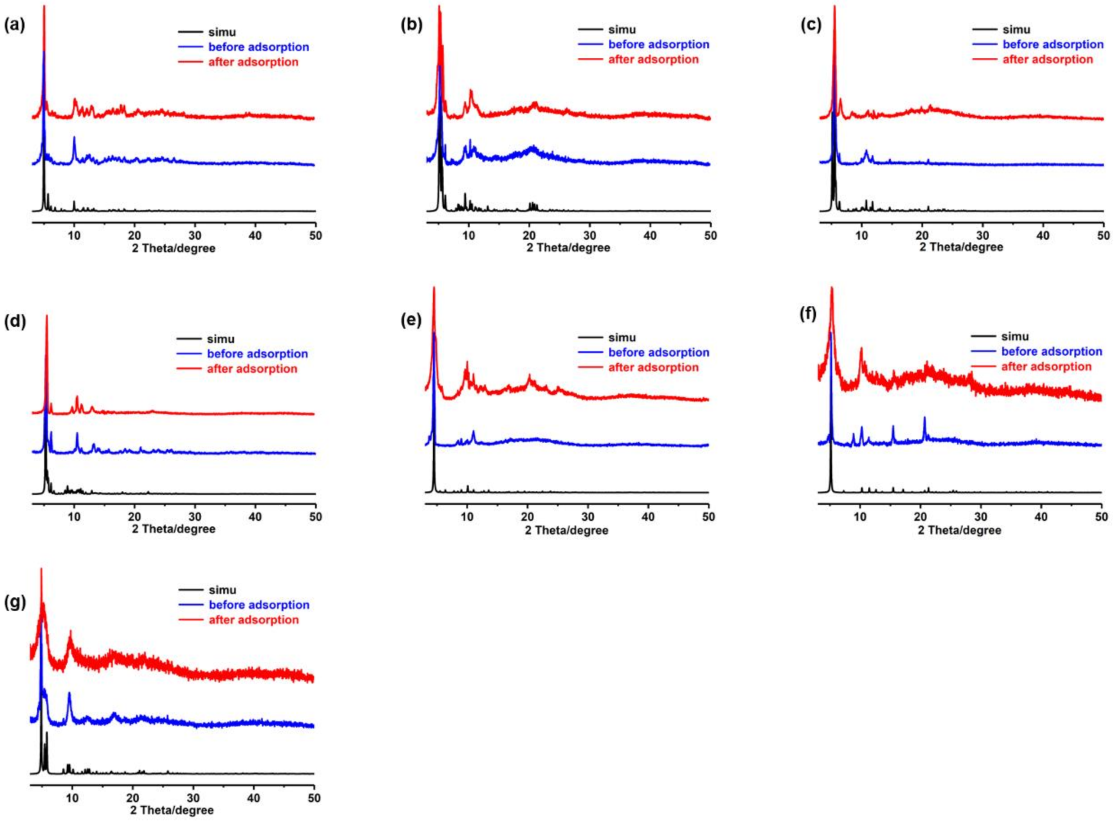

Supplementary Figure 113. PXRD patterns after adsorption. (a) **AlMC-1**; (b) **AlMC-2**; (c) **AlMC-3**; (d) **AlMC-4**; (e) **AlMC-5**; (f) **AlMC-6** and (g) **AlMC-7**.

3.9 Iodine adsorption tests

Removal rate calculation: **AlMC-1** (20 mg) was added into an aqueous I_2/KI (20 mL, 400 ppm). The supernatant solution was extracted at various time points for each UV-vis absorbance measurement. The absorbance peak at 286 nm was chosen to calculate the content, and the absorbance value for the original solution was normalized to 100%. The removal ratios (R) of iodine were calculated using $R = (C_0 - C_t) / C_0 \times 100\%$ (where C_0 and C_t represent the initial concentration and concentration at time t, respectively).

Bisulfite starch titration: To verify the uptake capacity, the I_2 content in the filtrate was also measured. First, a 2% aqueous starch indicator (2 mL) was added to the filtrate. The solution was then titrated by adding aqueous sodium bisulfite solution (0.05 M) dropwise until the solution color turned from dark to transparent. The I_2 content was calculated as 255.5 mg, indicating that the adsorbed I_2 in **AlMC-1** was 44.5 mg. This value is closed to gravimetric analysis (51.5 mg).

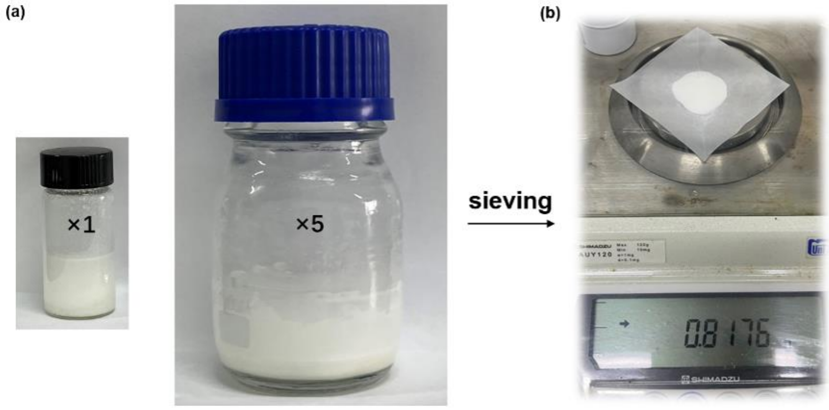

Supplementary Figure 114. The larger scale synthesis of **AlMC-1**. (a) The photos of reactors. (b) After sieving with a 200-mesh sieve, we can obtain 817.6 mg of pure phase crystals.

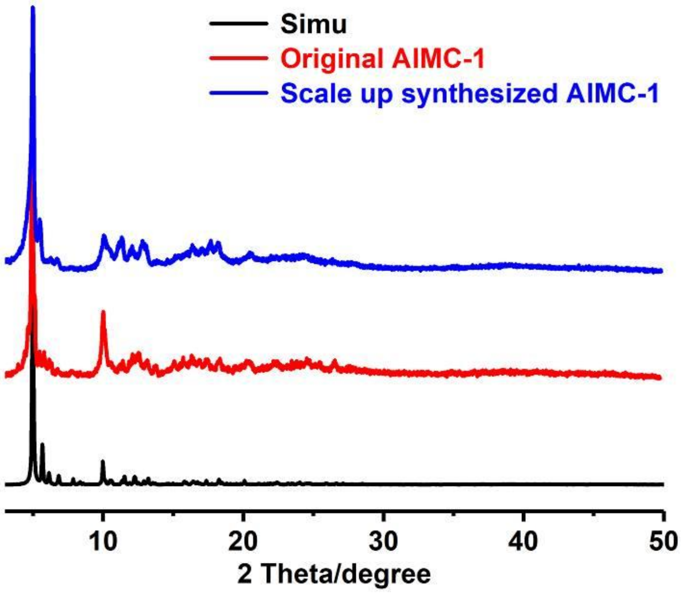

Supplementary Figure 115. The PXRD pattern of **AlMC-1** from scale up synthesis.

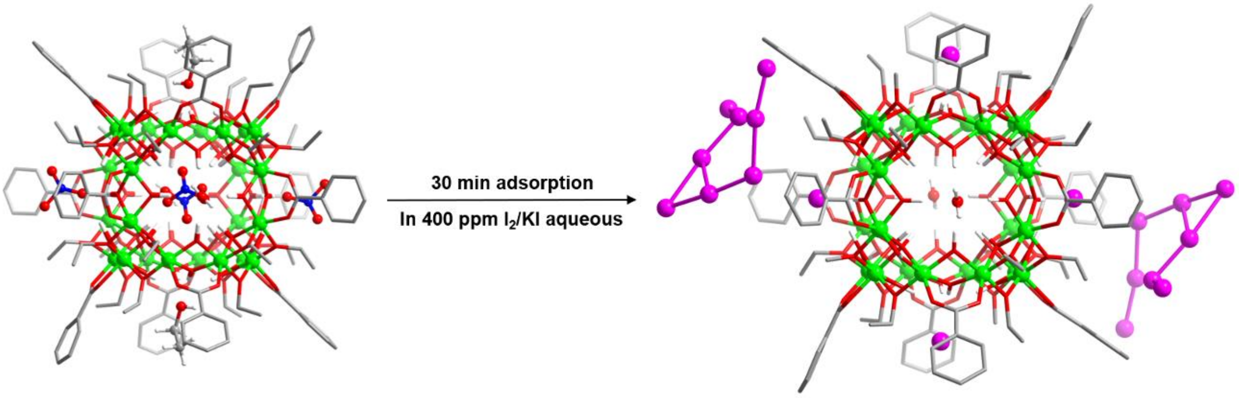

Supplementary Figure 116. The single crystal structure of **AlMC-1** after being soaked in 400 ppm I_2/KI aqueous for 30min. I^- ions have replaced the original HOEt and NO_3^- guests.

The iodine capture rate for **AlMC-1** is very fast. After immersion in 400 ppm I_2/KI aqueous for 30min, the HOEt and NO_3^- guests on the face-center of the ***tcu*** cage have been replaced by I^-, and 7.75 iodine around each Al_{24} cage can be located (denoted as: **I@Al$_{24}$–400ppm–30min**) (Supplementary Figure 116). New iodine species can also be observed on the outside of the cage. These iodine sites are consistent with those after adsorbing for 48h. Detailed iodine distribution, supramolecular forces and occupancies are provided in Supplementary Figure 128 and 129.

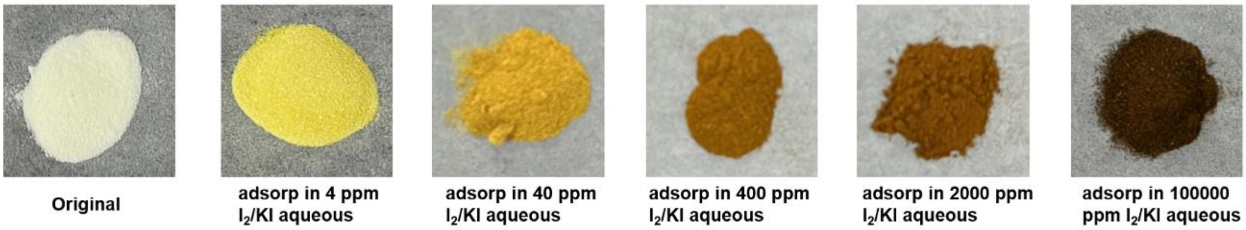

Supplementary Figure 117. Crystal color variation after adsorption in I_2/KI aqueous with different concentrations for 48h.

The crystal colors gradually deepened with the concentration of aqueous I_2/KI used. Their solid-state UV-vis absorption spectra have been performed, and the absorption edge shifts into the visible range which are respectively 2.42 eV, 2.09 eV, 1.73 eV, 1.66 eV and 1.65 eV for samples after immersing in 4 ppm, 40 ppm, 400 ppm, 2000 ppm and 100000 ppm I_2/KI aqueous.

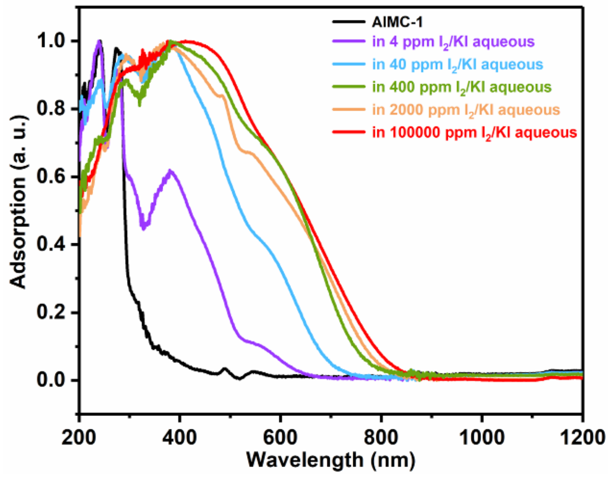

Supplementary Figure 118. The solid-state UV-vis absorption spectra of **AlMC-1** after soaking in aqueous I_2/KI at different concentrations.

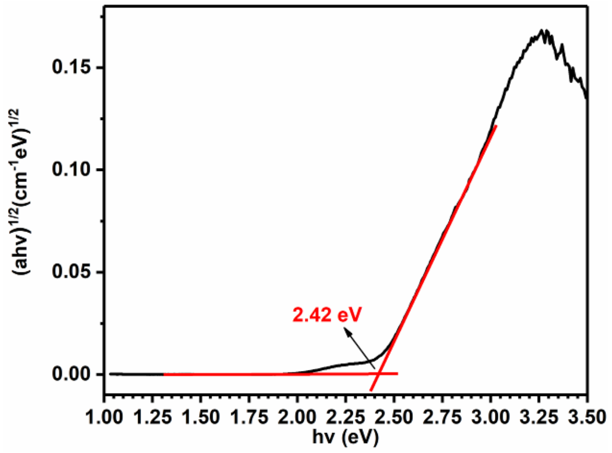

Supplementary Figure 119. The cut-off in the adsorption edge for **AlMC-1** after soaking in 4 ppm I_2/KI aqueous for 48h.

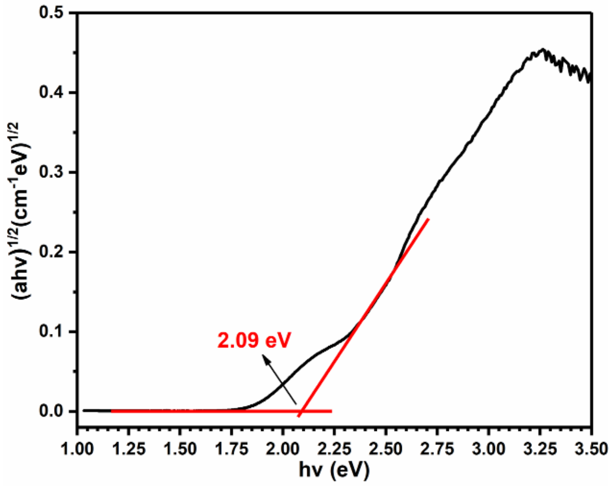

Supplementary Figure 120. The cut-off in the adsorption edge for **AlMC-1** after soaking in 40 ppm I_2/KI aqueous for 48h.

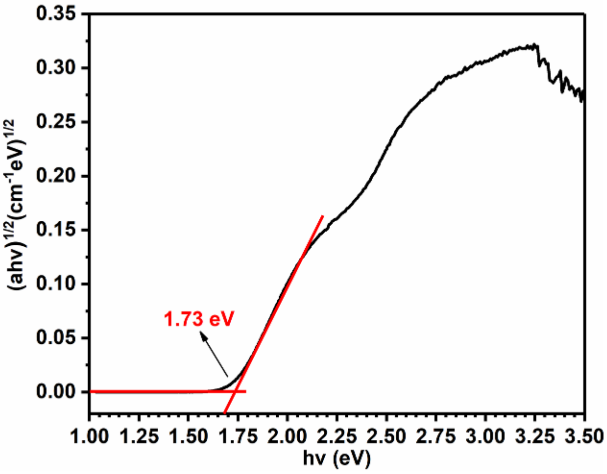

Supplementary Figure 121. The cut-off in the adsorption edge for **AlMC-1** after soaking in 400 ppm I_2/KI aqueous for 48h.

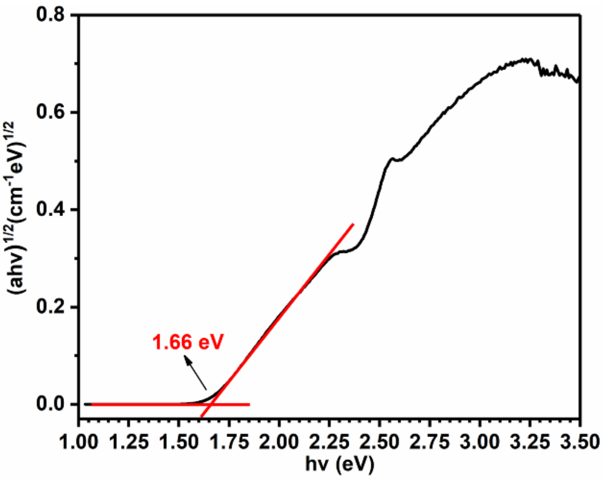

Supplementary Figure 122. The cut-off in the adsorption edge for **AlMC-1** after soaking in 2000 ppm I_2/KI aqueous for 48h.

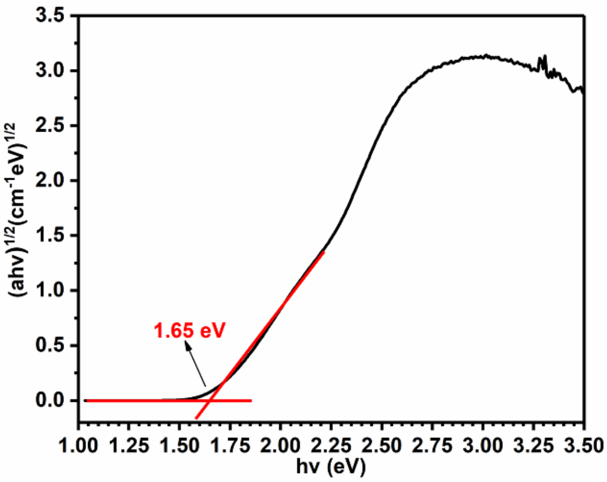

Supplementary Figure 123. The cut-off in the adsorption edge for **AlMC-1** after soaking in 100000 ppm I_2/KI aqueous for 48h.

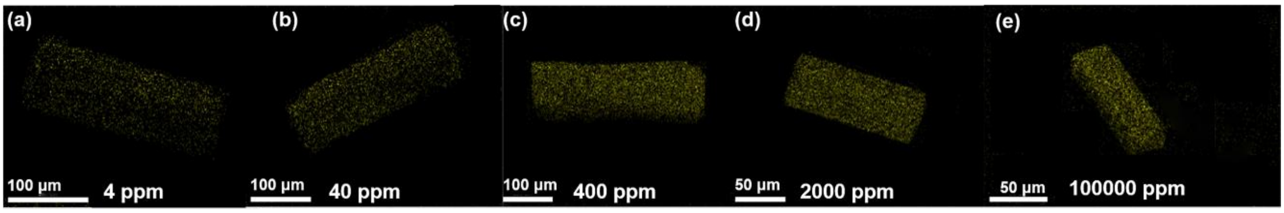

Supplementary Figure 124. EDS mapping of iodine element for **AlMC-1** after adsorption in I_2/KI aqueous at different concentrations for 48h. (a) In 4 ppm I_2/KI aqueous; (b) in 40 ppm I_2/KI aqueous; (c) in 400 ppm I_2/KI aqueous; (d) in 2000 ppm I_2/KI aqueous; (e) in 100000 ppm I_2/KI aqueous.

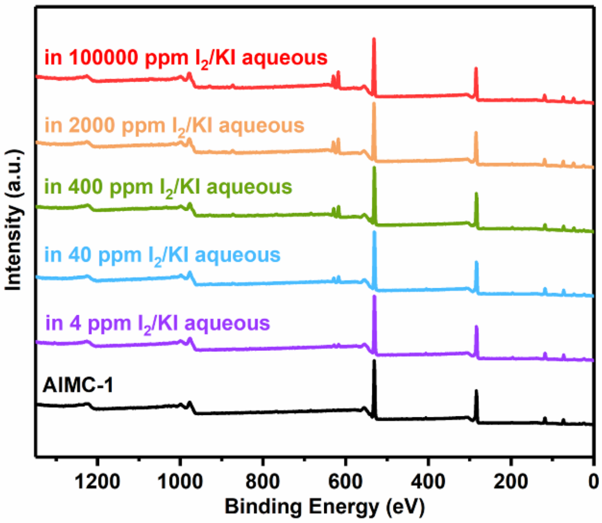

Supplementary Figure 125. Full survey *XPS* spectra of I-loaded **AlMC-1** crystals after adsorption in I_2/KI aqueous at different concentrations.

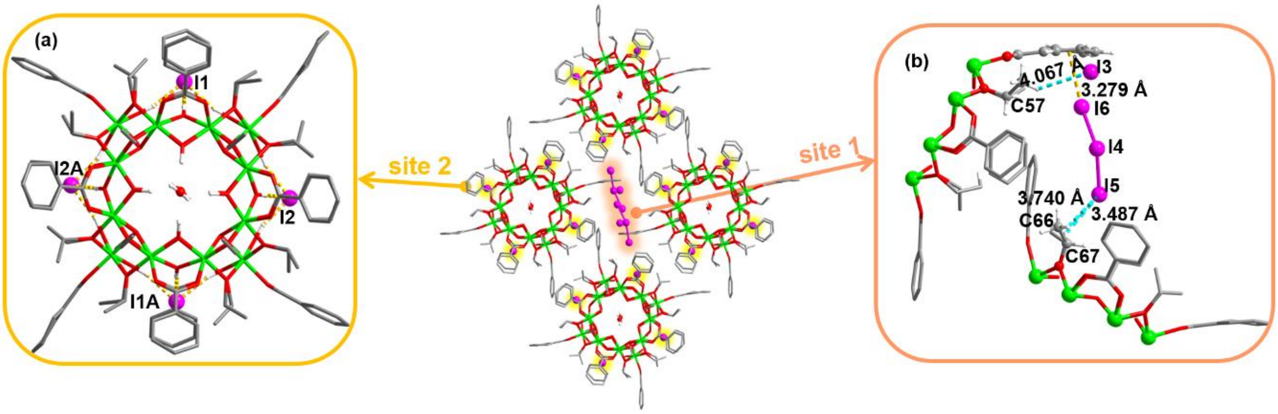

Supplementary Figure 126. The distributions and detailed interactions of iodine in **I@Al$_{24}$–4ppm–48h** when **AlMC-1** is immersed in aqueous 4 ppm I_2/KI for 48 h. (a) The O–H···I hydrogen-bond interactions between I and μ$_2$-OH groups in site 2; (b) the C–H···I hydrogen-bond interactions and I···π interactions in site 1.

The low concentration adsorbed state was obtained by immersing crystals of **AlMC-1** in aqueous 4 ppm I_2/KI for 48h (Supplementary Figure 126). The crystal structure indicates that there are 0.52 I atoms per unit cell (denoted as: **I@Al$_{24}$–4ppm–48h**). Four square windows (site 2) on O$_{24}$-***tro*** cages were occupied by I^- ions which form strong hydrogen-bonding interactions with the μ$_2$-OH groups (Supplementary Figure 126a). Concurrently, the channels (site 1) along the [001] direction can also accommodate I_3^- and I^- ion. Among them, I_3^- ion interactions with the Al$_{24}$ cage using benzoate groups (I(I6)···π interactions: 3.279 Å) and coordinated alkoxide (C(C66)-H···I(I5): 3.740 Å), and I^- ions interactions with alkoxide through weak C(C57)-H···I(I3) (4.067 Å) (Supplementary Figure 126b).

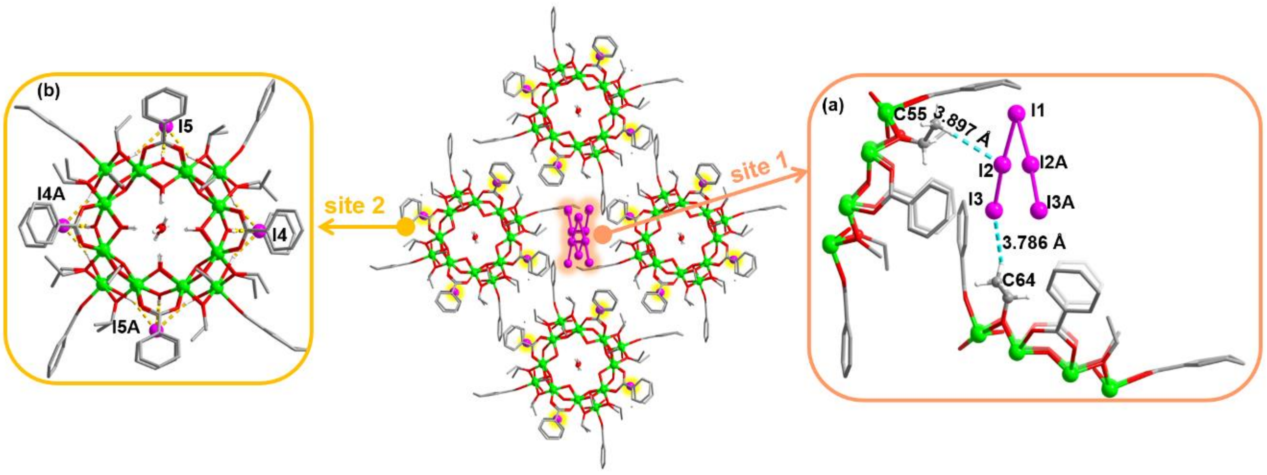

Supplementary Figure 127. The distributions and detailed interactions of iodine atoms in **I@Al$_{24}$–40ppm–48h** when **AlMC-1** are immersed in an aqueous 40 ppm I_2/KI solution for 48 h. (a) the C–H⋯I hydrogen-bond interactions in site 1; (b) the O–H⋯I hydrogen-bond interactions between I and μ$_2$-OH groups in site 2.

When the concentration of I_2/KI aqueous was increased to 40 ppm, each Al$_{24}$ cage can further contain 2.5 I atoms (**I@Al$_{24}$–40ppm–48h**), and I_3^- electron density peaks within channels (site 1) can be observed (Supplementary Figure 127). The I_3^- guests are disordered, approximately parallel to the square windows and generate two strong C-H⋯I interactions (3.897 Å and 3.786 Å) with alkoxide groups (Supplementary Figure 127a).

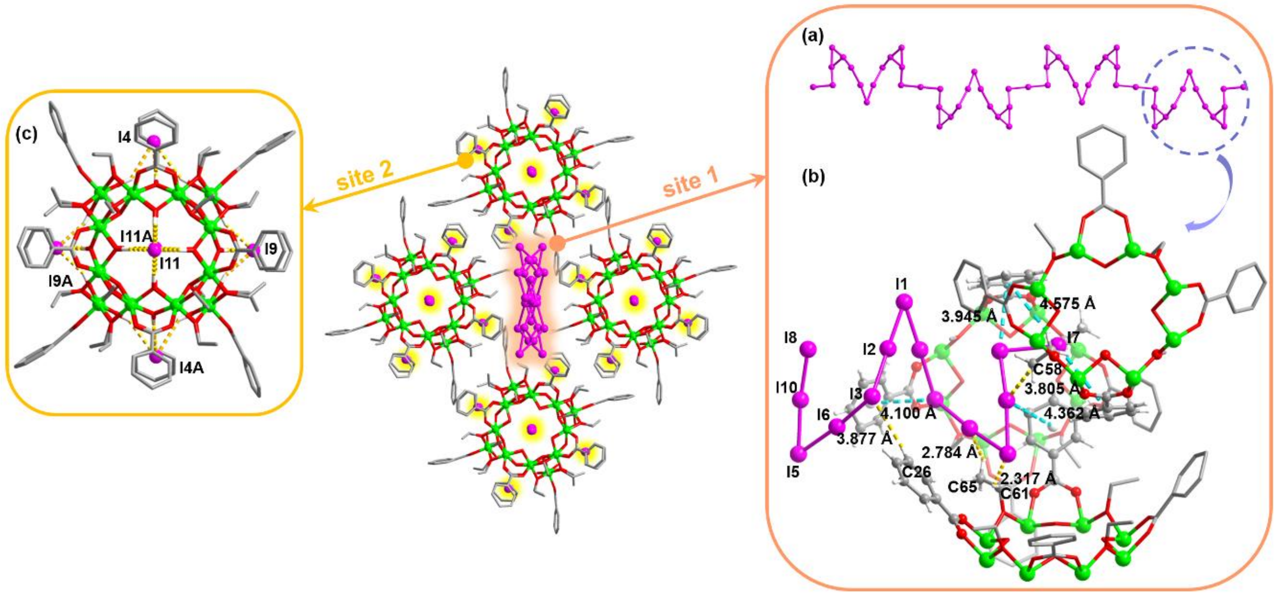

Supplementary Figure 128. The distributions and detailed interactions of iodine in **I@Al$_{24}$–400ppm–48h** when **AlMC-1** is immersed in 400 ppm aqueous I_2/KI solution for 48 h. (a) The polyiodide in the channel of **AlMC-1**; (b) the C–H⋯I hydrogen-bond interactions and I⋯π interactions in site 1; (c) the O–H⋯I hydrogen-bond interactions in site 2.

A third intermediate structure can be obtained by immersing crystals of **AlMC-1** in 400 ppm aqueous I_2/KI for 48h (Supplementary Figure 128) (denoted as: **I@Al$_{24}$–400ppm–48h**). The absorbed I species in the channel (site 1) are closely bonded with I–I bond lengths of 2.606–2.992 Å (Supplementary Figure 128a), and 10 I atoms surround each Al$_{24}$ cage. The interactions between these I guests and the Al$_{24}$ host are summarized in Supplementary Figure 128b and 128c.

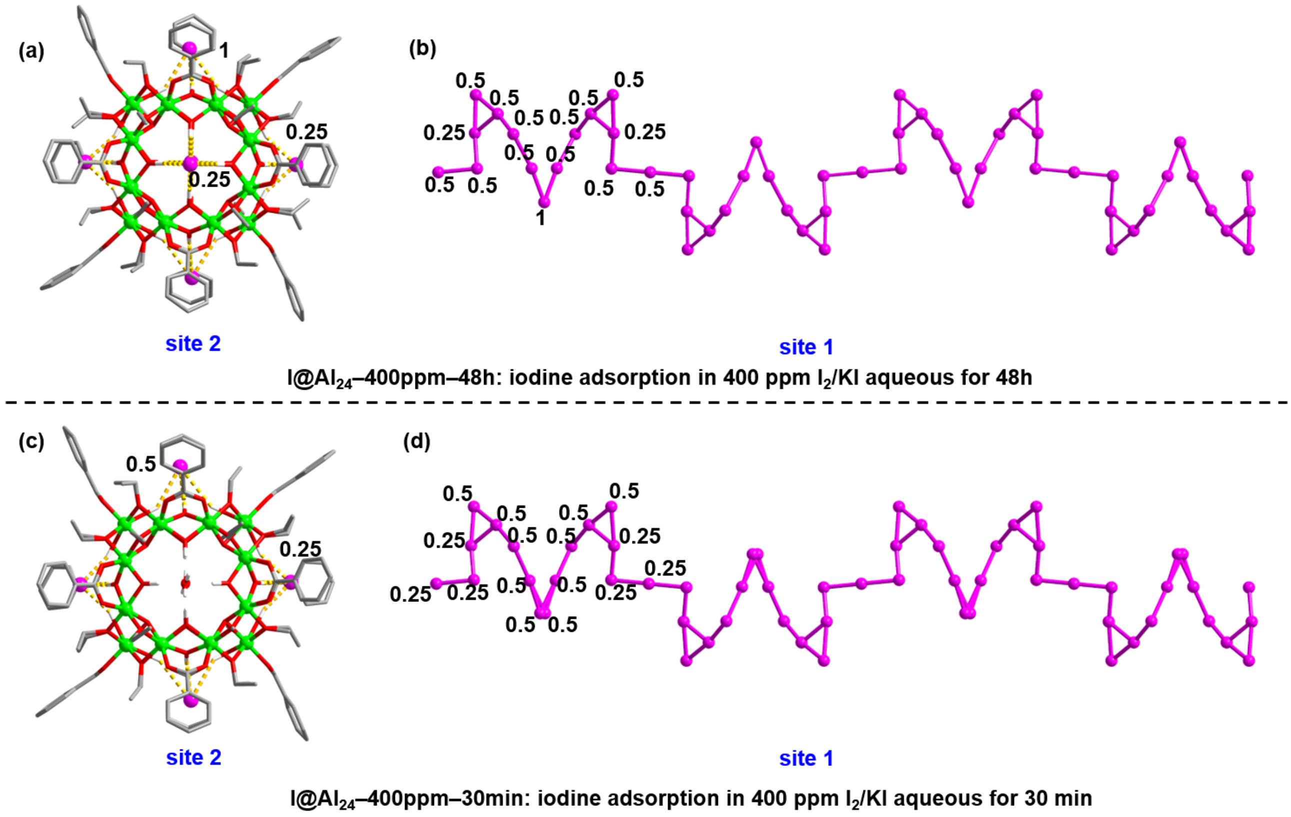

Supplementary Figure 129. The comparison of occupies of iodine after immersing crystals in 400 ppm aqueous I_2/KI solution for 48 h (a, site 2; b, site 1) and 30 min (c, site 2; d, site 1). (a and b) Iodine adsorption in 400 ppm I_2/KI solution for 48 h; (c and d) iodine adsorption in 400 ppm I_2/KI solution for 30 min.

Some iodine occupancies for 30 min adsorption are lower when compared with that of 48 h adsorption, indicating that the extension of adsorption time is favorable for iodine aggregation (Supplementary Figure 129). Notably, a pair of H_2O guests in site 2 were replaced by I^- when adsorption 48h rather than 30 min. Whereas, all of iodine species in site 1 can be found when adsorption only 30 min. Thus, the iodine capture in intermolecular channel is faster than that in μ_2-OH square window.

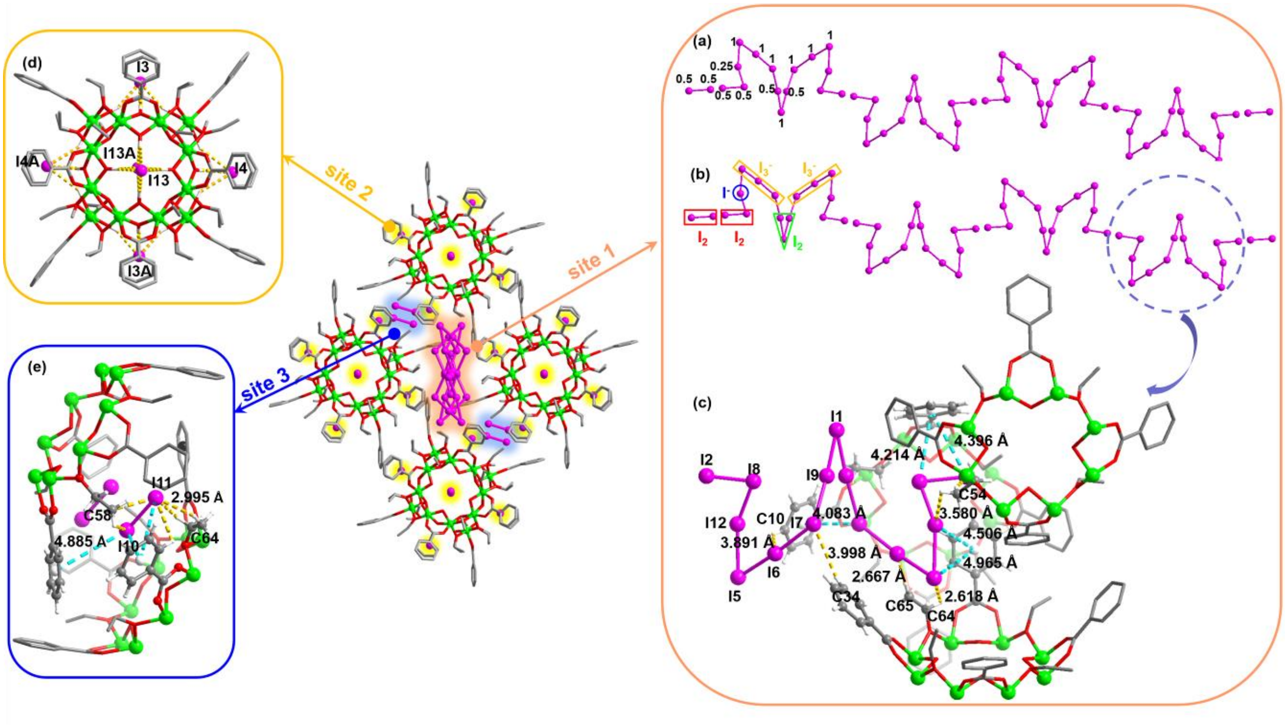

Supplementary Figure 130. The distributions and detailed interactions of iodine in **I@Al$_{24}$–2000ppm–48h** when **AlMC-1** is immersed in 2000 ppm aqueous I_2/KI solution for 48 h. (a) The occupies of iodine in the channel of **AlMC-1**; (b) the distribution of iodine species on site 1; (c) the C–H⋯I hydrogen-bond interactions and I⋯π interactions in the channels; (d) the O–H⋯I hydrogen-bond interactions in site 2; (e) the C–H⋯I hydrogen-bond interactions and I⋯π interactions in site 3.

As the concentration is increasing to 2000 ppm, the zigzag 1D chain is preserved in the channels (Supplementary Figure 130a), and the relevant peaks in Fourier difference map are further enhanced. The polyiodide in site 1 can be regarded as an aggregation of I^- ions, I_2 molecules and I_3^- ions (Supplementary Figure 130b and 130c). Interestingly, the calixarene-like macrocyclic units (site 3) can also accommodate one I_2 molecule for increased adsorption (Supplementary Figure 130e) which is bound through hydrogen bonding to two alkoxides and two benzoates (C-H⋯I interactions: 2.820–3.387 Å; I⋯π interactions: 4.799–4.885 Å). Here, 15.5 I atoms are located around the Al_{24} cage (denoted as: **I@Al$_{24}$–2000ppm–48h**).

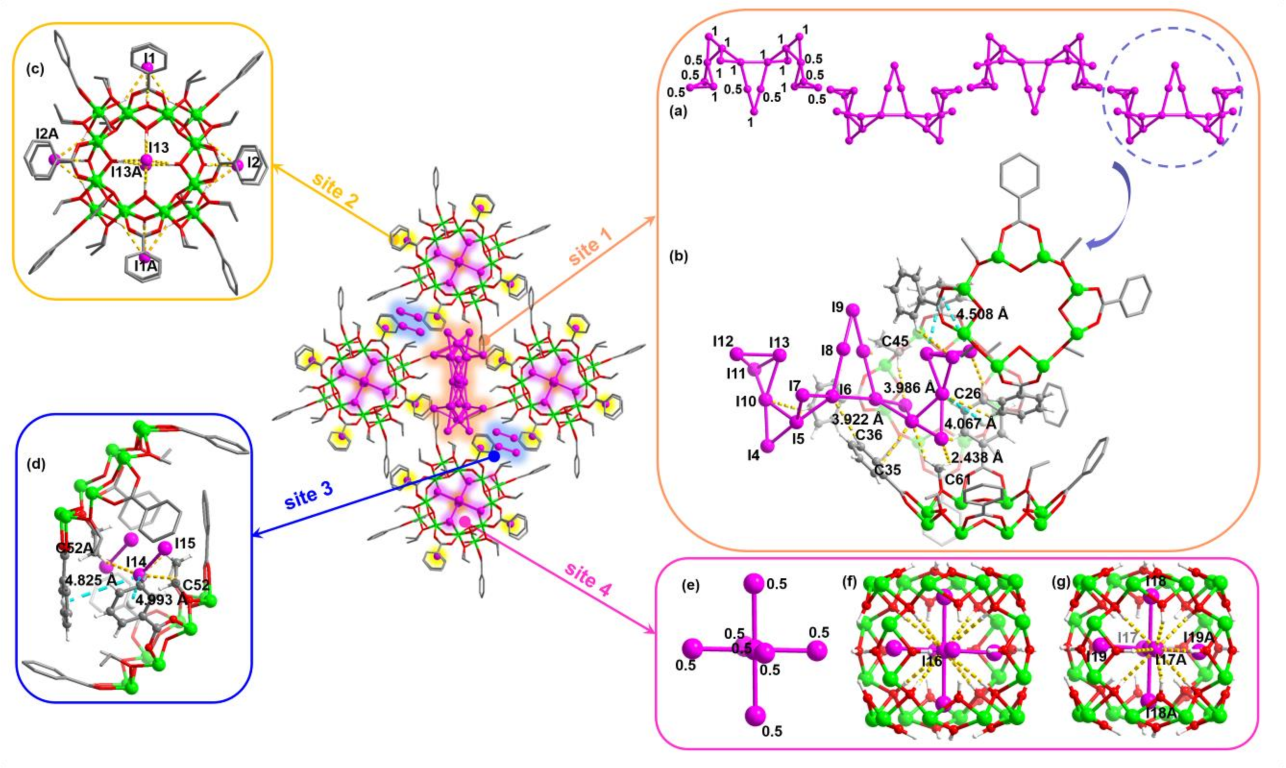

Supplementary Figure 131. The distributions and detailed interactions of iodine in **I@Al$_{24}$–100000ppm–48h** when **AlMC-1** is immersed in 100000 ppm aqueous I_2/KI solution for 48 h. (a) The occupies of iodine in the channels of **AlMC-1**; (b) the C–H···I hydrogen-bonding interactions and I···π interactions in site 1; (c) the O–H···I hydrogen-bond interactions in site 2; (d) the C–H···I hydrogen-bond interactions and I···π interactions in site 3; (e) the octahedral iodine species in site 4, and occupancy for each iodine atoms is 0.5; (f) the O–H···I hydrogen-bond interactions between central I16 atom and μ$_3$-OH groups; (g) the O–H···I hydrogen-bond interactions between iodine atoms located at the octahedral vertexes and μ$_3$-OH or μ$_2$-OH groups, I17A, I18A and I19A are generated through the symmetric operation.

The saturation point of absorption was gained by immersing crystals in high-concentration I_2/KI aqueous solution (300 mg KI, 300 mg I_2, 3 mL H_2O, about 100000 ppm) for 48h, and 26.5 I around each Al$_{24}$ cage can be clearly observed (denoted as: **I@Al$_{24}$–100000ppm–48h**). Intriguingly, besides site 1 to site 3 (Supplementary Figure 131a–131d), the inner Al$_{24}$ cage (site 4) can also capture I species (Supplementary Figure 131e), and octahedral iodine species can be resolved. Among them, 0.5 I (I16) is located at the body center of the Al$_{24}$ cage (Supplementary Figure 131f), and the other I ions are disordered at the inner side of the six square windows (I17, I17A, I18, I18A, I19 and I19A) (Supplementary Figure 131g) and further immobilized by μ$_2$-OH and μ$_3$-OH groups through strong O-H···I interactions.

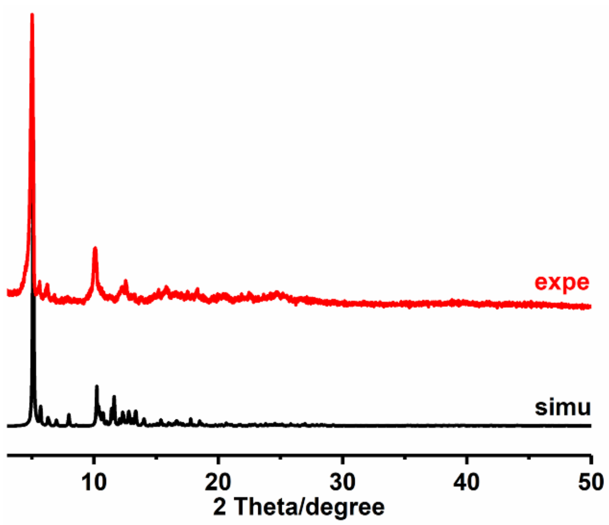

Supplementary Figure 132. The PXRD of the simulated and experimental patterns of **I@Al$_{24}$–4ppm–48h**.

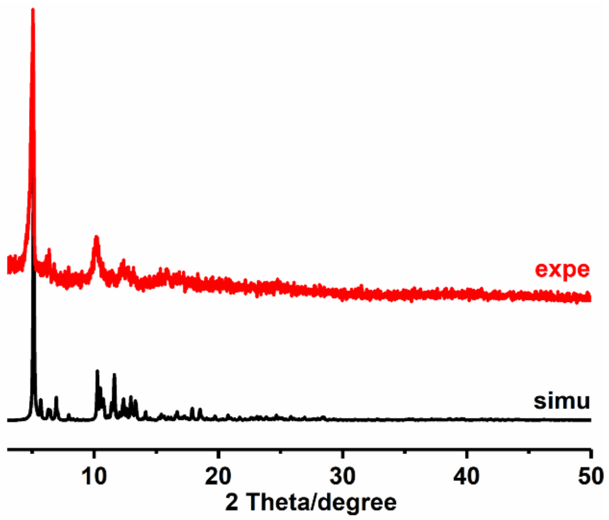

Supplementary Figure 133. The PXRD of the simulated and experimental patterns of **I@Al$_{24}$-–40ppm–48h**.

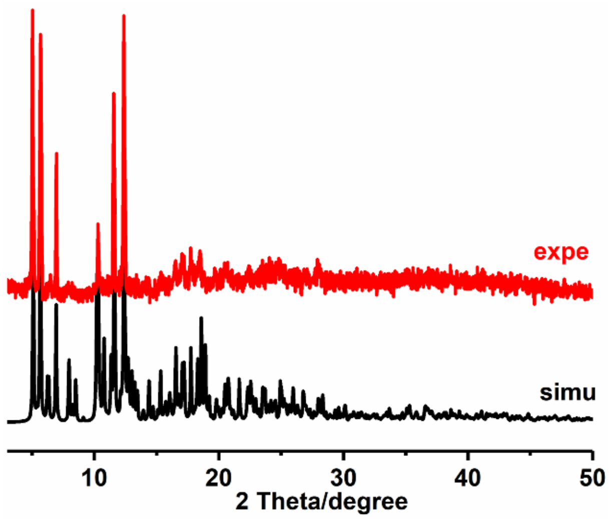

Supplementary Figure 134. The PXRD of the simulated and experimental patterns of **I@Al$_{24}$–400ppm–48h**.

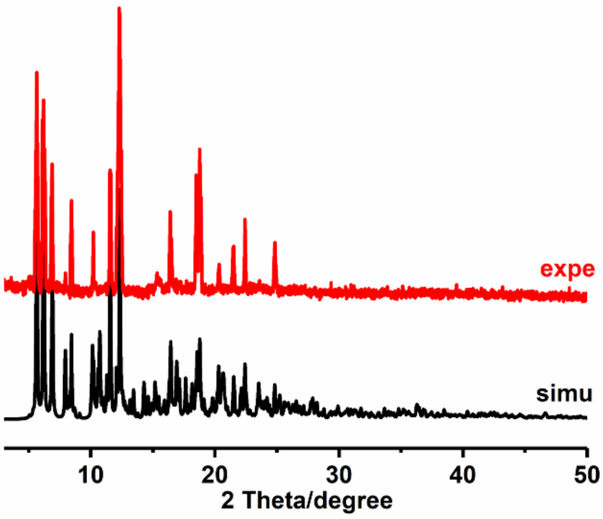

Supplementary Figure 135. The PXRD of the simulated and experimental patterns of **I@Al$_{24}$–2000ppm–48h**.

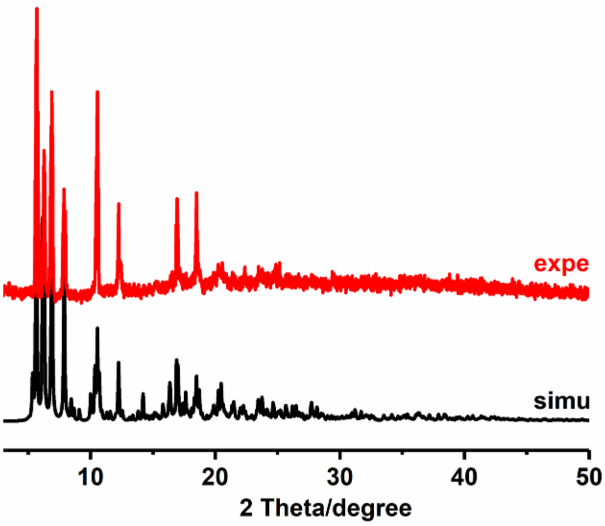

Supplementary Figure 136. The PXRD of the simulated and experimental patterns of **I@Al$_{24}$-–100000ppm–48h**.

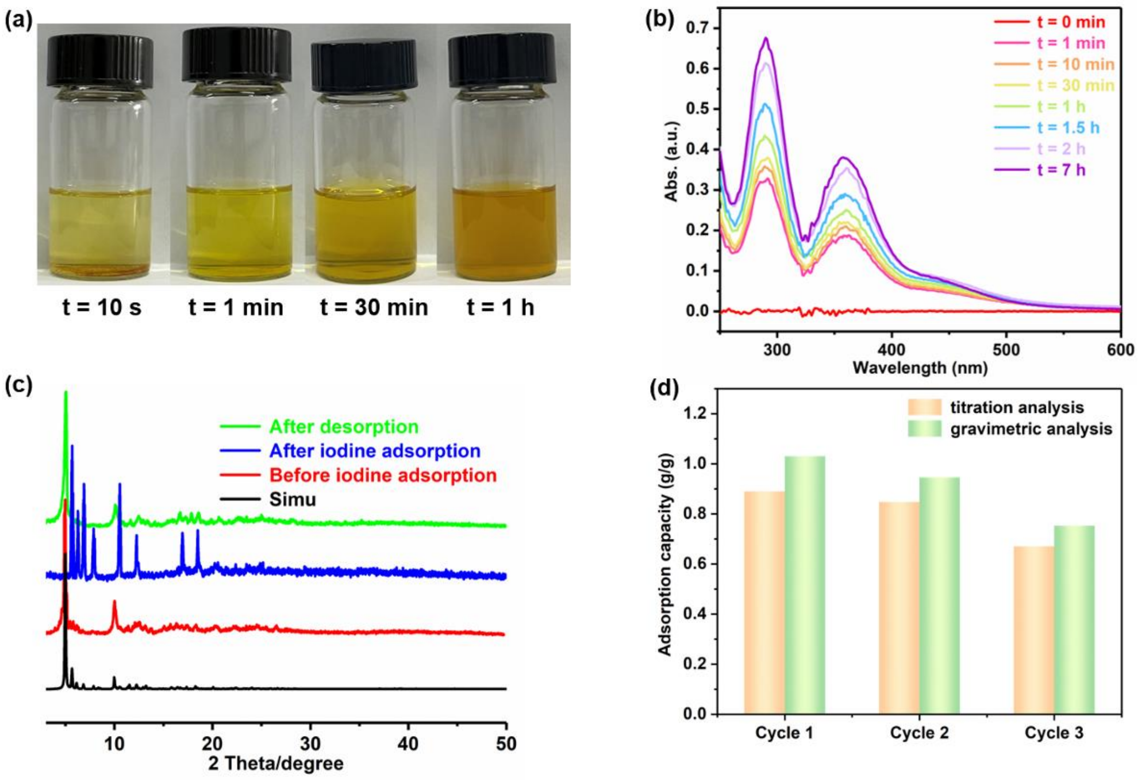

Supplementary Figure 137. Iodine release experiments. (a) Photos of the I-release process of **I@Al$_{24}$–100000ppm–48h** immersed in 10 mL HOEt. (b) Temporal evolution absorbance for the iodine release from 10 mL HOEt. (c) PXRD patterns of **AlMC-1** before iodine adsorption, after iodine adsorption and after desorption. (d) Recycling experiment.

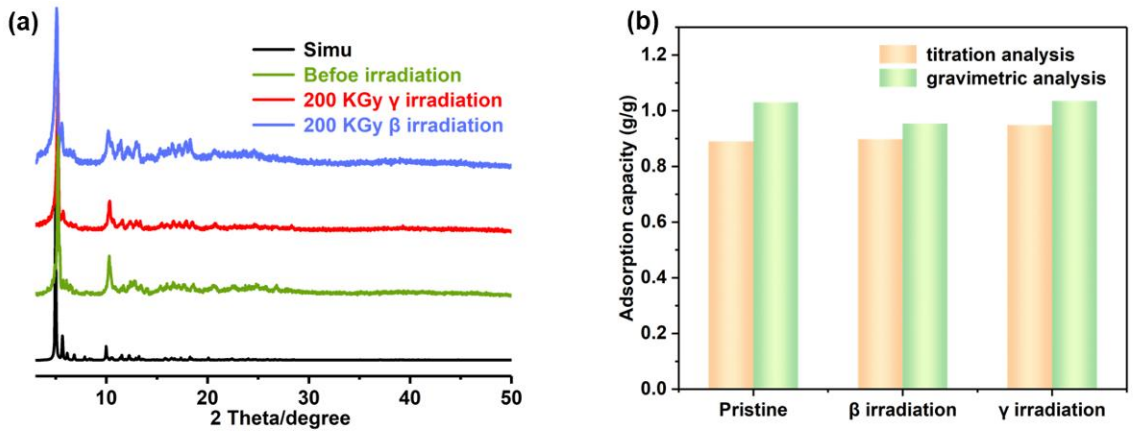

Supplementary Figure 138. Radiation resistance tests. (a) PXRD patterns of **AlMC-1** before and after β or γ irradiation. (b) Iodine adsorption capacity of **AlMC-1** before and after irradiation.

4. Supplementary Tables

4.1 The sizes of Al_8 macrocycle

Supplementary Table 1. The sizes of Al_8 macrocycle with different guests.

	guest type	Bottom Size (Å)	Top Size (Å)	Height (Å)
**AlMC-1**	H_2O	4.91	18.41	4.61
	NO_3^-	4.94	18.19	4.58
	OEt^-	4.92	16.83	5.25
**AlMC-2**	NO_3^-	4.97	18.06	4.72
	O^nPr^-	4.93	17.75	4.88
		4.91	17.73	4.90
**AlMC-3**	OEt^-	4.93	16.42	5.49
		4.94	18.28	4.60
		4.96	18.76	4.60
**AlMC-4**	Cl^-	4.96	17.32	5.09
		4.94	18.75	4.32
		4.94	17.16	5.19
		4.97	17.99	4.72
		4.96	17.18	5.15
		4.97	18.37	4.60
	average[a]	4.96	17.79	4.85
**AlMC-5**	Br^-	4.94	17.86	4.83
**AlMC-6**	I^-	4.98	17.85	4.80
**AlMC-7**	OEt^-	4.96	17.94	4.86

[a] Due to the disorder of the benzene rings in **AlMC-5** and its low molecular symmetry, we use the average values to estimate the size of Cl^- ions within the Al_8 macrocyclic units.

4.2 The solubility of AlMC-1 in different solvents

Supplementary Table 2. The solubility of **AlMC-1** in solvents with different polarity.

**solvent**	**polarity**	**solubleness [a]**
petroleum ether	0.01	insoluble
n-hexane	0.06	insoluble
cyclohexane	0.1	insoluble
methylbenzene	2.4	insoluble
dichloromethane	3.4	insoluble
n-butylalcohol	3.9	insoluble
n-propanol	4.0	insoluble
tetrahydrofuran	4.2	insoluble
ethanol	4.3	insoluble
ethyl acetate	4.3	insoluble
isopropanol	4.3	insoluble
dioxane	4.8	insoluble

acetone	5.4	insoluble
acetonitrile	6.2	soluble (solubility: ≈1mg/mL)
dimethylformamide	6.4	soluble (solubility: >1mg/mL)
methanol	6.6	insoluble
dimethylsulfoxide	7.2	soluble (solubility: >1mg/mL)

[a] The solubility was evaluated by soaking 1 mg of crystals into 2 mL of solvent for 5 minutes.

4.3 The ESI-MS data

Supplementary Table 3. The assignment of the key species for **AlMC-1** dissolved in DMSO under positive mode of ESI-MS tests. (Both calculated and experimental m/z values refer to the most intense peak in the isotopic envelope).

No.	Chemistry Formula	Cal. m/z	Exp. m/z
Species: $[Al_{24}(BA)_{12}(\mu_3\text{-}OH)_8(\mu_2\text{-}OH)_x(OEt)_{48\text{-}x}\cdot NO_3]^{3+}$ (x = 28, 29, ···, 38, 39)			
**1**	$[Al_{24}(BA)_{12}(\mu_3\text{-}OH)_8(\mu_2\text{-}OH)_{39}(OEt)_9\cdot NO_3]^{3+}$	1122.44	1122.44
**2**	$[Al_{24}(BA)_{12}(\mu_3\text{-}OH)_8(\mu_2\text{-}OH)_{38}(OEt)_{10}\cdot NO_3]^{3+}$	1131.79	1131.78
**3**	$[Al_{24}(BA)_{12}(\mu_3\text{-}OH)_8(\mu_2\text{-}OH)_{37}(OEt)_{11}\cdot NO_3]^{3+}$	1141.13	1141.12
**4**	$[Al_{24}(BA)_{12}(\mu_3\text{-}OH)_8(\mu_2\text{-}OH)_{36}(OEt)_{12}\cdot NO_3]^{3+}$	1150.47	1150.46
**5**	$[Al_{24}(BA)_{12}(\mu_3\text{-}OH)_8(\mu_2\text{-}OH)_{35}(OEt)_{13}\cdot NO_3]^{3+}$	1159.83	1159.81
**6**	$[Al_{24}(BA)_{12}(\mu_3\text{-}OH)_8(\mu_2\text{-}OH)_{34}(OEt)_{14}\cdot NO_3]^{3+}$	1169.16	1169.15
**7**	$[Al_{24}(BA)_{12}(\mu_3\text{-}OH)_8(\mu_2\text{-}OH)_{33}(OEt)_{15}\cdot NO_3]^{3+}$	1178.51	1178.50
**8**	$[Al_{24}(BA)_{12}(\mu_3\text{-}OH)_8(\mu_2\text{-}OH)_{32}(OEt)_{16}\cdot NO_3]^{3+}$	1187.85	1187.84
**9**	$[Al_{24}(BA)_{12}(\mu_3\text{-}OH)_8(\mu_2\text{-}OH)_{31}(OEt)_{17}\cdot NO_3]^{3+}$	1197.19	1197.18
**10**	$[Al_{24}(BA)_{12}(\mu_3\text{-}OH)_8(\mu_2\text{-}OH)_{30}(OEt)_{18}\cdot NO_3]^{3+}$	1206.54	1206.53
**11**	$[Al_{24}(BA)_{12}(\mu_3\text{-}OH)_8(\mu_2\text{-}OH)_{29}(OEt)_{19}\cdot NO_3]^{3+}$	1215.88	1215.87
**12**	$[Al_{24}(BA)_{12}(\mu_3\text{-}OH)_8(\mu_2\text{-}OH)_{28}(OEt)_{20}\cdot NO_3]^{3+}$	1225.22	1225.22
Species: $[Al_{24}(BA)_{12}(\mu_3\text{-}OH)_8(\mu_2\text{-}OH)_x(OEt)_{48\text{-}x}\cdot NO_3\cdot OEt\cdot DMSO\cdot 2H_2O]^{2+}$ (x = 2, 3, … , 16, 17)			
**1**	$[Al_{24}(BA)_{12}(\mu_3\text{-}OH)_8(\mu_2\text{-}OH)_{17}(OEt)_7\cdot NO_3\cdot OEt\cdot DMSO\cdot 2H_2O]^{2+}$	1735.17	1735.18
**2**	$[Al_{24}(BA)_{12}(\mu_3\text{-}OH)_8(\mu_2\text{-}OH)_{16}(OEt)_8\cdot NO_3\cdot OEt\cdot DMSO\cdot 2H_2O]^{2+}$	1749.18	1749.26
**3**	$[Al_{24}(BA)_{12}(\mu_3\text{-}OH)_8(\mu_2\text{-}OH)_{15}(OEt)_9\cdot NO_3\cdot OEt\cdot DMSO\cdot 2H_2O]^{2+}$	1763.20	1763.21
**4**	$[Al_{24}(BA)_{12}(\mu_3\text{-}OH)_8(\mu_2\text{-}OH)_{14}(OEt)_{10}\cdot NO_3\cdot OEt\cdot DMSO\cdot 2H_2O]^{2+}$	1777.22	1777.21
**5**	$[Al_{24}(BA)_{12}(\mu_3\text{-}OH)_8(\mu_2\text{-}OH)_{13}(OEt)_{11}\cdot NO_3\cdot OEt\cdot DMSO\cdot 2H_2O]^{2+}$	1791.23	1791.25
**6**	$[Al_{24}(BA)_{12}(\mu_3\text{-}OH)_8(\mu_2\text{-}OH)_{12}(OEt)_{12}\cdot NO_3\cdot OEt\cdot DMSO\cdot 2H_2O]^{2+}$	1805.25	1805.26
**7**	$[Al_{24}(BA)_{12}(\mu_3\text{-}OH)_8(\mu_2\text{-}OH)_{11}(OEt)_{13}\cdot NO_3\cdot OEt\cdot DMSO\cdot 2H_2O]^{2+}$	1819.26	1819.27

**8**	$[Al_{24}(BA)_{12}(\mu_3\text{-}OH)_8(\mu_2\text{-}OH)_{10}(OEt)_{14}\cdot NO_3\cdot OEt\cdot DMSO\cdot 2H_2O]^{2+}$	1833.29	1833.28
**9**	$[Al_{24}(BA)_{12}(\mu_3\text{-}OH)_8(\mu_2\text{-}OH)_9(OEt)_{15}\cdot NO_3\cdot OEt\cdot DMSO\cdot 2H_2O]^{2+}$	1847.29	1847.30
**10**	$[Al_{24}(BA)_{12}(\mu_3\text{-}OH)_8(\mu_2\text{-}OH)_8(OEt)_{16}\cdot NO_3\cdot OEt\cdot DMSO\cdot 2H_2O]^{2+}$	1861.31	1861.32
**11**	$[Al_{24}(BA)_{12}(\mu_3\text{-}OH)_8(\mu_2\text{-}OH)_7(OEt)_{17}\cdot NO_3\cdot OEt\cdot DMSO\cdot 2H_2O]^{2+}$	1875.32	1875.32
**12**	$[Al_{24}(BA)_{12}(\mu_3\text{-}OH)_8(\mu_2\text{-}OH)_6(OEt)_{18}\cdot NO_3\cdot OEt\cdot DMSO\cdot 2H_2O]^{2+}$	1889.34	1889.33
**13**	$[Al_{24}(BA)_{12}(\mu_3\text{-}OH)_8(\mu_2\text{-}OH)_5(OEt)_{19}\cdot NO_3\cdot OEt\cdot DMSO\cdot 2H_2O]^{2+}$	1903.36	1903.31
**14**	$[Al_{24}(BA)_{12}(\mu_3\text{-}OH)_8(\mu_2\text{-}OH)_4(OEt)_{20}\cdot NO_3\cdot OEt\cdot DMSO\cdot 2H_2O]^{2+}$	1917.37	1917.33
**15**	$[Al_{24}(BA)_{12}(\mu_3\text{-}OH)_8(\mu_2\text{-}OH)_3(OEt)_{21}\cdot NO_3\cdot OEt\cdot DMSO\cdot 2H_2O]^{2+}$	1931.39	1931.33
**16**	$[Al_{24}(BA)_{12}(\mu_3\text{-}OH)_8(\mu_2\text{-}OH)_2(OEt)_{22}\cdot NO_3\cdot OEt\cdot DMSO\cdot 2H_2O]^{2+}$	1945.40	1945.37
Species: $[Al_{24}(BA)_{12}(\mu_3\text{-}OH)_8(\mu_2\text{-}OH)_x(OEt)_{48-x}\cdot NO_3\cdot OEt\cdot 4CH_3CN\cdot 3DMSO\cdot 2H_2O]^{2+}$ (x = 37, 38, … , 43, 44)			
**1**	$[Al_{24}(BA)_{12}(\mu_3\text{-}OH)_8(\mu_2\text{-}OH)_{44}(OEt)_4\cdot NO_3\cdot OEt\cdot 4CH_3CN\cdot 3DMSO\cdot 2H_2O]^{2+}$	1853.19	1853.28
**2**	$[Al_{24}(BA)_{12}(\mu_3\text{-}OH)_8(\mu_2\text{-}OH)_{43}(OEt)_5\cdot NO_3\cdot OEt\cdot 4CH_3CN\cdot 3DMSO\cdot 2H_2O]^{2+}$	1867.20	1867.27
**3**	$[Al_{24}(BA)_{12}(\mu_3\text{-}OH)_8(\mu_2\text{-}OH)_{42}(OEt)_6\cdot NO_3\cdot OEt\cdot 4CH_3CN\cdot 3DMSO\cdot 2H_2O]^{2+}$	1881.22	1881.28
**4**	$[Al_{24}(BA)_{12}(\mu_3\text{-}OH)_8(\mu_2\text{-}OH)_{41}(OEt)_7\cdot NO_3\cdot OEt\cdot 4CH_3CN\cdot 3DMSO\cdot 2H_2O]^{2+}$	1895.24	1895.30
**5**	$[Al_{24}(BA)_{12}(\mu_3\text{-}OH)_8(\mu_2\text{-}OH)_{40}(OEt)_8\cdot NO_3\cdot OEt\cdot 4CH_3CN\cdot 3DMSO\cdot 2H_2O]^{2+}$	1909.25	1909.31
**6**	$[Al_{24}(BA)_{12}(\mu_3\text{-}OH)_8(\mu_2\text{-}OH)_{39}(OEt)_9\cdot NO_3\cdot OEt\cdot 4CH_3CN\cdot 3DMSO\cdot 2H_2O]^{2+}$	1923.27	1923.34
**7**	$[Al_{24}(BA)_{12}(\mu_3\text{-}OH)_8(\mu_2\text{-}OH)_{38}(OEt)_{10}\cdot NO_3\cdot OEt\cdot 4CH_3CN\cdot 3DMSO\cdot 2H_2O]^{2+}$	1937.28	1937.35
**8**	$[Al_{24}(BA)_{12}(\mu_3\text{-}OH)_8(\mu_2\text{-}OH)_{37}(OEt)_{11}\cdot NO_3\cdot OEt\cdot 4CH_3CN\cdot 3DMSO\cdot 2H_2O]^{2+}$	1951.30	1951.36
Species: $[Al_{24}(BA)_{12}(\mu_3\text{-}OH)_8(\mu_2\text{-}OH)_x(OEt)_{48-x}\cdot 2NO_3\cdot 2CH_3CN]^{2+}$ (x = 31, 32, … , 37, 38)			
**1**	$[Al_{24}(BA)_{12}(\mu_3\text{-}OH)_8(\mu_2\text{-}OH)_{38}(OEt)_{10}\cdot 2NO_3\cdot 2CH_3CN]^{2+}$	1769.70	1769.72
**2**	$[Al_{24}(BA)_{12}(\mu_3\text{-}OH)_8(\mu_2\text{-}OH)_{37}(OEt)_{11}\cdot 2NO_3\cdot 2CH_3CN]^{2+}$	1783.72	1783.73
**3**	$[Al_{24}(BA)_{12}(\mu_3\text{-}OH)_8(\mu_2\text{-}OH)_{36}(OEt)_{12}\cdot 2NO_3\cdot 2CH_3CN]^{2+}$	1797.73	1797.74
**4**	$[Al_{24}(BA)_{12}(\mu_3\text{-}OH)_8(\mu_2\text{-}OH)_{35}(OEt)_{13}\cdot 2NO_3\cdot 2CH_3CN]^{2+}$	1811.75	1811.77
**5**	$[Al_{24}(BA)_{12}(\mu_3\text{-}OH)_8(\mu_2\text{-}OH)_{34}(OEt)_{14}\cdot 2NO_3\cdot 2CH_3CN]^{2+}$	1825.76	1825.80
**6**	$[Al_{24}(BA)_{12}(\mu_3\text{-}OH)_8(\mu_2\text{-}OH)_{33}(OEt)_{15}\cdot 2NO_3\cdot 2CH_3CN]^{2+}$	1839.78	1839.80
**7**	$[Al_{24}(BA)_{12}(\mu_3\text{-}OH)_8(\mu_2\text{-}OH)_{32}(OEt)_{16}\cdot 2NO_3\cdot 2CH_3CN]^{2+}$	1853.79	1853.75
**8**	$[Al_{24}(BA)_{12}(\mu_3\text{-}OH)_8(\mu_2\text{-}OH)_{31}(OEt)_{17}\cdot 2NO_3\cdot 2CH_3CN]^{2+}$	1867.81	1867.77

Supplementary Table 4. The assignment of the key species for **AlMC-1** dissolved in MeCN under positive mode of ESI-MS tests. (Both calculated and experimental m/z values refer to the most intense peak in the isotopic envelope).

No.	Chemistry Formula	Cal. m/z	Exp. m/z

Species: $[Al_{24}(BA)_{12}(\mu_3\text{-}OH)_8(\mu_2\text{-}OH)_x(OEt)_{48\text{-}x}\cdot NO_3]^{3+}$ (x = 26, 27, ·…, 36, 37)			
**1**	$[Al_{24}(BA)_{12}(\mu_3\text{-}OH)_8(\mu_2\text{-}OH)_{37}(OEt)_{11}\cdot NO_3]^{3+}$	1141.13	1141.11
**2**	$[Al_{24}(BA)_{12}(\mu_3\text{-}OH)_8(\mu_2\text{-}OH)_{36}(OEt)_{12}\cdot NO_3]^{3+}$	1150.47	1150.45
**3**	$[Al_{24}(BA)_{12}(\mu_3\text{-}OH)_8(\mu_2\text{-}OH)_{35}(OEt)_{13}\cdot NO_3]^{3+}$	1159.82	1159.80
**4**	$[Al_{24}(BA)_{12}(\mu_3\text{-}OH)_8(\mu_2\text{-}OH)_{34}(OEt)_{14}\cdot NO_3]^{3+}$	1169.16	1169.14
**5**	$[Al_{24}(BA)_{12}(\mu_3\text{-}OH)_8(\mu_2\text{-}OH)_{33}(OEt)_{15}\cdot NO_3]^{3+}$	1178.51	1178.48
**6**	$[Al_{24}(BA)_{12}(\mu_3\text{-}OH)_8(\mu_2\text{-}OH)_{32}(OEt)_{16}\cdot NO_3]^{3+}$	1187.85	1187.83
**7**	$[Al_{24}(BA)_{12}(\mu_3\text{-}OH)_8(\mu_2\text{-}OH)_{31}(OEt)_{17}\cdot NO_3]^{3+}$	1197.19	1197.17
**8**	$[Al_{24}(BA)_{12}(\mu_3\text{-}OH)_8(\mu_2\text{-}OH)_{30}(OEt)_{18}\cdot NO_3]^{3+}$	1206.54	1206.52
**9**	$[Al_{24}(BA)_{12}(\mu_3\text{-}OH)_8(\mu_2\text{-}OH)_{29}(OEt)_{19}\cdot NO_3]^{3+}$	1215.88	1215.86
**10**	$[Al_{24}(BA)_{12}(\mu_3\text{-}OH)_8(\mu_2\text{-}OH)_{28}(OEt)_{20}\cdot NO_3]^{3+}$	1225.22	1225.20
**11**	$[Al_{24}(BA)_{12}(\mu_3\text{-}OH)_8(\mu_2\text{-}OH)_{27}(OEt)_{21}\cdot NO_3]^{3+}$	1234.57	1234.54
**12**	$[Al_{24}(BA)_{12}(\mu_3\text{-}OH)_8(\mu_2\text{-}OH)_{26}(OEt)_{22}\cdot NO_3]^{3+}$	1243.91	1243.89
Species: $[Al_{24}(BA)_{12}(\mu_3\text{-}OH)_8(\mu_2\text{-}OH)_x(OEt)_{48\text{-}x}\cdot 2NO_3\cdot 4MeCN\cdot 4H_2O]^{2+}$ (x = 31, 32, … , 43, 44)			
**1**	$[Al_{24}(BA)_{12}(\mu_3\text{-}OH)_8(\mu_2\text{-}OH)_{44}(OEt)_4\cdot 2NO_3\cdot 4CH_3CN\cdot 4H_2O]^{2+}$	1762.65	1762.69
**2**	$[Al_{24}(BA)_{12}(\mu_3\text{-}OH)_8(\mu_2\text{-}OH)_{43}(OEt)_5\cdot 2NO_3\cdot 4CH_3CN\cdot 4H_2O]^{2+}$	1776.67	1776.71
**3**	$[Al_{24}(BA)_{12}(\mu_3\text{-}OH)_8(\mu_2\text{-}OH)_{42}(OEt)_6\cdot 2NO_3\cdot 4CH_3CN\cdot 4H_2O]^{2+}$	1790.69	1790.73
**4**	$[Al_{24}(BA)_{12}(\mu_3\text{-}OH)_8(\mu_2\text{-}OH)_{41}(OEt)_7\cdot 2NO_3\cdot 4CH_3CN\cdot 4H_2O]^{2+}$	1804.70	1804.74
**5**	$[Al_{24}(BA)_{12}(\mu_3\text{-}OH)_8(\mu_2\text{-}OH)_{40}(OEt)_8\cdot 2NO_3\cdot 4CH_3CN\cdot 4H_2O]^{2+}$	1818.72	1818.76
**6**	$[Al_{24}(BA)_{12}(\mu_3\text{-}OH)_8(\mu_2\text{-}OH)_{39}(OEt)_9\cdot 2NO_3\cdot 4CH_3CN\cdot 4H_2O]^{2+}$	1832.73	1832.77
**7**	$[Al_{24}(BA)_{12}(\mu_3\text{-}OH)_8(\mu_2\text{-}OH)_{38}(OEt)_{10}\cdot 2NO_3\cdot 4CH_3CN\cdot 4H_2O]^{2+}$	1846.75	1846.79
**8**	$[Al_{24}(BA)_{12}(\mu_3\text{-}OH)_8(\mu_2\text{-}OH)_{37}(OEt)_{11}\cdot 2NO_3\cdot 4CH_3CN\cdot 4H_2O]^{2+}$	1860.76	1860.81
**9**	$[Al_{24}(BA)_{12}(\mu_3\text{-}OH)_8(\mu_2\text{-}OH)_{36}(OEt)_{12}\cdot 2NO_3\cdot 4CH_3CN\cdot 4H_2O]^{2+}$	1874.78	1874.82
**10**	$[Al_{24}(BA)_{12}(\mu_3\text{-}OH)_8(\mu_2\text{-}OH)_{35}(OEt)_{13}\cdot 2NO_3\cdot 4CH_3CN\cdot 4H_2O]^{2+}$	1888.80	1888.83
**11**	$[Al_{24}(BA)_{12}(\mu_3\text{-}OH)_8(\mu_2\text{-}OH)_{34}(OEt)_{14}\cdot 2NO_3\cdot 4CH_3CN\cdot 4H_2O]^{2+}$	1902.81	1902.84
**12**	$[Al_{24}(BA)_{12}(\mu_3\text{-}OH)_8(\mu_2\text{-}OH)_{33}(OEt)_{15}\cdot 2NO_3\cdot 4CH_3CN\cdot 4H_2O]^{2+}$	1916.83	1916.85
**13**	$[Al_{24}(BA)_{12}(\mu_3\text{-}OH)_8(\mu_2\text{-}OH)_{32}(OEt)_{16}\cdot 2NO_3\cdot 4CH_3CN\cdot 4H_2O]^{2+}$	1930.84	1930.85
**14**	$[Al_{24}(BA)_{12}(\mu_3\text{-}OH)_8(\mu_2\text{-}OH)_{31}(OEt)_{17}\cdot 2NO_3\cdot 4CH_3CN\cdot 4H_2O]^{2+}$	1944.86	1944.86
Species: $[Al_{24}(BA)_{12}(\mu_3\text{-}OH)_8(\mu_2\text{-}OH)_x(OEt)_{48\text{-}x}\cdot NO_3\cdot OEt\cdot 5CH_3CN\cdot 2H_2O]^{2+}$ (x = 31, 32, … , 39, 40)			
**1**	$[Al_{24}(BA)_{12}(\mu_3\text{-}OH)_8(\mu_2\text{-}OH)_{40}(OEt)_8\cdot NO_3\cdot OEt\cdot 5CH_3CN\cdot 2H_2O]^{2+}$	1812.75	1812.74
**2**	$[Al_{24}(BA)_{12}(\mu_3\text{-}OH)_8(\mu_2\text{-}OH)_{39}(OEt)_9\cdot NO_3\cdot OEt\cdot 5CH_3CN\cdot 2H_2O]^{2+}$	1826.76	1826.76
**3**	$[Al_{24}(BA)_{12}(\mu_3\text{-}OH)_8(\mu_2\text{-}OH)_{38}(OEt)_{10}\cdot NO_3\cdot OEt\cdot 5CH_3CN\cdot 2H_2O]^{2+}$	1840.78	1840.77

**4**	$[Al_{24}(BA)_{12}(\mu_3\text{-}OH)_8(\mu_2\text{-}OH)_{37}(OEt)_{11}\cdot NO_3\cdot OEt\cdot 5CH_3CN\cdot 2H_2O]^{2+}$	1854.79	1854.79
**5**	$[Al_{24}(BA)_{12}(\mu_3\text{-}OH)_8(\mu_2\text{-}OH)_{36}(OEt)_{12}\cdot NO_3\cdot OEt\cdot 5CH_3CN\cdot 2H_2O]^{2+}$	1868.81	1868.80
**6**	$[Al_{24}(BA)_{12}(\mu_3\text{-}OH)_8(\mu_2\text{-}OH)_{35}(OEt)_{13}\cdot NO_3\cdot OEt\cdot 5CH_3CN\cdot 2H_2O]^{2+}$	1882.82	1882.82
**7**	$[Al_{24}(BA)_{12}(\mu_3\text{-}OH)_8(\mu_2\text{-}OH)_{34}(OEt)_{14}\cdot NO_3\cdot OEt\cdot 5CH_3CN\cdot 2H_2O]^{2+}$	1896.84	1896.83
**8**	$[Al_{24}(BA)_{12}(\mu_3\text{-}OH)_8(\mu_2\text{-}OH)_{33}(OEt)_{15}\cdot NO_3\cdot OEt\cdot 5CH_3CN\cdot 2H_2O]^{2+}$	1910.84	1910.85
**9**	$[Al_{24}(BA)_{12}(\mu_3\text{-}OH)_8(\mu_2\text{-}OH)_{32}(OEt)_{16}\cdot NO_3\cdot OEt\cdot 5CH_3CN\cdot 2H_2O]^{2+}$	1924.87	1924.83
**10**	$[Al_{24}(BA)_{12}(\mu_3\text{-}OH)_8(\mu_2\text{-}OH)_{31}(OEt)_{17}\cdot NO_3\cdot OEt\cdot 5CH_3CN\cdot 2H_2O]^{2+}$	1938.88	1938.83
Species: $[Al_{24}(BA)_{12}(\mu_3\text{-}OH)_8(\mu_2\text{-}OH)_x(OEt)_{48\text{-}x}\cdot NO_3\cdot OEt\cdot 4CH_3CN\cdot H_2O]^{2+}$ (x = 29, 30, … , 38, 39)			
**1**	$[Al_{24}(BA)_{12}(\mu_3\text{-}OH)_8(\mu_2\text{-}OH)_{39}(OEt)_9\cdot NO_3\cdot OEt\cdot 4CH_3CN\cdot H_2O]^{2+}$	1797.23	1797.24
**2**	$[Al_{24}(BA)_{12}(\mu_3\text{-}OH)_8(\mu_2\text{-}OH)_{38}(OEt)_{10}\cdot NO_3\cdot OEt\cdot 4CH_3CN\cdot H_2O]^{2+}$	1811.25	1811.26
**3**	$[Al_{24}(BA)_{12}(\mu_3\text{-}OH)_8(\mu_2\text{-}OH)_{37}(OEt)_{11}\cdot NO_3\cdot OEt\cdot 4CH_3CN\cdot H_2O]^{2+}$	1825.27	1825.27
**4**	$[Al_{24}(BA)_{12}(\mu_3\text{-}OH)_8(\mu_2\text{-}OH)_{36}(OEt)_{12}\cdot NO_3\cdot OEt\cdot 4CH_3CN\cdot H_2O]^{2+}$	1839.28	1839.29
**5**	$[Al_{24}(BA)_{12}(\mu_3\text{-}OH)_8(\mu_2\text{-}OH)_{35}(OEt)_{13}\cdot NO_3\cdot OEt\cdot 4CH_3CN\cdot H_2O]^{2+}$	1853.30	1853.30
**6**	$[Al_{24}(BA)_{12}(\mu_3\text{-}OH)_8(\mu_2\text{-}OH)_{34}(OEt)_{14}\cdot NO_3\cdot OEt\cdot 4CH_3CN\cdot H_2O]^{2+}$	1867.31	1867.32
**7**	$[Al_{24}(BA)_{12}(\mu_3\text{-}OH)_8(\mu_2\text{-}OH)_{33}(OEt)_{15}\cdot NO_3\cdot OEt\cdot 4CH_3CN\cdot H_2O]^{2+}$	1881.32	1881.33
**8**	$[Al_{24}(BA)_{12}(\mu_3\text{-}OH)_8(\mu_2\text{-}OH)_{32}(OEt)_{16}\cdot NO_3\cdot OEt\cdot 4CH_3CN\cdot H_2O]^{2+}$	1895.31	1895.35
**9**	$[Al_{24}(BA)_{12}(\mu_3\text{-}OH)_8(\mu_2\text{-}OH)_{31}(OEt)_{17}\cdot NO_3\cdot OEt\cdot 4CH_3CN\cdot H_2O]^{2+}$	1909.37	1909.31
**10**	$[Al_{24}(BA)_{12}(\mu_3\text{-}OH)_8(\mu_2\text{-}OH)_{30}(OEt)_{18}\cdot NO_3\cdot OEt\cdot 4CH_3CN\cdot H_2O]^{2+}$	1923.38	1923.32
**11**	$[Al_{24}(BA)_{12}(\mu_3\text{-}OH)_8(\mu_2\text{-}OH)_{29}(OEt)_{19}\cdot NO_3\cdot OEt\cdot 4CH_3CN\cdot H_2O]^{2+}$	1937.40	1937.34
Species: $[Al_{24}(BA)_{12}(\mu_3\text{-}OH)_8(\mu_2\text{-}OH)_x(OEt)_{48\text{-}x}\cdot 2NO_3\cdot 9CH_3CN\cdot 2H_2O]^{2+}$ (x = 27, 28, … , 37, 38)			
**1**	$[Al_{24}(BA)_{12}(\mu_3\text{-}OH)_8(\mu_2\text{-}OH)_{38}(OEt)_{10}\cdot 2NO_3\cdot 9CH_3CN\cdot 2H_2O]^{2+}$	1931.30	1931.33
**2**	$[Al_{24}(BA)_{12}(\mu_3\text{-}OH)_8(\mu_2\text{-}OH)_{37}(OEt)_{11}\cdot 2NO_3\cdot 9CH_3CN\cdot 2H_2O]^{2+}$	1945.32	1945.36
**3**	$[Al_{24}(BA)_{12}(\mu_3\text{-}OH)_8(\mu_2\text{-}OH)_{36}(OEt)_{12}\cdot 2NO_3\cdot 9CH_3CN\cdot 2H_2O]^{2+}$	1959.34	1959.39
**4**	$[Al_{24}(BA)_{12}(\mu_3\text{-}OH)_8(\mu_2\text{-}OH)_{35}(OEt)_{13}\cdot 2NO_3\cdot 9CH_3CN\cdot 2H_2O]^{2+}$	1973.35	1973.40
**5**	$[Al_{24}(BA)_{12}(\mu_3\text{-}OH)_8(\mu_2\text{-}OH)_{34}(OEt)_{14}\cdot 2NO_3\cdot 9CH_3CN\cdot 2H_2O]^{2+}$	1987.37	1987.42
**6**	$[Al_{24}(BA)_{12}(\mu_3\text{-}OH)_8(\mu_2\text{-}OH)_{33}(OEt)_{15}\cdot 2NO_3\cdot 9CH_3CN\cdot 2H_2O]^{2+}$	2001.38	2001.44
**7**	$[Al_{24}(BA)_{12}(\mu_3\text{-}OH)_8(\mu_2\text{-}OH)_{32}(OEt)_{16}\cdot 2NO_3\cdot 9CH_3CN\cdot 2H_2O]^{2+}$	2015.40	2015.45
**8**	$[Al_{24}(BA)_{12}(\mu_3\text{-}OH)_8(\mu_2\text{-}OH)_{31}(OEt)_{17}\cdot 2NO_3\cdot 9CH_3CN\cdot 2H_2O]^{2+}$	2029.41	2029.47
**9**	$[Al_{24}(BA)_{12}(\mu_3\text{-}OH)_8(\mu_2\text{-}OH)_{30}(OEt)_{18}\cdot 2NO_3\cdot 9CH_3CN\cdot 2H_2O]^{2+}$	2043.43	2043.48
**10**	$[Al_{24}(BA)_{12}(\mu_3\text{-}OH)_8(\mu_2\text{-}OH)_{29}(OEt)_{19}\cdot 2NO_3\cdot 9CH_3CN\cdot 2H_2O]^{2+}$	2057.45	2057.48
**11**	$[Al_{24}(BA)_{12}(\mu_3\text{-}OH)_8(\mu_2\text{-}OH)_{28}(OEt)_{20}\cdot 2NO_3\cdot 9CH_3CN\cdot 2H_2O]^{2+}$	2071.46	2071.50
**12**	$[Al_{24}(BA)_{12}(\mu_3\text{-}OH)_8(\mu_2\text{-}OH)_{27}(OEt)_{21}\cdot 2NO_3\cdot 9CH_3CN\cdot 2H_2O]^{2+}$	2085.48	2085.50

Species: $[Al_{24}(BA)_{12}(\mu_3\text{-}OH)_8(\mu_2\text{-}OH)_x(OEt)_{48\text{-}x}\cdot NO_3\cdot OEt\cdot 8CH_3CN\cdot 3H_2O]^{2+}$ (*x* = 26, 27, … , 33, 34)			
**1**	$[Al_{24}(BA)_{12}(\mu_3\text{-}OH)_8(\mu_2\text{-}OH)_{34}(OEt)_{14}\cdot NO_3\cdot OEt\cdot 8CH_3CN\cdot 3H_2O]^{2+}$	1967.38	1967.39
**2**	$[Al_{24}(BA)_{12}(\mu_3\text{-}OH)_8(\mu_2\text{-}OH)_{33}(OEt)_{15}\cdot NO_3\cdot OEt\cdot 8CH_3CN\cdot 3H_2O]^{2+}$	1981.40	1981.42
**3**	$[Al_{24}(BA)_{12}(\mu_3\text{-}OH)_8(\mu_2\text{-}OH)_{32}(OEt)_{16}\cdot NO_3\cdot OEt\cdot 8CH_3CN\cdot 3H_2O]^{2+}$	1995.41	1995.42
**4**	$[Al_{24}(BA)_{12}(\mu_3\text{-}OH)_8(\mu_2\text{-}OH)_{31}(OEt)_{17}\cdot NO_3\cdot OEt\cdot 8CH_3CN\cdot 3H_2O]^{2+}$	2009.43	2009.44
**5**	$[Al_{24}(BA)_{12}(\mu_3\text{-}OH)_8(\mu_2\text{-}OH)_{30}(OEt)_{18}\cdot NO_3\cdot OEt\cdot 8CH_3CN\cdot 3H_2O]^{2+}$	2023.44	2023.45
**6**	$[Al_{24}(BA)_{12}(\mu_3\text{-}OH)_8(\mu_2\text{-}OH)_{29}(OEt)_{19}\cdot NO_3\cdot OEt\cdot 8CH_3CN\cdot 3H_2O]^{2+}$	2037.46	2037.47
**7**	$[Al_{24}(BA)_{12}(\mu_3\text{-}OH)_8(\mu_2\text{-}OH)_{28}(OEt)_{20}\cdot NO_3\cdot OEt\cdot 8CH_3CN\cdot 3H_2O]^{2+}$	2051.48	2051.46
**8**	$[Al_{24}(BA)_{12}(\mu_3\text{-}OH)_8(\mu_2\text{-}OH)_{27}(OEt)_{21}\cdot NO_3\cdot OEt\cdot 8CH_3CN\cdot 3H_2O]^{2+}$	2065.49	2065.44
**9**	$[Al_{24}(BA)_{12}(\mu_3\text{-}OH)_8(\mu_2\text{-}OH)_{26}(OEt)_{22}\cdot NO_3\cdot OEt\cdot 8CH_3CN\cdot 3H_2O]^{2+}$	2079.51	2079.49
Species: $[Al_{24}(BA)_{12}(\mu_3\text{-}OH)_8(\mu_2\text{-}OH)_x(OEt)_{48\text{-}x}\cdot 2NO_3\cdot 14CH_3CN]^{2+}$ (*x* = 24, 25, … , 30, 31)			
**1**	$[Al_{24}(BA)_{12}(\mu_3\text{-}OH)_8(\mu_2\text{-}OH)_{31}(OEt)_{17}\cdot 2NO_3\cdot 14CH_3CN]^{2+}$	2113.97	2114.03
**2**	$[Al_{24}(BA)_{12}(\mu_3\text{-}OH)_8(\mu_2\text{-}OH)_{30}(OEt)_{18}\cdot 2NO_3\cdot 14CH_3CN]^{2+}$	2127.99	2128.04
**3**	$[Al_{24}(BA)_{12}(\mu_3\text{-}OH)_8(\mu_2\text{-}OH)_{29}(OEt)_{19}\cdot 2NO_3\cdot 14CH_3CN]^{2+}$	2142.00	2142.08
**4**	$[Al_{24}(BA)_{12}(\mu_3\text{-}OH)_8(\mu_2\text{-}OH)_{28}(OEt)_{20}\cdot 2NO_3\cdot 14CH_3CN]^{2+}$	2156.02	2156.08
**5**	$[Al_{24}(BA)_{12}(\mu_3\text{-}OH)_8(\mu_2\text{-}OH)_{27}(OEt)_{21}\cdot 2NO_3\cdot 14CH_3CN]^{2+}$	2170.03	2170.10
**6**	$[Al_{24}(BA)_{12}(\mu_3\text{-}OH)_8(\mu_2\text{-}OH)_{26}(OEt)_{22}\cdot 2NO_3\cdot 14CH_3CN]^{2+}$	2184.05	2184.12
**7**	$[Al_{24}(BA)_{12}(\mu_3\text{-}OH)_8(\mu_2\text{-}OH)_{25}(OEt)_{23}\cdot 2NO_3\cdot 14CH_3CN]^{2+}$	2198.57	2198.63
**8**	$[Al_{24}(BA)_{12}(\mu_3\text{-}OH)_8(\mu_2\text{-}OH)_{24}(OEt)_{24}\cdot 2NO_3\cdot 14CH_3CN]^{2+}$	2212.58	2212.64

Supplementary Table 5. The assignment of the key species for **AlMC-2** dissolved in MeCN under the positive mode of ESI-MS tests. (Both calculated and experimental m/z values refer to the most intense peak in the isotopic envelope).

No.	Chemistry Formula	Cal. m/z	Exp. m/z
Species: $[Al_{24}(BA)_{12}(\mu_3\text{-}OH)_8(\mu_2\text{-}OH)_x(O^nPr)_{48\text{-}x}\cdot NO_3]^{3+}$ (x = 27, 28, ·…, 41, 42)			
**1**	$[Al_{24}(BA)_{12}(\mu_3\text{-}OH)_8(\mu_2\text{-}OH)_{42}(O^nPr)_6\cdot NO_3]^{3+}$	1122.44	1122.42
**2**	$[Al_{24}(BA)_{12}(\mu_3\text{-}OH)_8(\mu_2\text{-}OH)_{41}(O^nPr)_7\cdot NO_3]^{3+}$	1136.46	1136.43
**3**	$[Al_{24}(BA)_{12}(\mu_3\text{-}OH)_8(\mu_2\text{-}OH)_{40}(O^nPr)_8\cdot NO_3]^{3+}$	1150.47	1150.45
**4**	$[Al_{24}(BA)_{12}(\mu_3\text{-}OH)_8(\mu_2\text{-}OH)_{39}(O^nPr)_9\cdot NO_3]^{3+}$	1164.49	1164.47
**5**	$[Al_{24}(BA)_{12}(\mu_3\text{-}OH)_8(\mu_2\text{-}OH)_{38}(O^nPr)_{10}\cdot NO_3]^{3+}$	1178.51	1178.48
**6**	$[Al_{24}(BA)_{12}(\mu_3\text{-}OH)_8(\mu_2\text{-}OH)_{37}(O^nPr)_{11}\cdot NO_3]^{3+}$	1192.52	1192.49
**7**	$[Al_{24}(BA)_{12}(\mu_3\text{-}OH)_8(\mu_2\text{-}OH)_{36}(O^nPr)_{12}\cdot NO_3]^{3+}$	1206.54	1206.51

**8**	$[Al_{24}(BA)_{12}(\mu_3\text{-}OH)_8(\mu_2\text{-}OH)_{35}(O^nPr)_{13}\cdot NO_3]^{3+}$	1220.55	1220.53
**9**	$[Al_{24}(BA)_{12}(\mu_3\text{-}OH)_8(\mu_2\text{-}OH)_{34}(O^nPr)_{14}\cdot NO_3]^{3+}$	1234.57	1234.54
**10**	$[Al_{24}(BA)_{12}(\mu_3\text{-}OH)_8(\mu_2\text{-}OH)_{33}(O^nPr)_{15}\cdot NO_3]^{3+}$	1248.58	1248.56
**11**	$[Al_{24}(BA)_{12}(\mu_3\text{-}OH)_8(\mu_2\text{-}OH)_{32}(O^nPr)_{16}\cdot NO_3]^{3+}$	1262.60	1262.57
**12**	$[Al_{24}(BA)_{12}(\mu_3\text{-}OH)_8(\mu_2\text{-}OH)_{31}(O^nPr)_{17}\cdot NO_3]^{3+}$	1276.62	1276.59
**13**	$[Al_{24}(BA)_{12}(\mu_3\text{-}OH)_8(\mu_2\text{-}OH)_{30}(O^nPr)_{18}\cdot NO_3]^{3+}$	1290.63	1290.60
**14**	$[Al_{24}(BA)_{12}(\mu_3\text{-}OH)_8(\mu_2\text{-}OH)_{29}(O^nPr)_{19}\cdot NO_3]^{3+}$	1304.65	1304.62
**15**	$[Al_{24}(BA)_{12}(\mu_3\text{-}OH)_8(\mu_2\text{-}OH)_{28}(O^nPr)_{20}\cdot NO_3]^{3+}$	1318.66	1318.63
**16**	$[Al_{24}(BA)_{12}(\mu_3\text{-}OH)_8(\mu_2\text{-}OH)_{27}(O^nPr)_{21}\cdot NO_3]^{3+}$	1332.68	1332.64
Species: $[Al_{24}(BA)_{12}(\mu_3\text{-}OH)_8(\mu_2\text{-}OH)_x(O^nPr)_{48\text{-}x}\cdot NO_3\cdot 11CH_3CN\cdot 3H_2O]^{3+}$ (x = 32, 33, ·…, 38, 39)			
**1**	$[Al_{24}(BA)_{12}(\mu_3\text{-}OH)_8(\mu_2\text{-}OH)_{39}(O^nPr)_9\cdot NO_3\cdot 11CH_3CN\cdot 3H_2O]^{3+}$	1332.93	1332.98
**2**	$[Al_{24}(BA)_{12}(\mu_3\text{-}OH)_8(\mu_2\text{-}OH)_{38}(O^nPr)_{10}\cdot NO_3\cdot 11CH_3CN\cdot 3H_2O]^{3+}$	1346.95	1346.99
**3**	$[Al_{24}(BA)_{12}(\mu_3\text{-}OH)_8(\mu_2\text{-}OH)_{37}(O^nPr)_{11}\cdot NO_3\cdot 11CH_3CN\cdot 3H_2O]^{3+}$	1360.96	1361.00
**4**	$[Al_{24}(BA)_{12}(\mu_3\text{-}OH)_8(\mu_2\text{-}OH)_{36}(O^nPr)_{12}\cdot NO_3\cdot 11CH_3CN\cdot 3H_2O]^{3+}$	1374.98	1375.01
**5**	$[Al_{24}(BA)_{12}(\mu_3\text{-}OH)_8(\mu_2\text{-}OH)_{35}(O^nPr)_{13}\cdot NO_3\cdot 11CH_3CN\cdot 3H_2O]^{3+}$	1388.99	1389.03
**6**	$[Al_{24}(BA)_{12}(\mu_3\text{-}OH)_8(\mu_2\text{-}OH)_{34}(O^nPr)_{14}\cdot NO_3\cdot 11CH_3CN\cdot 3H_2O]^{3+}$	1403.01	1403.04
**7**	$[Al_{24}(BA)_{12}(\mu_3\text{-}OH)_8(\mu_2\text{-}OH)_{33}(O^nPr)_{15}\cdot NO_3\cdot 11CH_3CN\cdot 3H_2O]^{3+}$	1417.03	1417.06
**8**	$[Al_{24}(BA)_{12}(\mu_3\text{-}OH)_8(\mu_2\text{-}OH)_{32}(O^nPr)_{16}\cdot NO_3\cdot 11CH_3CN\cdot 3H_2O]^{3+}$	1431.04	1431.07
Species: $[Al_{24}(BA)_{12}(\mu_3\text{-}OH)_8(\mu_2\text{-}OH)_x(O^nPr)_{48\text{-}x}\cdot NO_3\cdot 10CH_3CN\cdot 3H_2O]^{3+}$ (*x* = 24, 25, … , 29, 30)			
**1**	$[Al_{24}(BA)_{12}(\mu_3\text{-}OH)_8(\mu_2\text{-}OH)_{30}(O^nPr)_{18}\cdot NO_3\cdot 10CH_3CN\cdot 3H_2O]^{3+}$	1445.40	1445.41
**2**	$[Al_{24}(BA)_{12}(\mu_3\text{-}OH)_8(\mu_2\text{-}OH)_{29}(O^nPr)_{19}\cdot NO_3\cdot 10CH_3CN\cdot 3H_2O]^{3+}$	1459.40	1459.42
**3**	$[Al_{24}(BA)_{12}(\mu_3\text{-}OH)_8(\mu_2\text{-}OH)_{28}(O^nPr)_{20}\cdot NO_3\cdot 10CH_3CN\cdot 3H_2O]^{3+}$	1473.43	1473.44
**4**	$[Al_{24}(BA)_{12}(\mu_3\text{-}OH)_8(\mu_2\text{-}OH)_{27}(O^nPr)_{21}\cdot NO_3\cdot 10CH_3CN\cdot 3H_2O]^{3+}$	1488.09	1488.12
**5**	$[Al_{24}(BA)_{12}(\mu_3\text{-}OH)_8(\mu_2\text{-}OH)_{26}(O^nPr)_{22}\cdot NO_3\cdot 10CH_3CN\cdot 3H_2O]^{3+}$	1501.79	1501.80
**6**	$[Al_{24}(BA)_{12}(\mu_3\text{-}OH)_8(\mu_2\text{-}OH)_{25}(O^nPr)_{23}\cdot NO_3\cdot 10CH_3CN\cdot 3H_2O]^{3+}$	1515.81	1515.82
**7**	$[Al_{24}(BA)_{12}(\mu_3\text{-}OH)_8(\mu_2\text{-}OH)_{24}(O^nPr)_{24}\cdot NO_3\cdot 10CH_3CN\cdot 3H_2O]^{3+}$	1529.49	1529.50
Species: $[Al_{24}(BA)_{12}(\mu_3\text{-}OH)_8(\mu_2\text{-}OH)_x(O^nPr)_{48\text{-}x}\cdot NO_3\cdot 4CH_3CN\cdot 4H_2O]^{3+}$ (*x* = 33, 34, … , 47, 48)			
**1**	$[Al_{24}(BA)_{12}(\mu_3\text{-}OH)_8(\mu_2\text{-}OH)_{48}\cdot NO_3\cdot 4CH_3CN\cdot 4H_2O]^{3+}$	1117.07	1117.04
**2**	$[Al_{24}(BA)_{12}(\mu_3\text{-}OH)_8(\mu_2\text{-}OH)_{47}(O^nPr)_1\cdot NO_3\cdot 4CH_3CN\cdot 4H_2O]^{3+}$	1131.08	1131.10
**3**	$[Al_{24}(BA)_{12}(\mu_3\text{-}OH)_8(\mu_2\text{-}OH)_{46}(O^nPr)_2\cdot NO_3\cdot 4CH_3CN\cdot 4H_2O]^{3+}$	1145.10	1145.07
**4**	$[Al_{24}(BA)_{12}(\mu_3\text{-}OH)_8(\mu_2\text{-}OH)_{45}(O^nPr)_3\cdot NO_3\cdot 4CH_3CN\cdot 4H_2O]^{3+}$	1159.11	1159.17

**5**	$[Al_{24}(BA)_{12}(\mu_3\text{-}OH)_8(\mu_2\text{-}OH)_{44}(O^nPr)_4\cdot NO_3\cdot 4CH_3CN\cdot 4H_2O]^{3+}$	1173.13	1173.30
**6**	$[Al_{24}(BA)_{12}(\mu_3\text{-}OH)_8(\mu_2\text{-}OH)_{43}(O^nPr)_5\cdot NO_3\cdot 4CH_3CN\cdot 4H_2O]^{3+}$	1187.14	1187.17
**7**	$[Al_{24}(BA)_{12}(\mu_3\text{-}OH)_8(\mu_2\text{-}OH)_{42}(O^nPr)_6\cdot NO_3\cdot 4CH_3CN\cdot 4H_2O]^{3+}$	1201.16	1201.13
**8**	$[Al_{24}(BA)_{12}(\mu_3\text{-}OH)_8(\mu_2\text{-}OH)_{41}(O^nPr)_7\cdot NO_3\cdot 4CH_3CN\cdot 4H_2O]^{3+}$	1215.17	1215.19
**9**	$[Al_{24}(BA)_{12}(\mu_3\text{-}OH)_8(\mu_2\text{-}OH)_{40}(O^nPr)_8\cdot NO_3\cdot 4CH_3CN\cdot 4H_2O]^{3+}$	1229.19	1229.16
**10**	$[Al_{24}(BA)_{12}(\mu_3\text{-}OH)_8(\mu_2\text{-}OH)_{39}(O^nPr)_9\cdot NO_3\cdot 4CH_3CN\cdot 4H_2O]^{3+}$	1243.21	1243.22
**11**	$[Al_{24}(BA)_{12}(\mu_3\text{-}OH)_8(\mu_2\text{-}OH)_{38}(O^nPr)_{10}\cdot NO_3\cdot 4CH_3CN\cdot 4H_2O]^{3+}$	1257.22	1257.24
**12**	$[Al_{24}(BA)_{12}(\mu_3\text{-}OH)_8(\mu_2\text{-}OH)_{37}(O^nPr)_{11}\cdot NO_3\cdot 4CH_3CN\cdot 4H_2O]^{3+}$	1217.24	1217.17
**13**	$[Al_{24}(BA)_{12}(\mu_3\text{-}OH)_8(\mu_2\text{-}OH)_{36}(O^nPr)_{12}\cdot NO_3\cdot 4CH_3CN\cdot 4H_2O]^{3+}$	1285.25	1285.23
**14**	$[Al_{24}(BA)_{12}(\mu_3\text{-}OH)_8(\mu_2\text{-}OH)_{35}(O^nPr)_{13}\cdot NO_3\cdot 4CH_3CN\cdot 4H_2O]^{3+}$	1299.27	1299.29
**15**	$[Al_{24}(BA)_{12}(\mu_3\text{-}OH)_8(\mu_2\text{-}OH)_{34}(O^nPr)_{14}\cdot NO_3\cdot 4CH_3CN\cdot 4H_2O]^{3+}$	1313.28	1313.24
**16**	$[Al_{24}(BA)_{12}(\mu_3\text{-}OH)_8(\mu_2\text{-}OH)_{33}(O^nPr)_{15}\cdot NO_3\cdot 4CH_3CN\cdot 4H_2O]^{3+}$	1327.30	1327.29
Species: $[Al_{24}(BA)_{12}(\mu_3\text{-}OH)_8(\mu_2\text{-}OH)_x(O^nPr)_{48\text{-}x}\cdot NO_3\cdot O^nPr\cdot 9CH_3CN\cdot 3H_2O]^{2+}$ (x = 36, 37, … , 47, 48)			
**1**	$[Al_{24}(BA)_{12}(\mu_3\text{-}OH)_8(\mu_2\text{-}OH)_{48}\cdot NO_3\cdot O^nPr\cdot 9CH_3CN\cdot 3H_2O]^{2+}$	1798.68	1798.71
**2**	$[Al_{24}(BA)_{12}(\mu_3\text{-}OH)_8(\mu_2\text{-}OH)_{47}(O^nPr)_1\cdot NO_3\cdot O^nPr\cdot 9CH_3CN\cdot 3H_2O]^{2+}$	1819.74	1819.73
**3**	$[Al_{24}(BA)_{12}(\mu_3\text{-}OH)_8(\mu_2\text{-}OH)_{46}(O^nPr)_2\cdot NO_3\cdot O^nPr\cdot 9CH_3CN\cdot 3H_2O]^{2+}$	1840.73	1840.76
**4**	$[Al_{24}(BA)_{12}(\mu_3\text{-}OH)_8(\mu_2\text{-}OH)_{45}(O^nPr)_3\cdot NO_3\cdot O^nPr\cdot 9CH_3CN\cdot 3H_2O]^{2+}$	1861.75	1861.78
**5**	$[Al_{24}(BA)_{12}(\mu_3\text{-}OH)_8(\mu_2\text{-}OH)_{44}(O^nPr)_4\cdot NO_3\cdot O^nPr\cdot 9CH_3CN\cdot 3H_2O]^{2+}$	1882.78	1882.81
**6**	$[Al_{24}(BA)_{12}(\mu_3\text{-}OH)_8(\mu_2\text{-}OH)_{43}(O^nPr)_5\cdot NO_3\cdot O^nPr\cdot 9CH_3CN\cdot 3H_2O]^{2+}$	1903.80	1903.83
**7**	$[Al_{24}(BA)_{12}(\mu_3\text{-}OH)_8(\mu_2\text{-}OH)_{42}(O^nPr)_6\cdot NO_3\cdot O^nPr\cdot 9CH_3CN\cdot 3H_2O]^{2+}$	1924.82	1924.85
**8**	$[Al_{24}(BA)_{12}(\mu_3\text{-}OH)_8(\mu_2\text{-}OH)_{41}(O^nPr)_7\cdot NO_3\cdot O^nPr\cdot 9CH_3CN\cdot 3H_2O]^{2+}$	1945.85	1945.87
**9**	$[Al_{24}(BA)_{12}(\mu_3\text{-}OH)_8(\mu_2\text{-}OH)_{40}(O^nPr)_8\cdot NO_3\cdot O^nPr\cdot 9CH_3CN\cdot 3H_2O]^{2+}$	1966.87	1966.90
**10**	$[Al_{24}(BA)_{12}(\mu_3\text{-}OH)_8(\mu_2\text{-}OH)_{39}(O^nPr)_9\cdot NO_3\cdot O^nPr\cdot 9CH_3CN\cdot 3H_2O]^{2+}$	1987.90	1987.92
**11**	$[Al_{24}(BA)_{12}(\mu_3\text{-}OH)_8(\mu_2\text{-}OH)_{38}(O^nPr)_{10}\cdot NO_3\cdot O^nPr\cdot 9CH_3CN\cdot 3H_2O]^{2+}$	2008.92	2008.94
**12**	$[Al_{24}(BA)_{12}(\mu_3\text{-}OH)_8(\mu_2\text{-}OH)_{37}(O^nPr)_{11}\cdot NO_3\cdot O^nPr\cdot 9CH_3CN\cdot 3H_2O]^{2+}$	2029.94	2029.96
**13**	$[Al_{24}(BA)_{12}(\mu_3\text{-}OH)_8(\mu_2\text{-}OH)_{36}(O^nPr)_{12}\cdot NO_3\cdot O^nPr\cdot 9CH_3CN\cdot 3H_2O]^{2+}$	2050.97	2050.99
Species: $[Al_{24}(BA)_{12}(\mu_3\text{-}OH)_8(\mu_2\text{-}OH)_x(O^nPr)_{48\text{-}x}\cdot NO_3\cdot O^nPr\cdot 8CH_3CN\cdot 3H_2O]^{2+}$ (x = 26, 27, … , 37, 38)			
**1**	$[Al_{24}(BA)_{12}(\mu_3\text{-}OH)_8(\mu_2\text{-}OH)_{38}(O^nPr)_{10}\cdot NO_3\cdot O^nPr\cdot 8CH_3CN\cdot 3H_2O]^{2+}$	2072.96	2072.99
**2**	$[Al_{24}(BA)_{12}(\mu_3\text{-}OH)_8(\mu_2\text{-}OH)_{37}(O^nPr)_{11}\cdot NO_3\cdot O^nPr\cdot 8CH_3CN\cdot 3H_2O]^{2+}$	2093.98	2094.01
**3**	$[Al_{24}(BA)_{12}(\mu_3\text{-}OH)_8(\mu_2\text{-}OH)_{36}(O^nPr)_{12}\cdot NO_3\cdot O^nPr\cdot 8CH_3CN\cdot 3H_2O]^{2+}$	2115.01	2115.04
**4**	$[Al_{24}(BA)_{12}(\mu_3\text{-}OH)_8(\mu_2\text{-}OH)_{35}(O^nPr)_{13}\cdot NO_3\cdot O^nPr\cdot 8CH_3CN\cdot 3H_2O]^{2+}$	2136.03	2136.06

**5**	$[Al_{24}(BA)_{12}(\mu_3\text{-}OH)_8(\mu_2\text{-}OH)_{34}(O^nPr)_{14}\cdot NO_3\cdot O^nPr\cdot 8CH_3CN\cdot 3H_2O]^{2+}$	2157.06	2157.08
**6**	$[Al_{24}(BA)_{12}(\mu_3\text{-}OH)_8(\mu_2\text{-}OH)_{33}(O^nPr)_{15}\cdot NO_3\cdot O^nPr\cdot 8CH_3CN\cdot 3H_2O]^{2+}$	2178.08	2178.10
**7**	$[Al_{24}(BA)_{12}(\mu_3\text{-}OH)_8(\mu_2\text{-}OH)_{32}(O^nPr)_{16}\cdot NO_3\cdot O^nPr\cdot 8CH_3CN\cdot 3H_2O]^{2+}$	2199.60	2199.61
**8**	$[Al_{24}(BA)_{12}(\mu_3\text{-}OH)_8(\mu_2\text{-}OH)_{31}(O^nPr)_{17}\cdot NO_3\cdot O^nPr\cdot 8CH_3CN\cdot 3H_2O]^{2+}$	2220.12	2220.13
**9**	$[Al_{24}(BA)_{12}(\mu_3\text{-}OH)_8(\mu_2\text{-}OH)_{30}(O^nPr)_{18}\cdot NO_3\cdot O^nPr\cdot 8CH_3CN\cdot 3H_2O]^{2+}$	2241.65	2241.65
**10**	$[Al_{24}(BA)_{12}(\mu_3\text{-}OH)_8(\mu_2\text{-}OH)_{29}(O^nPr)_{19}\cdot NO_3\cdot O^nPr\cdot 8CH_3CN\cdot 3H_2O]^{2+}$	2262.67	2262.68
**11**	$[Al_{24}(BA)_{12}(\mu_3\text{-}OH)_8(\mu_2\text{-}OH)_{28}(O^nPr)_{20}\cdot NO_3\cdot O^nPr\cdot 8CH_3CN\cdot 3H_2O]^{2+}$	2283.70	2283.70
**12**	$[Al_{24}(BA)_{12}(\mu_3\text{-}OH)_8(\mu_2\text{-}OH)_{27}(O^nPr)_{21}\cdot NO_3\cdot O^nPr\cdot 8CH_3CN\cdot 3H_2O]^{2+}$	2304.72	2304.72
**13**	$[Al_{24}(BA)_{12}(\mu_3\text{-}OH)_8(\mu_2\text{-}OH)_{26}(O^nPr)_{22}\cdot NO_3\cdot O^nPr\cdot 8CH_3CN\cdot 3H_2O]^{2+}$	2325.74	2325.75
Species: $[Al_{24}(BA)_{12}(\mu_3\text{-}OH)_8(\mu_2\text{-}OH)_x(O^nPr)_{48\text{-}x}\cdot 2NO_3\cdot 2CH_3CN\cdot H_2O]^{2+}$ (x = 29, 30, … , 41, 42)			
**1**	$[Al_{24}(BA)_{12}(\mu_3\text{-}OH)_8(\mu_2\text{-}OH)_{42}(O^nPr)_6\cdot 2NO_3\cdot 2CH_3CN\cdot H_2O]^{2+}$	1764.69	1764.68
**2**	$[Al_{24}(BA)_{12}(\mu_3\text{-}OH)_8(\mu_2\text{-}OH)_{41}(O^nPr)_7\cdot 2NO_3\cdot 2CH_3CN\cdot H_2O]^{2+}$	1785.71	1785.71
**3**	$[Al_{24}(BA)_{12}(\mu_3\text{-}OH)_8(\mu_2\text{-}OH)_{40}(O^nPr)_8\cdot 2NO_3\cdot 2CH_3CN\cdot H_2O]^{2+}$	1806.74	1806.72
**4**	$[Al_{24}(BA)_{12}(\mu_3\text{-}OH)_8(\mu_2\text{-}OH)_{39}(O^nPr)_9\cdot 2NO_3\cdot 2CH_3CN\cdot H_2O]^{2+}$	1827.76	1827.75
**5**	$[Al_{24}(BA)_{12}(\mu_3\text{-}OH)_8(\mu_2\text{-}OH)_{38}(O^nPr)_{10}\cdot 2NO_3\cdot 2CH_3CN\cdot H_2O]^{2+}$	1848.78	1848.77
**6**	$[Al_{24}(BA)_{12}(\mu_3\text{-}OH)_8(\mu_2\text{-}OH)_{37}(O^nPr)_{11}\cdot 2NO_3\cdot 2CH_3CN\cdot H_2O]^{2+}$	1869.81	1869.79
**7**	$[Al_{24}(BA)_{12}(\mu_3\text{-}OH)_8(\mu_2\text{-}OH)_{36}(O^nPr)_{12}\cdot 2NO_3\cdot 2CH_3CN\cdot H_2O]^{2+}$	1890.83	1890.82
**8**	$[Al_{24}(BA)_{12}(\mu_3\text{-}OH)_8(\mu_2\text{-}OH)_{35}(O^nPr)_{13}\cdot 2NO_3\cdot 2CH_3CN\cdot H_2O]^{2+}$	1911.85	1911.84
**9**	$[Al_{24}(BA)_{12}(\mu_3\text{-}OH)_8(\mu_2\text{-}OH)_{34}(O^nPr)_{14}\cdot 2NO_3\cdot 2CH_3CN\cdot H_2O]^{2+}$	1932.88	1932.86
**10**	$[Al_{24}(BA)_{12}(\mu_3\text{-}OH)_8(\mu_2\text{-}OH)_{33}(O^nPr)_{15}\cdot 2NO_3\cdot 2CH_3CN\cdot H_2O]^{2+}$	1953.90	1953.89
**11**	$[Al_{24}(BA)_{12}(\mu_3\text{-}OH)_8(\mu_2\text{-}OH)_{32}(O^nPr)_{16}\cdot 2NO_3\cdot 2CH_3CN\cdot H_2O]^{2+}$	1974.93	1974.91
**12**	$[Al_{24}(BA)_{12}(\mu_3\text{-}OH)_8(\mu_2\text{-}OH)_{31}(O^nPr)_{17}\cdot 2NO_3\cdot 2CH_3CN\cdot H_2O]^{2+}$	1995.95	1995.93
**13**	$[Al_{24}(BA)_{12}(\mu_3\text{-}OH)_8(\mu_2\text{-}OH)_{30}(O^nPr)_{18}\cdot 2NO_3\cdot 2CH_3CN\cdot H_2O]^{2+}$	2016.97	2016.95
**14**	$[Al_{24}(BA)_{12}(\mu_3\text{-}OH)_8(\mu_2\text{-}OH)_{29}(O^nPr)_{19}\cdot 2NO_3\cdot 2CH_3CN\cdot H_2O]^{2+}$	2038.00	2037.98
Species: $[Al_{24}(BA)_{12}(\mu_3\text{-}OH)_8(\mu_2\text{-}OH)_x(O^nPr)_{48\text{-}x}\cdot NO_3\cdot O^nPr\cdot 10CH_3CN\cdot 4H_2O]^{2+}$ (x = 32, 33, … , 36, 37)			
**1**	$[Al_{24}(BA)_{12}(\mu_3\text{-}OH)_8(\mu_2\text{-}OH)_{37}(O^nPr)_{11}\cdot NO_3\cdot O^nPr\cdot 10CH_3CN\cdot 4H_2O]^{2+}$	2059.46	2059.48
**2**	$[Al_{24}(BA)_{12}(\mu_3\text{-}OH)_8(\mu_2\text{-}OH)_{36}(O^nPr)_{12}\cdot NO_3\cdot O^nPr\cdot 10CH_3CN\cdot 4H_2O]^{2+}$	2080.48	2080.51
**3**	$[Al_{24}(BA)_{12}(\mu_3\text{-}OH)_8(\mu_2\text{-}OH)_{35}(O^nPr)_{13}\cdot NO_3\cdot O^nPr\cdot 10CH_3CN\cdot 4H_2O]^{2+}$	2101.51	2101.53
**4**	$[Al_{24}(BA)_{12}(\mu_3\text{-}OH)_8(\mu_2\text{-}OH)_{34}(O^nPr)_{14}\cdot NO_3\cdot O^nPr\cdot 10CH_3CN\cdot 4H_2O]^{2+}$	2122.53	2122.54
**5**	$[Al_{24}(BA)_{12}(\mu_3\text{-}OH)_8(\mu_2\text{-}OH)_{33}(O^nPr)_{15}\cdot NO_3\cdot O^nPr\cdot 10CH_3CN\cdot 4H_2O]^{2+}$	2143.55	2143.57
**6**	$[Al_{24}(BA)_{12}(\mu_3\text{-}OH)_8(\mu_2\text{-}OH)_{32}(O^nPr)_{16}\cdot NO_3\cdot O^nPr\cdot 10CH_3CN\cdot 4H_2O]^{2+}$	2164.58	2164.60
Species: $[Al_{24}(BA)_{12}(\mu_3\text{-}OH)_8(\mu_2\text{-}OH)_x(O^nPr)_{48\text{-}x}\cdot 2NO_3\cdot 9CH_3CN\cdot H_2O]^{2+}$ (x = 29, 30, … , 46, 47)			

**1**	$[Al_{24}(BA)_{12}(\mu_3\text{-}OH)_8(\mu_2\text{-}OH)_{47}(O^nPr)_1\cdot 2NO_3\cdot 9CH_3CN\cdot H_2O]^{2+}$	1803.17	1803.18
**2**	$[Al_{24}(BA)_{12}(\mu_3\text{-}OH)_8(\mu_2\text{-}OH)_{46}(O^nPr)_2\cdot 2NO_3\cdot 9CH_3CN\cdot H_2O]^{2+}$	1824.19	1824.21
**3**	$[Al_{24}(BA)_{12}(\mu_3\text{-}OH)_8(\mu_2\text{-}OH)_{45}(O^nPr)_3\cdot 2NO_3\cdot 9CH_3CN\cdot H_2O]^{2+}$	1845.21	1845.24
**4**	$[Al_{24}(BA)_{12}(\mu_3\text{-}OH)_8(\mu_2\text{-}OH)_{44}(O^nPr)_4\cdot 2NO_3\cdot 9CH_3CN\cdot H_2O]^{2+}$	1866.24	1866.26
**5**	$[Al_{24}(BA)_{12}(\mu_3\text{-}OH)_8(\mu_2\text{-}OH)_{43}(O^nPr)_5\cdot 2NO_3\cdot 9CH_3CN\cdot H_2O]^{2+}$	1887.26	1887.28
**6**	$[Al_{24}(BA)_{12}(\mu_3\text{-}OH)_8(\mu_2\text{-}OH)_{42}(O^nPr)_6\cdot 2NO_3\cdot 9CH_3CN\cdot H_2O]^{2+}$	1908.28	1908.30
**7**	$[Al_{24}(BA)_{12}(\mu_3\text{-}OH)_8(\mu_2\text{-}OH)_{41}(O^nPr)_7\cdot 2NO_3\cdot 9CH_3CN\cdot H_2O]^{2+}$	1929.31	1929.32
**8**	$[Al_{24}(BA)_{12}(\mu_3\text{-}OH)_8(\mu_2\text{-}OH)_{40}(O^nPr)_8\cdot 2NO_3\cdot 9CH_3CN\cdot H_2O]^{2+}$	1950.33	1950.35
**9**	$[Al_{24}(BA)_{12}(\mu_3\text{-}OH)_8(\mu_2\text{-}OH)_{39}(O^nPr)_9\cdot 2NO_3\cdot 9CH_3CN\cdot H_2O]^{2+}$	1971.35	1971.37
**10**	$[Al_{24}(BA)_{12}(\mu_3\text{-}OH)_8(\mu_2\text{-}OH)_{38}(O^nPr)_{10}\cdot 2NO_3\cdot 9CH_3CN\cdot H_2O]^{2+}$	1992.38	1992.40
**11**	$[Al_{24}(BA)_{12}(\mu_3\text{-}OH)_8(\mu_2\text{-}OH)_{37}(O^nPr)_{11}\cdot 2NO_3\cdot 9CH_3CN\cdot H_2O]^{2+}$	2013.40	2013.42
**12**	$[Al_{24}(BA)_{12}(\mu_3\text{-}OH)_8(\mu_2\text{-}OH)_{36}(O^nPr)_{12}\cdot 2NO_3\cdot 9CH_3CN\cdot H_2O]^{2+}$	2034.42	2034.44
**13**	$[Al_{24}(BA)_{12}(\mu_3\text{-}OH)_8(\mu_2\text{-}OH)_{35}(O^nPr)_{13}\cdot 2NO_3\cdot 9CH_3CN\cdot H_2O]^{2+}$	2055.45	2055.46
**14**	$[Al_{24}(BA)_{12}(\mu_3\text{-}OH)_8(\mu_2\text{-}OH)_{34}(O^nPr)_{14}\cdot 2NO_3\cdot 9CH_3CN\cdot H_2O]^{2+}$	2076.97	2076.97
**15**	$[Al_{24}(BA)_{12}(\mu_3\text{-}OH)_8(\mu_2\text{-}OH)_{33}(O^nPr)_{15}\cdot 2NO_3\cdot 9CH_3CN\cdot H_2O]^{2+}$	2097.49	2097.48
**16**	$[Al_{24}(BA)_{12}(\mu_3\text{-}OH)_8(\mu_2\text{-}OH)_{32}(O^nPr)_{16}\cdot 2NO_3\cdot 9CH_3CN\cdot H_2O]^{2+}$	2119.01	2119.00
**17**	$[Al_{24}(BA)_{12}(\mu_3\text{-}OH)_8(\mu_2\text{-}OH)_{31}(O^nPr)_{17}\cdot 2NO_3\cdot 9CH_3CN\cdot H_2O]^{2+}$	2140.04	2140.03
**18**	$[Al_{24}(BA)_{12}(\mu_3\text{-}OH)_8(\mu_2\text{-}OH)_{30}(O^nPr)_{18}\cdot 2NO_3\cdot 9CH_3CN\cdot H_2O]^{2+}$	2161.06	2161.05
**19**	$[Al_{24}(BA)_{12}(\mu_3\text{-}OH)_8(\mu_2\text{-}OH)_{29}(O^nPr)_{19}\cdot 2NO_3\cdot 9CH_3CN\cdot H_2O]^{2+}$	2182.09	2182.08

Supplementary Table 6. The assignment of the key species for **AlMC-3** dissolved in MeCN under positive mode of ESI-MS tests. (Both calculated and experimental m/z values refer to the most intense peak in the isotopic envelope).

No.	Chemistry Formula	Cal. m/z	Exp. m/z
Species: $[Al_{24}(BA)_{12}(\mu_3\text{-}OH)_8(\mu_2\text{-}OH)_x(OEt)_{48\text{-}x}\cdot NO_3\cdot 6CH_3CN\cdot H_2O]^{3+}$ (x = 30, 31, ·…, 38, 39)			
**1**	$[Al_{24}(BA)_{12}(\mu_3\text{-}OH)_8(\mu_2\text{-}OH)_{39}(OEt)_9\cdot NO_3\cdot 6CH_3CN\cdot H_2O]^{3+}$	1210.50	1210.48
**2**	$[Al_{24}(BA)_{12}(\mu_3\text{-}OH)_8(\mu_2\text{-}OH)_{38}(OEt)_{10}\cdot NO_3\cdot 6CH_3CN\cdot H_2O]^{3+}$	1219.84	1219.82
**3**	$[Al_{24}(BA)_{12}(\mu_3\text{-}OH)_8(\mu_2\text{-}OH)_{37}(OEt)_{11}\cdot NO_3\cdot 6CH_3CN\cdot H_2O]^{3+}$	1229.19	1229.20
**4**	$[Al_{24}(BA)_{12}(\mu_3\text{-}OH)_8(\mu_2\text{-}OH)_{36}(OEt)_{12}\cdot NO_3\cdot 6CH_3CN\cdot H_2O]^{3+}$	1238.53	1238.51
**5**	$[Al_{24}(BA)_{12}(\mu_3\text{-}OH)_8(\mu_2\text{-}OH)_{35}(OEt)_{13}\cdot NO_3\cdot 6CH_3CN\cdot H_2O]^{3+}$	1247.87	1247.85
**6**	$[Al_{24}(BA)_{12}(\mu_3\text{-}OH)_8(\mu_2\text{-}OH)_{34}(OEt)_{14}\cdot NO_3\cdot 6CH_3CN\cdot H_2O]^{3+}$	1257.22	1257.19
**7**	$[Al_{24}(BA)_{12}(\mu_3\text{-}OH)_8(\mu_2\text{-}OH)_{33}(OEt)_{15}\cdot NO_3\cdot 6CH_3CN\cdot H_2O]^{3+}$	1266.56	1266.53
**8**	$[Al_{24}(BA)_{12}(\mu_3\text{-}OH)_8(\mu_2\text{-}OH)_{32}(OEt)_{16}\cdot NO_3\cdot 6CH_3CN\cdot H_2O]^{3+}$	1275.91	1275.88

**9**	$[Al_{24}(BA)_{12}(\mu_3\text{-}OH)_8(\mu_2\text{-}OH)_{31}(OEt)_{17}\cdot NO_3\cdot 6CH_3CN\cdot H_2O]^{3+}$	1285.25	1285.22
**10**	$[Al_{24}(BA)_{12}(\mu_3\text{-}OH)_8(\mu_2\text{-}OH)_{30}(OEt)_{18}\cdot NO_3\cdot 6CH_3CN\cdot H_2O]^{3+}$	1294.59	1294.57
Species: $[Al_{24}(BA)_{12}(\mu_3\text{-}OH)_8(\mu_2\text{-}OH)_x(OEt)_{48\text{-}x}\cdot NO_3\cdot 12CH_3CN\cdot H_2O]^{3+}$ (x = 40, 41, … , 47, 48)			
**1**	$[Al_{24}(BA)_{12}(\mu_3\text{-}OH)_8(OH)_{48}\cdot NO_3\cdot 12CH_3CN\cdot H_2O]^{3+}$	1208.46	1208.50
**2**	$[Al_{24}(BA)_{12}(\mu_3\text{-}OH)_8(\mu_2\text{-}OH)_{47}(OEt)_1\cdot NO_3\cdot 12CH_3CN\cdot H_2O]^{3+}$	1217.80	1217.83
**3**	$[Al_{24}(BA)_{12}(\mu_3\text{-}OH)_8(\mu_2\text{-}OH)_{46}(OEt)_2\cdot NO_3\cdot 12CH_3CN\cdot H_2O]^{3+}$	1227.15	1227.17
**4**	$[Al_{24}(BA)_{12}(\mu_3\text{-}OH)_8(\mu_2\text{-}OH)_{45}(OEt)_3\cdot NO_3\cdot 12CH_3CN\cdot H_2O]^{3+}$	1236.49	1236.52
**5**	$[Al_{24}(BA)_{12}(\mu_3\text{-}OH)_8(\mu_2\text{-}OH)_{44}(OEt)_4\cdot NO_3\cdot 12CH_3CN\cdot H_2O]^{3+}$	1245.83	1245.86
**6**	$[Al_{24}(BA)_{12}(\mu_3\text{-}OH)_8(\mu_2\text{-}OH)_{43}(OEt)_5\cdot NO_3\cdot 12CH_3CN\cdot H_2O]^{3+}$	1255.18	1255.21
**7**	$[Al_{24}(BA)_{12}(\mu_3\text{-}OH)_8(\mu_2\text{-}OH)_{42}(OEt)_6\cdot NO_3\cdot 12CH_3CN\cdot H_2O]^{3+}$	1264.52	1264.55
**8**	$[Al_{24}(BA)_{12}(\mu_3\text{-}OH)_8(\mu_2\text{-}OH)_{41}(OEt)_7\cdot NO_3\cdot 12CH_3CN\cdot H_2O]^{3+}$	1273.87	1273.89
**9**	$[Al_{24}(BA)_{12}(\mu_3\text{-}OH)_8(\mu_2\text{-}OH)_{40}(OEt)_8\cdot NO_3\cdot 12CH_3CN\cdot H_2O]^{3+}$	1283.21	1283.25
Species: $[Al_{24}(BA)_{12}(\mu_3\text{-}OH)_8(\mu_2\text{-}OH)_x(OEt)_{48\text{-}x}\cdot NO_3\cdot 8CH_3CN\cdot 2H_2O]^{3+}$ (x = 36, 37, … , 43, 44)			
**1**	$[Al_{24}(BA)_{12}(\mu_3\text{-}OH)_8(\mu_2\text{-}OH)_{44}(OEt)_4\cdot NO_3\cdot 8CH_3CN\cdot 2H_2O]^{3+}$	1997.14	1197.16
**2**	$[Al_{24}(BA)_{12}(\mu_3\text{-}OH)_8(\mu_2\text{-}OH)_{43}(OEt)_5\cdot NO_3\cdot 8CH_3CN\cdot 2H_2O]^{3+}$	1206.48	1206.50
**3**	$[Al_{24}(BA)_{12}(\mu_3\text{-}OH)_8(\mu_2\text{-}OH)_{42}(OEt)_6\cdot NO_3\cdot 8CH_3CN\cdot 2H_2O]^{3+}$	1215.82	1215.85
**4**	$[Al_{24}(BA)_{12}(\mu_3\text{-}OH)_8(\mu_2\text{-}OH)_{41}(OEt)_7\cdot NO_3\cdot 8CH_3CN\cdot 2H_2O]^{3+}$	1225.17	1225.19
**5**	$[Al_{24}(BA)_{12}(\mu_3\text{-}OH)_8(\mu_2\text{-}OH)_{40}(OEt)_8\cdot NO_3\cdot 8CH_3CN\cdot 2H_2O]^{3+}$	1234.51	1234.53
**6**	$[Al_{24}(BA)_{12}(\mu_3\text{-}OH)_8(\mu_2\text{-}OH)_{39}(OEt)_9\cdot NO_3\cdot 8CH_3CN\cdot 2H_2O]^{3+}$	1243.85	1243.88
**7**	$[Al_{24}(BA)_{12}(\mu_3\text{-}OH)_8(\mu_2\text{-}OH)_{38}(OEt)_{10}\cdot NO_3\cdot 8CH_3CN\cdot 2H_2O]^{3+}$	1253.20	1253.22
**8**	$[Al_{24}(BA)_{12}(\mu_3\text{-}OH)_8(\mu_2\text{-}OH)_{37}(OEt)_{11}\cdot NO_3\cdot 8CH_3CN\cdot 2H_2O]^{3+}$	1262.54	1262.56
**9**	$[Al_{24}(BA)_{12}(\mu_3\text{-}OH)_8(\mu_2\text{-}OH)_{36}(OEt)_{12}\cdot NO_3\cdot 8CH_3CN\cdot 2H_2O]^{3+}$	1271.89	1271.85
Species: $[Al_{24}(BA)_{12}(\mu_3\text{-}OH)_8(\mu_2\text{-}OH)_x(OEt)_{48\text{-}x}\cdot NO_3\cdot 12CH_3CN\cdot 2H_2O]^{3+}$ (x = 36, 37)			
**1**	$[Al_{24}(BA)_{12}(\mu_3\text{-}OH)_8(\mu_2\text{-}OH)_{37}(OEt)_{11}\cdot NO_3\cdot 12CH_3CN\cdot 2H_2O]^{3+}$	1317.24	1317.22
**2**	$[Al_{24}(BA)_{12}(\mu_3\text{-}OH)_8(\mu_2\text{-}OH)_{36}(OEt)_{12}\cdot NO_3\cdot 12CH_3CN\cdot 2H_2O]^{3+}$	1326.59	1326.57
Species: $[Al_{24}(BA)_{12}(\mu_3\text{-}OH)_8(\mu_2\text{-}OH)_x(OEt)_{48\text{-}x}\cdot NO_3\cdot 11CH_3CN]^{3+}$ (x = 31, 32)			
**1**	$[Al_{24}(BA)_{12}(\mu_3\text{-}OH)_8(\mu_2\text{-}OH)_{32}(OEt)_{16}\cdot NO_3\cdot 11CH_3CN]^{3+}$	1338.28	1338.22
**2**	$[Al_{24}(BA)_{12}(\mu_3\text{-}OH)_8(\mu_2\text{-}OH)_{31}(OEt)_{17}\cdot NO_3\cdot 11CH_3CN]^{3+}$	1347.62	1347.59
Species: $[Al_{24}(BA)_{12}(\mu_3\text{-}OH)_8(\mu_2\text{-}OH)_x(OEt)_{48\text{-}x}\cdot NO_3\cdot 10CH_3CN]^{3+}$ (x = 33, 34, … , 37, 38)			
**1**	$[Al_{24}(BA)_{12}(\mu_3\text{-}OH)_8(\mu_2\text{-}OH)_{38}(OEt)_{10}\cdot NO_3\cdot 10CH_3CN]^{3+}$	1268.54	1268.51
**2**	$[Al_{24}(BA)_{12}(\mu_3\text{-}OH)_8(\mu_2\text{-}OH)_{37}(OEt)_{11}\cdot NO_3\cdot 10CH_3CN]^{3+}$	1277.89	1277.86
**3**	$[Al_{24}(BA)_{12}(\mu_3\text{-}OH)_8(\mu_2\text{-}OH)_{36}(OEt)_{12}\cdot NO_3\cdot 10CH_3CN]^{3+}$	1287.23	1287.20

**4**	$[Al_{24}(BA)_{12}(\mu_3\text{-}OH)_8(\mu_2\text{-}OH)_{35}(OEt)_{13}\cdot NO_3\cdot 10CH_3CN]^{3+}$	1296.57	1296.55
**5**	$[Al_{24}(BA)_{12}(\mu_3\text{-}OH)_8(\mu_2\text{-}OH)_{34}(OEt)_{14}\cdot NO_3\cdot 10CH_3CN]^{3+}$	1305.92	1305.89
**6**	$[Al_{24}(BA)_{12}(\mu_3\text{-}OH)_8(\mu_2\text{-}OH)_{33}(OEt)_{15}\cdot NO_3\cdot 10CH_3CN]^{3+}$	1315.26	1315.24
Species: $[Al_{24}(BA)_{12}(\mu_3\text{-}OH)_8(\mu_2\text{-}OH)_x(OEt)_{48\text{-}x}\cdot 2NO_3\cdot 10CH_3CN\cdot 4H_2O]^{2+}$ (x = 37, 38, … , 46, 47)			
**1**	$[Al_{24}(BA)_{12}(\mu_3\text{-}OH)_8(\mu_2\text{-}OH)_{47}(OEt)_1\cdot 2NO_3\cdot 10CH_3CN\cdot 4H_2O]^{2+}$	1843.69	1843.77
**2**	$[Al_{24}(BA)_{12}(\mu_3\text{-}OH)_8(\mu_2\text{-}OH)_{46}(OEt)_2\cdot 2NO_3\cdot 10CH_3CN\cdot 4H_2O]^{2+}$	1857.70	1857.74
**3**	$[Al_{24}(BA)_{12}(\mu_3\text{-}OH)_8(\mu_2\text{-}OH)_{45}(OEt)_3\cdot 2NO_3\cdot 10CH_3CN\cdot 4H_2O]^{2+}$	1871.72	1871.76
**4**	$[Al_{24}(BA)_{12}(\mu_3\text{-}OH)_8(\mu_2\text{-}OH)_{44}(OEt)_4\cdot 2NO_3\cdot 10CH_3CN\cdot 4H_2O]^{2+}$	1885.73	1885.78
**5**	$[Al_{24}(BA)_{12}(\mu_3\text{-}OH)_8(\mu_2\text{-}OH)_{43}(OEt)_5\cdot 2NO_3\cdot 10CH_3CN\cdot 4H_2O]^{2+}$	1899.75	1899.79
**6**	$[Al_{24}(BA)_{12}(\mu_3\text{-}OH)_8(\mu_2\text{-}OH)_{42}(OEt)_6\cdot 2NO_3\cdot 10CH_3CN\cdot 4H_2O]^{2+}$	1913.77	1913.81
**7**	$[Al_{24}(BA)_{12}(\mu_3\text{-}OH)_8(\mu_2\text{-}OH)_{41}(OEt)_7\cdot 2NO_3\cdot 10CH_3CN\cdot 4H_2O]^{2+}$	1927.78	1927.82
**8**	$[Al_{24}(BA)_{12}(\mu_3\text{-}OH)_8(\mu_2\text{-}OH)_{40}(OEt)_8\cdot 2NO_3\cdot 10CH_3CN\cdot 4H_2O]^{2+}$	1941.80	1941.84
**9**	$[Al_{24}(BA)_{12}(\mu_3\text{-}OH)_8(\mu_2\text{-}OH)_{39}(OEt)_9\cdot 2NO_3\cdot 10CH_3CN\cdot 4H_2O]^{2+}$	1955.81	1955.79
**10**	$[Al_{24}(BA)_{12}(\mu_3\text{-}OH)_8(\mu_2\text{-}OH)_{38}(OEt)_{10}\cdot 2NO_3\cdot 10CH_3CN\cdot 4H_2O]^{2+}$	1969.83	1969.79
**11**	$[Al_{24}(BA)_{12}(\mu_3\text{-}OH)_8(\mu_2\text{-}OH)_{37}(OEt)_{11}\cdot 2NO_3\cdot 10CH_3CN\cdot 4H_2O]^{2+}$	1983.84	1983.83
Species: $[Al_{24}(BA)_{12}(\mu_3\text{-}OH)_8(\mu_2\text{-}OH)_x(OEt)_{48\text{-}x}\cdot 2NO_3\cdot 9CH_3CN\cdot 2H_2O]^{2+}$ (x = 30, 31, … , 42, 43)			
**1**	$[Al_{24}(BA)_{12}(\mu_3\text{-}OH)_8(\mu_2\text{-}OH)_{43}(OEt)_5\cdot 2NO_3\cdot 9CH_3CN\cdot 2H_2O]^{2+}$	1861.23	1861.27
**2**	$[Al_{24}(BA)_{12}(\mu_3\text{-}OH)_8(\mu_2\text{-}OH)_{42}(OEt)_6\cdot 2NO_3\cdot 9CH_3CN\cdot 2H_2O]^{2+}$	1875.24	1875.29
**3**	$[Al_{24}(BA)_{12}(\mu_3\text{-}OH)_8(\mu_2\text{-}OH)_{41}(OEt)_7\cdot 2NO_3\cdot 9CH_3CN\cdot 2H_2O]^{2+}$	1889.26	1889.26
**4**	$[Al_{24}(BA)_{12}(\mu_3\text{-}OH)_8(\mu_2\text{-}OH)_{40}(OEt)_8\cdot 2NO_3\cdot 9CH_3CN\cdot 2H_2O]^{2+}$	1903.27	1903.28
**5**	$[Al_{24}(BA)_{12}(\mu_3\text{-}OH)_8(\mu_2\text{-}OH)_{39}(OEt)_9\cdot 2NO_3\cdot 9CH_3CN\cdot 2H_2O]^{2+}$	1917.29	1917.28
**6**	$[Al_{24}(BA)_{12}(\mu_3\text{-}OH)_8(\mu_2\text{-}OH)_{38}(OEt)_{10}\cdot 2NO_3\cdot 9CH_3CN\cdot 2H_2O]^{2+}$	1931.30	1931.30
**7**	$[Al_{24}(BA)_{12}(\mu_3\text{-}OH)_8(\mu_2\text{-}OH)_{37}(OEt)_{11}\cdot 2NO_3\cdot 9CH_3CN\cdot 2H_2O]^{2+}$	1945.32	1945.32
**8**	$[Al_{24}(BA)_{12}(\mu_3\text{-}OH)_8(\mu_2\text{-}OH)_{36}(OEt)_{12}\cdot 2NO_3\cdot 9CH_3CN\cdot 2H_2O]^{2+}$	1959.34	1959.34
**9**	$[Al_{24}(BA)_{12}(\mu_3\text{-}OH)_8(\mu_2\text{-}OH)_{35}(OEt)_{13}\cdot 2NO_3\cdot 9CH_3CN\cdot 2H_2O]^{2+}$	1973.35	1973.27
**10**	$[Al_{24}(BA)_{12}(\mu_3\text{-}OH)_8(\mu_2\text{-}OH)_{34}(OEt)_{14}\cdot 2NO_3\cdot 9CH_3CN\cdot 2H_2O]^{2+}$	1987.37	1987.30
**11**	$[Al_{24}(BA)_{12}(\mu_3\text{-}OH)_8(\mu_2\text{-}OH)_{33}(OEt)_{15}\cdot 2NO_3\cdot 9CH_3CN\cdot 2H_2O]^{2+}$	2001.38	2001.32
**12**	$[Al_{24}(BA)_{12}(\mu_3\text{-}OH)_8(\mu_2\text{-}OH)_{32}(OEt)_{16}\cdot 2NO_3\cdot 9CH_3CN\cdot 2H_2O]^{2+}$	2015.40	2015.33
**13**	$[Al_{24}(BA)_{12}(\mu_3\text{-}OH)_8(\mu_2\text{-}OH)_{31}(OEt)_{17}\cdot 2NO_3\cdot 9CH_3CN\cdot 2H_2O]^{2+}$	2029.41	2029.37
**14**	$[Al_{24}(BA)_{12}(\mu_3\text{-}OH)_8(\mu_2\text{-}OH)_{30}(OEt)_{18}\cdot 2NO_3\cdot 9CH_3CN\cdot 2H_2O]^{2+}$	2043.43	2043.38
Species: $[Al_{24}(BA)_{12}(\mu_3\text{-}OH)_8(\mu_2\text{-}OH)_x(OEt)_{48\text{-}x}\cdot NO_3\cdot OEt\cdot 5CH_3CN\cdot 2H_2O]^{2+}$ (x = 27, 28, … , 39, 40)			

**1**	$[Al_{24}(BA)_{12}(\mu_3\text{-}OH)_8(\mu_2\text{-}OH)_{40}(OEt)_8{\cdot}NO_3{\cdot}OEt{\cdot}5CH_3CN{\cdot}2H_2O]^{2+}$	1812.74	1812.73
**2**	$[Al_{24}(BA)_{12}(\mu_3\text{-}OH)_8(\mu_2\text{-}OH)_{39}(OEt)_9{\cdot}NO_3{\cdot}OEt{\cdot}5CH_3CN{\cdot}2H_2O]^{2+}$	1826.76	1826.74
**3**	$[Al_{24}(BA)_{12}(\mu_3\text{-}OH)_8(\mu_2\text{-}OH)_{38}(OEt)_{10}{\cdot}NO_3{\cdot}OEt{\cdot}5CH_3CN{\cdot}2H_2O]^{2+}$	1840.77	1840.76
**4**	$[Al_{24}(BA)_{12}(\mu_3\text{-}OH)_8(\mu_2\text{-}OH)_{37}(OEt)_{11}{\cdot}NO_3{\cdot}OEt{\cdot}5CH_3CN{\cdot}2H_2O]^{2+}$	1854.79	1854.76
**5**	$[Al_{24}(BA)_{12}(\mu_3\text{-}OH)_8(\mu_2\text{-}OH)_{36}(OEt)_{12}{\cdot}NO_3{\cdot}OEt{\cdot}5CH_3CN{\cdot}2H_2O]^{2+}$	1868.81	1868.79
**6**	$[Al_{24}(BA)_{12}(\mu_3\text{-}OH)_8(\mu_2\text{-}OH)_{35}(OEt)_{13}{\cdot}NO_3{\cdot}OEt{\cdot}5CH_3CN{\cdot}2H_2O]^{2+}$	1882.82	1882.79
**7**	$[Al_{24}(BA)_{12}(\mu_3\text{-}OH)_8(\mu_2\text{-}OH)_{34}(OEt)_{14}{\cdot}NO_3{\cdot}OEt{\cdot}5CH_3CN{\cdot}2H_2O]^{2+}$	1896.94	1896.82
**8**	$[Al_{24}(BA)_{12}(\mu_3\text{-}OH)_8(\mu_2\text{-}OH)_{33}(OEt)_{15}{\cdot}NO_3{\cdot}OEt{\cdot}5CH_3CN{\cdot}2H_2O]^{2+}$	1910.85	1910.83
**9**	$[Al_{24}(BA)_{12}(\mu_3\text{-}OH)_8(\mu_2\text{-}OH)_{32}(OEt)_{16}{\cdot}NO_3{\cdot}OEt{\cdot}5CH_3CN{\cdot}2H_2O]^{2+}$	1924.87	1924.84
**10**	$[Al_{24}(BA)_{12}(\mu_3\text{-}OH)_8(\mu_2\text{-}OH)_{31}(OEt)_{17}{\cdot}NO_3{\cdot}OEt{\cdot}5CH_3CN{\cdot}2H_2O]^{2+}$	1938.88	1938.82
**11**	$[Al_{24}(BA)_{12}(\mu_3\text{-}OH)_8(\mu_2\text{-}OH)_{30}(OEt)_{18}{\cdot}NO_3{\cdot}OEt{\cdot}5CH_3CN{\cdot}2H_2O]^{2+}$	1952.90	1952.83
**12**	$[Al_{24}(BA)_{12}(\mu_3\text{-}OH)_8(\mu_2\text{-}OH)_{29}(OEt)_{19}{\cdot}NO_3{\cdot}OEt{\cdot}5CH_3CN{\cdot}2H_2O]^{2+}$	1966.92	1966.88
**13**	$[Al_{24}(BA)_{12}(\mu_3\text{-}OH)_8(\mu_2\text{-}OH)_{28}(OEt)_{20}{\cdot}NO_3{\cdot}OEt{\cdot}5CH_3CN{\cdot}2H_2O]^{2+}$	1980.93	1980.84
**14**	$[Al_{24}(BA)_{12}(\mu_3\text{-}OH)_8(\mu_2\text{-}OH)_{27}(OEt)_{21}{\cdot}NO_3{\cdot}OEt{\cdot}5CH_3CN{\cdot}2H_2O]^{2+}$	1994.95	1994.85
Species: $[Al_{24}(BA)_{12}(\mu_3\text{-}OH)_8(\mu_2\text{-}OH)_x(OEt)_{48\text{-}x}{\cdot}2NO_3{\cdot}13CH_3CN{\cdot}H_2O]^{2+}$ (x = 35, 36, … , 46, 47)			
**1**	$[Al_{24}(BA)_{12}(\mu_3\text{-}OH)_8(\mu_2\text{-}OH)_{47}(OEt)_1{\cdot}2NO_3{\cdot}13CH_3CN{\cdot}H_2O]^{2+}$	1878.21	1878.22
**2**	$[Al_{24}(BA)_{12}(\mu_3\text{-}OH)_8(\mu_2\text{-}OH)_{46}(OEt)_2{\cdot}2NO_3{\cdot}13CH_3CN{\cdot}H_2O]^{2+}$	1892.23	1892.29
**3**	$[Al_{24}(BA)_{12}(\mu_3\text{-}OH)_8(\mu_2\text{-}OH)_{45}(OEt)_3{\cdot}2NO_3{\cdot}13CH_3CN{\cdot}H_2O]^{2+}$	1906.24	1906.27
**4**	$[Al_{24}(BA)_{12}(\mu_3\text{-}OH)_8(\mu_2\text{-}OH)_{44}(OEt)_4{\cdot}2NO_3{\cdot}13CH_3CN{\cdot}H_2O]^{2+}$	1920.26	1920.25
**5**	$[Al_{24}(BA)_{12}(\mu_3\text{-}OH)_8(\mu_2\text{-}OH)_{43}(OEt)_5{\cdot}2NO_3{\cdot}13CH_3CN{\cdot}H_2O]^{2+}$	1934.27	1934.27
**6**	$[Al_{24}(BA)_{12}(\mu_3\text{-}OH)_8(\mu_2\text{-}OH)_{42}(OEt)_6{\cdot}2NO_3{\cdot}13CH_3CN{\cdot}H_2O]^{2+}$	1948.29	1948.30
**7**	$[Al_{24}(BA)_{12}(\mu_3\text{-}OH)_8(\mu_2\text{-}OH)_{41}(OEt)_7{\cdot}2NO_3{\cdot}13CH_3CN{\cdot}H_2O]^{2+}$	1962.31	1962.31
**8**	$[Al_{24}(BA)_{12}(\mu_3\text{-}OH)_8(\mu_2\text{-}OH)_{40}(OEt)_8{\cdot}2NO_3{\cdot}13CH_3CN{\cdot}H_2O]^{2+}$	1976.32	1976.32
**9**	$[Al_{24}(BA)_{12}(\mu_3\text{-}OH)_8(\mu_2\text{-}OH)_{39}(OEt)_9{\cdot}2NO_3{\cdot}13CH_3CN{\cdot}H_2O]^{2+}$	1990.34	1990.34
**10**	$[Al_{24}(BA)_{12}(\mu_3\text{-}OH)_8(\mu_2\text{-}OH)_{38}(OEt)_{10}{\cdot}2NO_3{\cdot}13CH_3CN{\cdot}H_2O]^{2+}$	2004.35	2004.29
**11**	$[Al_{24}(BA)_{12}(\mu_3\text{-}OH)_8(\mu_2\text{-}OH)_{37}(OEt)_{11}{\cdot}2NO_3{\cdot}13CH_3CN{\cdot}H_2O]^{2+}$	2018.37	2018.31
**12**	$[Al_{24}(BA)_{12}(\mu_3\text{-}OH)_8(\mu_2\text{-}OH)_{36}(OEt)_{12}{\cdot}2NO_3{\cdot}13CH_3CN{\cdot}H_2O]^{2+}$	2032.38	2032.33
**13**	$[Al_{24}(BA)_{12}(\mu_3\text{-}OH)_8(\mu_2\text{-}OH)_{35}(OEt)_{13}{\cdot}2NO_3{\cdot}13CH_3CN{\cdot}H_2O]^{2+}$	2046.38	2046.40

Supplementary Table 7. The assignment of the key species for **AlMC-5** dissolved in MeCN under the positive mode of ESI-MS tests. (Both calculated and experimental m/z values refer to the most intense peak in the isotopic envelope).

No.	Chemistry Formula	Cal. m/z	Exp. m/z

Species: $[Al_{24}(BA)_{12}(\mu_3\text{-}OH)_8(\mu_2\text{-}OH)_x(OEt)_{48\text{-}x}\cdot NO_3]^{3+}$ (x = 25, 26, 27, ·…, 35, 36)			
**1**	$[Al_{24}(BA)_{12}(\mu_3\text{-}OH)_8(\mu_2\text{-}OH)_{36}(OEt)_{12}\cdot NO_3]^{3+}$	1150.47	1150.45
**2**	$[Al_{24}(BA)_{12}(\mu_3\text{-}OH)_8(\mu_2\text{-}OH)_{35}(OEt)_{13}\cdot NO_3]^{3+}$	1159.82	1159.79
**3**	$[Al_{24}(BA)_{12}(\mu_3\text{-}OH)_8(\mu_2\text{-}OH)_{34}(OEt)_{14}\cdot NO_3]^{3+}$	1169.16	1169.14
**4**	$[Al_{24}(BA)_{12}(\mu_3\text{-}OH)_8(\mu_2\text{-}OH)_{33}(OEt)_{15}\cdot NO_3]^{3+}$	1178.51	1178.48
**5**	$[Al_{24}(BA)_{12}(\mu_3\text{-}OH)_8(\mu_2\text{-}OH)_{32}(OEt)_{16}\cdot NO_3]^{3+}$	1187.85	1187.82
**6**	$[Al_{24}(BA)_{12}(\mu_3\text{-}OH)_8(\mu_2\text{-}OH)_{31}(OEt)_{17}\cdot NO_3]^{3+}$	1197.19	1197.17
**7**	$[Al_{24}(BA)_{12}(\mu_3\text{-}OH)_8(\mu_2\text{-}OH)_{30}(OEt)_{18}\cdot NO_3]^{3+}$	1206.54	1206.51
**8**	$[Al_{24}(BA)_{12}(\mu_3\text{-}OH)_8(\mu_2\text{-}OH)_{29}(OEt)_{19}\cdot NO_3]^{3+}$	1215.88	1215.86
**9**	$[Al_{24}(BA)_{12}(\mu_3\text{-}OH)_8(\mu_2\text{-}OH)_{28}(OEt)_{20}\cdot NO_3]^{3+}$	1225.22	1225.20
**10**	$[Al_{24}(BA)_{12}(\mu_3\text{-}OH)_8(\mu_2\text{-}OH)_{27}(OEt)_{21}\cdot NO_3]^{3+}$	1234.57	1234.54
**11**	$[Al_{24}(BA)_{12}(\mu_3\text{-}OH)_8(\mu_2\text{-}OH)_{26}(OEt)_{22}\cdot NO_3]^{3+}$	1243.91	1243.89
**12**	$[Al_{24}(BA)_{12}(\mu_3\text{-}OH)_8(\mu_2\text{-}OH)_{25}(OEt)_{23}\cdot NO_3]^{3+}$	1253.26	1253.23
Species: $[Al_{24}(BA)_{12}(\mu_3\text{-}OH)_8(\mu_2\text{-}OH)_x(OEt)_{48\text{-}x}\cdot NO_3\cdot Br]^{2+}$ (x = 27, 28, … , 32, 33)			
**1**	$[Al_{24}(BA)_{12}(\mu_3\text{-}OH)_8(\mu_2\text{-}OH)_{33}(OEt)_{15}\cdot NO_3\cdot Br]^{2+}$	1807.72	1807.68
**2**	$[Al_{24}(BA)_{12}(\mu_3\text{-}OH)_8(\mu_2\text{-}OH)_{32}(OEt)_{16}\cdot NO_3\cdot Br]^{2+}$	1821.73	1821.70
**3**	$[Al_{24}(BA)_{12}(\mu_3\text{-}OH)_8(\mu_2\text{-}OH)_{31}(OEt)_{17}\cdot NO_3\cdot Br]^{2+}$	1835.75	1835.71
**4**	$[Al_{24}(BA)_{12}(\mu_3\text{-}OH)_8(\mu_2\text{-}OH)_{30}(OEt)_{18}\cdot NO_3\cdot Br]^{2+}$	1849.76	1849.73
**5**	$[Al_{24}(BA)_{12}(\mu_3\text{-}OH)_8(\mu_2\text{-}OH)_{29}(OEt)_{19}\cdot NO_3\cdot Br]^{2+}$	1863.78	1863.75
**6**	$[Al_{24}(BA)_{12}(\mu_3\text{-}OH)_8(\mu_2\text{-}OH)_{28}(OEt)_{20}\cdot NO_3\cdot Br]^{2+}$	1877.80	1877.76
**7**	$[Al_{24}(BA)_{12}(\mu_3\text{-}OH)_8(\mu_2\text{-}OH)_{27}(OEt)_{21}\cdot NO_3\cdot Br]^{2+}$	1891.81	1891.77
Species: $[Al_{24}(BA)_{12}(\mu_3\text{-}OH)_8(\mu_2\text{-}OH)_x(OEt)_{48\text{-}x}\cdot 9CH_3CN\cdot NO_3\cdot Br]^{2+}$ (x = 36, 37, … , 47, 48)			
**1**	$[Al_{24}(BA)_{12}(\mu_3\text{-}OH)_8(\mu_2\text{-}OH)_{48}\cdot 9CH_3CN\cdot NO_3\cdot Br]^{2+}$	1782.11	1782.17
**2**	$[Al_{24}(BA)_{12}(\mu_3\text{-}OH)_8(\mu_2\text{-}OH)_{47}(OEt)_1\cdot 9CH_3CN\cdot NO_3\cdot Br]^{2+}$	1796.12	1796.18
**3**	$[Al_{24}(BA)_{12}(\mu_3\text{-}OH)_8(\mu_2\text{-}OH)_{46}(OEt)_2\cdot 9CH_3CN\cdot NO_3\cdot Br]^{2+}$	1810.13	1810.20
**4**	$[Al_{24}(BA)_{12}(\mu_3\text{-}OH)_8(\mu_2\text{-}OH)_{45}(OEt)_3\cdot 9CH_3CN\cdot NO_3\cdot Br]^{2+}$	1824.15	1824.22
**5**	$[Al_{24}(BA)_{12}(\mu_3\text{-}OH)_8(\mu_2\text{-}OH)_{44}(OEt)_4\cdot 9CH_3CN\cdot NO_3\cdot Br]^{2+}$	1838.16	1838.23
**6**	$[Al_{24}(BA)_{12}(\mu_3\text{-}OH)_8(\mu_2\text{-}OH)_{43}(OEt)_5\cdot 9CH3CN\cdot NO_3\cdot Br]^{2+}$	1852.18	1852.24
**7**	$[Al_{24}(BA)_{12}(\mu_3\text{-}OH)_8(\mu_2\text{-}OH)_{42}(OEt)_6\cdot 9CH_3CN\cdot NO_3\cdot Br]^{2+}$	1866.20	1866.26
**8**	$[Al_{24}(BA)_{12}(\mu_3\text{-}OH)_8(\mu_2\text{-}OH)_{41}(OEt)_7\cdot 9CH_3CN\cdot NO_3\cdot Br]^{2+}$	1880.21	1880.27
**9**	$[Al_{24}(BA)_{12}(\mu_3\text{-}OH)_8(\mu_2\text{-}OH)_{40}(OEt)_8\cdot 9CH_3CN\cdot NO_3\cdot Br]^{2+}$	1894.23	1894.29
**10**	$[Al_{24}(BA)_{12}(\mu_3\text{-}OH)_8(\mu_2\text{-}OH)_{39}(OEt)_9\cdot 9CH_3CN\cdot NO_3\cdot Br]^{2+}$	1908.24	1908.31

**11**	$[Al_{24}(BA)_{12}(\mu_3\text{-}OH)_8(\mu_2\text{-}OH)_{38}(OEt)_{10}\cdot 9CH_3CN\cdot NO_3\cdot Br]^{2+}$	1922.26	1922.32
**12**	$[Al_{24}(BA)_{12}(\mu_3\text{-}OH)_8(\mu_2\text{-}OH)_{37}(OEt)_{11}\cdot 9CH_3CN\cdot NO_3\cdot Br]^{2+}$	1936.27	1936.33
**13**	$[Al_{24}(BA)_{12}(\mu_3\text{-}OH)_8(\mu_2\text{-}OH)_{36}(OEt)_{12}\cdot 9CH_3CN\cdot NO3\cdot Br]^{2+}$	1950.29	1950.31
Species: $[Al_{24}(BA)_{12}(\mu_3\text{-}OH)_8(\mu_2\text{-}OH)_x(OEt)_{48\text{-}x}\cdot 3CH_3CN\cdot NO_3\cdot Br]^{2+}$ (x = 32, 33, … , 38, 39)			
**1**	$[Al_{24}(BA)_{12}(\mu_3\text{-}OH)_8(\mu_2\text{-}OH)_{39}(OEt)_9\cdot 3CH_3CN\cdot NO_3\cdot Br]^{2+}$	1785.16	1785.20
**2**	$[Al_{24}(BA)_{12}(\mu_3\text{-}OH)_8(\mu_2\text{-}OH)_{38}(OEt)_{10}\cdot 3CH_3CN\cdot NO_3\cdot Br]^{2+}$	1799.18	1799.22
**3**	$[Al_{24}(BA)_{12}(\mu_3\text{-}OH)_8(\mu_2\text{-}OH)_{37}(OEt)_{11}\cdot 3CH_3CN\cdot NO_3\cdot Br]^{2+}$	1813.19	1813.23
**4**	$[Al_{24}(BA)_{12}(\mu_3\text{-}OH)_8(\mu_2\text{-}OH)_{36}(OEt)_{12}\cdot 3CH_3CN\cdot NO_3\cdot Br]^{2+}$	1827.21	1827.24
**5**	$[Al_{24}(BA)_{12}(\mu_3\text{-}OH)_8(\mu_2\text{-}OH)_{35}(OEt)_{13}\cdot 3CH_3CN\cdot NO_3\cdot Br]^{2+}$	1841.23	1841.26
**6**	$[Al_{24}(BA)_{12}(\mu_3\text{-}OH)_8(\mu_2\text{-}OH)_{34}(OEt)_{14}\cdot 3CH_3CN\cdot NO_3\cdot Br]^{2+}$	1855.24	1855.28
**7**	$[Al_{24}(BA)_{12}(\mu_3\text{-}OH)_8(\mu_2\text{-}OH)_{33}(OEt)_{15}\cdot 3CH_3CN\cdot NO_3\cdot Br]^{2+}$	1869.26	1869.29
**8**	$[Al_{24}(BA)_{12}(\mu_3\text{-}OH)_8(\mu_2\text{-}OH)_{32}(OEt)_{16}\cdot 3CH_3CN\cdot NO_3\cdot Br]^{2+}$	1883.27	1883.31
Species: $[Al_{24}(BA)_{12}(\mu_3\text{-}OH)_8(\mu_2\text{-}OH)_x(OEt)_{48\text{-}x}\cdot 13CH_3CN\cdot NO_3\cdot Br]^{2+}$ (x = 39, 40, … , 44, 45)			
**1**	$[Al_{24}(BA)_{12}(\mu_3\text{-}OH)_8(\mu_2\text{-}OH)_{45}(OEt)_3\cdot 13CH_3CN\cdot NO_3\cdot Br]^{2+}$	1906.20	1906.28
**2**	$[Al_{24}(BA)_{12}(\mu_3\text{-}OH)_8(\mu_2\text{-}OH)_{44}(OEt)_4\cdot 13CH_3CN\cdot NO_3\cdot Br]^{2+}$	1920.22	1920.23
**3**	$[Al_{24}(BA)_{12}(\mu_3\text{-}OH)_8(\mu_2\text{-}OH)_{43}(OEt)_5\cdot 13CH_3CN\cdot NO_3\cdot Br]^{2+}$	1934.23	1934.25
**4**	$[Al_{24}(BA)_{12}(\mu_3\text{-}OH)_8(\mu_2\text{-}OH)_{42}(OEt)_6\cdot 13CH_3CN\cdot NO_3\cdot Br]^{2+}$	1948.25	1948.27
**5**	$[Al_{24}(BA)_{12}(\mu_3\text{-}OH)_8(\mu_2\text{-}OH)_{41}(OEt)_7\cdot 13CH_3CN\cdot NO_3\cdot Br]^{2+}$	1962.26	1962.27
**6**	$[Al_{24}(BA)_{12}(\mu_3\text{-}OH)_8(\mu_2\text{-}OH)_{40}(OEt)_8\cdot 13CH_3CN\cdot NO_3\cdot Br]^{2+}$	1976.28	1976.29
**7**	$[Al_{24}(BA)_{12}(\mu_3\text{-}OH)_8(\mu_2\text{-}OH)_{39}(OEt)_9\cdot 13CH_3CN\cdot NO_3\cdot Br]^{2+}$	1990.30	1990.31

Supplementary Table 8. The assignment of the key species for **AlMC-7** dissolved in the mixture solution of MeCN and DMF under the negative mode of ESI-MS tests. (Both calculated and experimental m/z values refer to the most intense peak in the isotopic envelope).

No.	Chemistry Formula	Cal. m/z	Exp. m/z
**1**	$[Al_6(BA)_6(NO_3)_2(OH)_{11}\cdot(CH_3CN)_4]^-$	1363.17	1363.10
Species: $[Al_{24}(BA)_{12}(\mu_3\text{-}OH)_8(\mu_2\text{-}OH)_x(OEt)_{48\text{-}x}\cdot(5\text{-}y)OEt\cdot yOH\cdot 8CH_3CN\cdot DMF\cdot NO_3]^{2-}$ (x = 48, y = 0, 1, 2, 3; x = 39, 40, … , 46, 47, y = 0)			
**2**	$[Al_{24}(BA)_{12}(\mu_3\text{-}OH)_8(OH)_{48}\cdot 2OEt\cdot 3OH\cdot 8CH_3CN\cdot DMF\cdot NO_3]^{2-}$	1828.70	1828.63
**3**	$[Al_{24}(BA)_{12}(\mu_3\text{-}OH)_8(\mu_2\text{-}OH)_{48}\cdot 3OEt\cdot 2OH\cdot 8CH_3CN\cdot DMF\cdot NO_3]^{2-}$	1842.71	1842.66
**4**	$[Al_{24}(BA)_{12}(\mu_3\text{-}OH)_8(\mu_2\text{-}OH)_{48}\cdot 4OEt\cdot OH\cdot 8CH_3CN\cdot DMF\cdot NO_3]^{2-}$	1856.73	1856.66
**5**	$[Al_{24}(BA)_{12}(\mu_3\text{-}OH)_8(\mu_2\text{-}OH)_{48}\cdot 5OEt\cdot 8CH_3CN\cdot DMF\cdot NO_3]^{2-}$	1870.74	1870.68
**6**	$[Al_{24}(BA)_{12}(\mu_3\text{-}OH)_8(\mu_2\text{-}OH)_{47}(OEt)\cdot 5OEt\cdot 8CH_3CN\cdot DMF\cdot NO_3]^{2-}$	1884.76	1884.69
**7**	$[Al_{24}(BA)_{12}(\mu_3\text{-}OH)_8(\mu_2\text{-}OH)_{46}(OEt)_2\cdot 5OEt\cdot 8CH_3CN\cdot DMF\cdot NO_3]^{2-}$	1898.77	1898.71

**8**	$[Al_{24}(BA)_{12}(\mu_3\text{-}OH)_8(\mu_2\text{-}OH)_{45}(OEt)_3\cdot 5OEt\cdot 8CH_3CN\cdot DMF\cdot NO_3]^{2-}$	1912.79	1912.72
**9**	$[Al_{24}(BA)_{12}(\mu_3\text{-}OH)_8(\mu_2\text{-}OH)_{44}(OEt)_4\cdot 5OEt\cdot 8CH_3CN\cdot DMF\cdot NO_3]^{2-}$	1926.81	1926.74
**10**	$[Al_{24}(BA)_{12}(\mu_3\text{-}OH)_8(\mu_2\text{-}OH)_{43}(OEt)_5\cdot 5OEt\cdot 8CH_3CN\cdot DMF\cdot NO_3]^{2-}$	1940.82	1940.76
**11**	$[Al_{24}(BA)_{12}(\mu_3\text{-}OH)_8(\mu_2\text{-}OH)_{42}(OEt)_6\cdot 5OEt\cdot 8CH_3CN\cdot DMF\cdot NO_3]^{2-}$	1954.84	1954.76
**12**	$[Al_{24}(BA)_{12}(\mu_3\text{-}OH)_8(\mu_2\text{-}OH)_{41}(OEt)_7\cdot 5OEt\cdot 8CH_3CN\cdot DMF\cdot NO_3]^{2-}$	1968.85	1968.73
**13**	$[Al_{24}(BA)_{12}(\mu_3\text{-}OH)_8(\mu_2\text{-}OH)_{40}(OEt)_8\cdot 5OEt\cdot 8CH_3CN\cdot DMF\cdot NO_3]^{2-}$	1982.87	1982.74
**14**	$[Al_{24}(BA)_{12}(\mu_3\text{-}OH)_8(\mu_2\text{-}OH)_{39}(OEt)_9\cdot 5OEt\cdot 8CH_3CN\cdot DMF\cdot NO_3]^{2-}$	1996.88	1996.79
Species: $[Al_{24}(BA)_{12}(\mu_3\text{-}OH)_8(\mu_2\text{-}OH)_x(OEt)_{48\text{-}x}\cdot(5\text{-}y)OEt\cdot yOH\cdot 3CH_3CN\cdot 6DMF\cdot NO_3]^{2-}$ (x = 48, y = 0, 1, 2; x = 46, 47, y = 0)			
**1**	$[Al_{24}(BA)_{12}(\mu_3\text{-}OH)_8(\mu_2\text{-}OH)_{48}\cdot 3(OEt)\cdot 2(OH)\cdot 3CH_3CN\cdot 6DMF\cdot NO_3]^{2-}$	1922.78	1922.71
**2**	$[Al_{24}(BA)_{12}(\mu_3\text{-}OH)_8(\mu_2\text{-}OH)_{48}\cdot 4(OEt)\cdot OH\cdot 3CH_3CN\cdot 6DMF\cdot NO3]^{2-}$	1936.79	1936.71
**3**	$[Al_{24}(BA)_{12}(\mu_3\text{-}OH)8(\mu_2\text{-}OH)_{48}\cdot 5(OEt)\cdot 3CH_3CN\cdot 6DMF\cdot NO_3]^{2-}$	1950.81	1950.73
**4**	$[Al_{24}(BA)_{12}(\mu_3\text{-}OH)_8(\mu_2\text{-}OH)_{47}(OEt)\cdot 5(OEt)\cdot 3CH_3CN\cdot 6DMF\cdot NO_3]^{2-}$	1964.82	1964.75
**5**	$[Al_{24}(BA)_{12}(\mu_3\text{-}OH)_8(\mu_2\text{-}OH)_{46}(OEt)_2\cdot 5(OEt)\cdot 3CH_3CN\cdot 6DMF\cdot NO_3]^{2-}$	1978.84	1978.76
Species: $[Al_{24}(BA)_{12}(\mu_3\text{-}OH)_8(\mu_2\text{-}OH)_x(OEt)_{48\text{-}x}\cdot(4\text{-}y)OEt\cdot yOH\cdot 10CH_3CN\cdot NO_3]^-$ (x = 48, y = 0, 1, 2; x =39, 40, … 46, 47, y = 0			
**1**	$[Al_{24}(BA)_{12}(\mu_3\text{-}OH)_8(\mu_2\text{-}OH)_{48}\cdot 2OEt\cdot 2OH\cdot 10CH_3CN\cdot NO_3]^-$	3649.39	3649.33
**2**	$[Al_{24}(BA)_{12}(\mu_3\text{-}OH)_8(\mu_2\text{-}OH)_{48}\cdot 3OEt\cdot OH\cdot 10CH_3CN\cdot NO_3]^-$	3677.42	3677.37
**3**	$[Al_{24}(BA)_{12}(\mu_3\text{-}OH)_8(\mu_2\text{-}OH)_{48}\cdot 4OEt\cdot 10CH_3CN\cdot NO_3]^-$	3705.45	3705.39
**4**	$[Al_{24}(BA)_{12}(\mu_3\text{-}OH)_8(\mu_2\text{-}OH)_{47}(OEt)_1\cdot 4OEt\cdot 10CH_3CN\cdot NO_3]^-$	3733.48	3733.43
**5**	$[Al_{24}(BA)_{12}(\mu_3\text{-}OH)_8(\mu_2\text{-}OH)_{46}(OEt)_2\cdot 4OEt\cdot 10CH_3CN\cdot NO_3]^-$	3761.51	3761.45
**6**	$[Al_{24}(BA)_{12}(\mu_3\text{-}OH)_8(\mu_2\text{-}OH)_{45}(OEt)_3\cdot 4OEt\cdot 10CH_3CN\cdot NO_3]^-$	3789.54	3789.48
**7**	$[Al_{24}(BA)_{12}(\mu_3\text{-}OH)_8(\mu_2\text{-}OH)_{44}(OEt)_4\cdot 4OEt\cdot 10CH_3CN\cdot NO_3]^-$	3817.58	3817.52
**8**	$[Al_{24}(BA)_{12}(\mu_3\text{-}OH)_8(\mu_2\text{-}OH)_{43}(OEt)_5\cdot 4OEt\cdot 10CH_3CN\cdot NO_3]^-$	3845.61	3845.55
**9**	$[Al_{24}(BA)_{12}(\mu_3\text{-}OH)_8(\mu_2\text{-}OH)_{42}(OEt)_6\cdot 4OEt\cdot 10CH_3CN\cdot NO_3]^-$	3873.64	3873.57
**10**	$[Al_{24}(BA)_{12}(\mu_3\text{-}OH)_8(\mu_2\text{-}OH)_{41}(OEt)_7\cdot 4OEt\cdot 10CH_3CN\cdot NO_3]^-$	3901.67	3901.59
**11**	$[Al_{24}(BA)_{12}(\mu_3\text{-}OH)_8(\mu_2\text{-}OH)_{40}(OEt)_8\cdot 4OEt\cdot 10CH_3CN\cdot NO_3]^-$	3929.70	3929.62
**12**	$[Al_{24}(BA)_{12}(\mu_3\text{-}OH)_8(\mu_2\text{-}OH)_{39}(OEt)_9\cdot 4OEt\cdot 10CH_3CN\cdot NO_3]^-$	3957.73	3957.67
Species: $[Al_{24}(BA)_{12}(\mu_3\text{-}OH)_8(\mu_2\text{-}OH)_x(OEt)_{48\text{-}x}\cdot 2OEt\cdot 6CH_3CN\cdot 4DMF\cdot 3NO_3]^-$ (x = 48, 47, 46)			
**1**	$[Al_{24}(BA)_{12}(\mu_3\text{-}OH)_8(\mu_2\text{-}OH)_{48}\cdot 2OEt\cdot 6CH_3CN\cdot 4DMF\cdot 3NO_3]^-$	3867.46	3867.50
**2**	$[Al_{24}(BA)_{12}(\mu_3\text{-}OH)_8(\mu_2\text{-}OH)_{47}(OEt)\cdot 2OEt\cdot 6CH_3CN\cdot 4DMF\cdot 3NO_3]^-$	3895.49	3895.52
**3**	$[Al_{24}(BA)_{12}(\mu_3\text{-}OH)_8(\mu_2\text{-}OH)_{46}(OEt)_2\cdot 2OEt\cdot 6CH_3CN\cdot 4DMF\cdot 3NO_3]^-$	3923.52	3923.56
Species: $[Al_{24}(BA)_{12}(\mu_3\text{-}OH)_8(\mu_2\text{-}OH)_x(OEt)_{48\text{-}x}\cdot 2OEt\cdot 8CH_3CN\cdot 3DMF\cdot 3NO_3]^-$ (x = 48, 47, 46)			

**1**	$[Al_{24}(BA)_{12}(\mu_3\text{-}OH)_8(\mu_2\text{-}OH)_{48}\cdot 2OEt\cdot 8CH_3CN\cdot 3DMF\cdot 3NO_3]^-$	3876.46	3876.46
**2**	$[Al_{24}(BA)_{12}(\mu_3\text{-}OH)_8(\mu_2\text{-}OH)_{47}(OEt)\cdot 2OEt\cdot 8CH_3CN\cdot 3DMF\cdot 3NO_3]^-$	3904.49	3904.50
**3**	$[Al_{24}(BA)_{12}(\mu_3\text{-}OH)_8(\mu_2\text{-}OH)_{46}(OEt)_2\cdot 2OEt\cdot 8CH_3CN\cdot 3DMF\cdot 3NO_3]^-$	3932.53	3932.54

4.4 Summary of sorbents for iodine ion capture in water

Supplementary Table 9. Summary of sorbents for iodine ion capture in water.

**Materials**	**BET suface area (m^2/g)**	**Iodine uptake (wt%)**	**Conditions**	**Mechanism**	**Release solvent / Recyclability**	**Ref.**
C[4]P-BTP	20.5 m^2/g	3.24 g/g	KI/I_2 aqueous solution	chemisorption	Heat release / Recyclability	37
C[4]P-BT	28.3 m^2/g	2.32 g/g				
C[4]P-TTP	19.0 m^2/g	2.51 g/g				
C[4]P-BP	41.9 m^2/g	2.37 g/g				
C[4]P-TPE	77.7 m^2/g	2.99 g/g				
C[4]P-DPP	110.0 m^2/g	1.58 g/g				
HPOC-101	373 m^2/g	1.38 g/g	KI/I_2 aqueous solution		In MeOH	38
CaCOP1	10.86 m^2/g	240 wt%	high-concentration I_2/KI aqueous solution	physical and chemical adsorption	In ethanol / 100% reversible sorption	39
CaCOP2	20.16 m^2/g	281 wt%				
CaCOP3	81.09 m^2/g	310 wt%				
H_COF-1	—	2.1 ± 0.1 g/g	high-concentration I_2/KI aqueous solution		In DMSO; >93% releasing efficiency	40
$\{[Mn_2(oxdz)_2(tpbn)(H_2O)_2]\cdot 2C_2H_5OH\}_n$	—	1.1 ± 0.05 g/g	I_2/KI aqueous solution	physisorption process	In ethanol / 100% reversible sorption	41
**AlMC-1**	233 m^2/g	1.03 g/g	high-concentration I_2/KI aqueous solution	ion-exchange	In ethanol / Recyclability	This work
$[Cd(L)_2(ClO_4)_2]\cdot H_2O$, L = 4-amino-3,5-bis (4-pyridyl-3phenyl)-1,2,4-triazole	—	0.18 g I_3^-/g	400 ppm I_2/KI aqueous solution	ion-exchange	Not mention	42
		0.10 g I^-/g	40 ppm $NaIO_3$ aqueous solution		In 40 ppm $NaClO_4/Cd(ClO_4)_2$ aqueous solution / Recyclability	
$[Zn_4(TPBA)_4(ClO_4)_8]\cdot$solvents (MOC-19)	—	144.9 $mg\cdot g^{-1}\cdot h^{-1}$	180 ppm KI/I_2 aqueous solution	ion-exchange	Not mention	43
		—	50 ppm KIO_3			
PVDF/ZIF-8 nanocomposite membrane	—	73.33 mg/g	iodine aqueous solution	membrane filtration: electrostatic attraction	In ethanol / Recyclability	44

$[Cu_6(AcNTB)_6]\cdot 6ClO_4\cdot 38H_2O$	—	46 mg/g	KI aqueous solution	ion-exchange	In ethanol	45
		41.5 mg/g	I_2/KI aqueous solution			
Lac-Zn polycrystalline film	227 m^2/g	31.72 mg/g	iodine aqueous solution	physical and chemical adsorption	In ethanol / Recyclability	46

4.5 Crystallographic data

Supplementary Table 10. Crystallographic data and structure refinement parameters for **AlOC-60, AlMC-1** and **AlMC-2**.

	AlOC-60	AlMC-1	AlMC-2
Empirical formula	$C_{74}H_{94}Al_8O_{33}$	$C_{136}H_{228}Al_{24}N_4O_{96}$	$C_{168}H_{292}Al_{24}N_4O_{96}$
Formula weight	1727.33	4102.73	4551.56
Temperature / K	293(2) K	149.99 K	293(2) K
Crystal system	tetragonal	Monoclinic	Triclinic
Space group	*P*4/nnc	*C*2/c	*P*-1
a [Å]	23.6200(15)	23.0926(5)	19.0134(4)
b [Å]	23.6200(15)	28.8050(7)	19.1840(3)
c [Å]	8.8479(6)	32.0457(6)	19.8749(3)
α [^{o}]	90	90	108.813(2)
Β [^{o}]	90	103.344(2)	112.023(2)
γ [^{o}]	90	90	98.225(2)
V [$Å^3$]	4936.3(7)	20740.7(8)	6066.7(2)
Z	2	4	1
ρ_{calcd} [g cm^{-3}]	1.162	1.314	1.246
μ [mm^{-1}]	0.901	1.183	1.042
F (000)	1812.0	8608.0	2408.0
Index ranges	–25 ≤ h ≤ 25<br>–16 ≤ k ≤ 26<br>–4 ≤ l ≤ 9	–24 ≤ h ≤ 25<br>–31 ≤ k ≤ 31<br>–35 ≤ l ≤ 21	–23 ≤ h ≤ 23<br>–23 ≤ k ≤ 23<br>–22 ≤ l ≤ 24
Reflections collected	11797	35879	121208
Independent reflections	1627 [R_{int} = 0.1000]	12435 [R_{int} = 0.0570]	24426 [R_{int} = 0.0549]
data/restraints/parameters	1627/40/143	12435/285/1257	24426/1631/1331
Goodness-of-fit on F^2	1.034	1.183	1.211
R_1, wR_2 [$I > 2\sigma(I)$]	0.0601, 0.1538	0.1060, 0.2768	0.0987, 0.2829
R_1, wR_2 [all data]	0.0837, 0.1713	0.1600, 0.3262	0.1258, 0.3210
Largest diff. Peak/hole/e $Å^{-3}$	0.28/-0.28	0.91/–0.52	0.80/–1.02
CCDC number	2193096	2193097	2193098

Supplementary Table 11. Crystallographic data and structure refinement parameters for **AlMC-3** to **AlMC-5**.

	**AlMC-3**	**AlMC-4**	**AlMC-5**
Empirical formula	$C_{144}H_{246}Al_{24}N_2O_{92}$	$C_{132}H_{212}Al_{24}Cl_3NO_{83}$	$C_{132}H_{212}Al_{24}Br_3NO_{83}$
Formula weight	4124.93	3894.88	4028.18
Temperature / K	100.00(14) K	99.98(12) K	100.00(10) K
Crystal system	Monoclinic	Triclinic	Cubic
Space group	$P2_1/n$	*P*-1	*I*a-3
a [Å]	20.0662(4)	19.2557(3)	39.1658(4)
b [Å]	19.4146(3)	19.9823(4)	39.1658(4)
c [Å]	28.2894(4)	34.8883(5)	39.1658(4)
α [^{o}]	90	80.275(2)	90
B [^{o}]	96.3760(10)	76.2980(10)	90
γ [^{o}]	90	66.060(2)	90
V [$Å^3$]	10952.7(3)	11879.9(4)	60078.8(18)
Z	2	2	8
ρ_{calcd} [g cm^{-3}]	1.251	1.089	0.891
μ [mm^{-1}]	1.110	1.191	1.089
F (000)	4344.0	4076.0	16736.0
	–21 ≤ h ≤ 20	–18 ≤ h ≤ 18	–25 ≤ h ≤ 42
Index ranges	–20 ≤ k ≤ 20	–19 ≤ k ≤ 19	–43 ≤ k ≤ 27
	–30≤ l ≤ 30	–33 ≤ l ≤ 33	–41 ≤ l ≤ 37
Reflections collected	47819	82182	24079
Independent reflections	13251 [R_{int} = 0.0367]	21932 [R_{int} = 0.0644]	5272 [R_{int} = 0.0331]
data/restraints/parameters	13251/209/1232	21932/358/2360	5272/92/365
Goodness-of-fit on F^2	1.222	1.222	1.241
R_1, wR_2 [$I > 2\sigma(I)$]	0.0996, 0.2733	0.1100, 0.2891	0.1002, 0.2857
R_1, wR_2 [all data]	0.1271, 0.3080	0.1424, 0.3219	0.1387, 0.3461
Largest diff. Peak/hole/e $Å^{-3}$	1.36/–0.63	1.22/–0.41	0.83/–0.30
CCDC number	2193099	2193100	2193101

Supplementary Table 12. Crystallographic data and structure refinement parameters for **AlMC-6** to **AlMC-8**.

	**AlMC-6**	**AlMC-7**	**AlMC-8**
Empirical formula	$C_{132}H_{212}Al_{24}I_4O_{80}$	$C_{171}H_{273}Al_{27}N_2O_{101}$	$C_{134}H_{217}Al_{24}Br_2NO_{84}$
Formula weight	4234.12	4701.36	3993.41
Temperature / K	293(2) K	100.00(10) K	116(20) K
Crystal system	Cubic	Trigonal	Monoclinic
Space group	*I*m-3m	*R*-3	$P2_1$/n
a [Å]	24.2781(7)	22.5474(4)	19.8943(4)
b [Å]	24.2781(7)	22.5474(4)	18.7946(4)
c [Å]	24.2781(7)	48.7676(6)	27.3905(5)
α [^{o}]	90	90	90
B [^{o}]	90	90	96.556(2)
γ [^{o}]	90	120	90
V [$Å^3$]	14299.0(12)	21471.1(5)	10174.5(4)
Z	2	3	2
ρ_{calcd} [g cm^{-3}]	0.983	1.091	1.304
μ [mm^{-1}]	3.079	0.956	1.459
F (000)	4336.0	7416.0	4164.0
Index ranges	–16 ≤ h ≤ 30<br>–25 ≤ k ≤ 21<br>–28 ≤ l ≤ 11	–26 ≤ h ≤ 25<br>–14 ≤ k ≤ 24<br>–57 ≤ l ≤ 46	–20 ≤ h ≤ 20<br>–18 ≤ k ≤ 19<br>–22 ≤ l ≤ 28
Reflections collected	8821	21578	36419
Independent reflections	1075 [R_{int} = 0.0828]	7969 [R_{int} = 0.0139]	11401 [R_{int} = 0.0553]
data/restraints/parameters	1075/34/71	7969/525/534	11401/342/1243
Goodness-of-fit on F^2	1.101	1.246	1.237
R_1, wR_2 [$I > 2\sigma(I)$]	0.1040, 0.2634	0.0954, 0.2644	0.1108, 0.3021
R_1, wR_2 [all data]	0.1583, 0.3280	0.1077, 0.2867	0.1524, 0.3377
Largest diff. Peak/hole/e $Å^{-3}$	0.87/–0.91	1.51/–0.54	1.19/-0.57
CCDC number	2193102	2193103	2193104

Supplementary Table 13. Crystallographic data and structure refinement parameters for **AlMC-1a, I@Al_{24}–400ppm–30min** and **I@Al_{24}–4ppm–48h**.

	**AlMC-1a**	**I@Al_{24}–400ppm–30min**	**I@Al_{24}–4ppm–48h**
Empirical formula	$C_{132}H_{216}Al_{24}O_{82}$	$C_{132}H_{216}Al_{24}I_{7.75}O_{82}$	$C_{132}H_{216}Al_{24}I_{0.52}O_{82}$
Formula weight	3762.56	4746.03	3828.54
Temperature / K	100.00(13) K	100.00(10) K	100.00(10) K
Crystal system	Monoclinic	Monoclinic	Monoclinic
Space group	*C*2/c	*C*2/c	*C*2/c
a [Å]	22.8063(3)	22.6436(15)	22.7815(6)
b [Å]	27.4541(4)	27.7544(14)	27.6685(7)
c [Å]	31.8602(3)	31.8697(11)	31.8874(6)
α [°]	90	90	90
Β [°]	103.7020(10)	102.758(5)	103.519(2)
γ [°]	90	90	90
V [$Å^3$]	19380.8(4)	19534.3(18)	19542.7(8)
Z	4	4	4
ρ_{calcd} [g cm^{-3}]	1.289	1.614	1.301
μ [mm^{-1}]	1.196	7.636	1.618
F (000)	7904.0	9547.0	8014.0
	–27 ≤ h ≤ 27	–22 ≤ h ≤ 21	–22 ≤ h ≤ 22
Index ranges	–32 ≤ k ≤ 32	–27 ≤ k ≤ 26	–27 ≤ k ≤ 27
	–37 ≤ l ≤ 30	–29 ≤ l ≤ 31	–31 ≤ l ≤ 32
Reflections collected	78884	16083	34300
Independent reflections	16367 [R_{int} = 0.0379]	7610 [R_{int} = 0.0468]	10338 [Rint: = 0.0318]
data/restraints/parameters	16367/66/1089	7610/376/1076	10338/665/1126
Goodness-of-fit on F^2	1.028	1.690	1.524
R_1, wR_2 [$I > 2\sigma(I)$]	0.0942, 0.2543	0.1681, 0.4096	0.1150, 0.3409
R_1, wR_2 [all data]	0.1256, 0.2962	0.2306, 0.4489	0.1381, 0.3651
Largest diff. Peak/hole/e $Å^{-3}$	1.04/–0.56	1.43/-0.91	1.37/-0.75
CCDC number	2193105	2193106	2193107

Supplementary Table 14. Crystallographic data and structure refinement parameters for **I@Al_{24}–40ppm–48h**, **I@Al_{24}–400ppm–48h**, and **I@Al_{24}–2000ppm–48h**.

	**I@Al_{24}–40ppm–48h**	**I@Al_{24}–400ppm–48h**	**I@Al_{24}–2000ppm–48h**
Empirical formula	$C_{132}H_{216}Al_{24}I_{2.5}O_{82}$	$C_{132}H_{212}Al_{24}I_{10}O_{80}$	$C_{132}H_{212}Al_{24}I_{15.5}O_{80}$
Formula weight	4079.81	4995.52	5693.47
Temperature / K	100.00(10) K	100.00(10) K	100.00(10) K
Crystal system	Monoclinic	Monoclinic	Monoclinic
Space group	*C*2/c	*C*2/c	*C*2/c
a [Å]	22.8262(7)	22.7207(6)	22.7608(6)
b [Å]	27.3383(11)	27.8382(6)	28.1528(7)
c [Å]	31.9138(8)	31.9530(6)	32.0483(9)
α [^{o}]	90	90	90
B [^{o}]	103.406(3)	102.807(2)	102.479(3)
γ [^{o}]	90	90	90
V [$Å^3$]	19372.5(11)	19707.6(8)	20050.8(9)
Z	4	4	4
ρ_{calcd} [g cm^{-3}]	1.399	1.684	1.886
μ [mm^{-1}]	3.294	9.417	14.068
F (000)	8434.0	9944.0	11110.0
	–22 ≤ h ≤ 22	–22 ≤ h ≤ 23	–22 ≤ h ≤ 22
Index ranges	–27 ≤ k ≤ 23	–28 ≤ k ≤ 28	–25 ≤ k ≤ 28
	–32 ≤ l ≤ 32	–32 ≤ l ≤ 32	–32 ≤ l ≤ 31
Reflections collected	31642	37302	28581
Independent reflections	10236 [R_{int} = 0.0440]	10468 [R_{int} = 0.0496]	9938 [R_{int} = 0.0651]
data/restraints/parameters	10236/151/1107	10468/443/1194	9938/472/1228
Goodness-of-fit on F^2	1.516	1.798	1.799
R_1, wR_2 [$I > 2\sigma(I)$]	0.1250, 0.3527	0.1627, 0.4016	0.1733, 0.4153
R_1, wR_2 [all data]	0.1548, 0.3824	0.1915, 0.4301	0.2038, 0.4434
Largest diff. Peak/hole/e $Å^{-3}$	1.30/–0.81	2.14/–1.61	2.00/–1.59
CCDC number	2193108	2193109	2193110

Supplementary Table 15. Crystallographic data and structure refinement parameters for **I@Al_{24}–100000ppm–48h**.

	**I@Al_{24}–100000ppm–48h**
Empirical formula	$C_{132}H_{212}Al_{24}I_{26.5}O_{80}$
Formula weight	7089.37
Temperature / K	100.00(10) K
Crystal system	Monoclinic
Space group	*C*2/c
a [Å]	22.9427(7)
b [Å]	28.1959(7)
c [Å]	32.0386(7)
α [°]	90
B [°]	102.267(2)
γ [°]	90
V [Å^3]	20252.2(9)
Z	4
ρ_{calcd} [g cm^{-3}]	2.325
μ [mm^{-1}]	22.409
F (000)	13442.0
	–23 ≤ h ≤ 14
Index ranges	–24 ≤ k ≤ 28
	–32 ≤ l ≤ 32
Reflections collected	29059
Independent reflections	9509 [R_{int} = 0.0604]
data/restraints/parameters	9509/653/1228
Goodness-of-fit on F^2	1.444
R_1, wR_2 [$I > 2\sigma(I)$]	0.1489, 0.3522
R_1, wR_2 [all data]	0.2015, 0.3970
Largest diff. Peak/hole/e Å^{-3}	1.03/–1.17
CCDC number	2193111

4.6 BVS analysis

Supplementary Table 16. BVS analysis for Al^{3+} and OH groups in **AlMC-1**.

**AlMC-1**	Al	Al1	3.131	Al2	3.126
		Al3	3.073	Al4	3.154
		Al5	3.114	Al6	3.102
		Al7	3.157	Al8	3.111
		Al9	3.112	Al10	3.124
		Al11	3.068	Al12	3.193
	μ_3-OH	O1	1.237	O2	1.244
		O4	1.235	O12	1.225
	μ_2-OH	O3	1.116	O5	1.098
		O6	0.950	O8	1.061
		O10	1.111	O11	1.051
		O13	1.107	O14	1.095
		O15	1.135	O16	1.072
		O20	1.095	O22	1.123

Supplementary Table 17. BVS analysis for Al^{3+} and OH groups in **AlMC-2**.

**AlMC-2**	Al	Al1	3.099	Al2	3.111
		Al3	3.131	Al4	3.149
		Al5	3.103	Al6	3.110
		Al7	3.109	Al8	3.113
		Al9	3.105	Al10	3.118
		Al11	3.127	Al12	3.089
	μ_3-OH	O5	1.211	O10	1.220
		O12	1.205	O25	1.215
	μ_2-OH	O3	1.087	O8	1.095
		O11	1.079	O16	1.103
		O18	1.156	O20	1.147
		O22	1.155	O24	1.097
		O27	1.092	O30	1.155
		O31	1.101	O37	1.108

Supplementary Table 18. BVS analysis for Al^{3+} and OH groups in **AlMC-3**.

**AlMC-3**	Al	Al1	3.109	Al2	3.138
		Al3	3.141	Al4	3.110
		Al5	3.119	Al6	3.148
		Al7	3.133	Al8	3.149
		Al9	3.150	Al10	3.125

		Al11	3.110	Al12	3.144
	μ_3-OH	O1	1.237	O2	1.221
		O5	1.247	O12	1.246
	μ_2-OH	O3	1.134	O4	1.105
		O6	1.094	O7	1.120
		O10	1.084	O11	1.143
		O13	1.145	O15	1.146
		O17	1.132	O20	1.140
		O27	1.151	O32	1.095

Supplementary Table 19. BVS analysis for Al^{3+} and OH groups in **AlMC-4**.

**AlMC-4**	Al	Al1	3.174	Al2	3.205
		Al3	3.191	Al4	3.140
		Al5	3.132	Al6	3.200
		Al7	3.189	Al8	3.229
		Al9	3.147	Al10	3.153
		Al11	3.166	Al12	3.231
		Al13	3.122	Al14	3.137
		Al15	3.181	Al16	3.167
		Al17	3.287	Al18	3.156
		Al19	3.176	Al20	3.129
		Al21	3.148	Al22	3.229
		Al23	3.178	Al24	3.177
	μ_3-OH	O2	1.287	O4	1.273
		O8	1.265	O19	1.262
		O35	1.231	O37	1.299
		O41	1.245	O42	1.275
	μ_2-OH	O1	1.168	O3	1.120
		O5	1.111	O6	1.111
		O7	1.101	O9	1.143
		O14	1.152	O15	1.169
		O16	1.120	O20	1.141
		O24	1.112	O36	1.157
		O44	1.158	O46	1.149
		O47	1.101	O48	1.125
		O50	1.136	O52	1.130
		O53	1.068	O54	1.114
		O55	1.147	O56	1.100
		O60	1.117	O62	1.081

Supplementary Table 20. BVS analysis for Al^{3+} and OH groups in **AlMC-5**.

**AlMC-5**	Al	Al1	3.236	Al2	3.236
		Al3	3.082	Al4	3.110
	μ_3-OH	O1	1.287	O3	1.273
	μ_2-OH	O2	1.102	O4	1.130
		O5	1.142	O6	1.100

Supplementary Table 21. BVS analysis for Al^{3+} and OH groups in **AlMC-6**.

**AlMC-6**	Al	Al1	3.127
	μ_3-OH	O2	1.269
	μ_2-OH	O1	1.117

Supplementary Table 22. BVS analysis for Al^{3+} and OH groups in **AlMC-7**.

**AlMC-7**	Al	Al1	3.128	Al2	3.158
		Al3	3.138	Al4	3.144
		Al5	2.732	Al6	2.732
	μ_3-OH	O1	1.236	O7	1.229
	μ_2-OH	O2	1.122	O3	1.135
		O5	1.127	O6	1.127

Supplementary Table 23. BVS analysis for Al^{3+} and OH groups in **AlMC-8**.

**AlMC-8**	Al	Al1	3.143	Al2	3.177
		Al3	3.156	Al4	3.180
		Al5	3.182	Al6	3.204
		Al7	3.204	Al8	3.232
		Al9	3.164	Al10	3.197
		Al11	3.190	Al12	3.229
	μ_3-OH	O1	1.249	O3	1.188
		O5	1.226	O7	1.211
	μ_2-OH	O2	1.142	O4	1.137
		O6	1.157	O8	1.120
		O9	1.106	O10	1.123
		O12	1.152	O13	1.147
		O18	1.117	O19	1.091
		O21	1.144	O29	1.166

4.7 Hydrogen bond parameters

Supplementary Table 24. Hydrogen bond parameters for **AlOC-60**.

D–H⋯A	d(D–H)	d(H⋯A)	d(D-A)	∠(D–H⋯A)
O2–H2⋯O5	0.92	2.00	2.857(4)	155

Supplementary Table 25. Hydrogen bond parameters for **AlMC-1**.

D–H⋯A	d(D–H)	d(H⋯A)	d(D-A)	∠(D–H⋯A)
O1–H1⋯O46	1.00	2.28	3.20(5)	153
O1–H1⋯O47	1.00	2.14	3.07(5)	154
O2–H2⋯O48	1.00	1.75	2.74(5)	167
O3–H3⋯O41^{i}	0.95	1.95	2.896(10)	173
O4–H4⋯O46	1.00	1.98	2.92(5)	156
C4–H4⋯O48	1.00	2.40	3.33(5)	154
O5–H5⋯ O44	0.95	1.99	2.936(8)	175
O6–H6⋯O41^{i}	0.95	1.95	2.897(9)	175
O8–H8⋯O45	0.95	2.26	3.186(17)	165
O10–H10⋯O44	0.95	1.92	2.865(7)	170
O11–H11⋯O44	0.95	1.94	2.882(7)	173
O12–H12⋯O47	1.00	1.80	2.76(5)	160
O12–H12⋯O46^{i}	1.00	2.60	3.51(6)	151
O13–H13⋯O44	0.95	1.96	2.909(7)	172
O14–H14⋯O41	0.95	1.96	2.902(8)	174
O15–H15⋯O41^{i}	0.95	1.96	2.910(7)	176
O16–H16⋯O45^{i}	0.95	2.41	3.353(14)	169
O20–H20⋯O45	0.95	2.04	2.952(14)	159
O22–H22⋯O45^{i}	0.95	2.21	3.125(15)	161
C16–H16A⋯O37	0.95	2.47	2.787(12)	100
C21–H21B⋯O29	0.99	2.53	2.925(14)	104
C22–H22A⋯O40	0.99	2.48	2.894(13)	105
C22–H22B⋯O9	0.99	2.39	2.835(13)	107
C23–H23⋯O27	0.95	2.44	2.770(13)	100
C24–24⋯O35	0.95	2.41	2.745(13)	101
C26–H26A⋯O21	0.99	2.54	2.962(12)	105
C34–H34A⋯O38	0.99	2.57	2.962(14)	103
C37–H37⋯O25	0.95	2.42	2.742(15)	100
C39–H39A⋯O25	0.99	2.45	2.870(13)	105
C39–H39B⋯O37	0.99	2.49	2.899(16)	105
C50–H50A⋯O39	0.99	2.48	2.898(17)	105
C50–H50B⋯O31^{i}	0.99	2.58	2.962(16)	103
C52–H52A⋯O25	0.99	2.31	2.86(3)	114
C52–H52B⋯O34	0.99	2.35	2.88(3)	113
C55–H55A⋯O35^{i}	0.99	2.40	2.86(3)	107

C55–H55B···O27	0.99	2.28	2.73(2)	106
C59–H59A···O37	0.99	2.52	2.926(16)	104
C59–H59B···O34	0.99	2.57	2.942(15)	102
C63–H63A···O38	0.99	2.57	2.96(3)	104
C63–H63B···O40	0.99	2.59	2.95(2)	101
C65–H65A···.29	0.99	2.27	2.85(2)	117
C65–H65B···O39	0.99	2.32	2.90(2)	116
C73–H73C···O38	0.99	2.59	3.15(5)	116
C74–H74A···O38	0.99	2.32	2.83(3)	111
C74–H74B···O40	0.99	2.46	2.98(3)	112

Symmetric code: (i) 1-x, 1-y, 1-z

Supplementary Table 26. Hydrogen bond parameters for **AlMC-2**.

D–H···A	d(D–H)	d(H···A)	d(D-A)	∠(D–H···A)
O3–H3···O42^{i}	0.93	2.13	3.044(6)	167
O5–H5···O48	0.98	2.17	3.111(14)	159
O5–H5···O46^{i}	0.98	2.06	3.000(16)	160
O8–H8···O45^{i}	0.93	2.07	2.987(7)	170
O10–H10···O48	0.98	1.97	2.925(14)	164
O11–H11···O42	0.93	2.11	3.022(8)	167
O12–H12···O47	0.98	1.90	2.853(18)	162
O16–H16···O45^{i}	0.93	2.10	3.025(7)	171
O18–H18···O41	0.93	1.99	2.911(5)	172
O20–H20···O41	0.93	2.01	2.933(5)	175
O22–H22···O41	0.93	2.00	2.929(5)	174
O24–H24···O45	0.93	2.11	3.029(6)	170
O25–H25···O47	0.98	2.18	3.103(19)	157
O25–H25···O46^{i}	0.98	2.15	3.085(17)	159
O27–H27···O42	0.93	2.12	3.034(6)	168
O30–H30···O41	0.93	2.03	2.954(5)	175
O31–H31···O45	0.93	2.09	3.005(7)	170
O37–H37···O42^{i}	0.93	2.11	3.027(8)	167
C14–H14···O13	0.93	2.44	2.753(9)	100
C23–H23···O38	0.93	2.45	2.765(8)	100
C30–H30A···O39	0.93	2.44	2.757(9)	100
C31–H31B···O32	0.97	2.55	2.949(8)	105
C34–H34A···O29	0.97	2.57	2.969(9)	105
C34–H34B···O28	0.97	2.57	2.925(9)	102
C38–H38A···O36	0.97	2.57	2.953(10)	103
C39–H39A···O36	0.97	2.55	2.912(11)	102
C39–H39B···O28	0.97	2.51	2.922(10)	105
C44–H44A···O38	0.97	2.57	2.936(10)	102

C44–H44B···O13	0.97	2.56	2.965(9)	105
C45–H45A···O35	0.97	2.44	2.870(12)	107
C45–H45B···O26	0.97	2.50	2.890(12)	104
C46–H46A···O34^{i}	0.97	2.51	2.890(12)	103
C46–H46B···O14	0.97	2.44	2.886(11)	107
C49–H49A···O40^{i}	0.97	2.55	2.928(12)	103
C49–H49B···O13	0.97	2.55	2.945(11)	105
C51–H51A···O39	0.97	2.41	2.858(12)	108
C55–H55A···O26	0.97	2.53	2.938(14)	105
C57–H57A···O32	0.97	2.52	2.905(11)	104
C57–H57B···O34^{i}	0.97	2.50	2.905(11)	105
C76–H76A···O38	0.97	2.44	2.824(13)	103

Symmetric code: (i) 1-x, 1-y, 1-z

Supplementary Table 27. Hydrogen bond parameters for **AlMC-3**.

D–H···A	d(D–H)	d(H···A)	d(D-A)	∠(D–H···A)
O1–H1···O43	1.00	1.97	2.915(16)	157
O1–H1···O48	1.00	2.12	3.080(3)	161
O1–H1···O44^{i}	1.00	2.03	2.990(17)	160
O2–H2···O42	1.00	2.13	3.082(19)	159
O2–H2···O44^{i}	1.00	2.01	2.970(18)	161
O2–H2···O47^{i}	1.00	2.20	2.900(3)	125
O2–H2···O49^{i}	1.00	2.25	3.040(6)	135
O3–H3···O47^{i}	1.00	2.13	2.840(3)	126
O3–H3···O48^{i}	1.00	2.14	3.110(3)	163
O3–H3···O49^{i}	1.00	2.53	2.370(8)	137
O3–H3···O50^{i}	1.00	2.09	3.050(3)	160
O4–H4···O43	1.00	2.01	2.960(17)	157
O4–H4···O42	1.00	2.00	2.969(19)	161
O4–H4···O50	1.00	2.03	2.990(3)	159
O5–H5···O45	0.95	1.98	2.934(7)	176
O6–H6···O41	0.95	1.96	2.912(7)	178
O8–H8···O41	0.95	1.96	2.909(7)	175
O9–H9···O46^{i}	0.95	1.99	2.938(7)	172
O11–H11···O45^{i}	0.95	1.93	2.877(7)	174
O12–H12···O46	0.95	1.97	2.910(7)	172
O14–H14···O46^{i}	0.95	1.97	2.912(7)	172
O16–H16···O45	0.95	1.97	2.917(7)	174
O18–H18···O41	0.95	1.97	2.923(7)	178
O21–H21···O41	0.95	1.88	2.822(7)	174
O23–H23···O45^{i}	0.95	1.93	2.870(7)	172
O30–H30···O46	0.95	1.99	2.932(7)	173

C26–H26A⋯O27	0.99	2.58	2.939(12)	101
C26–H26B⋯O26	0.99	2.60	2.968(10)	102
C37–H37B⋯O22	0.99	2.55	2.948(12)	104
C43–H43A⋯O26	0.99	2.47	2.908(13)	106
C45–H45A⋯O22	0.99	2.54	2.945(13)	104
C48–H48A⋯O27	0.99	2.35	2.873(13)	112
C48–H48B⋯O38	0.99	2.16	2.759(12)	117
C57–H57A⋯O32^{i}	0.99	2.53	2.952(18)	105
C58–H58A⋯O35	0.99	2.32	2.870(3)	114
C59–H59A⋯O37	0.99	2.52	2.944(15)	105
C59–H59B⋯O33	0.99	2.57	2.910(16)	100
C61–H61A⋯O7^{i}	0.99	2.50	2.870(3)	102
C61–H61B⋯O39	0.99	2.47	2.860(3)	103
C62–H62A⋯O33	0.99	2.37	2.903(15)	113
C65–H65A⋯O29	0.99	2.48	2.920(13)	106
C65–H65B⋯O37	0.99	2.49	2.903(14)	104
C69–H69B⋯O21	0.98	2.48	3.450(3)	169
C72–H72C⋯O32^{i}	0.98	2.15	2.720(6)	116

Symmetric code: (i) 1-x, 1-y, 1-z

Supplementary Table 28. Hydrogen bond parameters for **AlMC-4**.

D–H⋯A	d(D–H)	d(H⋯A)	d(D-A)	∠(D–H⋯A)
O1–H1⋯Cl3^{i}	0.95	2.33	3.263(7)	167
O2–H2⋯O84	1.00	2.09	3.040(6)	158
O2–H2⋯O85^{i}	1.00	2.21	3.150(6)	156
O3–H3⋯Cl3	0.95	2.28	3.201(7)	163
O4–H4⋯O84	1.00	2.09	3.040(4)	157
O4–H4⋯O86^{i}	1.00	2.50	3.430(6)	153
O5–H5⋯Cl2	0.95	2.27	3.190(7)	163
O6–H6⋯Cl2	0.95	2.29	3.208(7)	163
O7–H7⋯Cl2	0.95	2.27	3.196(8)	164
O8–H8⋯O85	1.00	2.49	3.420(6)	154
O8–H8⋯O86	1.00	2.36	3.280(6)	154
O9–H9⋯Cl3^{i}	0.95	2.32	3.243(6)	163
O14–H14⋯Cl3	0.95	2.32	3.249(7)	165
O15–H15⋯Cl1^{i}	0.95	2.33	3.256(6)	166
O16–H16⋯Cl1	0.95	2.29	3.214(7)	165
O19–H19⋯O85	1.00	2.40	3.330(5)	155
O19–H19⋯O86^{i}	1.00	2.18	3.120(6)	156
O20–H20⋯Cl1	0.95	2.28	3.205(6)	165
O24–H24⋯Cl2	0.95	2.34	3.260(7)	165
O35–H35⋯O82^{ii}	1.00	2.07	3.040(6)	162

O35–H35···O83[ii]	1.00	2.46	3.380(6)	153
O36–H36A···Cl1	0.95	2.27	3.192(6)	163
O37–H37···O82	1.00	2.06	3.040(6)	163
O37–H37···O83[ii]	1.00	2.39	3.310(5)	152
O41–H41···O81	1.00	1.96	2.910(4)	160
O41–H41···O83[ii]	1.00	2.31	3.230(5)	153
O42–H42···O81[ii]	1.00	2.05	3.000(4)	159
O42–H42···O83[ii]	1.00	2.52	3.440(5)	152
O44–H44···Cl5	0.95	2.27	3.201(8)	167
O46–H46···Cl4	0.95	2.29	3.218(8)	165
O47–H47···Cl4[ii]	0.95	2.26	3.186(8)	164
O48–H48···Cl4	0.95	2.28	3.209(8)	166
O50–H50···Cl5	0.95	2.24	3.165(8)	165
O52–H52···Cl5	0.95	2.27	3.197(8)	164
O53–H53···Cl6	0.95	2.22	3.149(10)	167
O54–H54···Cl5	0.95	2.21	3.141(7)	166
O55–H55···Cl4[ii]	0.95	2.30	3.232(8)	167
O56–H56···Cl6[ii]	0.95	2.23	3.162(9)	168
O60–H60···Cl6[ii]	0.95	2.20	3.131(10)	166
O62–H62···Cl6	0.95	2.20	3.132(10)	168
C103–H10B···O65	0.99	2.42	2.820(3)	104
C104–H10D···O65	0.99	2.57	2.920(2)	100
C106–H10I···O49	0.99	2.36	2.840(3)	109
C120–H12A···O61	0.99	2.58	2.940(2)	102
C140–H14B···O17	0.99	2.57	2.950(3)	102
C18–H18···O11	0.95	2.40	2.728(14)	100
C20–H20A···O39	0.99	2.60	2.950(15)	101
C20–H20B···O18	0.99	2.58	2.952(15)	102
C24–H24A···O25	0.95	2.41	2.739(13)	100
C25–H25···O49	0.95	2.33	2.691(19)	102
C36–H36···O65	0.95	2.41	2.739(19)	100
C41–H41A···O21	0.99	2.57	2.948(16)	103
C41–H41B···O45	0.99	2.51	2.900(2)	103
C42–H42A···O39	0.95	2.41	2.751(19)	101
C44–H44A···O45	0.99	2.52	2.905(15)	103
C44–H44B···O30	0.99	2.60	2.946(14)	101
C49–H49···O45	0.95	2.42	2.745(14)	100
C54–H54A···O22	0.99	2.47	2.875(18)	104
C54–H54B···O39	0.99	2.50	2.883(17)	103
C55–H55A···O34	0.99	2.54	2.915(17)	102
C55–H55B···O11	0.99	2.42	2.846(15)	105
C57–H57B···O21	0.99	2.44	2.866(18)	106

C58–H58B···O73	0.99	2.50	2.880(2)	103
C64–H64A···O57	0.99	2.57	2.950(2)	102
C64–H64B···O75^{ii}	0.99	2.55	2.920(2)	102
C65–H65B···O59	0.99	2.53	2.940(2)	105
C73–H73A···O28^{i}	0.99	2.51	2.920(2)	105
C75–H75A···O17	0.99	2.44	2.857(15)	105
C80–H80···O40	0.95	2.40	2.727(16)	100
C86–H86A···O59	0.99	2.54	2.920(19)	103
C86–H86B···O77	0.99	2.56	2.920(18)	101
C88–H88A···O18	0.99	2.58	2.970(3)	103
C88–H88B···O22	0.99	2.46	2.850(3)	103
C89–H89B···O57	0.99	2.39	2.830(3)	106
C94–H94B···O79	0.99	2.43	2.880(2)	107
C95–H95C···O77	0.98	2.53	3.110(2)	118
C96–H96B···O73	0.98	2.40	3.050(3)	123
C99–H99···O75	0.95	2.43	2.750(3)	100
C101–H101···O70	0.95	2.30	2.650(3)	101
C114–H114···O77	0.95	2.46	2.820(3)	102
C131–H131···O73	0.95	2.43	2.780(3)	102
C143–H143···O79	0.95	2.38	2.760(2)	103

Symmetric code: (i) 1-x, 2-y, -z; (ii) 2-x, 1-y, 1-z

Supplementary Table 29. Hydrogen bond parameters for **AlMC-5**.

D–H···A	d(D–H)	d(H···A)	d(D-A)	∠(D–H···A)
O1–H1···O15	1.00	1.99	2.940(2)	158
O1–H1···O15^{iii}	1.00	1.99	2.940(2)	158
O1–H1···O15^{i}	1.00	1.99	2.940(2)	158
O2–H2···Br1	0.95	2.50	3.404(5)	158
O3–H3···O16	1.00	1.92	2.860(3)	157
O3–H3···O15^{iii}	1.00	2.02	2.960(2)	157
O3–H3···O16^{iv}	1.00	2.01	2.950(3)	155
O4–H4···Br1^{iii}	0.95	2.50	3.408(5)	159
O5–H5···Br1	0.95	2.55	3.455(5)	160
O6–H6···Br1^{ii}	0.95	2.48	3.391(5)	160
C15–H15A···O14	0.99	2.59	2.972(11)	103
C17–H17A···O10	0.99	2.53	2.890(13)	101
C17–H17B···O10	0.99	2.58	2.960(13)	103
C20–H20B···O12^{ii}	0.99	2.40	2.845(17)	107
C21–H21A···O14	0.99	2.50	2.920(13)	105

Symmetric code: (i) 1/2-y, 1-z, 1/2+x; (ii) -1/2+y, z, 1/2-x; (iii) -1/2+z, 1/2-x, 1-y; (iv) 1/2-z, 1/2+x, y

Supplementary Table 30. Hydrogen bond parameters for **AlMC-6**.

D–H⋯A	d(D–H)	d(H⋯A)	d(D-A)	∠(D–H⋯A)
O1–H1⋯I2	0.90	2.83	3.728(6)	177
O2–H2⋯I1	0.91	3.04	3.943(5)	180
C3–H3A⋯O4	0.97	2.58	2.951(11)	103
C3–H3B⋯O4^{i}	0.99	2.56	2.951(11)	103
C4–H4⋯O4	0.93	2.40	2.742(10)	102
Symmetric code: (i) z, y, x				

Supplementary Table 31. Hydrogen bond parameters for **AlMC-7**.

D–H⋯A	d(D–H)	d(H⋯A)	d(D-A)	∠(D–H⋯A)
O1–H1⋯O21^{iii}	1.00	2.02	2.978(13)	159
O1–H1⋯O20^{iv}	1.00	1.99	2.940(13)	158
O1–H1⋯O21^{iv}	1.00	2.01	2.965(11)	159
O2–H2⋯O15	0.95	1.97	2.915(4)	174
O3–H3⋯O15^{ii}	0.95	1.95	2.898(4)	173
O5–H5⋯O15^{i}	0.95	1.96	2.906(5)	174
O6–H6⋯O15	0.95	2.02	2.967(4)	176
O7–H7⋯O20^{iv}	1.00	2.02	2.966(11)	158
O7–H7⋯O20^{ii}	1.00	2.02	2.966(11)	158
O7–H7⋯O20^{v}	1.00	2.02	2.965(11)	158
C5–H5B⋯O9	0.99	2.60	2.952(5)	101
C9–H9A⋯O14	0.99	2.55	2.904(7)	101
C9–H9B⋯O9^{ii}	0.99	2.57	2.960(7)	103
C12–H12A⋯O12^{i}	0.99	2.57	2.921(8)	101
C12–H12B⋯O12	0.99	2.51	2.924(8)	105
C15–H15A⋯O11	0.99	2.55	2.915(7)	102
C15–H15B⋯O14	0.99	2.49	2.905(8)	105
C31–H31A⋯O16	0.99	2.55	3.030(2)	109
Symmetric code: (i) -x+y, 1-x, z; (ii) -1/3+y, 1/3-x+y, 4/3-z; (iii) 1-y, 1+x-y, z; (iv) 2/3-x, 4/3-y, 4/3-z; (v) 2/3+x-y, 1/3+x, 4/3-z				

Supplementary Table 32. Hydrogen bond parameters for **AlMC-8**.

D–H···A	d(D–H)	d(H···A)	d(D-A)	∠(D–H···A)
O1–H1···O42	1.00	1.95	2.910(6)	159
O1–H1···O43	1.00	2.52	3.460(8)	156
O1–H1···O44^{i}	1.00	2.25	3.200(6)	156
O2–H2···O41	0.95	2.32	3.233(13)	161
O3–H3···O42	1.00	2.40	3.320(7)	152
O3–H3···O43^{i}	1.00	2.30	3.240(8)	155
O3–H3···O44^{i}	1.00	2.39	3.320(7)	155
O4–H4···O41	0.95	2.31	3.227(13)	161
O5–H5···O44	1.00	2.36	3.310(8)	156
O5–H5···O43^{i}	1.00	2.28	3.220(6)	157
O6–H6···O41	0.95	2.30	3.213(12)	160
O7–H7···O42	1.00	2.51	3.430(6)	153
O7–H7···O43	1.00	2.56	3.490(8)	155
O8–H8···Br1^{i}	0.95	2.42	3.313(6)	156
O9–H9···Br2	0.95	2.34	3.247(7)	159
O10–H10···Br1	0.95	2.38	3.290(6)	160
O12–H12···Br1^{i}	0.95	2.47	3.395(6)	164
O13–H13···Br1^{i}	0.95	2.46	3.374(6)	162
O18–H18···Br2	0.95	2.41	3.328(6)	163
O19–H19···Br2^{i}	0.95	2.27	3.182(6)	160
O21–H21···O41	0.95	2.31	3.231(11)	163
O29–H29···Br2^{i}	0.95	2.39	3.311(6)	163
C11–H11···O38	0.95	2.45	2.771(12)	100
C23–H23···O37	0.95	2.36	2.699(14)	100
C36–H36B···O15	0.99	2.42	2.864(16)	107
C37–H37···O35	0.95	2.49	2.870(2)	104
C38–H38A···O30	0.99	2.58	2.964(15)	103
C38–H38B···O40	0.99	2.39	2.825(15)	106
C41–H41A···O37	0.99	2.53	2.901(17)	102
C41–H41B···O32	0.99	2.56	2.945(15)	103
C49–H49···Br2^{ii}	0.95	2.86	3.580(2)	134
C51–H51A···O39	0.99	2.39	2.800(2)	104
C51–H51B···O23	0.99	2.56	2.930(2)	102
C54–H54A···O32	0.99	2.57	2.931(19)	102
C55–H55A···O37	0.99	2.18	2.750(3)	115
C55–H55B···O34	0.99	2.34	2.920(3)	116
C56–H56A···O40	0.99	2.41	2.854(17)	106
C56–H56B···O38^{i}	0.99	2.55	2.900(17)	100
C57–H57A···O36	0.99	2.55	2.930(2)	103

C57–H57B···O33[i]	0.99	2.37	2.840(2)	108
C58–H58A···O33[i]	0.99	2.34	2.800(2)	107
C59–H59B···O36	0.99	2.58	2.930(2)	101
C61–H61B···O41	0.98	2.14	3.030(3)	151
C63–H63A···O15	0.99	2.44	2.830(2)	103
C63–H63B···O39	0.99	2.44	2.850(2)	104
C80–H80A···O33[i]	0.98	2.57	3.160(3)	119

Symmetric code: (i) 1-x, 1-y, 1-z; (ii) 3/2-x, -1/2+y, 1/2-z

5. Supplementary References

(1) Allouche, L.; Gerardin, C.; Loiseau, T.; Ferey, G.; Taulelle, F. Al_{30}: A giant aluminum polycation. *Angew. Chem. Int. Ed.* **2000**, *39*, 511-514.

(2) Johansson, G. On the crystal structures of some basic aluminium salts. *Acta Chem. Scand.* **1960**, *14*, 771-773.

(3) Baes, C. F.; Mesmer, R. E. The hydrolysis of cations. *John Wiley and Sons: New York, NY* **1976**,

(4) Anseau, M. R.; Leung, J. P.; Sahai, N.; Swaddle, T. W. Interactions of Silicate Ions with Zinc(II) and Aluminum(III) in Alkaline Aqueous Solution. *Inorg. Chem.* **2005**, *44*, 8023−8032.

(5) Swaddle Thomas, W.; Rosenqvist, J.; Yu, P.; Bylaska, E.; Phillips Brian, L.; Casey William, H. Kinetic Evidence for Five-Coordination in $AlOH(aq)^{2+}$ Ion. *Science* **2005**, *308*, 1450-1453.

(6) Casey, W. H. Large aqueous aluminum hydroxide molecules. *Chem. Rev.* **2006**, *106*, 1-16.

(7) Son, J. H.; Kwon, Y. U. Crystal engineering through face interactions between tetrahedral and octahedral building blocks: crystal structure of $[\varepsilon\text{-}Al_{13}O_4(OH)_{24}(H_2O)_{12}]_2[V_2W_4O_{19}]_3(OH)_2 \cdot 27H_2O$. *Inorg. Chem.* **2004**, *43*, 1929-1932.

(8) Abeysinghe, S.; Corum, K. W.; Neff, D. L.; Mason, S. E.; Forbes, T. Z. Contaminant adsorption on nanoscale particles: structural and theoretical characterization of Cu^{2+} bonding on the surface of Keggin-type polyaluminum (Al_{30}) molecular species. *Langmuir* **2013**, *29*, 14124-14134.

(9) Abeysinghe, S.; Unruh, D. K.; Forbes, T. Z. Crystallization of Keggin-Type Polyaluminum Species by Supramolecular Interactions with Disulfonate Anions. *Cryst. Growth Des.* **2012**, *12*, 2044-2051.

(10) Sun, Z.; Wang, H.; Tong, H.; Sun, S. A giant polyaluminum species S-Al_{32} and two aluminum polyoxocations involving coordination by sulfate ions S-Al_{32} and S-K-Al_{13}. *Inorg. Chem.* **2011**, *50*, 559-564.

(11) Shohel, M.; Bjorklund, J. L.; Smith, J. A.; Kravchuk, D. V.; Mason, S. E.; Forbes, T. Z. Formation of Nanoscale $[Ge_4O_{16}Al_{48}(OH)_{108}(H_2O)_{24}]^{20+}$ from Condensation of ε-$GeAl_{12}^{8+}$ Keggin Polycations. *Angew. Chem. Int. Ed.* **2021**, *60*, 8755-8759.

(12) Casey, W. H.; Olmstead, M. M.; Phillips, B. L. A new aluminum hydroxide octamer, $[Al_8(OH)_{14}(H_2O)_{18}](SO_4)_5 \cdot 16H_2O$. *Inorg. Chem.* **2005**, *44*, 4888-4890.

(13) Jin, X.; Yang, W.; Tang, J.; Yan, Y.; Shi, W.; Bi, S. Insight into the structural characteristics of core-links and flat-aluminum tridecamers: a density functional theory study. *Dalton Trans.* **2012**, *41*, 1027-1032.

(14) Schmitt, W.; Baissa, E.; Mandel, A.; Anson, C. E.; Powell, A. K. $[Al_{15}(\mu_3\text{-}O)_4(\mu_3\text{-}OH)_6(\mu\text{-}OH)_{14}(hpdta)_4]^{3-}$—A New Al_{15} Aggregate Which Forms a Supramolecular Zeotype. *Angew. Chem. Int. Ed.* **2001**, *40*, 3577-3581.

(15) Geng, L.; Liu, C. H.; Wang, S. T.; Fang, W. H.; Zhang, J. Designable Aluminum Molecular Rings: Ring Expansion and Ligand Functionalization. *Angew.Chem. Int. Ed.* **2020**, *59*, 16735-16740.

(16) Li, Y.; Zheng, C.; Wang, S. T.; Liu, Y. J.; Fang, W. H.; Zhang, J. Record Aluminum Molecular Rings for Optical Limiting and Nonlinear Optics. *Angew. Chem. Int. Ed.* **2022**, *61*, e202116563.

(17) Huo, Q.; Margolese, D. I.; Ciesla, U.; Feng, P.; Gier, T. E.; Sieger, P.; Leon, R.; Petroff, P. M.; Schüth, F.; Stucky, G. D. Generalized synthesis of periodic surfactant/inorganic composite materials. *Nature* **1994**, *368*, 317-321.

(18) Wang, Q. M.; Lin, Y. M.; Liu, K. G. Role of Anions Associated with the Formation and Properties of Silver Clusters. *Acc. Chem. Res.* **2015**, *48*, 1570-1579.

(19) Chen, X.-M.; Aubin, S. M. J.; Wu, Y.-L.; Yang, Y.-S.; Mak, T. C. W.; Hendrickson, D. N. Polynuclear $Cu^{II}_{12}M^{III}_6$ (M = Y, Nd, or Gd) Complexes Encapsulating a ClO_4^- Anion: $[Cu_{12}M_6(OH)_{24}(H2O)_8(pyb)_{12}(ClO_4)](ClO_4)_{17} \cdot nH_2O$ (Pyb = Pyridine Betaine). *J. Am. Chem. Soc.* **1995**, *117*, 9600—9601.

(20) Zheng, X. Y.; Jiang, Y. H.; Zhuang, G. L.; Liu, D. P.; Liao, H. G.; Kong, X. J.; Long, L. S.; Zheng, L. S. A Gigantic Molecular Wheel of {Gd_{140}}: A New Member of the Molecular Wheel Family. *J. Am. Chem. Soc.* **2017**, *139*, 18178-18181.
(21) Zheng, X.-Y.; Xie, J.; Kong, X.-J.; Long, L.-S.; Zheng, L.-S. Recent advances in the assembly of high-nuclearity lanthanide clusters. *Coord. Chem. Rev.* **2019**, *378*, 222-236.
(22) Zhao, D.; Feng, J.; Huo, Q.; Melosh, N.; Fredrickson Glenn, H.; Chmelka Bradley, F.; Stucky Galen, D. Triblock Copolymer Syntheses of Mesoporous Silica with Periodic 50 to 300 Angstrom Pores. *Science* **1998**, *279*, 548-552.
(23) Mizuno, K.; Mura, T.; Uchida, S. Control of Polymorphisms and Functions in All-Inorganic Ionic Crystals Based on Polyaluminum Hydroxide and Polyoxometalates. *Cryst. Growth Des.* **2016**, *16*, 4968-4974.
(24) Ge, C. Y.; Hou, J. L.; Zhou, Z. Y.; Zhu, Q. Y.; Dai, J. A Cyclic Titanium-Oxo Cluster with a Tetrathiafulvalene Connector as a Precursor for Highly Efficient Adsorbent of Cationic Dyes. *Inorg Chem* **2022**, *61*, 486-495.
(25) Gao, M. Y.; Wang, F.; Gu, Z. G.; Zhang, D. X.; Zhang, L.; Zhang, J. Fullerene-like Polyoxotitanium Cage with High Solution Stability. *J. Am. Chem. Soc.* **2016**, *138*, 2556-2559.
(26) Liu, Y. J.; Li, Q. H.; Li, D. J.; Zhang, X. Z.; Fang, W. H.; Zhang, J. Designable Al_{32} -Oxo Clusters with Hydrotalcite-like Structures: Snapshots of Boundary Hydrolysis and Optical Limiting. *Angew. Chem. Int. Ed.* **2021**, *60*, 4849-4854.
(27) Gao, J.; Yan, J.; Beeg, S.; Long, D. L.; Cronin, L. Assembly of molecular "layered" heteropolyoxometalate architectures. *Angew. Chem. Int. Ed.* **2012**, *51*, 3373-3376.
(28) Geng, L.; Li, Q. H.; Wang, S. T.; Liu, Y. J.; Fang, W. H.; Zhang, J. Aluminium nanorings: configuration deformation and structural transformation. *Chem. Commun.* **2021**, *57*, 2085-2088.
(29) Zhang, Y.; Li, Q.-H.; Fang, W.-H.; Zhang, J. Aluminum molecular rings bearing amino-polyalcohol for iodine capture. *Inorg. Chem. Front.* **2022**, *9*, 592-598.
(30) James, D. W.; Kimber, G. M. Studies of anhydrous metal nitrates V.* vibrational spectra of $In(NO_3)_3$ and Pd(bipy)$(NO_3)_2$. *Aust. J. Chem.* **1970**, *23*, 829-831.
(31) Kim, M. K.; Jo, V.; Ok, K. M. New variant of highly symmetric layered perovskite with coordinated NO_3^- ligand: hydrothermal synthesis, structure, and characterization of $Cs_2PbCl_2(NO_3)_2$. *Inorg. Chem.* **2009**, *48*, 7368-7372.
(32) Zhao, J.-W.; Wang, C.-M.; Zhang, J.; Zheng, S.-T.; Yang, G.-Y. Combination of Lacunary Polyoxometalates and High-Nuclear Transition Metal Clusters under Hydrothermal Conditions: IX. A Series of Novel Polyoxotungstates Sandwiched by Octa-Copper Clusters. *Chem. Eur. J.* **2008**, *14*, 9223-9239.
(33) Li, X.-Z.; Zhou, L.-P.; Hu, S.-J.; Cai, L.-X.; Guo, X.-Q.; Wang, Z.; Sun, Q.-F. Metal ion adaptive self-assembly of photoactive lanthanide-based supramolecular hosts. *Chem. Commun.* **2020**, *56*, 4416-4419.
(34) Li, X.-Z.; Zhou, L.-P.; Yan, L.-L.; Yuan, D.-Q.; Lin, C.-S.; Sun, Q.-F. Evolution of Luminescent Supramolecular Lanthanide $M_{2n}L_{3n}$ Complexes from Helicates and Tetrahedra to Cubes. *J. Am. Chem. Soc.* **2017**, *139*, 8237-8244.
(35) Kieffer, M.; Bilbeisi, R. A.; Thoburn, J. D.; Clegg, J. K.; Nitschke, J. R. Guest Binding Drives Host Redistribution in Libraries of $Co^{II}_4L_4$ Cages. *Angew. Chem. Int. Ed.* **2020**, *59*, 11369-11373.
(36) Luo, D.; Zhou, X.-P.; Li, D. Beyond molecules: mesoporous supramolecular frameworks self-assembled from coordination cages and inorganic anions. *Angew. Chem. Int. Ed.* **2015**, *54*, 6190-6195.
(37) Xie, L.; Zheng, Z.; Lin, Q.; Zhou, H.; Ji, X.; Sessler, J. L.; Wang, H. Calix[4]pyrrole-based Crosslinked Polymer Networks for Highly Effective Iodine Adsorption from Water. *Angew. Chem. Int. Ed.* **2022**, *61*, e202113724.
(38) Yang, M.; Qiu, F.; ES, M. E.-S.; Wang, W.; Du, S.; Su, K.; Yuan, D. Water-stable hydrazone-linked porous organic cages. *Chem. Sci.* **2021**, *12*, 13307-13315.
(39) An, D.; Li, L.; Zhang, Z.; Asiri, A. M.; Alamry, K. A.; Zhang, X. Amino-bridged covalent organic Polycalix[4]arenes for ultra efficient adsorption of iodine in water. *Mater. Chem. Phys.* **2020**, *239*, 122328.
(40) Lin, Y.; Jiang, X.; Kim, S. T.; Alahakoon, S. B.; Hou, X.; Zhang, Z.; Thompson, C. M.; Smaldone, R. A.; Ke, C. An Elastic Hydrogen-Bonded Cross-Linked Organic Framework for Effective Iodine Capture in Water. *J. Am. Chem. Soc.* **2017**, *139*, 7172-7175.
(41) Gogia, A.; Das, P.; Mandal, S. K. Tunable Strategies Involving Flexibility and Angularity of Dual Linkers for a 3D Metal-Organic Framework Capable of Multimedia Iodine Capture. *ACS Appl. Mater. Interfaces* **2020**, *12*, 46107-46118.
(42) Liu, Q.-K.; Ma, J.-P.; Dong, Y.-B. Highly efficient iodine species enriching and guest-driven tunable luminescent properties based on a cadmium(II)-triazole MOF. *Chem. Commun.* **2011**, *47*, 7185-7187.

(43) Xu, W.-Q.; Li, Y.-H.; Wang, H.-P.; Jiang, J.-J.; Fenske, D.; Su, C.-Y. Face-Capped M^4L_4 Tetrahedral Metal-Organic Cage: Iodine Capture and Release, Ion Exchange, and Electrical Conductivity. *Chem. Asian J.* **2016**, *11*, 216-220.
(44) Long, X.; Chen, Y.-S.; Zheng, Q.; Xie, X.-X.; Tang, H.; Jiang, L.-P.; Jiang, J.-T.; Qiu, J.-H. Removal of iodine from aqueous solution by PVDF/ZIF-8 nanocomposite membranes. *Sep. Purif. Technol.* **2020**, *238*, 116488.
(45) Fu, L.; Liu, Y.; Pan, M.; Kuang, X.-J.; Yan, C.; Li, K.; Wei, S.-C.; Su, C.-Y. Accumulation of versatile iodine species by a porous hydrogen-bonding Cu(II) coordination framework. *J. Mater. Chem. A* **2013**, *1*, 8575-8580.
(46) Xu, L.; Zheng, Q.; Wang, Y.; Jiang, L.; Jiang, J.; Qiu, J. A pillared double-wall metal-organic framework adsorption membrane for the efficient removal of iodine from solution. *Sep. Purif. Technol.* **2021**, *274*, 118436.
