# Supplementary material for: Water-stable porous Al24 Archimedean solids for removal of trace iodine
Source: Nat Commun. 2022 Nov 4;13:6632. doi: 10.1038/s41467-022-34296-4 (PMC9636137; doi:10.1038/s41467-022-34296-4)
Supplement: Supplementary file 3 — Description of Additional Supplementary Files [file 41467_2022_34296_MOESM3_ESM.pdf]

Description of Additional Supplementary Files

File Name: Supplementary Data 1
Description: Crystallographic data

File Name: Supplementary Movie 1
Description: Archimedean Al_{24} molecular structrue

File Name: Supplementary Movie 2
Description: Archimedean Al_{24} structrue model

File Name: Supplementary Movie 3
Description: Fast Removal of Trace Iodine by Al_{24} Archimedean Solids
