# Supplementary figures and images for: Water-stable porous Al24 Archimedean solids for removal of trace iodine
Source: Nat Commun. 2022 Nov 4;13:6632. doi: 10.1038/s41467-022-34296-4 (PMC9636137; doi:10.1038/s41467-022-34296-4)

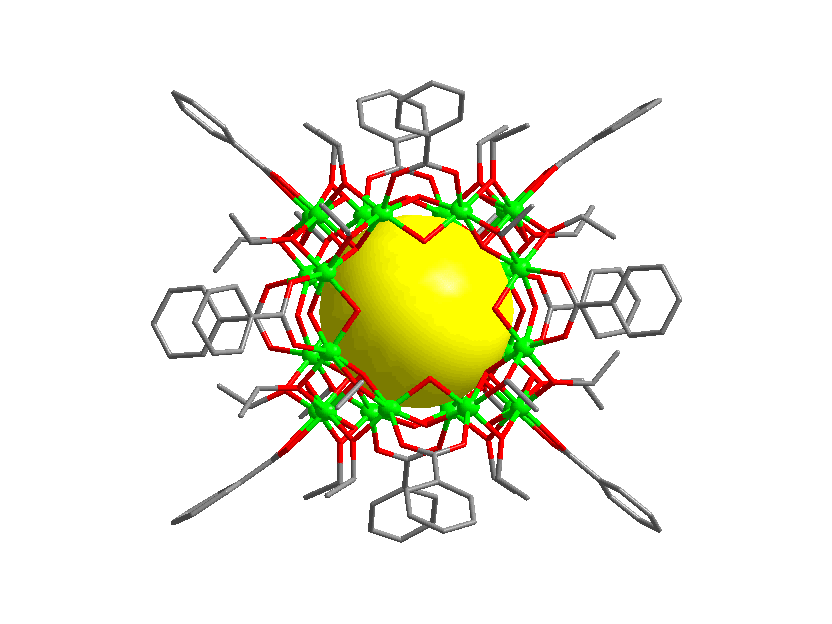

Supplement: Supplementary file 5 — Supplementary Movie 1 [file 41467_2022_34296_MOESM5_ESM.gif]

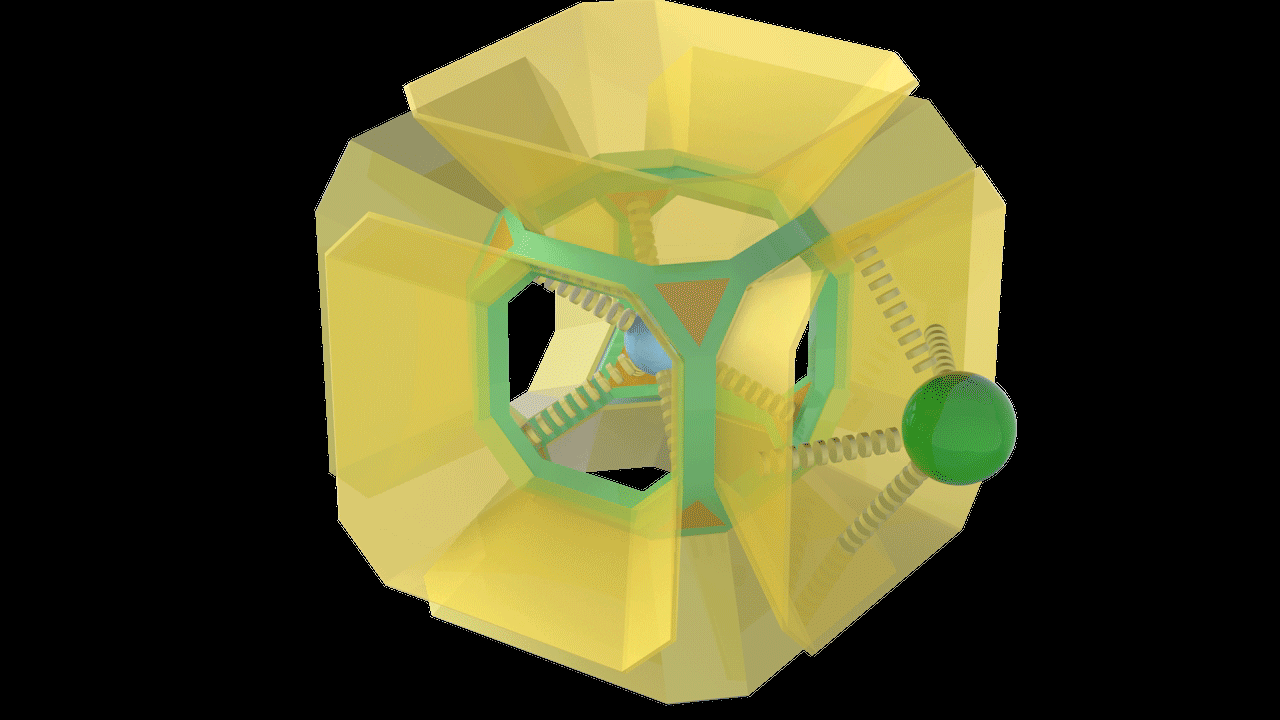

Supplement: Supplementary file 6 — Supplementary Movie 2 [file 41467_2022_34296_MOESM6_ESM.gif]
